# The Stain of the Original Salt: *Red Heats* on Chrome Tanned Leathers and *Purple Spots* on Ancient Parchments Are Two Sides of the Same Ecological Coin

**DOI:** 10.3389/fmicb.2019.02459

**Published:** 2019-10-29

**Authors:** Nicoletta Perini, Fulvio Mercuri, Maria Cristina Thaller, Silvia Orlanducci, Domenico Castiello, Valerio Talarico, Luciana Migliore

**Affiliations:** ^1^Department of Biology, Tor Vergata University of Rome, Rome, Italy; ^2^Department of Industrial Engineering, Tor Vergata University of Rome, Rome, Italy; ^3^Department of Chemical Science and Technology, Tor Vergata University of Rome, Rome, Italy; ^4^Po.Te.Co. s.c.r.l., Santa Croce sull’Arno, Italy

**Keywords:** *Halobacterium salinarum*, *red heat* deterioration, *purple spot* deterioration, tanned rawhides, leather, parchment, salt-curing of hides

## Abstract

Animal hides are one of man’s earliest and mostly used materials; many rawhide products, primarily leather, have for centuries been used for several purposes. The peculiar mechanical properties of leather depend on the hide composition, a dense collagen feltwork. Unfortunately, due to their proteic composition, rawhides may undergo microbial attack and biodeterioration. Over centuries, different processes and treatments (brining, vegetal or chrome tanning, tawing, etc.) were set up to face the biological attack and modify/stabilise the hide’s mechanical properties. Nevertheless, even present-day rawhides are subjected to biological colonisation, and traces of this colonisation are clearly shown in Chrome(III) tanned leathers (in the wet blue stage), with obvious economic damages. The colonisation traces on tanned leathers consist of isolated or coalescent red patches, known as *red heat* deterioration. Parchments are rawhide products, too; they derive from another manufacturing procedure. Even parchments undergo microbial attack; the parchment biodeterioration seems comparable to leather *red heat* deterioration and is known as *purple spots*. Recently, an ecological succession model explained the process of historical parchment *purple spot* deterioration; the haloarchaea *Halobacterium salinarum* is the pioneer organism triggering this attack. The marine salt used to prevent rawhide rotting is the carrier of haloarchaea colonisers (Migliore et al., 2019). The aim of this study was to investigate the dynamics of biodeterioration on Chrome(III) tanned leathers and its effects on the stability/integrity of collagen structure. To this end, standard cultivation methods were integrated with three updated technologies, Next-Generation Sequencing (NGS), Raman spectroscopy, and Light Transmitted Analysis (LTA). A bioinformatic comparison between chrome tanned leather vs. historical parchment colonisers was performed to evaluate if leather and parchment share common culprits; furthermore, the effect of the biodeterioration on the physical properties of the hide product was evaluated.

## Introduction

Almost all human cultures have exploited the unique properties of rawhide for millennia, developing specialised techniques to take profit from this readily available material. These properties depend on its complex structure, consisting predominantly of a dense collagen fibre network. This network is composed of hierarchical substructures (molecules, fibrils, fibres) forming the collagen tissue, a two-dimensional feltwork stabilised by molecular cross-links. Denaturation, oxidation and hydrolysis unhinge this hierarchical structure (Badea et al., 2015). Leather is the most important product obtained from processing animal rawhides, used to produce clothes, working and military equipment, household items, other objects used for education and entertainment purposes (Grömer et al., 2017). The most striking feature of leather is the ability to withstand repeating flexing without failure (Ward, 1974), and this useful feature is gained by processing hides through several techniques (Kite and Thomson, 2006). Chrome(III) tanning is the present-day dominant method in leather manufacturing (Saxena et al., 2017); chrome tanned leather has extraordinary stability after washing, an excellent softness, fullness and elasticity for making leather products (Ward, 1974; Zhang et al., 2017). These special features have substantially contributed to the success of the leather industry.

Rawhides, due to their animal origin, have a high tendency to undergo biodeterioration. Biodeterioration is defined as any unwanted change in artefact properties due to the activity of living organisms; it is responsible for an irreversible loss of value and/or information of the object (Hueck, 1968; Urzì and Krumbein, 1994).

Salting of hides has been used in the Mediterranean’s warm areas since ancient times to avoid leather biodeterioration; this treatment was/is made before any manufacturing process to store rawhides and prevent their decay (Reed, 1972). A high amount of salt (NaCl) is required to inhibit biodeterioration due to the growth of heterotrophic, environmentally available bacteria. In particular, a proportion of 40–50% salt (W salt/W hide) is required for rawhide salt-curing; the process may be performed by dry (sprinkling crystal salt) or wet salting (brining) (Kanagaraj et al., 2001).

**FIGURE 1:**
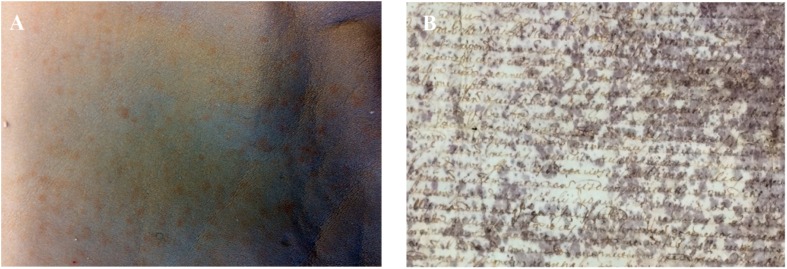
**(A)** *Red heat* deterioration in Chrome(III) tanned leather. **(B)** *Purple spot* deterioration in ancient parchment (*Faldone Patrizi A19*, Vatican Secret Archive). Photos by NP.

Unfortunately, even nowadays, chrome tanned leathers often show signs of biodeterioration, identified as *red heat* discolouration since 1929 by Jordon-Lloyd and Bergman (Bergman, 1929; Jordon-Lloyd, 1929). The *red heat* deterioration consists of isolated or coalescent red patches, clearly evident in the *wet blue* stage of the Chrome(III) tanning process (Figure 1A). The red patches have for years been supposed to be caused by halophilic Archaea (Anderson, 1954), but only recently halophilic/halotolerant microorganisms were isolated from tannery effluent (Ghosh et al., 2010).

Another rawhide product, parchment, shows signs of biodeterioration similar to the *red heat* deterioration. A frequent alteration found in ancient parchment is the so-called *purple spot* deterioration (Figure 1B). *Purple spots* are responsible for parchment discolouration and detachment of the superficial layer in the most colonised areas (Migliore et al., 2017, 2019). Two recent studies shed light on the dynamics of the *purple spot* deterioration by analysing four historical parchments kept at the Vatican Secret Archive (Migliore et al., 2017, 2019). The studies gave interesting insights into the process by identifying and demonstrating a microbial heterotrophic succession, devoted to the complete degradation and recycling of organic matter. The succession, triggered by the halophilic Archaea, *Halobacterium salinarum*, responsible of the deterioration, made clear that salting of hides was the *primum movens* of the entire process (Migliore et al., 2017, 2019); in particular, the microbial succession on the basis of parchment biodeterioration acts in two main phases. The first phase is predictable, mainly driven by the process of rawhide salt-curing, and it is common to all the damaged parchments. In fact, the marine salt used for salt-curing is responsible for conveying halophilic and halotolerant microbes adhering to salt micro-crystals. Furthermore, marine salt determines a salty environment in the hides, limiting the growth of almost all heterotrophic colonisers of the rawhides. Conversely, colonisers of the long lasting second phase of parchment heterotrophic succession (which leads to the complete degradation of the collagen) are unpredictable, with the biological attack being driven by randomly selected microbes from those available in the environment. Hence, these microbes change with the individual history of each parchment.

This study focused on three main goals: (i) definitive identification of the microbial agents of the *red heat* deterioration in chrome tanned leathers; (ii) effects of colonisation on the stability and integrity of Chrome(III) tanned leathers; and (iii) comparison between parchment *purple spots* and *red heat* deterioration agents. To these aims, we used a multidisciplinary approach. We combined standard microbiological methods (to identify in present-day rawhides living colonisers), Next-Generation Sequencing (NGS, Illumina platform, to describe the “dead” microbial colonisers of tanned leathers), RAMAN spectroscopy (to analyse the red pigments in present-day rawhides and tanned leathers), and Light Transmitted Analysis (LTA, to evaluate the deterioration degree of the collagen structure). Furthermore, updated bioinformatic and statistical analyses were used to compare the microbial colonisers of ancient parchments and present-day tanned leathers.

## Materials and Methods

### Rawhides and Leathers

Rawhides and chrome tanned hides were obtained by Polo Tecnologico Conciario PO.TE.CO. s.c.r.l. (trad. *Tanning Technology Center*)^1^ Santa Croce sull’Arno (PI), Italy. Leather samples were only bovine and included both red damaged tanned leather samples (DTLs) and undamaged tanned leather samples (UTLs). Rawhide samples included bovine rawhide (BRH) and sheep rawhide (SRH) samples, which were included in the study as a further control, as all BRHs are routinely and systematically salt-cured. Each sample was stored at 4°C in a sealed non-sterile plastic bag until processing.

### NaCl Salts

Both industrial and commercial alimentary salts were used. Industrial NaCl (Piazzolla sali^®^) was obtained from PO.TE.CO.: this is the commonly used salt for hide preservation before industrial tanning (NaCl_POTECO_). Alimentary salt (Gemma di Mare – Compagnia Italiana Sali S.P.A.), previously sterilised in a biosafety cabinet under UV lighting for 48 h (NaCl_LAB_), was used as a control salt.

### Rawhide Sample Preparation

All the BRH samples [BRH + NaCl_POTECO_] had been salt-preserved with industrial salt (from now on: NaCl_POTECO_) before their arrival in the lab; hence, for the growth experiments, a further control was introduced, consisting of unsalted SRH samples, obtained from a freshly slaughtered animal_._ Once in the lab, these samples were treated with both sterilised alimentary salts, NaCl_LAB_ [SRH + NaCl_LAB_] and industrial salt, NaCl_POTECO_ [SRH + NaCl_POTECO_]. All the salt-cured samples were stored at room temperature (25°C ± 2) in sealed non-sterile plastic bags.

### Microbiological Analyses

#### Cultivation on M97

To evaluate the presence of alive halophilic Archaea in chrome tanned and rawhide samples, 1 g of tanned or rawhide sample (three replicates) was introduced into each sterile flask containing 25 ml of homemade liquid medium #97 at 37°C. This medium (DSMZ liquid medium #97) is selective for the growth of *H. salinarum*. Solid M97 medium was also prepared. After 7 days of rotating incubation, turbidity and reddish colour of medium indicated the presence of  Halobacteria, hence haloarchaeal total count was performed (see section “Haloarchaea Growth”).

To evaluate the presence of alive halophilic Archaea in salt, NaCl_LAB_ and NaCl_POTECO_ were utilised in the recipe to prepare M97: 250 g of each salt was added to the medium after its autoclave sterilisation. As described before, turbidity and reddish colour of medium indicated Haloarchaea growth, which were measured as total count.

To have a reference for haloarchaeal growth, *H. salinarum* (reference strain DSM 3754) was grown on both liquid and solid M97 medium.

#### Haloarchaea Growth

To evaluate the haloarchaeal count (both reference strain and lab isolates), cultures were incubated at 37°C for 7 days on liquid media; 10 μl of each microbial suspension were adjusted to 0.5 McFarland standard (O.D. = 0.14) and inoculated at different dilutions (from 10^–2^ up to 10^–14^, geometric progression 2) on solid M97. Plates were then incubated at 37°C in a humidified chamber, and colony-forming units (CFU) were counted after 21 days; 0.5 McFarland microbial suspensions (O.D._600 nm_ = 0.14) corresponded to 10^8^ CFU/ml.

### Metagenomics

#### Bacterial Pellet and DNA Extraction

A piece (0.25 g) from each rawhide (BRH) or tanned leather (DTL and UTL) was processed by using the Power Soil^®^ DNA isolation kit (Mo Bio, Carlsbad, CA, United States) according to the manufacturer’s guidelines; DNA extraction was performed in three replicates for each sample.

#### Illumina Paired-End Sequencing

PCR amplification of the highly phylogenetically variable region V4 of 16S rRNA gene (Caporaso et al., 2011) was performed by using the almost universal primers 515F (forward, 5-GTGCCAGCMGCCGCGGTAA-3) and 806R (reverse, 5-GGACTACHVGGGTWTCTAAT-3). These primers had been employed in the Earth Microbiome Project^2^ in order to anneal a wide range of bacterial and archaeal 16S sequences, except for the SAR11 clade and some Crenarchaeota/Thaumarchaeota (Hugerth et al., 2014; Apprill et al., 2015). As a test performed with the Probe Match tool in RDP found out (Cole et al., 2009), this primer pair matches the Euryarchaeota (515F = 0.95; 806R = 0.95) and even better the Halobacteria (515F = 0.97 and 806R = 0.96), which were actually looked for, according to the Raman results (Migliore et al., 2019). The NGS output showed significant differences among the communities of the three sets of samples, better highlighted by the taxonomical assignation of the operational taxonomic units (OTUs) and the relative frequencies of the different microbial phyla and orders. The amplification consisted of a 30-cycle PCR, using the HotStarTaq Plus Master Mix Kit (Qiagen, United States) under the following conditions: 94°C for 3 min, followed by 28 cycles of 94°C for 30 s, 53°C for 40 s and 72°C for 1 min, final elongation step at 72°C for 5 min. After amplification, PCR products were checked in 1% agarose gel and purified. Pure DNA amplicons were sent to the Molecular Research LP in Shallowater, TX, United States (MR DNA^3^, Shallowater, TX, United States) to prepare DNA library by following Illumina TruSeq DNA library preparation protocol (Illumina TruSeq^®^ DNA Sample Preparation Guide. ©2011–2012 Illumina, Inc.). Sequencing was performed on a MiSeq following the manufacturer’s guidelines.

#### Illumina Paired-End Sequencing Data Processing

The metagenomic analysis of raw DNA sequencing data was performed in house by using Quantitative Insights Into Microbial Ecology (QIIME) 1.1.9, an open-source bioinformatics pipeline. Briefly, raw DNA sequencing data in the form of full.fasta and full.qual files were subsequently converted into.fastq files using the free software available on www.mrdnafreesoftware.com. After the mapping file validation, the pipeline performed a quality filtering (only sequences >150 bp and without ambiguous base calls are retained) and barcode extraction. To standardise the differences between the samples, the whole dataset was normalised by random subsampling to the lowest number of reads/sample, i.e., the UTL_3 sample, at the common depth of 62,624 reads. The V4 sequences were clustered into OTUs at 3% divergence (97% similarity) by using two similarity algorithms implemented in QIIME, Usearch61, and Blast. Then, a representative sequence from each OTU cluster was chosen and used for the OTU identification, using RDP Classifier and a reference taxonomic database derived from Greengenes^4^ (DeSantis et al., 2006, respectively). The taxonomic annotations were used to compare the microbial communities of the different samples; principal coordinates analysis (PCoA) plots were generated on Bray–Curtis distance metrics. Only 3 OTUs (72 sequences) were unclassified, 4 were mitochondria and 2 chloroplasts (91 and 43 sequences, respectively). The complete set of assembled 16S RNA gene sequences obtained in this study has been deposited in GenBank under the study accession no. SUB5675294, MN023317 – MN025256.

#### Bacterial Community Analyses

Microbial community structure and composition (normalised OTU dataset) were analysed in QIIME as follows: (i) rarefaction curves were built to evaluate differences in sampling effort (Supplementary Figure S1); (ii) Shannon index (H’) was used to evaluate bacterial community diversity; (iii) Venn diagram was used to quantify the shared/unshared OTUs in sets of samples; and (iv) pie charts were built to visualise the bacterial community composition.

#### Statistical Analyses

Principal coordinates analysis and ANOSIM, UniFrac unweighted and PERMANOVA on the OTU dataset were performed in QIIME. PCoA ordination was conducted on a Bray–Curtis distance matrix calculated between sampling plots with log (*x* + 1)-transformed OTUs abundance data. ANOSIM (*n* = 99 randomisations; Clarke and Warwick, 2001) and PERMANOVA with Bonferroni correction (*n* = 999 permutations) were employed to test for significant differences in microbial communities between bovine red damaged and undamaged tanned leather (UTL) samples, between the pooled dataset of tanned leathers and BRH samples, and among all samples belonging to the entire dataset. Significance (ANOSIM, *p* > 0.05) was calculated by permutation test with pseudo F-ratio. The UniFrac unweighted algorithm was used to evaluate if the microbial community structure and composition of the three sets of samples, based on their phylogenetic relationships and the abundances of taxonomic groups, were statistically significant at *p* < 0.05 (Lozupone and Knight, 2005; Lozupone et al., 2011).

### Raman Spectroscopy

Raman analyses were performed on both the pigments of alive haloarchaea and the pigments extracted from red damaged tanned leathers (DTLs). The reddish colonies of alive haloarchaea grown on M97 medium derived from (i) SRHs treated with industrial salt (SRH_+_ NaCl_POTECO_), (ii) BRHs treated with industrial salt (BRH + NaCl_POTECO_), (iii) industrial salt (NaCl_POTECO_), and (iv) reference strain, *H. salinarum* DSM 3754. The pigments of the alive haloarchaea were directly analysed. Pigments of the red damaged tanned leathers (DTLs) were chemically extracted from the red patches. Raman analyses were performed by using a micro-Raman spectrometer explora system (Horiba) with a laser source at 532 nm and power less than 1 mW, 100× magnification and 3 cm^–1^ spectral resolution, as already used in Migliore et al. (2019).

### Light Transmission Analysis

The induced hydrothermal denaturation of the collagen populations in the chrome tanned leather samples was investigated by means of the LTA technique, as already described in Migliore et al. (2019).

## Results

### Microbiological Analyses

#### Microbial Growth in M97

Standard cultivation methods in liquid M97 allowed increasing reddening and turbidity of the media due to *H. salinarum* growth after 7 days to be observed. The reference strain (*H. salinarum* DSM 3754), NaCl_POTECO_ salt-cured rawhides (BRH + NaCl_POTECO_; SRH + NaCl_POTEC__O_) and NaCl_POTECO_ alone gave positive cultures; no growth was observed for NaCl_LAB_ salt-cured rawhides (SRH + NaCl_LAB_) and for chrome tanned leather (DTL; UTL) samples (Supplementary Figure S2).

#### Microbial Community Analyses

QIIME bioinformatic analysis of BRHs and chrome tanned leather, including both red damaged tanned leather (DTL) and UTL, produced a total of 1,098,435 bacterial and haloarchaeal sequences, assigned to a total of 1,945 OTUs. On average, BRH yielded 549,285 microbial sequences assigned to 1,661 OTUs, DTL samples yielded 339,750 microbial sequences assigned to 1,713 OTUs, while UTL samples yielded 209,400 microbial sequences assigned to 1,142 OTUs (Table 1).

**TABLE 1:** Bovine rawhides (BRHs), red damaged tanned leathers (DTLs), and undamaged tanned leathers (UTLs) sample yields from each replicate (#1, 2, 3).

**Replicate**	**BRH**	**DTL**	**UTL**
#1	170,617	116,256	83,293
#2	133,185	114,732	63,483
#3	245,483	108,762	62,624
Total sequences	549,285	339,750	209,400

To standardise differences among samples, the dataset was normalised to the lowest common depth of 62,624 sequence reads per sample (sample UTL#3) by random subsampling. The rarefaction curves, built to evaluate differences and efficiency in the sampling effort, confirmed that the sequencing coverage was good (Supplementary Figure S1).

The Shannon diversity index (H’) on the entire dataset was comparable in all samples: 4.6 ± 0.2 in DTL, 4.3 ± 0.1 in UTL, and 4.2 ± 0.1 in BRH.

**FIGURE 2:**
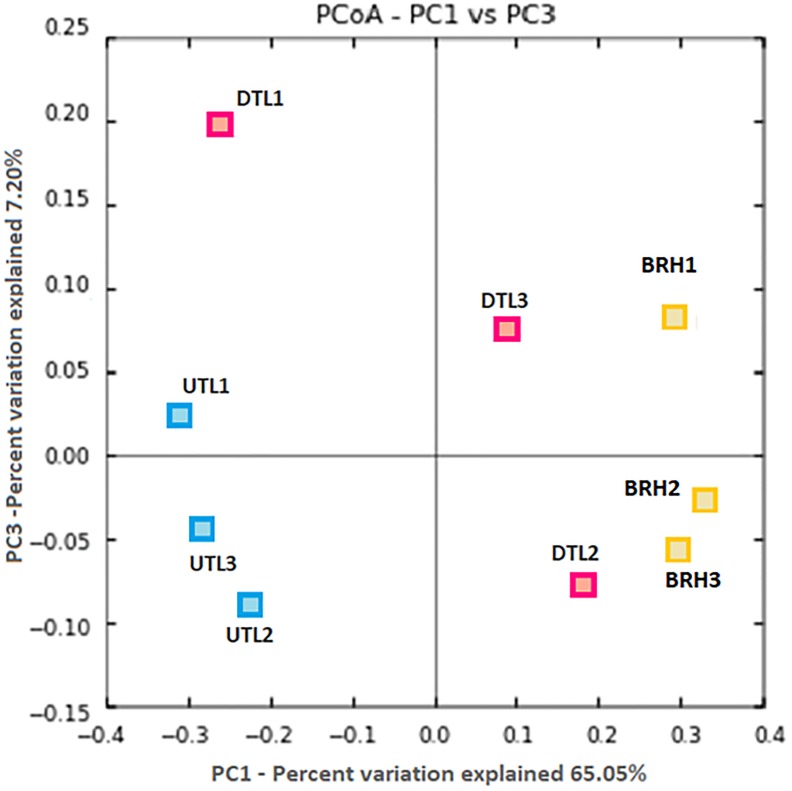
PCoA based on Bray–Curtis distances shows clear patterns of separation in the microbial composition associated to the three replicates (#1, 2, 3) of each sets of samples, including red damaged tanned leather (DTL) and undamaged tanned leather (UTL), and bovine rawhides (BRHs).

Principal coordinates analysis plot compared the microbial community composition of tanned leathers (DTL and UTL) and rawhides (Figure 2). UTL and BRH are quite homogeneous and clearly separated, while DTL are intermediate among the other two batches more disperse. ANOSIM analysis confirmed that differences were statistically significant (99 permutations, *p* < 0.05) if the entire dataset was analysed (*p* < 0.05), or in the comparison UTL vs. BRH (*p* < 0.05). All the other comparisons did not highlight statistically significant differences (99 permutations, *p* > 0.05; DTL + UTL vs. BRH = n.s., DTL vs. UTL = n.s., BRH vs. DTL = n.s.).

**FIGURE 3:**
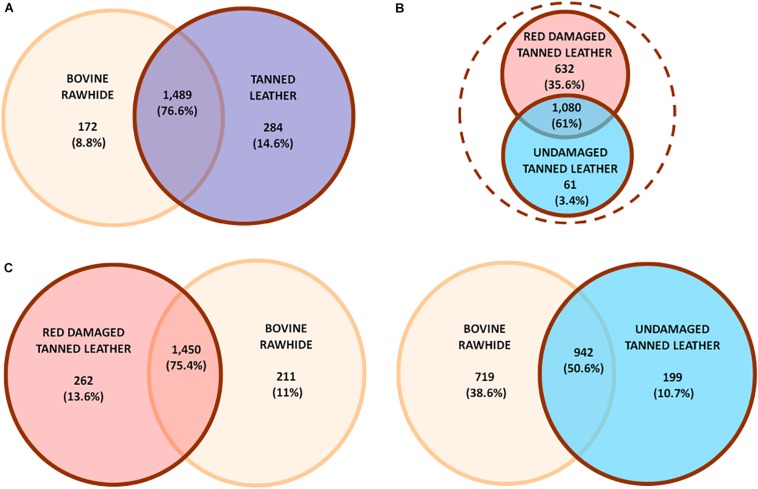
Unique or shared OTUs in **(A)** comparison between bovine rawhides (BRHs) (orange) and tanned leather samples (blue), these last included both red damaged (DTL) and undamaged tanned leathers (UTL); **(B)** comparison within tanned leather sample subset: DTL (red) vs. UTL (light blue); (**C**, left) comparison between DTL (red) and BRHs (orange); (**C**, right) comparison between BRHs (orange) and UTL (light blue). Number and percent of OTUs are reported.

The Venn diagrams in Figure 3 show the different distribution of OTUs among rawhide (found positive to Halobacteria growth) and tanned leather (DTL and UTL) samples. In particular, the first comparison (Figure 3A) highlights that the great majority of OTUs (1,489 OTUs, 76.6% of the OTUs; accounting for 1,090,688 sequences, 99.3% of all sequences) were shared between BRH and the tanned leather sample dataset. Comparable numbers of unique OTUs were found in BRH (172 OTUs, 8.8% of the OTUs; accounting for 2,228 sequences, 0.2% of all sequences) and in tanned leather samples (284 OTUs, 14.6% of the OTUs; accounting for 5,519 sequences, 0.5% of all sequences). As already reported, the tanned leather dataset included both DTL and UTL samples: the comparison of these subsets of data (Figure 3B) show that even in these samples the majority of OTUs were shared (1,080 OTUs, 61% of the OTUs; accounting for 538,565 sequences, 98.1% of the sequences in the subsets), but UTL samples included only 61 unique OTUs (3.4% of the OTUs), accounting for 644 sequences (0.1% of the sequences), one order of magnitude lower than those found in DTL, 632 OTUs (35.6% of the OTUs), accounting for 9,941 sequences (1.8% of the sequences).

While comparing each of the two tanned leather datasets to the rawhide one, a clear difference was appreciated (Figure 3C): DTL and BRH shared the great majority of OTUs (1,450 OTUs, 74.5%; accounting for 882,548 sequences, 80.3% of all sequences; Figure 3C, left) while UTL shared only about 50% OTUs with BRH (942 OTUs, 50.6%; accounting for 715,810 sequences, 50.6% of the sequences; Figure 3C, right). Furthermore, BRH samples had a comparable number of unique OTUs with DTL (211 vs. 262 OTUs, respectively), while they had about a three times higher number of OTUs compared to UTL (719 vs. 199 OTUs, respectively). The complete list of OTUs is reported in Supplementary Table S1.

**FIGURE 4:**
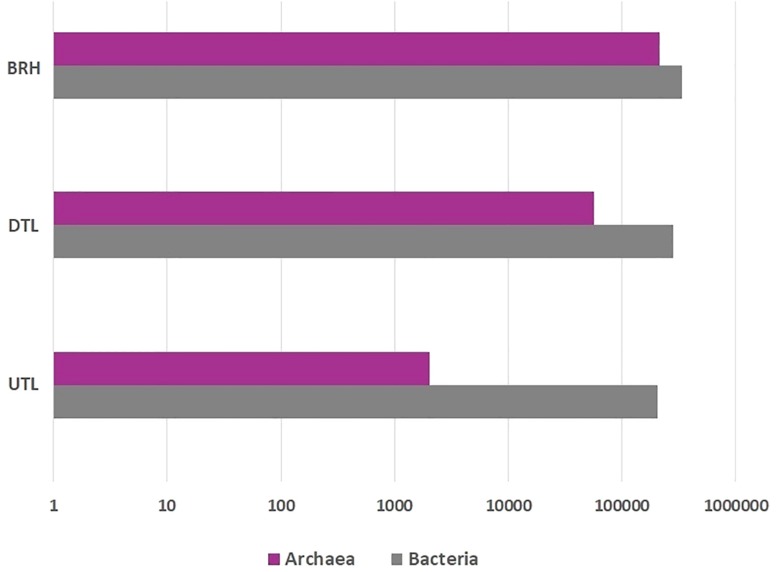
Archaeal (purple), bacterial (grey) sequence distribution among bovine rawhides (BRHs), red damaged tanned leathers (DTLs), and undamaged tanned leathers (UTLs).

The OTUs were assigned to both Archaea and Bacteria in all samples (Figure 4; Supplementary Table S2). Archaea accounted for a total of 270,464 sequences assigned to 505 OTUs (212,077 sequences in BRH; 56,361 in DTL and 2,026 in UTL). Bacteria accounted for a total of 827,796 sequences, assigned to 1,431 OTUs (337,195 sequences in BRH; 283,344 in DTL and 207,360 in UTL). Only 3 OTUs (72 sequences) were unclassified, 4 were mitochondria, and 2 chloroplasts (91 and 43 sequences, respectively).

Among Archaea, Euryarchaeota was the major component of microbial colonisers in BRH (99.1%) and in DTL (96.5%); it had a slight prevalence even in UTL (41.3%) where the major component was Crenarchaeota (58.3%). The class Halobacteria were prevalent in both BRH (96.7%) and DTL (95.1%), while in UTL samples they were found at 30%. Halobacteria were entirely represented by the order Halobacteriales (95.8% of the sequences), among them *Halobacterium* and *Halorubrum* were the most represented genera. *H. salinarum* represented 29.6% sequences in BRH samples, 32.1% in DTL samples, and 8.2% in UTL samples.

Among bacteria, Proteobacteria and Firmicutes were observed in all samples. Gamma-Proteobacteria were dominant in BRH (73.3%), DTL (54.2%), and UTL (52.9%). Bacilli were mainly found in DTL (31.9%), UTL (37.3%) and less represented in BRH (13%). The rare component belonged to the classes Bacteroidetes (from 8% in BRH to 0.5% in UTL) and Actinobacteria (from 1% in BRH to 0.3% in UTL).

**FIGURE 5:**
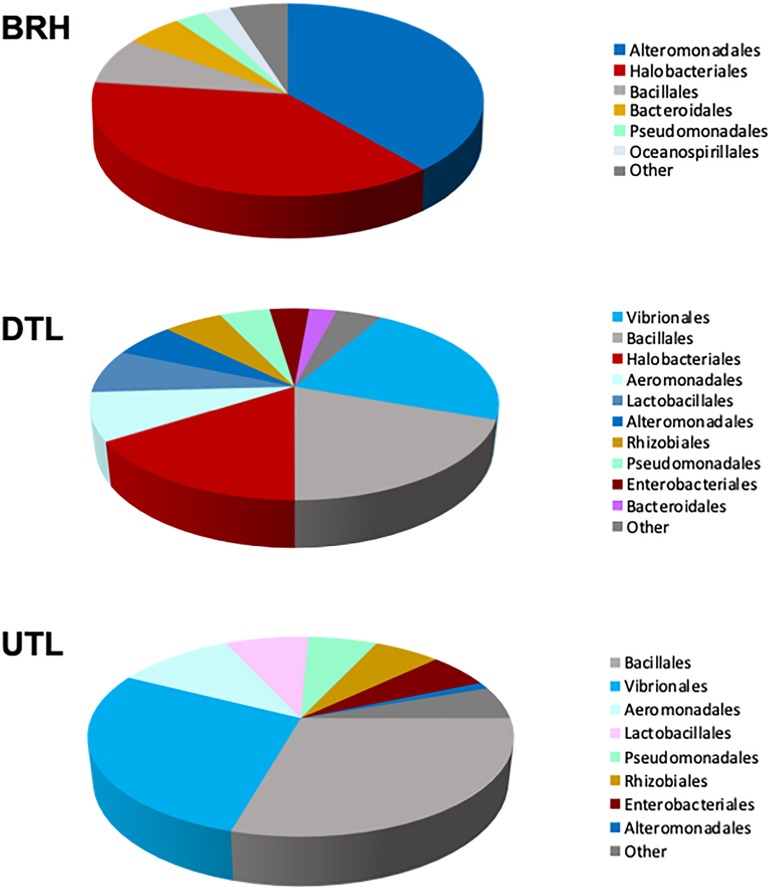
Composition of the microbial communities, as percentage of sequences per taxonomic group, in rawhide (BRH), red damaged tanned leather (DTL), and undamaged tanned leather (UTL).

The Figure 5 shows how OTUs are partitioned among the three sets of samples. It is worth noting that Halobacteriales and Alteromonas are found in BRH and DTL, Aeromonadales and Vibrionales are found in DTL and UTL, while other taxa, such as Bacillales, Bacterioidales, and Pseudomonadales, are common to the three sets of samples.

The most abundant orders found in each of the three datasets, the “top 70%,” are clearly different. The top 70% is composed of Halobacteriales and Alteromonadales in BRH samples (38.4% and 38.5%, respectively); Vibrionales, Bacillales, Halobacteriales, and Aeromonadales (22.1, 19.5, 16.0, and 8.1%, respectively) in DTL samples; and Bacillales, Vibrionales, Aeromonadales, and Lactobacillales (29.4, 28.1, 10.6, and 7.5%, respectively) in UTL samples. Accordingly, UniFrac unweighted and PERMANOVA (with Bonferroni correction, 999 permutations) showed significant differences when the three sets of samples are compared (UniFrac unweighted: *p* < 0.05; PERMANOVA: *p* = 0.01), because of the significant differences between BRH and UTL (UniFrac unweighted: *p* < 0.05; PERMANOVA: *p* = 0.03); no significant differences were found in the other pairing tests (BRH vs. DTL; DTL vs. UTL; DTL + UTL vs. BRH).

### Chemical Analyses

#### Raman Spectroscopy

**FIGURE 6:**
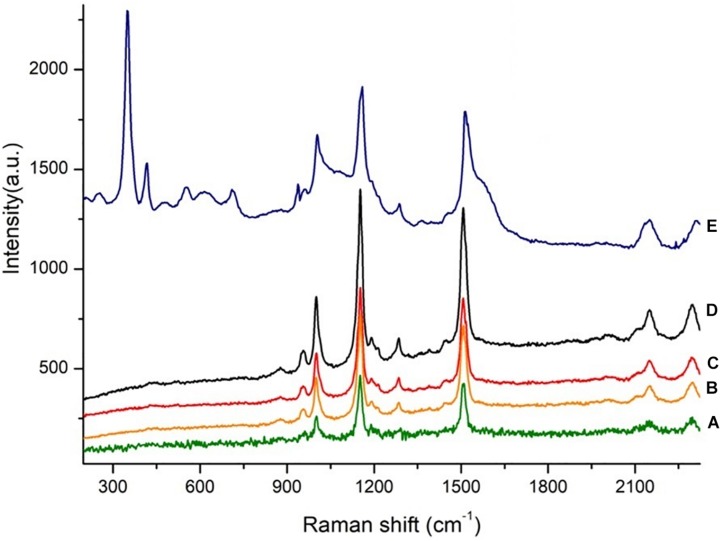
Raman spectra of red colonies deriving from (a) *H. salinarum*; (b) salted sheep rawhides [SRH + NaCl_POTECO_]; (c) salted bovine rawhides [BRH + NaCl_POTECO_]; (d) NaCl_POTECO_; and (e) red damaged tanned leathers (DTLs).

In Figure 6, Raman spectra of reddish pigments extracted from (a) red colonies of *H. salinarum* DSM 3754 grown on M97, (b) salted sheep (RH_S_ + NaCl_POTEC__O_) and (c) bovine (RH_*S*_ + NaCl_POTECO_) rawhides, and (d) industrial salt (NaCl_POTECO_) isolated haloarchaea are reported. The pigments extracted from (e) red damaged tanned leathers (DTL) were directly analysed. The spectra are strongly similar and the main peaks at 1,506, 1,152, and 1,001 cm^–1^ of all the analysed samples can be attributed to the α-bacterioruberin, the principal carotenoid present in *H. salinarum* cells (Marshall et al., 2007; Fendrihan et al., 2009; Jehlička et al., 2013). According to the literature (Vaskova, 2016), the main peaks in the range 300–900 cm^–1^ are due to the presence of chromium used in tanning.

### LTA Analysis

#### Denaturation Analysis

**FIGURE 7:**
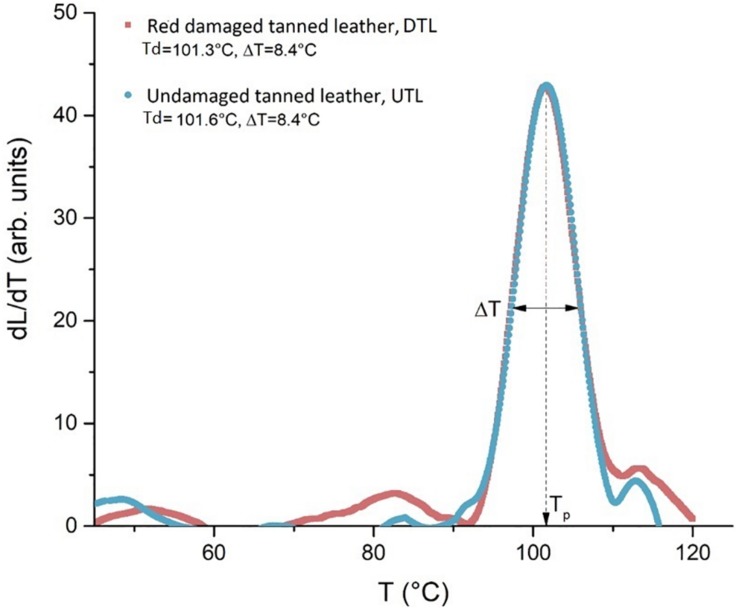
The plot of the quantity dL/dT describes the intensity of the denaturation process as a function of temperature: the similar profile shown by the LTA curves indicates an analogous structural stability of the collagen in red damaged (DTL) and undamaged tanned leathers (UTLs).

As shown in Figure 7, the LTA analysis of the collagen hydrothermal denaturation process in red damaged tanned leathers (DTL) and UTLs did not show any differences in the temperature of the peaks *T*_d_ (101.3°C in DTL and 101.6°C in UTL samples) or in their width *T*_1__/__2_ (8.4°C in both DTL and UTL samples). This indicates that the micro-structural chemical stability and its homogeneity are the same for the two samples.

## Discussion

In this study, the dynamics of *red heat* biodeterioration of Chrome(III) tanned leathers has been revealed by unequivocally identifying the microbial responsible – haloarchaea, mainly *H. salinarum* – and the source of these microbes – the salt used in the brining of rawhides. The haloarchaea are found to be the major component of the microbial assemblages in red damaged chrome tanned leathers while they are rare/absent in the undamaged ones. Salt has been unequivocally demonstrated as the source of the halophilic microorganisms; these microbial colonisers of the rawhides, indeed, are absent in the leathers cured with sterilised salt.

In this study, the metagenomic analyses demonstrated that all the samples which had been salt-cured with industrial salt, i.e., BRHs and DTL or UTL, showed the presence of haloarchaeal DNA: Halobacteriales were dominant in BRH and DTL (38.4% and 16.0% of the sequences, respectively). They were present, at a very low percentage, even in UTL (0.3% sequences).

The standard cultivation methods, with the selective medium M97, demonstrated the presence of live *H. salinarum* in both industrial salt and industrial salt-cured rawhides. Hence, the salt used since ancient times to preserve the hides (by inhibiting the growth of environmental microbes because of the high salinity) is the *reservoir* and the vehicle of the Halobacteriales colonisers. Haloarchaea-contaminated industrial salt triggers their (slow) growth on the surface of the hide on the flesh side. As shown in Supplementary Figure S3, a transversely cut sample of BRH clearly shows that, after 3 months of incubation in the lab, the reddish discolouration is limited to the superficial layer of the flesh side. Conversely, both sterilised salt and sterilised salt-cured rawhides did not contain live *H. salinarum*. In fact, sterilised salt-cured hides did not show any growth of haloarchaea (Supplementary Figure S2).

The Raman spectroscopy confirmed these data by detecting the halobacterial bacteriorhodopsin, the purple transmembrane protein containing retinal; these pigments were clearly found in the red patches of red damaged tanned leathers, where NGS demonstrated the presence of Haloarchaea, just as in the colonies harvested from standard cultivation methods of industrial salt-cured leather and industrial salt samples. These results unequivocally confirmed that haloarchaea are responsible for *red heat* deterioration and that salt is their vehicle of colonisation.

As a last point, neither DTL nor UTL were positive for the growth of *H. salinarum* with standard cultivation methods. As expected, this result demonstrated that chrome tanning, among other effects, is able to tear down the microbial load of the hides.

In the last decades, *red heat* deterioration of hides has been investigated, although a definitive answer had never been found – Anderson (1954) tried to preliminarily characterise the halophilic microbes, from red deteriorated salted hides, by standard microbiological techniques; [opetwcite]B6,B5[clotwcite]Birbir et al. (1996); Birbir and Bailey (2000) succeeded in growing halophilic and halotolerant microorganisms on brine cured cattle hides without a specific taxonomic identification; and Vreeland et al. (1999) studied the red colour found on *red heat* deteriorated hides attributing it to pigments of halophilic bacteria, without deepening the microbial taxonomic aspects.

NGS also revealed all the other microbial colonisers of rawhides and tanned leathers. The microbial colonisers, accounting for 1945 OTUs, were differently partitioned among the three batches. Venn diagrams demonstrated that the great majority of OTUs were shared between rawhides and DTL/UTL samples, and they shed light on the differences in the rare components. In fact, the red damaged and UTL harbour different communities: the unique OTUs in red damaged samples are one order of magnitude higher than in undamaged tanned ones, as the colonisation itself probably triggers a microbial succession which allows a higher colonisation rate, by producing a different environment in the damaged areas. Furthermore, even the comparison of rawhides with DTL/UTL shows clear differences, with the undamaged samples being the less colonised.

The “top 70%” sequences in the different batches revealed that the most prevalent microbes were Alteromonadales, Halobacteriales, Lactobacillales, Pseudomonadales, and Vibrionales as the most abundant orders. Interestingly, the “top 70%” confirmed the dominance of Halobacteriales in rawhides and red damaged tanned samples, and their absence in the UTL batch. Strictly halophilic Halobacteriales and Alteromonas are dominant in BRH and DTL, while halotolerant marine Aeromonadales and the marine/brackish Vibrionales are found in DTL and UTL, with Vibrionales being prevalent in UTL; other taxa such as Bacillales, Bacterioidales and Pseudomonadales are common to the three sets of samples. This progressive modification of the microbial community must depend on the presence of salt: rawhides are the saltiest environment of the three sample sets due to routine salt-curing preservation. Marine salt is produced in saltworks based on evaporation ponds – shallow, lined earthen basins in which concentrate evaporates naturally as a result of solar irradiation. As fresh water evaporates from the ponds, the minerals in the concentrate are precipitated in salt crystals, which include the microbial coloniser of the ponds, halophilic and halotolerant microorganisms (archaea, marine bacteria and their resting phases); so, salt must be the mark of the “original stain.” The actual importance of salt as the *reservoir* and vehicle for halophilic and marine bacterial colonisers is also stressed by the high percentage of Alteromonadales (38.5% of the sequences) observed in the salty environment of BRH samples, directly sprinkled with high amount of salt. Alteromonadales sharply decrease in DTL and UTL (5 and 1.1%, respectively) after the pickling, which makes the environment less salty.

The reduction of salinity and the toxic conditions created in the leather environment during the phases of leather processing should easily rid of Halobacteria, which are more Na+ requiring than Alteromonadales. This is clearly indicated by the reduction of DNA traces of Halobacteria in Chrome(III) tanned samples, if compared to rawhides. Nevertheless, it could be hypothesised that the salt-curing process may allow the entrance and embedding inside the hide of microscopic salt crystals carrying Halobacteria. On these microcrystals they could persist, and the *red heat* would occur wherever the salt-associated Halobacteria grew to be later killed and lysed, *in situ*, by the treatment, the lysed Halobacteria colonies being responsible for the *red heat* discolouration. The rare DNA traces of Haloarchaea found even in undamaged samples may derive from the very high abundance of haloarchaea in the colonised rawhides, which can be a source of DNA contamination for the entire tannery.

The DTL and UTL shelter different communities; hence, a different structural damage of collagen fibres due to the microbial colonisation was hypothesised in the two leather batches. However, LTA analysis highlighted that the denaturation temperature of the collagen fibres was the same in DTL and UTL (101.3 and 101.6°C, respectively). These data are in strict agreement with what is reported in the literature ones, a TS of 100–120°C for Chrome(III) tanned leather (Kite and Thomson, 2006). In addition to the identical mean thermal stability between the two kinds of sample, proved by the same *T*_d_ values, the same width of the peaks (8.4°C in both batches) also reveals an identical spectrum of the stability, i.e., the same micro-structural homogeneity, for both batches. The tanning process makes high and homogeneous the stability of collagen fibres by creating new crosslink bonds in the collagen triple helix, regardless of the initial and superficial microbial attack (Figure 7).

### Comparison Between Leather Red Deterioration and Parchment Purple Spots

Recently, Migliore et al. (2017, 2019) applied a metagenomic approach to study the *purple spot* deterioration on another hide product, historical parchments, and identified *H. salinarum* as the pioneer species of an ecological succession responsible for the parchment *purple spots*.

Parchment *purple spots* have been shown to be triggered by haloarchaeal colonisation (Migliore et al., 2019) as we demonstrated, in this study, for *red heat* deterioration of Chrome(III) tanned leathers.

Hence, the comparison of the colonisers found in damaged Chrome(III) tanned leathers vs. damaged historical parchments (analysed by the same NGS platform, using the same PRC primers) shows that they share the pioneer species, *H. salinarum*, which triggers the ecological succession. It is worth noting that only the first phase of the succession can be traced in salt-cured rawhides, because hides are rapidly tanned, and the Chrome(III) tanning procedure makes toxic the environmental condition within the hide, killing all the microorganisms and definitively interrupting the succession. This “chemical sterilisation” inhibits the biological attack and limits the damage to the collagen structure.

Conversely, the parchment manufacturing procedure prevents the growth but does not kill the microbes within the hide, keeping the halotolerant and halophilic ones in a quiescent state until moisture, temperature, and light change, allowing their growth. The unicity of the pioneer coloniser is confirmed by Raman spectroscopy, showing the same pigment array in all the red/purple spotted samples.

**FIGURE 8:**
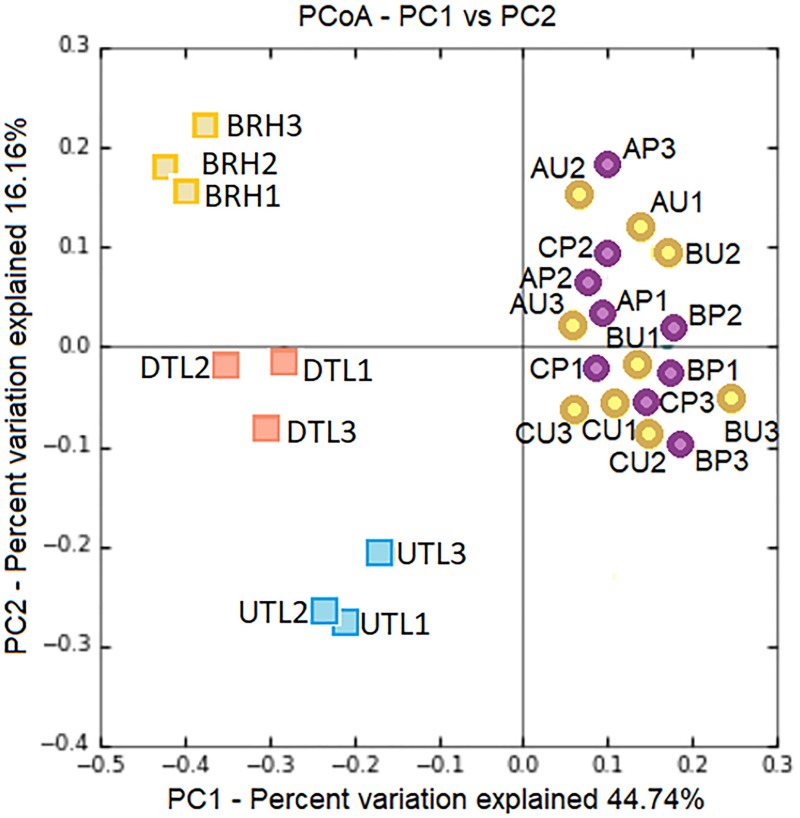
PCoA based on Bray–Curtis distances shows clear patterns of separation by microbial composition among red damaged/undamaged tanned leathers (DTL/UTL), bovine rawhides (BRHs), three purple damaged/uncoloured-less damaged parchments (AP, BP, CP/AU, BU, CU). The number of each replicates (#1, 2, 3) is reported. The plot was generated on the base of an OTU table, Supplementary Table S2, after concatenating seqs.fna files from the two projects (cat file1_seqs.fna file2_seqs.fna > combined_file_seqs.fna), mapping file merging (merge_mapping_files.py) and validation (validate_mapping_file.py), and dataset normalisation.

Therefore, the manufacturing procedure and the history of each hide product affect the structure and composition of haloarchaeal/bacterial colonisers, as shown in Figure 8. Although *H. salinarum* is the pioneer species of the succession in both leather and parchment, a great extent of variation (44.74%, PC1 axis) is explained by the manufacturing procedure, which determines the dynamics and composition of the microbial colonisers.

The combined effect of microbial damage and the manufacturing procedure determines the final thermal and chemical stability of the hide product. Microbial damage affects the denaturation temperature, which is different in purple-damaged and uncoloured-less damaged areas of the same parchment. On the contrary, Chrome(III) tanning nullifies the differences in the denaturation temperature between red-damaged and UTLs by creating new crosslink bonds in the collagen triple helix, regardless of the microbial damage. These new bonds between adjacent collagen molecules both repair the damaged structures and confer resistance to further microbiological attack (Kite and Thomson, 2006).

Hence, the parchment thermal stability strictly depends on the microbial colonisation of each document – only in *purple spots* does microbial colonisation over time make the collagen structure “crumbly,” which is much more stable in undamaged areas (Migliore et al., 2017, 2019); conversely, the early chrome tanning makes the stability of collagen fibres high and homogeneous.

In conclusion, both the *purple spots* and the *red heat* deterioration depend on the pioneering *H. salinarum* colonisation, producing the reddish spot discolouration due to bacteriorhodopsin. The marine salt used for the hide-salt curing is responsible for conveying the halobacteria into hides. Halobacteria are able to damage the hide structure by utilising the collagen fibres as nutrients. Then, the hide manufacturing procedure to get parchment or tanned leather affects the survival of *H. salinarum*. Parchment treatment does not kill the halotolerant and halophilic microbes present within the inner portion of the salt-cured hide, but they remain in a quiescent state until the environmental conditions (moisture, temperature, and light) are limiting. Unfortunately, these colonisers are a kind of time bomb for parchment, determining its damage entity and fate. On the contrary, chrome tanning kills all the microbes present on and within the hides. Furthermore, chrome tanning is able to introduce new crosslink bonds, chemically stabilising the collagen and making the leather products useful and persistent. These analyses can promote further studies on the complex dynamics on the basis of hide product biodeterioration and help in avoiding leather colonisation and biodeterioration.

## Data Availability Statement

The complete 16S RNA gene sequence dataset (SUB5675294, MN023317–MN025256) for this study can be found in the GenBank repository.

## Author Contributions

NP and LM conceived, designed the study and sampling strategy, performed the sampling, and prepared the first draft of the manuscript. DC and VT chose and provided the bovine/sheep rawhides and Chrome(III) tanned leathers. NP performed the microbiological analyses, DNA extraction, PCR amplification, QIIME bioinformatics, and statistical analyses. MT performed the qualitative analysis of the microbial community. FM performed the LTA analyses. SO performed the Raman analyses. NP, LM, and MT edited and reviewed the draft to obtain the final manuscript, which has been discussed and approved by all the authors.

## Conflict of Interest

DC and VT were employed by Po.Te.Co. s.c.r.l. The remaining authors declare that the research was conducted in the absence of any commercial or financial relationships that could be construed as a potential conflict of interest.

## Footnotes

1 www.polotecnologico.com

2 www.earthmicrobiome.org

3 http://www.mrdnalab.com/

4 http://rdp.cme.msu.edu

## Abbreviations

BRH: bovine rawhide
DTL: red damaged tanned leather
SRH: sheep rawhide
UTL: undamaged tanned leather
