# Supplementary material for: The Stain of the Original Salt: Red Heats on Chrome Tanned Leathers and Purple Spots on Ancient Parchments Are Two Sides of the Same Ecological Coin
Source: Front Microbiol. 2019 Oct 29;10:2459. doi: 10.3389/fmicb.2019.02459 (PMC6828845; doi:10.3389/fmicb.2019.02459)
Supplement: Supplementary file 1 [file Data_Sheet_1.docx]

Supplementary Material

# Supplementary Figures


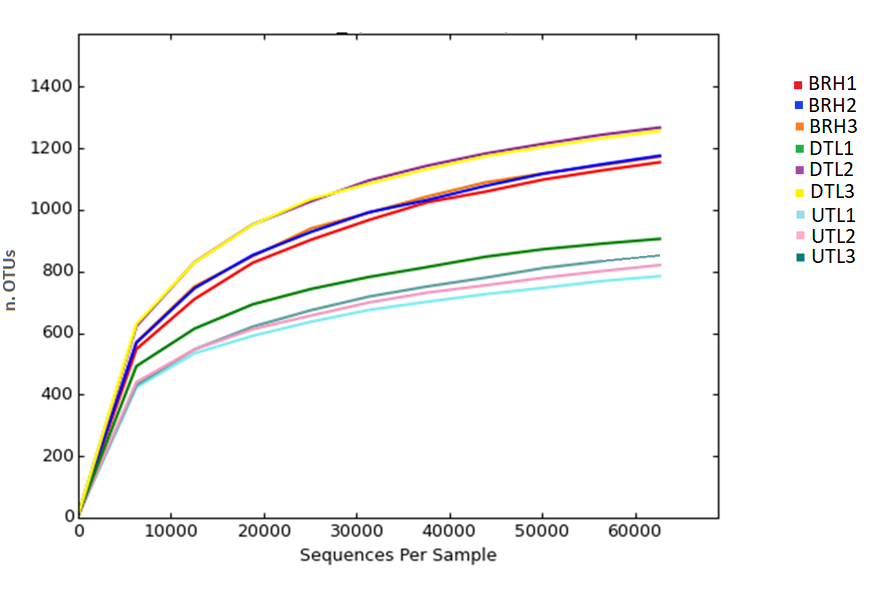


## Supplementary Figure 1. Rarefaction curves built on each replicate sample (#1, 2, 3) from red damaged tanned leathers (DTL), undamaged tanned leathers (UTL) and bovine rawhides (BRH).


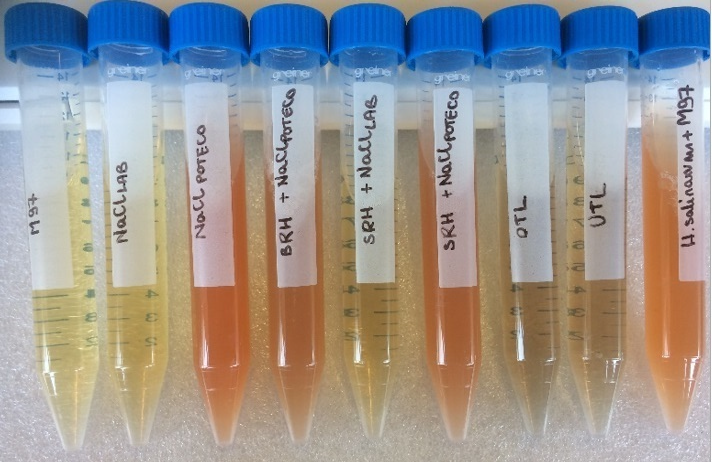


## Supplementary Figure 2. Haloarchaeal growth in liquid M97 after 7 days of incubation at 37 °C. The image shows the reddening and roiling of liquid M97 medium under the different treatments. NaCl_LAB_ = sterilized alimentary salt; NaCl_POTECO_ = not sterilized industrial salt; BRH = bovine rawhides; SRH = sheep rawhides; DTL = red damaged tanned leather; UTL = undamaged tanned leather. Photo by N. Perini.

| 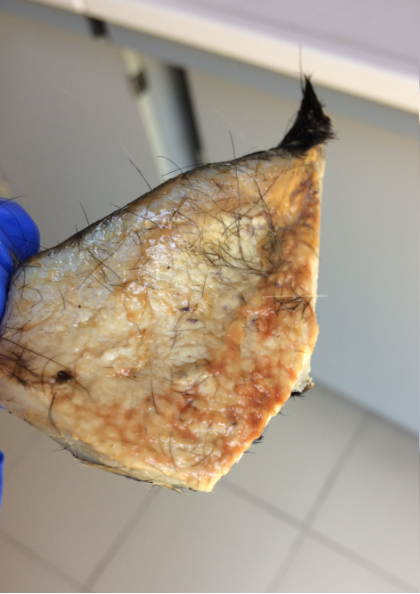 | 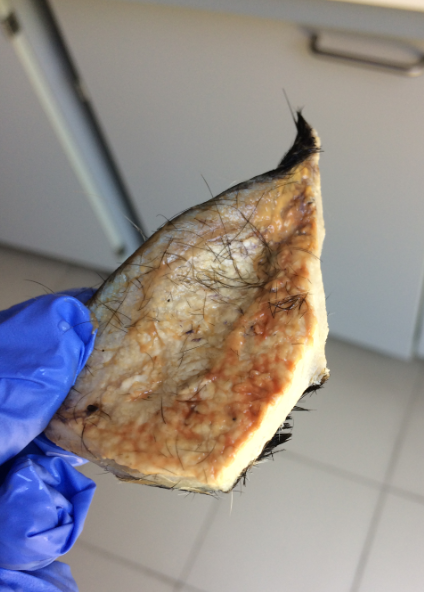 | 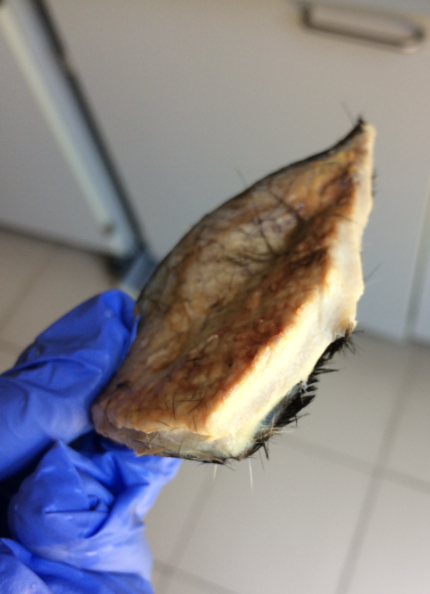 |
| --- | --- | --- |

**Supplementary Figure 3.** Bovine rawhide shows reddish and superficial discolouration on the flesh side, after salt curing with industrial salt contaminated by Haloarchaea, and incubation for three months. Photos by N. Perini.
