# Supplementary material for: The Stain of the Original Salt: Red Heats on Chrome Tanned Leathers and Purple Spots on Ancient Parchments Are Two Sides of the Same Ecological Coin
Source: Front Microbiol. 2019 Oct 29;10:2459. doi: 10.3389/fmicb.2019.02459 (PMC6828845; doi:10.3389/fmicb.2019.02459)
Supplement: Supplementary file 2 [file Table_1.docx]

Supplementary Material

**Supplementary Table 1.** Number of sequences found in each replicate sample from bovine rawhide (BRH), red damaged tanned leather (DTL) and undamaged tanned leather (UTL) samples.

| **#OTU** | **RHB1** | **RHB2** | **RHB3** | **DTL1** | **DTL2** | **DTL3** | **UTL1** | **UTL2** | **UTL3** |
| --- | --- | --- | --- | --- | --- | --- | --- | --- | --- |
| **OTU0001** | 148 | 104 | 176 | 6 | 32 | 22 | 2 | 3 | 4 |
| **OTU0002** | 1 | 1 | 2 | 2 | 12 | 10 | 5 | 10 | 7 |
| **OTU0003** | 16 | 35 | 29 | 0 | 6 | 11 | 0 | 0 | 1 |
| **OTU0004** | 2 | 1 | 8 | 33 | 16 | 22 | 13 | 10 | 8 |
| **OTU0005** | 18 | 18 | 38 | 2 | 4 | 2 | 1 | 0 | 0 |
| **OTU0006** | 1 | 3 | 2 | 35 | 19 | 12 | 31 | 15 | 13 |
| **OTU0007** | 0 | 0 | 0 | 17 | 11 | 5 | 15 | 8 | 4 |
| **OTU0008** | 30 | 27 | 46 | 0 | 14 | 5 | 0 | 0 | 1 |
| **OTU0009** | 0 | 2 | 3 | 54 | 30 | 23 | 33 | 21 | 12 |
| **OTU0010** | 1 | 3 | 0 | 20 | 4 | 13 | 19 | 12 | 7 |
| **OTU0011** | 33 | 34 | 69 | 0 | 10 | 9 | 0 | 0 | 0 |
| **OTU0012** | 2 | 2 | 2 | 89 | 26 | 22 | 43 | 33 | 36 |
| **OTU0013** | 5 | 5 | 9 | 0 | 3 | 2 | 0 | 0 | 0 |
| **OTU0014** | 0 | 0 | 0 | 12 | 5 | 5 | 4 | 3 | 6 |
| **OTU0015** | 86 | 68 | 166 | 0 | 83 | 93 | 0 | 3 | 1 |
| **OTU0016** | 8 | 7 | 15 | 2 | 2 | 5 | 0 | 1 | 0 |
| **OTU0017** | 1 | 0 | 0 | 8 | 3 | 7 | 7 | 0 | 1 |
| **OTU0018** | 12 | 7 | 11 | 0 | 1 | 3 | 1 | 0 | 1 |
| **OTU0019** | 0 | 1 | 0 | 12 | 8 | 7 | 11 | 5 | 11 |
| **OTU0020** | 12 | 4 | 19 | 0 | 0 | 0 | 0 | 0 | 1 |
| **OTU0021** | 6 | 5 | 8 | 0 | 2 | 2 | 0 | 0 | 0 |
| **OTU0022** | 0 | 1 | 3 | 27 | 8 | 10 | 13 | 6 | 8 |
| **OTU0023** | 1 | 1 | 0 | 6 | 4 | 7 | 3 | 5 | 3 |
| **OTU0024** | 28 | 15 | 28 | 0 | 3 | 2 | 0 | 0 | 0 |
| **OTU0025** | 2 | 0 | 1 | 0 | 0 | 0 | 1 | 2 | 1 |
| **OTU0026** | 0 | 0 | 1 | 7 | 2 | 1 | 6 | 1 | 2 |
| **OTU0027** | 2 | 2 | 3 | 3 | 39 | 48 | 14 | 29 | 36 |
| **OTU0028** | 5 | 3 | 11 | 0 | 3 | 1 | 0 | 0 | 0 |
| **OTU0029** | 0 | 1 | 0 | 7 | 7 | 8 | 7 | 7 | 7 |
| **OTU0030** | 0 | 1 | 0 | 12 | 5 | 1 | 6 | 8 | 5 |
| **OTU0031** | 5 | 6 | 7 | 0 | 0 | 1 | 0 | 0 | 0 |
| **OTU0032** | 3 | 2 | 2 | 47 | 28 | 21 | 45 | 25 | 40 |
| **OTU0033** | 279 | 342 | 832 | 6 | 63 | 65 | 2 | 6 | 3 |
| **OTU0034** | 2 | 9 | 7 | 0 | 0 | 0 | 0 | 0 | 0 |
| **OTU0035** | 7 | 10 | 16 | 0 | 4 | 2 | 0 | 0 | 0 |
| **OTU0036** | 13 | 4 | 11 | 0 | 2 | 3 | 0 | 1 | 0 |
| **OTU0037** | 37 | 41 | 55 | 0 | 13 | 4 | 0 | 0 | 0 |
| **OTU0038** | 254 | 187 | 287 | 1 | 83 | 66 | 0 | 0 | 0 |
| **OTU0039** | 1 | 0 | 5 | 116 | 28 | 21 | 41 | 29 | 42 |
| **OTU0040** | 115 | 72 | 173 | 5063 | 2365 | 2308 | 2973 | 2325 | 2559 |
| **OTU0041** | 0 | 0 | 0 | 3 | 2 | 2 | 2 | 0 | 1 |
| **OTU0042** | 7 | 3 | 4 | 0 | 4 | 1 | 0 | 0 | 1 |
| **OTU0043** | 3 | 0 | 1 | 13 | 4 | 27 | 15 | 8 | 9 |
| **OTU0044** | 200 | 214 | 424 | 1 | 27 | 27 | 4 | 1 | 2 |
| **OTU0045** | 0 | 0 | 0 | 7 | 5 | 3 | 1 | 0 | 3 |
| **OTU0046** | 13 | 18 | 35 | 0 | 2 | 2 | 0 | 0 | 0 |
| **OTU0047** | 35 | 49 | 95 | 1 | 75 | 57 | 0 | 0 | 0 |
| **OTU0048** | 0 | 2 | 0 | 5 | 4 | 1 | 2 | 1 | 1 |
| **OTU0049** | 225 | 173 | 370 | 1 | 12 | 5 | 2 | 0 | 2 |
| **OTU0050** | 151 | 203 | 602 | 3 | 25 | 54 | 1 | 3 | 3 |
| **OTU0051** | 14 | 22 | 45 | 0 | 9 | 4 | 0 | 0 | 0 |
| **OTU0052** | 7 | 7 | 5 | 2 | 1 | 3 | 4 | 1 | 0 |
| **OTU0053** | 183 | 127 | 274 | 3 | 11 | 3 | 0 | 4 | 1 |
| **OTU0054** | 149 | 111 | 189 | 0 | 2 | 4 | 0 | 3 | 1 |
| **OTU0055** | 0 | 0 | 0 | 8 | 1 | 2 | 4 | 4 | 1 |
| **OTU0056** | 31 | 24 | 91 | 1 | 4 | 1 | 1 | 1 | 1 |
| **OTU0057** | 14 | 3 | 14 | 582 | 346 | 297 | 342 | 198 | 247 |
| **OTU0058** | 0 | 0 | 1 | 13 | 6 | 11 | 25 | 5 | 4 |
| **OTU0059** | 116 | 162 | 222 | 6 | 28 | 32 | 6 | 3 | 4 |
| **OTU0060** | 10 | 19 | 14 | 120 | 137 | 62 | 237 | 47 | 58 |
| **OTU0061** | 9 | 15 | 16 | 0 | 2 | 1 | 0 | 0 | 0 |
| **OTU0062** | 0 | 0 | 0 | 1 | 1 | 1 | 1 | 0 | 2 |
| **OTU0063** | 0 | 0 | 0 | 34 | 11 | 11 | 30 | 12 | 0 |
| **OTU0064** | 11 | 2 | 4 | 73 | 42 | 113 | 98 | 52 | 32 |
| **OTU0065** | 0 | 0 | 0 | 19 | 2 | 6 | 4 | 6 | 3 |
| **OTU0066** | 2 | 5 | 5 | 157 | 41 | 10 | 91 | 91 | 130 |
| **OTU0067** | 0 | 0 | 0 | 5 | 3 | 3 | 3 | 1 | 0 |
| **OTU0068** | 0 | 0 | 2 | 106 | 9 | 4 | 6 | 0 | 0 |
| **OTU0069** | 4 | 0 | 3 | 118 | 13 | 2 | 86 | 103 | 24 |
| **OTU0070** | 86 | 74 | 176 | 8 | 12 | 10 | 3 | 2 | 0 |
| **OTU0071** | 5 | 5 | 15 | 0 | 3 | 2 | 0 | 0 | 0 |
| **OTU0072** | 52 | 23 | 67 | 0 | 24 | 15 | 0 | 0 | 0 |
| **OTU0073** | 137 | 77 | 222 | 3 | 19 | 3 | 0 | 12 | 1 |
| **OTU0074** | 0 | 0 | 0 | 27 | 8 | 16 | 17 | 9 | 1 |
| **OTU0075** | 21 | 15 | 31 | 0 | 15 | 5 | 0 | 0 | 0 |
| **OTU0076** | 0 | 0 | 0 | 1 | 1 | 2 | 5 | 1 | 0 |
| **OTU0077** | 55 | 71 | 119 | 2 | 64 | 17 | 0 | 0 | 0 |
| **OTU0078** | 6 | 5 | 1 | 92 | 50 | 21 | 1 | 74 | 16 |
| **OTU0079** | 91 | 56 | 124 | 1 | 13 | 16 | 3 | 0 | 0 |
| **OTU0080** | 22 | 22 | 36 | 1 | 2 | 0 | 0 | 0 | 0 |
| **OTU0081** | 40 | 30 | 69 | 1776 | 994 | 1040 | 1015 | 1011 | 892 |
| **OTU0082** | 2 | 4 | 3 | 435 | 2 | 2 | 0 | 1 | 0 |
| **OTU0083** | 4 | 2 | 6 | 1 | 0 | 13 | 5 | 0 | 2 |
| **OTU0084** | 15 | 8 | 26 | 0 | 7 | 15 | 0 | 0 | 0 |
| **OTU0085** | 41 | 26 | 66 | 20 | 4 | 4 | 2 | 16 | 0 |
| **OTU0086** | 103 | 81 | 144 | 3 | 11 | 17 | 1 | 2 | 0 |
| **OTU0087** | 0 | 0 | 0 | 1 | 3 | 2 | 0 | 4 | 2 |
| **OTU0088** | 169 | 62 | 123 | 1 | 0 | 16 | 0 | 1 | 0 |
| **OTU0089** | 14 | 18 | 29 | 599 | 336 | 310 | 579 | 267 | 350 |
| **OTU0090** | 1 | 1 | 9 | 84 | 23 | 10 | 122 | 27 | 44 |
| **OTU0091** | 11 | 11 | 15 | 0 | 2 | 1 | 0 | 0 | 0 |
| **OTU0092** | 0 | 0 | 1 | 179 | 1 | 11 | 0 | 13 | 10 |
| **OTU0093** | 55 | 51 | 96 | 0 | 31 | 19 | 0 | 0 | 0 |
| **OTU0094** | 142 | 50 | 133 | 0 | 23 | 2 | 0 | 0 | 1 |
| **OTU0095** | 13 | 9 | 22 | 0 | 5 | 3 | 0 | 1 | 0 |
| **OTU0096** | 272 | 294 | 433 | 1 | 72 | 52 | 0 | 1 | 0 |
| **OTU0097** | 34 | 23 | 101 | 0 | 5 | 12 | 0 | 0 | 1 |
| **OTU0098** | 0 | 1 | 0 | 7 | 3 | 1 | 2 | 3 | 1 |
| **OTU0099** | 6 | 4 | 7 | 92 | 45 | 59 | 167 | 69 | 83 |
| **OTU0100** | 0 | 2 | 7 | 92 | 36 | 17 | 41 | 48 | 35 |
| **OTU0101** | 64 | 55 | 53 | 0 | 6 | 0 | 1 | 0 | 1 |
| **OTU0102** | 59 | 43 | 119 | 2 | 2 | 9 | 0 | 0 | 2 |
| **OTU0103** | 0 | 0 | 2 | 4 | 0 | 5 | 3 | 1 | 0 |
| **OTU0104** | 33 | 22 | 83 | 0 | 9 | 13 | 0 | 0 | 0 |
| **OTU0105** | 2 | 1 | 2 | 327 | 2 | 2 | 1 | 3 | 3 |
| **OTU0106** | 126 | 91 | 101 | 3 | 7 | 6 | 2 | 1 | 0 |
| **OTU0107** | 4 | 2 | 3 | 3 | 31 | 43 | 156 | 2 | 30 |
| **OTU0108** | 0 | 0 | 0 | 4 | 1 | 1 | 0 | 0 | 3 |
| **OTU0109** | 0 | 0 | 2 | 8 | 1 | 3 | 11 | 3 | 1 |
| **OTU0110** | 31 | 35 | 88 | 1 | 0 | 7 | 0 | 1 | 0 |
| **OTU0111** | 1 | 0 | 2 | 0 | 0 | 0 | 0 | 0 | 0 |
| **OTU0112** | 4 | 3 | 3 | 8 | 24 | 39 | 63 | 65 | 39 |
| **OTU0113** | 1 | 1 | 3 | 52 | 26 | 23 | 42 | 55 | 31 |
| **OTU0114** | 0 | 0 | 3 | 217 | 2 | 2 | 1 | 1 | 2 |
| **OTU0115** | 44 | 61 | 104 | 1 | 11 | 0 | 0 | 3 | 1 |
| **OTU0116** | 0 | 0 | 3 | 79 | 22 | 26 | 5 | 52 | 11 |
| **OTU0117** | 2 | 0 | 3 | 94 | 36 | 27 | 28 | 23 | 26 |
| **OTU0118** | 0 | 0 | 0 | 7 | 3 | 3 | 4 | 5 | 2 |
| **OTU0119** | 16 | 10 | 19 | 1 | 2 | 6 | 0 | 0 | 0 |
| **OTU0120** | 50 | 47 | 63 | 1 | 1 | 4 | 0 | 0 | 0 |
| **OTU0121** | 0 | 0 | 1 | 5 | 5 | 1 | 0 | 0 | 1 |
| **OTU0122** | 2 | 2 | 4 | 0 | 0 | 0 | 0 | 0 | 0 |
| **OTU0123** | 0 | 1 | 0 | 25 | 0 | 1 | 0 | 0 | 0 |
| **OTU0124** | 16 | 23 | 46 | 0 | 21 | 9 | 1 | 1 | 0 |
| **OTU0125** | 4 | 3 | 0 | 124 | 51 | 42 | 65 | 29 | 25 |
| **OTU0126** | 1 | 1 | 0 | 24 | 28 | 21 | 0 | 1 | 20 |
| **OTU0127** | 20 | 38 | 67 | 1 | 1 | 0 | 0 | 1 | 1 |
| **OTU0128** | 3 | 10 | 10 | 0 | 1 | 3 | 0 | 0 | 0 |
| **OTU0129** | 51 | 78 | 110 | 0 | 23 | 30 | 0 | 0 | 0 |
| **OTU0130** | 0 | 1 | 0 | 48 | 17 | 14 | 23 | 18 | 14 |
| **OTU0131** | 10 | 10 | 18 | 0 | 1 | 1 | 0 | 0 | 0 |
| **OTU0132** | 23 | 28 | 41 | 0 | 7 | 9 | 0 | 0 | 0 |
| **OTU0133** | 21 | 12 | 23 | 0 | 1 | 1 | 0 | 0 | 0 |
| **OTU0134** | 71 | 31 | 65 | 3 | 8 | 0 | 0 | 0 | 1 |
| **OTU0135** | 3 | 3 | 0 | 0 | 2 | 1 | 6 | 0 | 0 |
| **OTU0136** | 0 | 3 | 0 | 16 | 8 | 12 | 82 | 0 | 2 |
| **OTU0137** | 0 | 1 | 5 | 4 | 39 | 30 | 43 | 40 | 12 |
| **OTU0138** | 78 | 71 | 104 | 1 | 0 | 3 | 1 | 1 | 0 |
| **OTU0139** | 20 | 12 | 83 | 1 | 1 | 5 | 0 | 0 | 0 |
| **OTU0140** | 78 | 60 | 80 | 2 | 3 | 3 | 0 | 1 | 0 |
| **OTU0141** | 2 | 3 | 6 | 9 | 6 | 2 | 0 | 105 | 44 |
| **OTU0142** | 88 | 25 | 70 | 0 | 0 | 0 | 0 | 1 | 3 |
| **OTU0143** | 3 | 7 | 4 | 13 | 11 | 3 | 6 | 2 | 2 |
| **OTU0144** | 0 | 0 | 0 | 0 | 4 | 4 | 3 | 2 | 0 |
| **OTU0145** | 10 | 24 | 53 | 0 | 8 | 0 | 0 | 0 | 1 |
| **OTU0146** | 63 | 42 | 66 | 1 | 2 | 34 | 1 | 0 | 1 |
| **OTU0147** | 1 | 0 | 1 | 12 | 2 | 0 | 104 | 0 | 2 |
| **OTU0148** | 26 | 26 | 36 | 0 | 7 | 7 | 0 | 1 | 0 |
| **OTU0149** | 1 | 0 | 2 | 7 | 6 | 0 | 6 | 0 | 0 |
| **OTU0150** | 0 | 2 | 2 | 80 | 2 | 0 | 76 | 2 | 0 |
| **OTU0151** | 0 | 1 | 2 | 30 | 18 | 34 | 0 | 1 | 0 |
| **OTU0152** | 0 | 2 | 1 | 151 | 45 | 41 | 55 | 27 | 41 |
| **OTU0153** | 1 | 4 | 6 | 0 | 1 | 3 | 0 | 0 | 0 |
| **OTU0154** | 0 | 0 | 2 | 76 | 3 | 15 | 12 | 12 | 7 |
| **OTU0155** | 2 | 1 | 2 | 41 | 20 | 18 | 16 | 18 | 12 |
| **OTU0156** | 17 | 29 | 86 | 1 | 1 | 2 | 0 | 0 | 0 |
| **OTU0157** | 6 | 5 | 4 | 270 | 58 | 65 | 99 | 78 | 64 |
| **OTU0158** | 119 | 140 | 218 | 1 | 25 | 24 | 0 | 0 | 0 |
| **OTU0159** | 156 | 145 | 239 | 2 | 11 | 4 | 1 | 2 | 0 |
| **OTU0160** | 0 | 0 | 0 | 9 | 21 | 26 | 13 | 26 | 17 |
| **OTU0161** | 9 | 17 | 31 | 0 | 1 | 1 | 0 | 0 | 2 |
| **OTU0162** | 2 | 5 | 25 | 0 | 26 | 0 | 0 | 0 | 0 |
| **OTU0163** | 1 | 0 | 3 | 56 | 20 | 25 | 52 | 24 | 2 |
| **OTU0164** | 1 | 1 | 2 | 84 | 0 | 1 | 0 | 0 | 68 |
| **OTU0165** | 0 | 0 | 1 | 26 | 12 | 29 | 0 | 0 | 32 |
| **OTU0166** | 13 | 14 | 21 | 21 | 9 | 4 | 53 | 19 | 2 |
| **OTU0167** | 0 | 0 | 0 | 1 | 1 | 0 | 3 | 0 | 2 |
| **OTU0168** | 0 | 2 | 3 | 46 | 0 | 8 | 27 | 28 | 2 |
| **OTU0169** | 1 | 2 | 0 | 0 | 0 | 0 | 0 | 1 | 0 |
| **OTU0170** | 13 | 7 | 19 | 0 | 0 | 6 | 0 | 0 | 0 |
| **OTU0171** | 0 | 0 | 5 | 24 | 19 | 11 | 19 | 13 | 9 |
| **OTU0172** | 26 | 39 | 50 | 0 | 17 | 15 | 0 | 0 | 0 |
| **OTU0173** | 0 | 0 | 0 | 1 | 6 | 0 | 72 | 0 | 0 |
| **OTU0174** | 78 | 24 | 43 | 1 | 0 | 1 | 0 | 2 | 1 |
| **OTU0175** | 0 | 0 | 0 | 1 | 0 | 0 | 0 | 1 | 0 |
| **OTU0176** | 40 | 62 | 77 | 0 | 23 | 11 | 0 | 0 | 0 |
| **OTU0177** | 2 | 1 | 0 | 0 | 1 | 0 | 0 | 0 | 0 |
| **OTU0178** | 0 | 0 | 1 | 2 | 10 | 9 | 3 | 7 | 8 |
| **OTU0179** | 5 | 6 | 3 | 0 | 1 | 2 | 1 | 2 | 2 |
| **OTU0180** | 6 | 6 | 4 | 0 | 3 | 6 | 0 | 0 | 0 |
| **OTU0181** | 98 | 63 | 162 | 2 | 71 | 59 | 0 | 0 | 1 |
| **OTU0182** | 5 | 3 | 10 | 0 | 0 | 2 | 0 | 0 | 0 |
| **OTU0183** | 5 | 2 | 0 | 0 | 0 | 0 | 0 | 0 | 0 |
| **OTU0184** | 16 | 17 | 29 | 0 | 6 | 9 | 0 | 0 | 0 |
| **OTU0185** | 0 | 1 | 0 | 40 | 0 | 0 | 1 | 29 | 5 |
| **OTU0186** | 3 | 5 | 2 | 0 | 1 | 4 | 0 | 0 | 0 |
| **OTU0187** | 2 | 2 | 6 | 85 | 54 | 46 | 56 | 61 | 57 |
| **OTU0188** | 4 | 2 | 9 | 2 | 0 | 1 | 0 | 1 | 0 |
| **OTU0189** | 2 | 1 | 6 | 0 | 0 | 0 | 0 | 0 | 0 |
| **OTU0190** | 7 | 10 | 19 | 1 | 18 | 41 | 0 | 1 | 0 |
| **OTU0191** | 1 | 0 | 3 | 0 | 1 | 0 | 0 | 0 | 0 |
| **OTU0192** | 0 | 0 | 0 | 0 | 2 | 0 | 0 | 0 | 0 |
| **OTU0193** | 9 | 4 | 8 | 0 | 5 | 2 | 0 | 0 | 0 |
| **OTU0194** | 1 | 0 | 0 | 39 | 28 | 30 | 26 | 17 | 23 |
| **OTU0195** | 21 | 9 | 20 | 0 | 11 | 8 | 0 | 0 | 1 |
| **OTU0196** | 0 | 0 | 0 | 1 | 1 | 3 | 6 | 0 | 0 |
| **OTU0197** | 6 | 3 | 6 | 0 | 1 | 0 | 0 | 0 | 0 |
| **OTU0198** | 2 | 3 | 4 | 0 | 0 | 3 | 0 | 0 | 0 |
| **OTU0199** | 0 | 0 | 0 | 37 | 18 | 21 | 4 | 31 | 22 |
| **OTU0200** | 22 | 47 | 47 | 0 | 14 | 6 | 0 | 0 | 0 |
| **OTU0201** | 1 | 0 | 1 | 42 | 13 | 15 | 0 | 11 | 10 |
| **OTU0202** | 1 | 5 | 3 | 0 | 4 | 2 | 1 | 0 | 0 |
| **OTU0203** | 21 | 17 | 21 | 3 | 0 | 0 | 0 | 0 | 0 |
| **OTU0204** | 48 | 58 | 113 | 0 | 8 | 10 | 0 | 0 | 0 |
| **OTU0205** | 1 | 0 | 1 | 81 | 2 | 0 | 14 | 1 | 2 |
| **OTU0206** | 8 | 10 | 23 | 0 | 4 | 5 | 0 | 0 | 0 |
| **OTU0207** | 5 | 20 | 50 | 1 | 0 | 0 | 0 | 0 | 0 |
| **OTU0208** | 4 | 3 | 3 | 0 | 2 | 1 | 0 | 0 | 0 |
| **OTU0209** | 3 | 0 | 0 | 32 | 6 | 9 | 25 | 0 | 0 |
| **OTU0210** | 0 | 1 | 2 | 0 | 0 | 1 | 0 | 0 | 0 |
| **OTU0211** | 1 | 2 | 1 | 2 | 0 | 1 | 59 | 1 | 1 |
| **OTU0212** | 0 | 0 | 0 | 8 | 4 | 1 | 4 | 1 | 2 |
| **OTU0213** | 5 | 6 | 7 | 0 | 1 | 3 | 0 | 0 | 0 |
| **OTU0214** | 0 | 0 | 0 | 4 | 2 | 6 | 4 | 2 | 3 |
| **OTU0215** | 104 | 58 | 106 | 0 | 12 | 15 | 1 | 0 | 0 |
| **OTU0216** | 8 | 4 | 6 | 43 | 5 | 72 | 16 | 12 | 5 |
| **OTU0217** | 1 | 3 | 2 | 0 | 2 | 2 | 0 | 0 | 0 |
| **OTU0218** | 15 | 20 | 27 | 0 | 6 | 4 | 0 | 0 | 0 |
| **OTU0219** | 5 | 5 | 22 | 0 | 5 | 36 | 0 | 0 | 0 |
| **OTU0220** | 0 | 2 | 1 | 63 | 0 | 0 | 0 | 0 | 0 |
| **OTU0221** | 14 | 23 | 47 | 0 | 15 | 22 | 0 | 0 | 0 |
| **OTU0222** | 0 | 0 | 0 | 1 | 0 | 2 | 0 | 2 | 0 |
| **OTU0223** | 17 | 21 | 39 | 1 | 6 | 4 | 0 | 0 | 1 |
| **OTU0224** | 0 | 0 | 1 | 1 | 0 | 50 | 0 | 0 | 4 |
| **OTU0225** | 1 | 0 | 3 | 0 | 0 | 5 | 37 | 0 | 1 |
| **OTU0226** | 1 | 1 | 1 | 0 | 0 | 0 | 0 | 0 | 0 |
| **OTU0227** | 14 | 20 | 57 | 0 | 0 | 1 | 0 | 0 | 0 |
| **OTU0228** | 6 | 3 | 7 | 0 | 2 | 3 | 0 | 0 | 0 |
| **OTU0229** | 1 | 0 | 0 | 0 | 0 | 1 | 0 | 0 | 0 |
| **OTU0230** | 1 | 3 | 4 | 108 | 18 | 24 | 79 | 36 | 27 |
| **OTU0231** | 21 | 8 | 36 | 0 | 15 | 9 | 0 | 0 | 0 |
| **OTU0232** | 1 | 0 | 1 | 11 | 1 | 0 | 0 | 26 | 10 |
| **OTU0233** | 1 | 0 | 0 | 32 | 0 | 38 | 0 | 1 | 0 |
| **OTU0234** | 1 | 0 | 1 | 13 | 24 | 31 | 0 | 0 | 25 |
| **OTU0235** | 3 | 1 | 3 | 0 | 0 | 0 | 0 | 0 | 0 |
| **OTU0236** | 62 | 58 | 96 | 1 | 12 | 1 | 0 | 2 | 0 |
| **OTU0237** | 33 | 16 | 37 | 1 | 3 | 0 | 0 | 0 | 0 |
| **OTU0238** | 36 | 14 | 35 | 1 | 16 | 1 | 0 | 0 | 1 |
| **OTU0239** | 1 | 0 | 0 | 45 | 7 | 0 | 0 | 0 | 1 |
| **OTU0240** | 41 | 39 | 70 | 0 | 6 | 9 | 0 | 0 | 0 |
| **OTU0241** | 8 | 1 | 5 | 0 | 2 | 1 | 0 | 0 | 0 |
| **OTU0242** | 0 | 0 | 1 | 81 | 0 | 0 | 0 | 0 | 0 |
| **OTU0243** | 11 | 18 | 39 | 0 | 19 | 0 | 1 | 0 | 0 |
| **OTU0244** | 17 | 17 | 46 | 0 | 5 | 21 | 2 | 0 | 0 |
| **OTU0245** | 4 | 7 | 21 | 0 | 1 | 0 | 0 | 0 | 0 |
| **OTU0246** | 27 | 10 | 17 | 30 | 0 | 0 | 0 | 0 | 0 |
| **OTU0247** | 2 | 0 | 0 | 16 | 0 | 0 | 0 | 32 | 0 |
| **OTU0248** | 3 | 5 | 6 | 0 | 5 | 3 | 0 | 0 | 0 |
| **OTU0249** | 1 | 1 | 5 | 0 | 1 | 0 | 0 | 0 | 0 |
| **OTU0250** | 0 | 1 | 0 | 15 | 6 | 4 | 10 | 4 | 1 |
| **OTU0251** | 24 | 14 | 31 | 0 | 6 | 14 | 0 | 0 | 0 |
| **OTU0252** | 19 | 19 | 40 | 0 | 0 | 3 | 0 | 0 | 0 |
| **OTU0253** | 62 | 57 | 118 | 0 | 9 | 12 | 0 | 0 | 0 |
| **OTU0254** | 2 | 3 | 3 | 225 | 96 | 109 | 158 | 91 | 99 |
| **OTU0255** | 1 | 0 | 2 | 0 | 1 | 1 | 51 | 0 | 3 |
| **OTU0256** | 0 | 0 | 2 | 48 | 0 | 0 | 0 | 10 | 13 |
| **OTU0257** | 38 | 39 | 64 | 1 | 0 | 0 | 0 | 0 | 0 |
| **OTU0258** | 0 | 0 | 1 | 2 | 0 | 1 | 1 | 0 | 0 |
| **OTU0259** | 0 | 0 | 1 | 113 | 35 | 31 | 44 | 32 | 28 |
| **OTU0260** | 3 | 0 | 0 | 0 | 0 | 2 | 0 | 0 | 0 |
| **OTU0261** | 0 | 0 | 0 | 1 | 1 | 1 | 0 | 0 | 0 |
| **OTU0262** | 15 | 21 | 29 | 0 | 7 | 11 | 0 | 0 | 0 |
| **OTU0263** | 6 | 3 | 8 | 0 | 5 | 2 | 0 | 0 | 0 |
| **OTU0264** | 33 | 23 | 29 | 0 | 14 | 14 | 0 | 0 | 0 |
| **OTU0265** | 18 | 16 | 35 | 0 | 16 | 11 | 0 | 0 | 0 |
| **OTU0266** | 0 | 0 | 0 | 1 | 0 | 6 | 38 | 0 | 6 |
| **OTU0267** | 11 | 14 | 11 | 0 | 13 | 9 | 0 | 1 | 0 |
| **OTU0268** | 0 | 0 | 1 | 49 | 6 | 0 | 0 | 0 | 0 |
| **OTU0269** | 22 | 21 | 45 | 0 | 11 | 15 | 0 | 0 | 0 |
| **OTU0270** | 5 | 7 | 6 | 0 | 1 | 2 | 0 | 0 | 0 |
| **OTU0271** | 17 | 18 | 37 | 0 | 1 | 4 | 0 | 0 | 0 |
| **OTU0272** | 0 | 0 | 2 | 339 | 18 | 11 | 9 | 14 | 18 |
| **OTU0273** | 1 | 3 | 1 | 87 | 36 | 17 | 36 | 14 | 15 |
| **OTU0274** | 1 | 1 | 0 | 0 | 3 | 10 | 0 | 16 | 5 |
| **OTU0275** | 1 | 0 | 2 | 15 | 5 | 7 | 0 | 23 | 0 |
| **OTU0276** | 2 | 3 | 8 | 109 | 31 | 36 | 67 | 56 | 49 |
| **OTU0277** | 3 | 1 | 0 | 17 | 41 | 2 | 1 | 0 | 2 |
| **OTU0278** | 2 | 2 | 3 | 0 | 1 | 2 | 49 | 1 | 0 |
| **OTU0279** | 8 | 4 | 15 | 0 | 1 | 2 | 0 | 0 | 0 |
| **OTU0280** | 49 | 21 | 64 | 128 | 7 | 2 | 0 | 2 | 1 |
| **OTU0281** | 3 | 1 | 4 | 0 | 2 | 3 | 0 | 0 | 0 |
| **OTU0282** | 2 | 2 | 3 | 17 | 17 | 1 | 1 | 10 | 7 |
| **OTU0283** | 38 | 43 | 81 | 0 | 12 | 9 | 0 | 0 | 0 |
| **OTU0284** | 11 | 8 | 5 | 0 | 3 | 0 | 0 | 0 | 0 |
| **OTU0285** | 0 | 0 | 1 | 22 | 18 | 16 | 1 | 0 | 0 |
| **OTU0286** | 8 | 6 | 12 | 0 | 4 | 4 | 0 | 0 | 0 |
| **OTU0287** | 1 | 1 | 2 | 57 | 17 | 24 | 24 | 32 | 17 |
| **OTU0288** | 8 | 0 | 1 | 1 | 0 | 12 | 0 | 41 | 0 |
| **OTU0289** | 18 | 9 | 21 | 0 | 9 | 7 | 0 | 0 | 0 |
| **OTU0290** | 16 | 22 | 30 | 0 | 11 | 22 | 0 | 0 | 0 |
| **OTU0291** | 11 | 8 | 12 | 0 | 2 | 7 | 0 | 0 | 0 |
| **OTU0292** | 1 | 1 | 2 | 0 | 8 | 37 | 2 | 0 | 10 |
| **OTU0293** | 13 | 11 | 23 | 0 | 14 | 9 | 0 | 0 | 0 |
| **OTU0294** | 19 | 4 | 7 | 0 | 13 | 4 | 0 | 0 | 0 |
| **OTU0295** | 9 | 10 | 29 | 1 | 2 | 1 | 0 | 0 | 1 |
| **OTU0296** | 0 | 0 | 1 | 3 | 1 | 1 | 2 | 1 | 3 |
| **OTU0297** | 0 | 1 | 0 | 0 | 0 | 1 | 56 | 1 | 1 |
| **OTU0298** | 0 | 0 | 0 | 25 | 8 | 15 | 6 | 7 | 5 |
| **OTU0299** | 2 | 1 | 0 | 27 | 13 | 14 | 17 | 12 | 4 |
| **OTU0300** | 36 | 11 | 16 | 0 | 0 | 1 | 0 | 0 | 0 |
| **OTU0301** | 46 | 38 | 79 | 0 | 7 | 4 | 0 | 0 | 0 |
| **OTU0302** | 0 | 0 | 0 | 3 | 4 | 5 | 52 | 13 | 1 |
| **OTU0303** | 0 | 0 | 0 | 3 | 0 | 0 | 0 | 5 | 1 |
| **OTU0304** | 1 | 0 | 0 | 19 | 5 | 5 | 14 | 12 | 10 |
| **OTU0305** | 0 | 0 | 0 | 4 | 1 | 0 | 0 | 3 | 0 |
| **OTU0306** | 2 | 0 | 4 | 7 | 9 | 14 | 14 | 13 | 8 |
| **OTU0307** | 16 | 26 | 18 | 0 | 0 | 0 | 1 | 2 | 0 |
| **OTU0308** | 1 | 0 | 2 | 7 | 0 | 0 | 0 | 11 | 7 |
| **OTU0309** | 10 | 2 | 16 | 1 | 4 | 3 | 0 | 1 | 0 |
| **OTU0310** | 8 | 17 | 9 | 87 | 95 | 57 | 129 | 10 | 31 |
| **OTU0311** | 78 | 61 | 224 | 14 | 14 | 8 | 2 | 7 | 1 |
| **OTU0312** | 3 | 2 | 6 | 0 | 3 | 0 | 0 | 0 | 0 |
| **OTU0313** | 66 | 53 | 95 | 1 | 2 | 5 | 0 | 1 | 0 |
| **OTU0314** | 2 | 3 | 3 | 0 | 0 | 0 | 0 | 0 | 0 |
| **OTU0315** | 0 | 0 | 2 | 0 | 2 | 2 | 0 | 0 | 0 |
| **OTU0316** | 2 | 0 | 2 | 0 | 0 | 10 | 43 | 0 | 0 |
| **OTU0317** | 7 | 7 | 12 | 0 | 2 | 8 | 0 | 0 | 0 |
| **OTU0318** | 44 | 54 | 101 | 2853 | 1841 | 1385 | 1818 | 1212 | 925 |
| **OTU0319** | 3 | 0 | 0 | 15 | 0 | 0 | 0 | 12 | 3 |
| **OTU0320** | 9 | 8 | 9 | 0 | 1 | 3 | 0 | 0 | 0 |
| **OTU0321** | 20 | 30 | 41 | 0 | 7 | 4 | 0 | 0 | 0 |
| **OTU0322** | 3 | 2 | 12 | 0 | 1 | 5 | 0 | 0 | 0 |
| **OTU0323** | 0 | 0 | 1 | 0 | 1 | 1 | 51 | 0 | 0 |
| **OTU0324** | 3 | 1 | 3 | 0 | 0 | 0 | 0 | 0 | 0 |
| **OTU0325** | 7 | 12 | 23 | 0 | 0 | 0 | 0 | 1 | 0 |
| **OTU0326** | 8 | 12 | 8 | 0 | 1 | 0 | 0 | 0 | 0 |
| **OTU0327** | 13 | 6 | 20 | 0 | 4 | 8 | 0 | 0 | 0 |
| **OTU0328** | 1 | 0 | 1 | 1 | 0 | 0 | 0 | 25 | 3 |
| **OTU0329** | 1 | 2 | 2 | 47 | 38 | 37 | 31 | 32 | 43 |
| **OTU0330** | 6 | 1 | 1 | 38 | 1 | 20 | 0 | 15 | 1 |
| **OTU0331** | 33 | 20 | 69 | 0 | 10 | 6 | 0 | 0 | 0 |
| **OTU0332** | 8 | 3 | 28 | 0 | 0 | 15 | 1 | 0 | 0 |
| **OTU0333** | 0 | 0 | 1 | 1 | 2 | 0 | 44 | 0 | 0 |
| **OTU0334** | 1 | 0 | 0 | 5 | 15 | 14 | 13 | 4 | 3 |
| **OTU0335** | 0 | 0 | 2 | 1 | 14 | 6 | 0 | 0 | 34 |
| **OTU0336** | 13 | 15 | 16 | 0 | 3 | 5 | 0 | 0 | 0 |
| **OTU0337** | 18 | 0 | 2 | 8 | 0 | 5 | 0 | 0 | 25 |
| **OTU0338** | 1 | 1 | 0 | 20 | 1 | 3 | 2 | 28 | 0 |
| **OTU0339** | 0 | 1 | 3 | 0 | 0 | 0 | 0 | 0 | 0 |
| **OTU0340** | 0 | 0 | 0 | 1 | 3 | 2 | 3 | 3 | 3 |
| **OTU0341** | 36 | 42 | 70 | 0 | 15 | 16 | 0 | 0 | 0 |
| **OTU0342** | 18 | 13 | 21 | 0 | 0 | 0 | 0 | 1 | 1 |
| **OTU0343** | 2 | 0 | 4 | 0 | 0 | 1 | 0 | 0 | 0 |
| **OTU0344** | 9 | 20 | 21 | 0 | 1 | 0 | 0 | 0 | 0 |
| **OTU0345** | 0 | 1 | 0 | 0 | 0 | 0 | 34 | 1 | 12 |
| **OTU0346** | 4 | 20 | 30 | 0 | 3 | 0 | 1 | 0 | 0 |
| **OTU0347** | 0 | 0 | 1 | 0 | 0 | 1 | 0 | 48 | 1 |
| **OTU0348** | 85 | 119 | 171 | 0 | 28 | 18 | 1 | 1 | 0 |
| **OTU0349** | 0 | 0 | 0 | 0 | 17 | 0 | 0 | 0 | 28 |
| **OTU0350** | 0 | 1 | 1 | 18 | 5 | 0 | 0 | 0 | 22 |
| **OTU0351** | 3 | 2 | 4 | 0 | 1 | 1 | 0 | 0 | 0 |
| **OTU0352** | 31 | 15 | 21 | 1 | 9 | 0 | 0 | 0 | 0 |
| **OTU0353** | 1 | 1 | 1 | 17 | 6 | 8 | 10 | 10 | 15 |
| **OTU0354** | 2 | 7 | 27 | 1 | 2 | 0 | 0 | 0 | 0 |
| **OTU0355** | 7 | 13 | 9 | 0 | 17 | 0 | 0 | 0 | 0 |
| **OTU0356** | 12 | 14 | 15 | 0 | 3 | 3 | 0 | 0 | 0 |
| **OTU0357** | 6 | 2 | 5 | 2 | 0 | 1 | 0 | 0 | 0 |
| **OTU0358** | 1 | 0 | 0 | 3 | 4 | 3 | 1 | 0 | 2 |
| **OTU0359** | 19 | 7 | 21 | 0 | 12 | 0 | 1 | 0 | 1 |
| **OTU0360** | 1 | 0 | 0 | 4 | 1 | 3 | 7 | 0 | 1 |
| **OTU0361** | 0 | 0 | 0 | 1 | 0 | 0 | 1 | 42 | 0 |
| **OTU0362** | 0 | 0 | 0 | 4 | 3 | 3 | 5 | 3 | 4 |
| **OTU0363** | 0 | 4 | 0 | 0 | 35 | 0 | 0 | 0 | 0 |
| **OTU0364** | 1 | 0 | 0 | 24 | 0 | 1 | 0 | 0 | 6 |
| **OTU0365** | 3 | 2 | 1 | 0 | 2 | 0 | 0 | 0 | 1 |
| **OTU0366** | 0 | 0 | 0 | 43 | 0 | 0 | 0 | 0 | 0 |
| **OTU0367** | 22 | 16 | 34 | 0 | 10 | 17 | 0 | 0 | 0 |
| **OTU0368** | 0 | 0 | 1 | 1 | 0 | 0 | 47 | 0 | 0 |
| **OTU0369** | 0 | 0 | 0 | 0 | 0 | 0 | 27 | 0 | 0 |
| **OTU0370** | 1 | 10 | 8 | 10 | 4 | 1 | 1 | 2 | 1 |
| **OTU0371** | 40 | 56 | 82 | 0 | 5 | 17 | 1 | 0 | 0 |
| **OTU0372** | 0 | 0 | 0 | 3 | 1 | 0 | 3 | 0 | 0 |
| **OTU0373** | 9 | 12 | 9 | 0 | 1 | 1 | 0 | 0 | 0 |
| **OTU0374** | 2 | 0 | 1 | 13 | 0 | 0 | 0 | 0 | 36 |
| **OTU0375** | 4 | 4 | 21 | 0 | 0 | 0 | 0 | 0 | 0 |
| **OTU0376** | 7 | 13 | 24 | 0 | 0 | 1 | 0 | 0 | 1 |
| **OTU0377** | 90 | 60 | 147 | 0 | 78 | 31 | 2 | 0 | 0 |
| **OTU0378** | 3 | 4 | 17 | 5 | 63 | 76 | 31 | 62 | 58 |
| **OTU0379** | 34 | 12 | 19 | 0 | 0 | 0 | 0 | 0 | 0 |
| **OTU0380** | 0 | 1 | 1 | 0 | 0 | 0 | 0 | 0 | 0 |
| **OTU0381** | 8 | 12 | 22 | 0 | 2 | 6 | 0 | 0 | 0 |
| **OTU0382** | 92 | 88 | 125 | 0 | 1 | 1 | 0 | 0 | 0 |
| **OTU0383** | 13 | 0 | 3 | 0 | 0 | 11 | 0 | 0 | 0 |
| **OTU0384** | 30 | 3 | 13 | 0 | 0 | 0 | 0 | 0 | 0 |
| **OTU0385** | 0 | 3 | 17 | 1 | 0 | 0 | 0 | 0 | 0 |
| **OTU0386** | 0 | 1 | 4 | 7 | 18 | 14 | 0 | 2 | 1 |
| **OTU0387** | 0 | 0 | 0 | 5 | 0 | 1 | 0 | 1 | 0 |
| **OTU0388** | 1 | 0 | 1 | 0 | 0 | 0 | 0 | 0 | 0 |
| **OTU0389** | 1 | 2 | 1 | 46 | 40 | 33 | 1 | 2 | 29 |
| **OTU0390** | 0 | 0 | 0 | 15 | 0 | 7 | 2 | 0 | 0 |
| **OTU0391** | 2 | 0 | 4 | 8 | 20 | 6 | 1 | 0 | 10 |
| **OTU0392** | 8 | 3 | 8 | 0 | 1 | 2 | 0 | 0 | 0 |
| **OTU0393** | 0 | 0 | 0 | 38 | 0 | 0 | 0 | 1 | 0 |
| **OTU0394** | 9 | 6 | 15 | 0 | 7 | 6 | 0 | 0 | 0 |
| **OTU0395** | 10 | 4 | 5 | 0 | 2 | 2 | 0 | 0 | 1 |
| **OTU0396** | 28 | 36 | 79 | 0 | 18 | 23 | 0 | 0 | 0 |
| **OTU0397** | 0 | 0 | 1 | 20 | 15 | 5 | 10 | 11 | 13 |
| **OTU0398** | 35 | 32 | 40 | 1 | 8 | 9 | 0 | 0 | 1 |
| **OTU0399** | 26 | 11 | 1 | 0 | 15 | 0 | 0 | 0 | 0 |
| **OTU0400** | 0 | 0 | 0 | 1 | 17 | 0 | 1 | 0 | 11 |
| **OTU0401** | 0 | 1 | 13 | 0 | 11 | 17 | 1 | 0 | 0 |
| **OTU0402** | 0 | 1 | 3 | 11 | 6 | 1 | 1 | 12 | 1 |
| **OTU0403** | 1 | 0 | 2 | 20 | 22 | 2 | 0 | 0 | 0 |
| **OTU0404** | 2 | 1 | 2 | 0 | 1 | 0 | 0 | 0 | 0 |
| **OTU0405** | 2 | 0 | 2 | 1 | 0 | 0 | 0 | 41 | 0 |
| **OTU0406** | 5 | 2 | 4 | 0 | 0 | 0 | 0 | 0 | 0 |
| **OTU0407** | 12 | 2 | 6 | 0 | 0 | 15 | 0 | 0 | 0 |
| **OTU0408** | 1 | 0 | 2 | 0 | 0 | 1 | 0 | 0 | 0 |
| **OTU0409** | 17 | 16 | 37 | 0 | 5 | 2 | 1 | 0 | 0 |
| **OTU0410** | 11 | 3 | 15 | 0 | 2 | 3 | 0 | 0 | 0 |
| **OTU0411** | 15 | 7 | 13 | 0 | 1 | 4 | 0 | 1 | 0 |
| **OTU0412** | 0 | 0 | 0 | 1 | 1 | 0 | 35 | 0 | 0 |
| **OTU0413** | 1 | 0 | 0 | 19 | 0 | 0 | 0 | 22 | 0 |
| **OTU0414** | 15 | 8 | 12 | 0 | 8 | 6 | 0 | 0 | 0 |
| **OTU0415** | 4 | 1 | 1 | 0 | 0 | 0 | 0 | 0 | 28 |
| **OTU0416** | 9 | 3 | 12 | 0 | 1 | 0 | 0 | 0 | 0 |
| **OTU0417** | 4 | 2 | 2 | 0 | 0 | 1 | 0 | 0 | 0 |
| **OTU0418** | 0 | 0 | 0 | 0 | 0 | 0 | 1 | 1 | 0 |
| **OTU0419** | 0 | 3 | 1 | 111 | 26 | 31 | 45 | 19 | 22 |
| **OTU0420** | 2 | 0 | 0 | 60 | 13 | 24 | 23 | 19 | 9 |
| **OTU0421** | 0 | 1 | 5 | 5 | 22 | 3 | 1 | 9 | 7 |
| **OTU0422** | 4 | 4 | 22 | 4 | 1 | 0 | 5 | 0 | 0 |
| **OTU0423** | 9 | 4 | 15 | 0 | 0 | 0 | 0 | 0 | 0 |
| **OTU0424** | 15 | 21 | 37 | 0 | 16 | 10 | 0 | 1 | 0 |
| **OTU0425** | 0 | 0 | 0 | 44 | 12 | 25 | 30 | 22 | 23 |
| **OTU0426** | 2 | 1 | 2 | 42 | 17 | 36 | 37 | 13 | 35 |
| **OTU0427** | 0 | 0 | 0 | 14 | 5 | 1 | 8 | 8 | 3 |
| **OTU0428** | 5 | 5 | 1 | 0 | 2 | 1 | 0 | 0 | 0 |
| **OTU0429** | 0 | 1 | 2 | 0 | 14 | 0 | 0 | 0 | 22 |
| **OTU0430** | 68 | 60 | 99 | 2740 | 1334 | 1214 | 1761 | 1268 | 921 |
| **OTU0431** | 6 | 2 | 6 | 0 | 4 | 2 | 0 | 0 | 0 |
| **OTU0432** | 1 | 0 | 1 | 3 | 5 | 2 | 2 | 0 | 3 |
| **OTU0433** | 1 | 0 | 0 | 0 | 3 | 0 | 30 | 0 | 0 |
| **OTU0434** | 6 | 5 | 1 | 0 | 5 | 2 | 0 | 0 | 0 |
| **OTU0435** | 8 | 3 | 12 | 0 | 1 | 0 | 0 | 0 | 0 |
| **OTU0436** | 0 | 0 | 1 | 18 | 9 | 6 | 10 | 10 | 6 |
| **OTU0437** | 20 | 6 | 9 | 0 | 0 | 0 | 0 | 0 | 0 |
| **OTU0438** | 1 | 0 | 1 | 0 | 0 | 0 | 22 | 0 | 7 |
| **OTU0439** | 0 | 1 | 0 | 0 | 0 | 7 | 0 | 33 | 0 |
| **OTU0440** | 14 | 9 | 10 | 0 | 6 | 7 | 1 | 0 | 0 |
| **OTU0441** | 0 | 0 | 0 | 34 | 0 | 0 | 0 | 0 | 0 |
| **OTU0442** | 0 | 1 | 0 | 38 | 2 | 0 | 0 | 0 | 0 |
| **OTU0443** | 107 | 150 | 232 | 0 | 26 | 29 | 0 | 0 | 0 |
| **OTU0444** | 8 | 6 | 12 | 0 | 4 | 10 | 0 | 0 | 0 |
| **OTU0445** | 0 | 1 | 0 | 16 | 11 | 0 | 3 | 4 | 0 |
| **OTU0446** | 0 | 0 | 0 | 0 | 0 | 0 | 0 | 0 | 12 |
| **OTU0447** | 22 | 10 | 46 | 0 | 13 | 28 | 0 | 1 | 0 |
| **OTU0448** | 0 | 0 | 2 | 22 | 10 | 0 | 0 | 0 | 1 |
| **OTU0449** | 22 | 18 | 45 | 0 | 4 | 3 | 0 | 0 | 0 |
| **OTU0450** | 0 | 1 | 3 | 124 | 39 | 26 | 60 | 19 | 14 |
| **OTU0451** | 1 | 1 | 2 | 0 | 2 | 0 | 0 | 24 | 0 |
| **OTU0452** | 2 | 1 | 1 | 0 | 1 | 14 | 0 | 0 | 2 |
| **OTU0453** | 8 | 10 | 20 | 0 | 14 | 1 | 0 | 0 | 0 |
| **OTU0454** | 1 | 0 | 1 | 13 | 0 | 0 | 49 | 4 | 0 |
| **OTU0455** | 1 | 2 | 6 | 3 | 0 | 20 | 0 | 0 | 0 |
| **OTU0456** | 0 | 0 | 0 | 26 | 6 | 0 | 0 | 1 | 0 |
| **OTU0457** | 0 | 1 | 1 | 0 | 0 | 1 | 0 | 0 | 0 |
| **OTU0458** | 1 | 2 | 4 | 20 | 2 | 9 | 0 | 0 | 0 |
| **OTU0459** | 1 | 2 | 9 | 105 | 46 | 45 | 63 | 49 | 35 |
| **OTU0460** | 0 | 0 | 1 | 0 | 1 | 0 | 2 | 0 | 1 |
| **OTU0461** | 19 | 25 | 55 | 0 | 10 | 2 | 0 | 0 | 0 |
| **OTU0462** | 0 | 0 | 0 | 1 | 2 | 0 | 0 | 2 | 5 |
| **OTU0463** | 0 | 0 | 2 | 42 | 22 | 21 | 22 | 17 | 6 |
| **OTU0464** | 303 | 252 | 616 | 3 | 339 | 346 | 0 | 1 | 1 |
| **OTU0465** | 0 | 0 | 0 | 0 | 0 | 0 | 50 | 0 | 0 |
| **OTU0466** | 7 | 6 | 21 | 1 | 0 | 0 | 0 | 0 | 0 |
| **OTU0467** | 8 | 9 | 11 | 0 | 0 | 0 | 0 | 0 | 0 |
| **OTU0468** | 12 | 12 | 25 | 1 | 17 | 0 | 2 | 6 | 14 |
| **OTU0469** | 0 | 0 | 2 | 9 | 4 | 3 | 2 | 1 | 2 |
| **OTU0470** | 2 | 0 | 0 | 88 | 19 | 19 | 35 | 19 | 21 |
| **OTU0471** | 1 | 3 | 4 | 0 | 2 | 2 | 0 | 0 | 0 |
| **OTU0472** | 19 | 6 | 3 | 0 | 0 | 1 | 1 | 0 | 0 |
| **OTU0473** | 0 | 0 | 0 | 3 | 1 | 0 | 2 | 0 | 2 |
| **OTU0474** | 0 | 3 | 0 | 1 | 41 | 33 | 13 | 21 | 32 |
| **OTU0475** | 19 | 4 | 27 | 0 | 0 | 0 | 1 | 0 | 0 |
| **OTU0476** | 8 | 3 | 15 | 0 | 4 | 5 | 0 | 0 | 0 |
| **OTU0477** | 0 | 0 | 0 | 1 | 0 | 0 | 1 | 1 | 26 |
| **OTU0478** | 8 | 17 | 20 | 0 | 5 | 4 | 0 | 0 | 0 |
| **OTU0479** | 12 | 8 | 18 | 390 | 280 | 169 | 308 | 265 | 148 |
| **OTU0480** | 0 | 0 | 0 | 16 | 0 | 0 | 0 | 0 | 0 |
| **OTU0481** | 0 | 0 | 0 | 27 | 0 | 0 | 0 | 0 | 0 |
| **OTU0482** | 1 | 0 | 2 | 0 | 0 | 0 | 0 | 0 | 0 |
| **OTU0483** | 0 | 0 | 0 | 5 | 8 | 1 | 14 | 5 | 9 |
| **OTU0484** | 10 | 12 | 33 | 0 | 9 | 4 | 0 | 0 | 0 |
| **OTU0485** | 7 | 8 | 19 | 0 | 0 | 0 | 0 | 0 | 0 |
| **OTU0486** | 15 | 16 | 26 | 1 | 1 | 0 | 0 | 0 | 0 |
| **OTU0487** | 2 | 2 | 2 | 0 | 7 | 3 | 0 | 0 | 0 |
| **OTU0488** | 8 | 9 | 25 | 0 | 0 | 0 | 0 | 0 | 0 |
| **OTU0489** | 3 | 1 | 0 | 0 | 0 | 0 | 0 | 0 | 1 |
| **OTU0490** | 0 | 0 | 0 | 0 | 0 | 0 | 1 | 8 | 21 |
| **OTU0491** | 0 | 1 | 1 | 0 | 1 | 0 | 0 | 0 | 0 |
| **OTU0492** | 0 | 1 | 1 | 0 | 0 | 0 | 0 | 0 | 24 |
| **OTU0493** | 25 | 30 | 40 | 1 | 1 | 2 | 0 | 0 | 0 |
| **OTU0494** | 13 | 24 | 15 | 1 | 0 | 0 | 1 | 0 | 0 |
| **OTU0495** | 3 | 13 | 17 | 0 | 0 | 0 | 0 | 0 | 0 |
| **OTU0496** | 8 | 10 | 21 | 0 | 1 | 1 | 0 | 0 | 0 |
| **OTU0497** | 2 | 1 | 1 | 53 | 20 | 31 | 32 | 28 | 17 |
| **OTU0498** | 4 | 3 | 11 | 57 | 3 | 4 | 1 | 5 | 1 |
| **OTU0499** | 7155 | 5853 | 10395 | 24 | 3157 | 3282 | 20 | 15 | 18 |
| **OTU0500** | 20 | 11 | 37 | 4 | 0 | 0 | 0 | 0 | 0 |
| **OTU0501** | 19 | 9 | 14 | 0 | 0 | 0 | 0 | 0 | 0 |
| **OTU0502** | 2 | 1 | 2 | 0 | 0 | 2 | 0 | 0 | 0 |
| **OTU0503** | 15 | 6 | 13 | 0 | 0 | 1 | 0 | 0 | 0 |
| **OTU0504** | 17 | 10 | 21 | 0 | 2 | 5 | 0 | 0 | 0 |
| **OTU0505** | 3 | 0 | 2 | 47 | 17 | 19 | 37 | 16 | 25 |
| **OTU0506** | 6 | 3 | 5 | 0 | 0 | 0 | 0 | 0 | 0 |
| **OTU0507** | 2 | 0 | 0 | 5 | 1 | 1 | 2 | 1 | 0 |
| **OTU0508** | 0 | 2 | 0 | 0 | 0 | 0 | 0 | 0 | 25 |
| **OTU0509** | 1 | 6 | 26 | 0 | 0 | 0 | 0 | 0 | 1 |
| **OTU0510** | 0 | 0 | 2 | 0 | 0 | 1 | 0 | 12 | 2 |
| **OTU0511** | 6 | 8 | 6 | 0 | 1 | 4 | 0 | 0 | 0 |
| **OTU0512** | 0 | 0 | 0 | 4 | 5 | 1 | 9 | 1 | 2 |
| **OTU0513** | 0 | 0 | 0 | 7 | 2 | 1 | 1 | 1 | 1 |
| **OTU0514** | 10 | 11 | 15 | 0 | 1 | 2 | 0 | 0 | 0 |
| **OTU0515** | 805 | 566 | 1595 | 14 | 109 | 89 | 6 | 7 | 6 |
| **OTU0516** | 127 | 82 | 109 | 1 | 20 | 16 | 2 | 1 | 0 |
| **OTU0517** | 0 | 0 | 0 | 1 | 0 | 1 | 2 | 0 | 0 |
| **OTU0518** | 3 | 2 | 4 | 51 | 12 | 10 | 16 | 15 | 14 |
| **OTU0519** | 1 | 0 | 1 | 0 | 0 | 0 | 0 | 22 | 0 |
| **OTU0520** | 1 | 1 | 1 | 32 | 23 | 17 | 22 | 9 | 16 |
| **OTU0521** | 22 | 0 | 1 | 1 | 0 | 0 | 1 | 0 | 0 |
| **OTU0522** | 202 | 226 | 306 | 0 | 40 | 44 | 0 | 1 | 1 |
| **OTU0523** | 0 | 0 | 0 | 23 | 0 | 0 | 0 | 2 | 0 |
| **OTU0524** | 35 | 37 | 68 | 0 | 21 | 12 | 0 | 0 | 0 |
| **OTU0525** | 12 | 5 | 4 | 0 | 0 | 2 | 0 | 0 | 0 |
| **OTU0526** | 0 | 0 | 0 | 1 | 0 | 2 | 1 | 0 | 0 |
| **OTU0527** | 15 | 22 | 79 | 0 | 0 | 0 | 0 | 0 | 0 |
| **OTU0528** | 10 | 0 | 4 | 0 | 14 | 0 | 0 | 0 | 0 |
| **OTU0529** | 1 | 4 | 3 | 48 | 19 | 18 | 61 | 34 | 33 |
| **OTU0530** | 0 | 0 | 0 | 30 | 0 | 1 | 0 | 0 | 0 |
| **OTU0531** | 0 | 0 | 0 | 16 | 4 | 4 | 9 | 3 | 4 |
| **OTU0532** | 1 | 0 | 0 | 0 | 0 | 24 | 0 | 0 | 0 |
| **OTU0533** | 1 | 0 | 0 | 8 | 4 | 4 | 4 | 2 | 2 |
| **OTU0534** | 0 | 0 | 0 | 12 | 2 | 4 | 15 | 9 | 3 |
| **OTU0535** | 0 | 0 | 0 | 11 | 0 | 0 | 0 | 0 | 22 |
| **OTU0536** | 6 | 5 | 12 | 0 | 0 | 0 | 0 | 0 | 0 |
| **OTU0537** | 231 | 290 | 366 | 4 | 9 | 11 | 2 | 2 | 2 |
| **OTU0538** | 2 | 3 | 16 | 1 | 0 | 0 | 0 | 0 | 0 |
| **OTU0539** | 0 | 0 | 1 | 1 | 3 | 0 | 5 | 1 | 1 |
| **OTU0540** | 34 | 44 | 62 | 0 | 11 | 12 | 1 | 0 | 0 |
| **OTU0541** | 252 | 186 | 409 | 0 | 329 | 239 | 1 | 2 | 1 |
| **OTU0542** | 19 | 5 | 1 | 0 | 0 | 0 | 0 | 0 | 0 |
| **OTU0543** | 1 | 2 | 1 | 0 | 1 | 0 | 0 | 0 | 0 |
| **OTU0544** | 0 | 0 | 0 | 21 | 0 | 0 | 0 | 13 | 0 |
| **OTU0545** | 6 | 7 | 15 | 0 | 0 | 0 | 0 | 0 | 0 |
| **OTU0546** | 0 | 0 | 0 | 0 | 4 | 0 | 0 | 0 | 24 |
| **OTU0547** | 32 | 43 | 45 | 0 | 14 | 5 | 0 | 1 | 1 |
| **OTU0548** | 0 | 1 | 3 | 21 | 5 | 0 | 1 | 30 | 9 |
| **OTU0549** | 69 | 52 | 69 | 5 | 9 | 10 | 0 | 2 | 0 |
| **OTU0550** | 68 | 61 | 144 | 1 | 62 | 71 | 0 | 0 | 1 |
| **OTU0551** | 0 | 7 | 26 | 0 | 0 | 0 | 1 | 0 | 0 |
| **OTU0552** | 27 | 34 | 25 | 0 | 8 | 6 | 0 | 0 | 0 |
| **OTU0553** | 8 | 8 | 7 | 1 | 0 | 0 | 0 | 0 | 0 |
| **OTU0554** | 54 | 57 | 110 | 0 | 17 | 21 | 0 | 0 | 0 |
| **OTU0555** | 6 | 6 | 5 | 0 | 1 | 0 | 0 | 0 | 0 |
| **OTU0556** | 38 | 37 | 72 | 0 | 16 | 4 | 0 | 0 | 0 |
| **OTU0557** | 10 | 10 | 20 | 0 | 10 | 6 | 0 | 0 | 0 |
| **OTU0558** | 1 | 1 | 0 | 2 | 6 | 2 | 4 | 2 | 10 |
| **OTU0559** | 6 | 7 | 23 | 0 | 1 | 0 | 0 | 0 | 0 |
| **OTU0560** | 0 | 1 | 1 | 0 | 0 | 0 | 0 | 0 | 0 |
| **OTU0561** | 3 | 0 | 1 | 0 | 0 | 1 | 0 | 0 | 0 |
| **OTU0562** | 0 | 0 | 0 | 0 | 1 | 0 | 1 | 0 | 2 |
| **OTU0563** | 3 | 10 | 14 | 30 | 12 | 8 | 17 | 3 | 7 |
| **OTU0564** | 0 | 0 | 1 | 30 | 2 | 0 | 0 | 0 | 0 |
| **OTU0565** | 0 | 0 | 0 | 2 | 13 | 11 | 0 | 0 | 0 |
| **OTU0566** | 0 | 3 | 1 | 0 | 15 | 6 | 1 | 0 | 2 |
| **OTU0567** | 1 | 0 | 2 | 45 | 38 | 32 | 2 | 37 | 29 |
| **OTU0568** | 0 | 1 | 1 | 0 | 0 | 0 | 0 | 0 | 0 |
| **OTU0569** | 163 | 138 | 253 | 2 | 97 | 79 | 0 | 0 | 1 |
| **OTU0570** | 3 | 0 | 17 | 1 | 0 | 0 | 0 | 0 | 0 |
| **OTU0571** | 0 | 0 | 0 | 0 | 0 | 23 | 0 | 0 | 0 |
| **OTU0572** | 41 | 32 | 80 | 3 | 7 | 13 | 1 | 0 | 0 |
| **OTU0573** | 0 | 0 | 0 | 23 | 0 | 0 | 0 | 0 | 0 |
| **OTU0574** | 0 | 0 | 0 | 25 | 0 | 0 | 0 | 0 | 1 |
| **OTU0575** | 53 | 43 | 132 | 1 | 64 | 55 | 0 | 0 | 0 |
| **OTU0576** | 0 | 0 | 0 | 2 | 0 | 0 | 1 | 0 | 0 |
| **OTU0577** | 1 | 2 | 0 | 44 | 16 | 15 | 36 | 16 | 18 |
| **OTU0578** | 53 | 60 | 108 | 0 | 10 | 11 | 0 | 1 | 1 |
| **OTU0579** | 0 | 0 | 0 | 0 | 0 | 0 | 1 | 0 | 1 |
| **OTU0580** | 0 | 0 | 0 | 4 | 1 | 1 | 1 | 0 | 0 |
| **OTU0581** | 1 | 0 | 0 | 17 | 0 | 0 | 0 | 0 | 7 |
| **OTU0582** | 7 | 1 | 9 | 0 | 4 | 0 | 0 | 0 | 0 |
| **OTU0583** | 0 | 0 | 0 | 0 | 0 | 0 | 0 | 0 | 2 |
| **OTU0584** | 41 | 56 | 79 | 1 | 5 | 5 | 0 | 1 | 0 |
| **OTU0585** | 0 | 0 | 1 | 18 | 1 | 3 | 5 | 1 | 3 |
| **OTU0586** | 0 | 1 | 0 | 40 | 6 | 9 | 12 | 5 | 7 |
| **OTU0587** | 5 | 3 | 4 | 1 | 0 | 1 | 0 | 0 | 0 |
| **OTU0588** | 34 | 42 | 63 | 0 | 12 | 8 | 0 | 0 | 0 |
| **OTU0589** | 58 | 66 | 125 | 1 | 20 | 18 | 0 | 0 | 0 |
| **OTU0590** | 5 | 5 | 26 | 0 | 3 | 1 | 0 | 0 | 0 |
| **OTU0591** | 0 | 0 | 0 | 0 | 0 | 0 | 0 | 0 | 6 |
| **OTU0592** | 2 | 1 | 18 | 0 | 8 | 0 | 0 | 0 | 0 |
| **OTU0593** | 0 | 0 | 0 | 8 | 5 | 6 | 3 | 4 | 3 |
| **OTU0594** | 12 | 8 | 12 | 0 | 1 | 0 | 0 | 0 | 0 |
| **OTU0595** | 1 | 0 | 1 | 1 | 3 | 13 | 0 | 0 | 0 |
| **OTU0596** | 0 | 0 | 0 | 3 | 3 | 2 | 6 | 0 | 0 |
| **OTU0597** | 1 | 2 | 0 | 0 | 11 | 0 | 0 | 0 | 8 |
| **OTU0598** | 0 | 2 | 3 | 0 | 0 | 23 | 0 | 0 | 0 |
| **OTU0599** | 216 | 159 | 269 | 5 | 35 | 30 | 4 | 0 | 3 |
| **OTU0600** | 1 | 0 | 0 | 0 | 0 | 18 | 0 | 0 | 0 |
| **OTU0601** | 3 | 1 | 4 | 0 | 10 | 0 | 0 | 0 | 0 |
| **OTU0602** | 0 | 0 | 0 | 1 | 3 | 2 | 1 | 1 | 1 |
| **OTU0603** | 0 | 0 | 0 | 26 | 0 | 0 | 0 | 0 | 0 |
| **OTU0604** | 3 | 8 | 13 | 0 | 2 | 4 | 0 | 0 | 1 |
| **OTU0605** | 12 | 2 | 14 | 1 | 0 | 0 | 0 | 1 | 1 |
| **OTU0606** | 1 | 0 | 0 | 3 | 4 | 5 | 8 | 2 | 8 |
| **OTU0607** | 15 | 19 | 28 | 402 | 271 | 241 | 459 | 241 | 220 |
| **OTU0608** | 0 | 0 | 0 | 20 | 0 | 0 | 0 | 0 | 0 |
| **OTU0609** | 0 | 0 | 0 | 0 | 1 | 2 | 3 | 0 | 0 |
| **OTU0610** | 0 | 1 | 4 | 0 | 3 | 1 | 0 | 0 | 1 |
| **OTU0611** | 318 | 204 | 416 | 3 | 58 | 49 | 1 | 1 | 1 |
| **OTU0612** | 0 | 0 | 0 | 2 | 0 | 0 | 0 | 1 | 2 |
| **OTU0613** | 15 | 13 | 39 | 0 | 10 | 8 | 0 | 0 | 0 |
| **OTU0614** | 4 | 3 | 8 | 0 | 0 | 0 | 1 | 0 | 0 |
| **OTU0615** | 10 | 9 | 16 | 0 | 3 | 0 | 0 | 0 | 0 |
| **OTU0616** | 6 | 4 | 12 | 206 | 81 | 109 | 117 | 72 | 91 |
| **OTU0617** | 38 | 47 | 83 | 1 | 4 | 1 | 0 | 1 | 0 |
| **OTU0618** | 5 | 2 | 6 | 0 | 0 | 1 | 0 | 0 | 0 |
| **OTU0619** | 3 | 4 | 10 | 0 | 0 | 0 | 0 | 0 | 0 |
| **OTU0620** | 4 | 11 | 19 | 0 | 1 | 2 | 0 | 0 | 0 |
| **OTU0621** | 0 | 1 | 0 | 0 | 0 | 2 | 0 | 23 | 0 |
| **OTU0622** | 5 | 3 | 12 | 0 | 0 | 0 | 0 | 0 | 0 |
| **OTU0623** | 0 | 0 | 2 | 0 | 16 | 1 | 0 | 0 | 0 |
| **OTU0624** | 1 | 1 | 0 | 0 | 0 | 2 | 0 | 0 | 0 |
| **OTU0625** | 617 | 393 | 1121 | 3 | 76 | 54 | 4 | 5 | 3 |
| **OTU0626** | 1 | 2 | 1 | 37 | 25 | 21 | 32 | 27 | 5 |
| **OTU0627** | 1 | 0 | 1 | 30 | 10 | 7 | 1 | 7 | 5 |
| **OTU0628** | 1 | 6 | 6 | 0 | 6 | 1 | 0 | 1 | 0 |
| **OTU0629** | 13 | 21 | 27 | 0 | 0 | 1 | 1 | 0 | 0 |
| **OTU0630** | 6 | 3 | 2 | 0 | 8 | 0 | 0 | 0 | 0 |
| **OTU0631** | 1 | 1 | 7 | 39 | 31 | 29 | 44 | 35 | 24 |
| **OTU0632** | 2 | 1 | 0 | 7 | 0 | 7 | 0 | 0 | 0 |
| **OTU0633** | 8 | 11 | 36 | 1 | 0 | 0 | 0 | 0 | 0 |
| **OTU0634** | 41 | 19 | 49 | 0 | 12 | 7 | 0 | 0 | 0 |
| **OTU0635** | 0 | 1 | 0 | 4 | 2 | 1 | 4 | 3 | 1 |
| **OTU0636** | 0 | 5 | 14 | 0 | 1 | 0 | 0 | 0 | 0 |
| **OTU0637** | 2 | 7 | 9 | 0 | 0 | 0 | 0 | 0 | 0 |
| **OTU0638** | 0 | 0 | 1 | 14 | 8 | 3 | 4 | 0 | 2 |
| **OTU0639** | 5 | 3 | 3 | 0 | 0 | 2 | 0 | 0 | 0 |
| **OTU0640** | 1 | 1 | 4 | 0 | 13 | 0 | 0 | 0 | 0 |
| **OTU0641** | 3 | 4 | 0 | 17 | 0 | 0 | 0 | 0 | 0 |
| **OTU0642** | 24 | 21 | 33 | 0 | 8 | 11 | 0 | 0 | 1 |
| **OTU0643** | 0 | 1 | 0 | 45 | 21 | 26 | 31 | 19 | 23 |
| **OTU0644** | 2 | 4 | 2 | 0 | 21 | 0 | 0 | 0 | 0 |
| **OTU0645** | 0 | 0 | 0 | 0 | 0 | 0 | 0 | 0 | 22 |
| **OTU0646** | 6 | 3 | 2 | 2 | 1 | 0 | 23 | 0 | 0 |
| **OTU0647** | 0 | 1 | 0 | 1 | 0 | 0 | 0 | 0 | 1 |
| **OTU0648** | 10 | 12 | 13 | 1 | 0 | 0 | 0 | 0 | 0 |
| **OTU0649** | 0 | 0 | 0 | 0 | 0 | 19 | 0 | 0 | 0 |
| **OTU0650** | 0 | 0 | 0 | 0 | 19 | 0 | 1 | 0 | 0 |
| **OTU0651** | 10 | 2 | 13 | 0 | 0 | 0 | 0 | 1 | 0 |
| **OTU0652** | 0 | 0 | 0 | 3 | 0 | 0 | 1 | 1 | 1 |
| **OTU0653** | 2 | 8 | 8 | 2 | 2 | 3 | 0 | 0 | 0 |
| **OTU0654** | 11 | 10 | 14 | 448 | 271 | 306 | 303 | 235 | 203 |
| **OTU0655** | 0 | 3 | 0 | 0 | 8 | 0 | 0 | 0 | 0 |
| **OTU0656** | 1 | 0 | 4 | 0 | 0 | 16 | 0 | 0 | 0 |
| **OTU0657** | 921 | 874 | 1371 | 2 | 299 | 337 | 3 | 2 | 0 |
| **OTU0658** | 1 | 1 | 1 | 9 | 3 | 5 | 3 | 4 | 1 |
| **OTU0659** | 6 | 3 | 9 | 54 | 17 | 16 | 26 | 25 | 12 |
| **OTU0660** | 3 | 0 | 3 | 0 | 0 | 18 | 0 | 0 | 0 |
| **OTU0661** | 0 | 0 | 0 | 0 | 0 | 19 | 0 | 0 | 0 |
| **OTU0662** | 10 | 6 | 8 | 0 | 0 | 0 | 0 | 0 | 0 |
| **OTU0663** | 14 | 9 | 22 | 0 | 7 | 1 | 0 | 0 | 0 |
| **OTU0664** | 108 | 58 | 180 | 2 | 9 | 9 | 2 | 2 | 2 |
| **OTU0665** | 9 | 7 | 11 | 0 | 3 | 4 | 0 | 0 | 0 |
| **OTU0666** | 10 | 8 | 14 | 12 | 0 | 1 | 34 | 0 | 2 |
| **OTU0667** | 1 | 0 | 8 | 0 | 8 | 0 | 1 | 0 | 0 |
| **OTU0668** | 2 | 1 | 3 | 10 | 22 | 19 | 10 | 31 | 23 |
| **OTU0669** | 2 | 2 | 2 | 13 | 22 | 23 | 11 | 19 | 25 |
| **OTU0670** | 0 | 0 | 0 | 0 | 0 | 2 | 3 | 1 | 0 |
| **OTU0671** | 0 | 1 | 1 | 18 | 9 | 16 | 13 | 10 | 9 |
| **OTU0672** | 0 | 0 | 5 | 75 | 39 | 44 | 38 | 43 | 38 |
| **OTU0673** | 83 | 69 | 136 | 2 | 12 | 5 | 0 | 0 | 0 |
| **OTU0674** | 57 | 53 | 68 | 2 | 7 | 17 | 0 | 2 | 0 |
| **OTU0675** | 0 | 0 | 2 | 38 | 6 | 6 | 12 | 5 | 9 |
| **OTU0676** | 0 | 1 | 1 | 12 | 3 | 5 | 11 | 5 | 1 |
| **OTU0677** | 16 | 29 | 29 | 0 | 3 | 3 | 0 | 1 | 0 |
| **OTU0678** | 0 | 0 | 0 | 2 | 4 | 2 | 1 | 2 | 3 |
| **OTU0679** | 3 | 1 | 1 | 4 | 41 | 29 | 17 | 24 | 27 |
| **OTU0680** | 1 | 2 | 5 | 0 | 0 | 13 | 0 | 0 | 0 |
| **OTU0681** | 6 | 14 | 7 | 0 | 3 | 1 | 0 | 0 | 0 |
| **OTU0682** | 8 | 5 | 17 | 1 | 0 | 0 | 0 | 0 | 0 |
| **OTU0683** | 1 | 1 | 1 | 26 | 6 | 5 | 8 | 4 | 4 |
| **OTU0684** | 2 | 1 | 8 | 0 | 0 | 0 | 0 | 0 | 0 |
| **OTU0685** | 0 | 0 | 0 | 6 | 1 | 0 | 0 | 0 | 0 |
| **OTU0686** | 1 | 0 | 0 | 18 | 0 | 0 | 0 | 0 | 0 |
| **OTU0687** | 0 | 3 | 2 | 4 | 8 | 5 | 5 | 6 | 0 |
| **OTU0688** | 0 | 0 | 0 | 0 | 1 | 2 | 2 | 0 | 2 |
| **OTU0689** | 6 | 7 | 3 | 0 | 0 | 0 | 0 | 0 | 0 |
| **OTU0690** | 7 | 5 | 7 | 0 | 0 | 1 | 0 | 0 | 0 |
| **OTU0691** | 0 | 0 | 0 | 0 | 0 | 4 | 0 | 0 | 16 |
| **OTU0692** | 0 | 0 | 0 | 4 | 1 | 0 | 1 | 0 | 2 |
| **OTU0693** | 26 | 19 | 21 | 3 | 6 | 3 | 0 | 0 | 1 |
| **OTU0694** | 62 | 42 | 97 | 3 | 15 | 22 | 2 | 1 | 0 |
| **OTU0695** | 0 | 0 | 0 | 6 | 2 | 2 | 5 | 1 | 2 |
| **OTU0696** | 0 | 0 | 0 | 20 | 0 | 0 | 0 | 0 | 0 |
| **OTU0697** | 22 | 36 | 38 | 0 | 4 | 11 | 0 | 0 | 0 |
| **OTU0698** | 3 | 0 | 0 | 0 | 0 | 15 | 0 | 0 | 0 |
| **OTU0699** | 7 | 4 | 10 | 2 | 1 | 1 | 1 | 0 | 1 |
| **OTU0700** | 7 | 4 | 7 | 0 | 0 | 0 | 0 | 0 | 0 |
| **OTU0701** | 3 | 7 | 8 | 0 | 1 | 1 | 0 | 0 | 0 |
| **OTU0702** | 1 | 1 | 2 | 48 | 9 | 8 | 11 | 10 | 11 |
| **OTU0703** | 88 | 75 | 128 | 0 | 43 | 28 | 0 | 0 | 0 |
| **OTU0704** | 4 | 6 | 14 | 0 | 0 | 0 | 0 | 0 | 0 |
| **OTU0705** | 0 | 0 | 0 | 0 | 0 | 1 | 0 | 0 | 14 |
| **OTU0706** | 0 | 1 | 0 | 5 | 7 | 1 | 5 | 1 | 4 |
| **OTU0707** | 1 | 1 | 0 | 13 | 0 | 0 | 1 | 0 | 0 |
| **OTU0708** | 0 | 0 | 0 | 0 | 0 | 17 | 0 | 0 | 0 |
| **OTU0709** | 0 | 0 | 0 | 20 | 0 | 0 | 0 | 0 | 0 |
| **OTU0710** | 0 | 2 | 1 | 21 | 19 | 17 | 20 | 16 | 17 |
| **OTU0711** | 7 | 5 | 18 | 0 | 3 | 2 | 0 | 0 | 0 |
| **OTU0712** | 0 | 0 | 0 | 12 | 5 | 4 | 5 | 1 | 6 |
| **OTU0713** | 8 | 1 | 4 | 0 | 5 | 0 | 0 | 0 | 0 |
| **OTU0714** | 1 | 5 | 4 | 0 | 0 | 0 | 0 | 0 | 0 |
| **OTU0715** | 0 | 3 | 2 | 19 | 10 | 6 | 16 | 6 | 8 |
| **OTU0716** | 10 | 9 | 17 | 0 | 15 | 10 | 0 | 0 | 0 |
| **OTU0717** | 0 | 0 | 0 | 16 | 0 | 0 | 0 | 0 | 0 |
| **OTU0718** | 1 | 0 | 0 | 0 | 4 | 5 | 2 | 3 | 3 |
| **OTU0719** | 1 | 0 | 1 | 0 | 0 | 12 | 0 | 0 | 0 |
| **OTU0720** | 574 | 374 | 1040 | 2 | 309 | 216 | 1 | 0 | 1 |
| **OTU0721** | 56 | 57 | 84 | 0 | 1 | 2 | 0 | 2 | 0 |
| **OTU0722** | 2 | 1 | 0 | 0 | 0 | 0 | 0 | 0 | 0 |
| **OTU0723** | 6 | 5 | 7 | 0 | 0 | 0 | 0 | 0 | 0 |
| **OTU0724** | 0 | 0 | 0 | 4 | 1 | 0 | 1 | 0 | 16 |
| **OTU0725** | 8 | 7 | 10 | 0 | 3 | 0 | 0 | 0 | 0 |
| **OTU0726** | 35 | 50 | 96 | 1 | 17 | 14 | 0 | 0 | 0 |
| **OTU0727** | 2 | 4 | 6 | 179 | 80 | 43 | 155 | 50 | 22 |
| **OTU0728** | 3 | 2 | 4 | 1 | 0 | 0 | 0 | 0 | 0 |
| **OTU0729** | 8 | 6 | 9 | 2 | 0 | 0 | 0 | 0 | 0 |
| **OTU0730** | 0 | 2 | 4 | 0 | 0 | 0 | 0 | 0 | 0 |
| **OTU0731** | 0 | 0 | 0 | 1 | 0 | 0 | 0 | 17 | 0 |
| **OTU0732** | 0 | 0 | 1 | 0 | 0 | 0 | 1 | 19 | 17 |
| **OTU0733** | 0 | 0 | 1 | 3 | 6 | 5 | 5 | 8 | 2 |
| **OTU0734** | 20 | 23 | 25 | 1 | 4 | 10 | 0 | 1 | 0 |
| **OTU0735** | 20 | 8 | 18 | 0 | 6 | 4 | 0 | 0 | 0 |
| **OTU0736** | 0 | 0 | 0 | 3 | 13 | 0 | 0 | 0 | 1 |
| **OTU0737** | 0 | 0 | 0 | 0 | 1 | 0 | 0 | 0 | 14 |
| **OTU0738** | 7 | 3 | 10 | 11 | 8 | 8 | 9 | 8 | 10 |
| **OTU0739** | 29 | 40 | 62 | 0 | 5 | 4 | 0 | 0 | 1 |
| **OTU0740** | 2 | 2 | 6 | 20 | 41 | 31 | 21 | 29 | 34 |
| **OTU0741** | 55 | 28 | 29 | 3 | 1 | 0 | 1 | 1 | 0 |
| **OTU0742** | 1 | 0 | 3 | 0 | 1 | 1 | 0 | 0 | 0 |
| **OTU0743** | 5 | 5 | 2 | 0 | 0 | 5 | 0 | 0 | 0 |
| **OTU0744** | 3 | 0 | 3 | 36 | 10 | 9 | 16 | 12 | 13 |
| **OTU0745** | 7 | 15 | 24 | 0 | 0 | 5 | 0 | 0 | 0 |
| **OTU0746** | 0 | 0 | 0 | 17 | 6 | 5 | 7 | 3 | 0 |
| **OTU0747** | 2 | 3 | 6 | 155 | 53 | 53 | 81 | 49 | 36 |
| **OTU0748** | 10 | 13 | 24 | 0 | 1 | 2 | 1 | 0 | 0 |
| **OTU0749** | 1 | 2 | 0 | 16 | 0 | 0 | 0 | 0 | 0 |
| **OTU0750** | 4 | 2 | 12 | 0 | 0 | 0 | 0 | 0 | 0 |
| **OTU0751** | 0 | 1 | 0 | 17 | 0 | 0 | 0 | 0 | 1 |
| **OTU0752** | 0 | 0 | 0 | 0 | 13 | 0 | 0 | 0 | 0 |
| **OTU0753** | 0 | 0 | 3 | 53 | 16 | 15 | 38 | 21 | 19 |
| **OTU0754** | 0 | 0 | 0 | 0 | 0 | 14 | 0 | 0 | 0 |
| **OTU0755** | 1 | 0 | 3 | 18 | 6 | 6 | 10 | 1 | 9 |
| **OTU0756** | 0 | 1 | 0 | 37 | 10 | 7 | 18 | 6 | 3 |
| **OTU0757** | 6 | 0 | 2 | 0 | 15 | 6 | 0 | 0 | 1 |
| **OTU0758** | 1 | 0 | 0 | 15 | 0 | 0 | 0 | 0 | 0 |
| **OTU0759** | 0 | 0 | 0 | 0 | 0 | 0 | 0 | 0 | 16 |
| **OTU0760** | 20 | 14 | 25 | 29 | 535 | 383 | 122 | 275 | 285 |
| **OTU0761** | 110 | 140 | 170 | 3 | 13 | 10 | 3 | 3 | 8 |
| **OTU0762** | 61 | 61 | 133 | 1 | 74 | 70 | 0 | 1 | 1 |
| **OTU0763** | 64 | 63 | 117 | 2 | 24 | 25 | 1 | 0 | 0 |
| **OTU0764** | 9 | 3 | 3 | 0 | 0 | 0 | 2 | 1 | 0 |
| **OTU0765** | 0 | 1 | 0 | 1 | 0 | 9 | 0 | 0 | 0 |
| **OTU0766** | 0 | 0 | 0 | 15 | 0 | 0 | 0 | 0 | 0 |
| **OTU0767** | 0 | 0 | 0 | 6 | 3 | 3 | 0 | 0 | 1 |
| **OTU0768** | 9 | 2 | 6 | 56 | 11 | 0 | 5 | 2 | 1 |
| **OTU0769** | 1 | 0 | 0 | 1 | 0 | 18 | 0 | 0 | 0 |
| **OTU0770** | 17 | 9 | 18 | 0 | 5 | 4 | 0 | 0 | 2 |
| **OTU0771** | 0 | 0 | 0 | 13 | 0 | 0 | 0 | 0 | 0 |
| **OTU0772** | 146 | 68 | 185 | 3 | 9 | 2 | 0 | 0 | 3 |
| **OTU0773** | 0 | 0 | 0 | 0 | 0 | 14 | 0 | 0 | 0 |
| **OTU0774** | 26 | 20 | 30 | 0 | 4 | 5 | 0 | 0 | 0 |
| **OTU0775** | 2 | 0 | 0 | 2 | 2 | 5 | 6 | 2 | 1 |
| **OTU0776** | 302 | 280 | 619 | 1 | 345 | 347 | 0 | 1 | 0 |
| **OTU0777** | 607 | 588 | 941 | 8 | 95 | 33 | 12 | 6 | 5 |
| **OTU0778** | 1 | 0 | 0 | 0 | 0 | 16 | 0 | 0 | 0 |
| **OTU0779** | 16 | 12 | 27 | 0 | 0 | 0 | 0 | 0 | 0 |
| **OTU0780** | 34 | 34 | 48 | 1 | 0 | 1 | 0 | 0 | 0 |
| **OTU0781** | 14 | 7 | 12 | 0 | 0 | 8 | 0 | 0 | 0 |
| **OTU0782** | 56 | 38 | 70 | 1805 | 899 | 762 | 1119 | 842 | 588 |
| **OTU0783** | 1 | 0 | 0 | 47 | 16 | 10 | 20 | 8 | 14 |
| **OTU0784** | 1 | 2 | 7 | 38 | 89 | 67 | 44 | 74 | 86 |
| **OTU0785** | 1 | 0 | 2 | 30 | 9 | 5 | 9 | 9 | 8 |
| **OTU0786** | 19 | 24 | 32 | 1 | 4 | 1 | 0 | 0 | 0 |
| **OTU0787** | 0 | 0 | 0 | 14 | 0 | 0 | 0 | 0 | 0 |
| **OTU0788** | 0 | 0 | 1 | 1 | 0 | 0 | 0 | 0 | 0 |
| **OTU0789** | 0 | 0 | 1 | 0 | 14 | 0 | 0 | 0 | 0 |
| **OTU0790** | 0 | 0 | 0 | 7 | 6 | 1 | 5 | 2 | 0 |
| **OTU0791** | 0 | 0 | 0 | 5 | 1 | 1 | 4 | 2 | 1 |
| **OTU0792** | 8 | 0 | 8 | 62 | 17 | 22 | 21 | 19 | 9 |
| **OTU0793** | 1 | 6 | 12 | 5 | 0 | 3 | 1 | 0 | 0 |
| **OTU0794** | 15 | 4 | 11 | 27 | 0 | 0 | 0 | 0 | 0 |
| **OTU0795** | 4 | 10 | 9 | 0 | 0 | 1 | 0 | 0 | 0 |
| **OTU0796** | 102 | 99 | 179 | 1 | 19 | 27 | 0 | 0 | 0 |
| **OTU0797** | 19 | 47 | 85 | 0 | 18 | 16 | 0 | 0 | 1 |
| **OTU0798** | 0 | 0 | 2 | 80 | 11 | 14 | 14 | 8 | 7 |
| **OTU0799** | 0 | 0 | 0 | 29 | 5 | 8 | 8 | 12 | 3 |
| **OTU0800** | 14 | 19 | 37 | 0 | 10 | 5 | 0 | 0 | 0 |
| **OTU0801** | 59 | 51 | 112 | 0 | 11 | 8 | 0 | 0 | 0 |
| **OTU0802** | 2 | 4 | 13 | 0 | 0 | 0 | 0 | 0 | 0 |
| **OTU0803** | 3 | 1 | 6 | 206 | 65 | 57 | 72 | 70 | 46 |
| **OTU0804** | 1 | 1 | 0 | 0 | 0 | 0 | 0 | 0 | 0 |
| **OTU0805** | 2 | 1 | 4 | 40 | 52 | 48 | 46 | 44 | 31 |
| **OTU0806** | 6 | 2 | 8 | 80 | 36 | 29 | 105 | 41 | 31 |
| **OTU0807** | 0 | 1 | 9 | 0 | 6 | 0 | 0 | 0 | 0 |
| **OTU0808** | 1 | 0 | 0 | 7 | 7 | 6 | 7 | 2 | 4 |
| **OTU0809** | 0 | 0 | 1 | 5 | 6 | 0 | 4 | 4 | 4 |
| **OTU0810** | 23 | 13 | 44 | 0 | 7 | 3 | 0 | 0 | 0 |
| **OTU0811** | 0 | 0 | 0 | 1 | 1 | 0 | 1 | 1 | 0 |
| **OTU0812** | 35 | 26 | 48 | 1 | 9 | 10 | 0 | 0 | 0 |
| **OTU0813** | 0 | 0 | 1 | 0 | 12 | 0 | 1 | 0 | 0 |
| **OTU0814** | 12 | 5 | 4 | 0 | 5 | 0 | 0 | 0 | 0 |
| **OTU0815** | 1 | 1 | 0 | 0 | 0 | 0 | 0 | 0 | 14 |
| **OTU0816** | 1 | 10 | 3 | 0 | 0 | 0 | 0 | 0 | 0 |
| **OTU0817** | 0 | 0 | 0 | 1 | 2 | 2 | 4 | 1 | 1 |
| **OTU0818** | 5 | 6 | 5 | 0 | 2 | 0 | 0 | 0 | 0 |
| **OTU0819** | 0 | 0 | 1 | 15 | 6 | 5 | 11 | 2 | 3 |
| **OTU0820** | 1 | 1 | 2 | 17 | 7 | 13 | 17 | 6 | 4 |
| **OTU0821** | 1 | 1 | 1 | 7 | 3 | 7 | 8 | 4 | 3 |
| **OTU0822** | 3 | 0 | 0 | 4 | 4 | 0 | 6 | 1 | 0 |
| **OTU0823** | 6 | 9 | 15 | 1 | 1 | 1 | 0 | 0 | 0 |
| **OTU0824** | 7 | 7 | 6 | 0 | 1 | 0 | 0 | 0 | 0 |
| **OTU0825** | 0 | 0 | 1 | 12 | 2 | 0 | 1 | 0 | 0 |
| **OTU0826** | 1 | 0 | 1 | 0 | 0 | 16 | 0 | 0 | 0 |
| **OTU0827** | 15 | 7 | 12 | 166 | 102 | 86 | 169 | 75 | 89 |
| **OTU0828** | 0 | 0 | 2 | 0 | 0 | 12 | 0 | 0 | 0 |
| **OTU0829** | 2 | 1 | 0 | 0 | 9 | 0 | 0 | 0 | 0 |
| **OTU0830** | 41 | 40 | 66 | 2 | 11 | 24 | 0 | 0 | 3 |
| **OTU0831** | 19 | 22 | 38 | 0 | 11 | 13 | 0 | 0 | 0 |
| **OTU0832** | 0 | 0 | 0 | 0 | 0 | 16 | 0 | 0 | 0 |
| **OTU0833** | 0 | 0 | 0 | 0 | 0 | 0 | 0 | 0 | 12 |
| **OTU0834** | 22 | 13 | 27 | 2 | 8 | 2 | 0 | 0 | 1 |
| **OTU0835** | 15 | 11 | 17 | 0 | 0 | 2 | 0 | 0 | 0 |
| **OTU0836** | 0 | 2 | 4 | 8 | 0 | 0 | 0 | 1 | 3 |
| **OTU0837** | 0 | 0 | 0 | 1 | 1 | 0 | 1 | 2 | 1 |
| **OTU0838** | 7 | 6 | 11 | 0 | 3 | 2 | 0 | 0 | 0 |
| **OTU0839** | 57 | 68 | 89 | 0 | 10 | 14 | 0 | 0 | 0 |
| **OTU0840** | 0 | 0 | 0 | 0 | 0 | 1 | 0 | 0 | 11 |
| **OTU0841** | 17 | 16 | 31 | 0 | 11 | 10 | 0 | 0 | 0 |
| **OTU0842** | 0 | 0 | 0 | 0 | 1 | 0 | 0 | 11 | 0 |
| **OTU0843** | 7 | 10 | 8 | 0 | 0 | 15 | 0 | 0 | 0 |
| **OTU0844** | 0 | 1 | 12 | 0 | 0 | 0 | 0 | 0 | 0 |
| **OTU0845** | 4 | 14 | 17 | 0 | 0 | 8 | 0 | 0 | 0 |
| **OTU0846** | 1 | 0 | 0 | 25 | 10 | 8 | 11 | 12 | 6 |
| **OTU0847** | 47 | 46 | 68 | 1657 | 732 | 601 | 1239 | 721 | 790 |
| **OTU0848** | 0 | 0 | 1 | 14 | 3 | 5 | 7 | 2 | 3 |
| **OTU0849** | 10 | 18 | 21 | 0 | 4 | 3 | 0 | 0 | 0 |
| **OTU0850** | 5 | 3 | 12 | 0 | 0 | 0 | 0 | 0 | 0 |
| **OTU0851** | 30 | 50 | 84 | 0 | 13 | 12 | 0 | 0 | 0 |
| **OTU0852** | 31114 | 21268 | 39514 | 270 | 3828 | 4074 | 217 | 192 | 145 |
| **OTU0853** | 1 | 0 | 3 | 13 | 7 | 0 | 1 | 0 | 1 |
| **OTU0854** | 0 | 0 | 0 | 2 | 0 | 1 | 0 | 0 | 0 |
| **OTU0855** | 0 | 0 | 0 | 2 | 1 | 5 | 0 | 1 | 4 |
| **OTU0856** | 7 | 11 | 11 | 0 | 0 | 0 | 0 | 0 | 0 |
| **OTU0857** | 1 | 0 | 0 | 14 | 3 | 4 | 1 | 5 | 4 |
| **OTU0858** | 34 | 20 | 43 | 1 | 3 | 4 | 0 | 0 | 0 |
| **OTU0859** | 20 | 13 | 53 | 0 | 3 | 4 | 0 | 1 | 0 |
| **OTU0860** | 0 | 4 | 9 | 0 | 0 | 0 | 0 | 0 | 0 |
| **OTU0861** | 11 | 18 | 11 | 0 | 0 | 0 | 0 | 1 | 0 |
| **OTU0862** | 19 | 15 | 31 | 0 | 7 | 7 | 0 | 0 | 1 |
| **OTU0863** | 7 | 7 | 17 | 317 | 188 | 162 | 192 | 196 | 142 |
| **OTU0864** | 0 | 0 | 0 | 1 | 0 | 0 | 0 | 0 | 12 |
| **OTU0865** | 1 | 0 | 2 | 0 | 9 | 0 | 0 | 0 | 0 |
| **OTU0866** | 36 | 38 | 105 | 0 | 29 | 50 | 0 | 1 | 0 |
| **OTU0867** | 4 | 1 | 4 | 0 | 2 | 1 | 0 | 0 | 0 |
| **OTU0868** | 17 | 17 | 21 | 1 | 1 | 0 | 0 | 0 | 1 |
| **OTU0869** | 0 | 0 | 0 | 10 | 0 | 0 | 0 | 0 | 1 |
| **OTU0870** | 0 | 0 | 0 | 13 | 0 | 0 | 0 | 0 | 0 |
| **OTU0871** | 5 | 1 | 5 | 212 | 57 | 41 | 85 | 55 | 49 |
| **OTU0872** | 13 | 7 | 17 | 0 | 2 | 2 | 0 | 0 | 0 |
| **OTU0873** | 4 | 7 | 7 | 18 | 15 | 1 | 8 | 5 | 11 |
| **OTU0874** | 6 | 7 | 0 | 0 | 0 | 0 | 0 | 0 | 0 |
| **OTU0875** | 0 | 0 | 0 | 0 | 0 | 0 | 0 | 0 | 16 |
| **OTU0876** | 0 | 0 | 1 | 1 | 0 | 2 | 1 | 0 | 0 |
| **OTU0877** | 1 | 1 | 3 | 0 | 0 | 9 | 0 | 0 | 0 |
| **OTU0878** | 0 | 0 | 2 | 0 | 0 | 5 | 8 | 0 | 1 |
| **OTU0879** | 318 | 408 | 554 | 9 | 13 | 19 | 6 | 2 | 6 |
| **OTU0880** | 2 | 5 | 3 | 0 | 0 | 0 | 1 | 0 | 0 |
| **OTU0881** | 0 | 0 | 0 | 10 | 0 | 1 | 0 | 0 | 1 |
| **OTU0882** | 0 | 0 | 0 | 0 | 0 | 15 | 0 | 0 | 0 |
| **OTU0883** | 0 | 0 | 0 | 1 | 0 | 0 | 0 | 0 | 13 |
| **OTU0884** | 7 | 15 | 25 | 0 | 11 | 5 | 0 | 0 | 0 |
| **OTU0885** | 0 | 0 | 0 | 14 | 0 | 0 | 0 | 0 | 6 |
| **OTU0886** | 6 | 7 | 13 | 0 | 8 | 6 | 0 | 0 | 1 |
| **OTU0887** | 9 | 6 | 15 | 0 | 0 | 4 | 0 | 0 | 0 |
| **OTU0888** | 0 | 0 | 0 | 1 | 0 | 0 | 10 | 0 | 0 |
| **OTU0889** | 0 | 0 | 1 | 13 | 0 | 0 | 0 | 0 | 0 |
| **OTU0890** | 0 | 2 | 1 | 51 | 23 | 14 | 16 | 13 | 12 |
| **OTU0891** | 1 | 0 | 0 | 0 | 12 | 0 | 0 | 0 | 0 |
| **OTU0892** | 0 | 0 | 0 | 12 | 0 | 0 | 0 | 0 | 0 |
| **OTU0893** | 101 | 109 | 232 | 1 | 24 | 34 | 0 | 0 | 0 |
| **OTU0894** | 0 | 0 | 0 | 12 | 0 | 0 | 0 | 0 | 0 |
| **OTU0895** | 0 | 0 | 0 | 18 | 2 | 5 | 15 | 11 | 4 |
| **OTU0896** | 2 | 10 | 6 | 0 | 6 | 6 | 0 | 0 | 0 |
| **OTU0897** | 41 | 80 | 86 | 0 | 10 | 14 | 0 | 0 | 0 |
| **OTU0898** | 2 | 1 | 1 | 41 | 21 | 13 | 14 | 19 | 16 |
| **OTU0899** | 66 | 58 | 108 | 0 | 19 | 25 | 0 | 0 | 0 |
| **OTU0900** | 39 | 42 | 84 | 0 | 22 | 12 | 1 | 0 | 0 |
| **OTU0901** | 3 | 1 | 0 | 55 | 24 | 14 | 27 | 11 | 15 |
| **OTU0902** | 1 | 0 | 0 | 30 | 4 | 2 | 4 | 3 | 5 |
| **OTU0903** | 2 | 4 | 8 | 0 | 2 | 1 | 0 | 0 | 0 |
| **OTU0904** | 7 | 8 | 22 | 0 | 8 | 6 | 0 | 0 | 0 |
| **OTU0905** | 8 | 1 | 10 | 0 | 0 | 0 | 0 | 0 | 0 |
| **OTU0906** | 0 | 1 | 1 | 1 | 0 | 2 | 0 | 0 | 0 |
| **OTU0907** | 0 | 0 | 0 | 0 | 0 | 2 | 0 | 1 | 2 |
| **OTU0908** | 2 | 2 | 1 | 9 | 7 | 25 | 2 | 1 | 4 |
| **OTU0909** | 0 | 0 | 0 | 0 | 1 | 1 | 0 | 0 | 1 |
| **OTU0910** | 0 | 0 | 0 | 0 | 1 | 0 | 0 | 1 | 1 |
| **OTU0911** | 1 | 1 | 0 | 0 | 12 | 0 | 0 | 0 | 0 |
| **OTU0912** | 0 | 0 | 1 | 3 | 1 | 2 | 1 | 3 | 2 |
| **OTU0913** | 3 | 6 | 12 | 44 | 24 | 16 | 29 | 13 | 12 |
| **OTU0914** | 3 | 1 | 1 | 0 | 1 | 1 | 0 | 0 | 0 |
| **OTU0915** | 1 | 0 | 0 | 0 | 0 | 12 | 0 | 0 | 16 |
| **OTU0916** | 4 | 5 | 1 | 0 | 2 | 4 | 0 | 0 | 0 |
| **OTU0917** | 1 | 3 | 7 | 0 | 0 | 0 | 0 | 0 | 1 |
| **OTU0918** | 0 | 0 | 1 | 0 | 9 | 0 | 0 | 0 | 0 |
| **OTU0919** | 9 | 1 | 7 | 2 | 0 | 0 | 0 | 0 | 0 |
| **OTU0920** | 0 | 0 | 0 | 13 | 0 | 0 | 0 | 0 | 0 |
| **OTU0921** | 5 | 3 | 3 | 15 | 1 | 116 | 0 | 25 | 18 |
| **OTU0922** | 0 | 0 | 0 | 0 | 0 | 14 | 0 | 0 | 0 |
| **OTU0923** | 0 | 1 | 2 | 0 | 1 | 0 | 0 | 0 | 0 |
| **OTU0924** | 12 | 0 | 2 | 1 | 0 | 0 | 0 | 0 | 0 |
| **OTU0925** | 4 | 1 | 3 | 52 | 15 | 6 | 16 | 18 | 5 |
| **OTU0926** | 0 | 0 | 0 | 1 | 0 | 0 | 9 | 0 | 0 |
| **OTU0927** | 11 | 1 | 0 | 0 | 0 | 14 | 0 | 0 | 0 |
| **OTU0928** | 9 | 3 | 3 | 0 | 1 | 4 | 0 | 0 | 0 |
| **OTU0929** | 0 | 0 | 0 | 14 | 0 | 0 | 0 | 0 | 0 |
| **OTU0930** | 48 | 93 | 83 | 0 | 7 | 13 | 0 | 1 | 0 |
| **OTU0931** | 0 | 0 | 0 | 0 | 0 | 11 | 1 | 0 | 0 |
| **OTU0932** | 0 | 0 | 0 | 0 | 10 | 0 | 0 | 0 | 0 |
| **OTU0933** | 0 | 0 | 0 | 0 | 9 | 0 | 0 | 0 | 0 |
| **OTU0934** | 0 | 1 | 0 | 11 | 8 | 8 | 7 | 1 | 3 |
| **OTU0935** | 0 | 0 | 0 | 21 | 0 | 0 | 0 | 5 | 0 |
| **OTU0936** | 0 | 0 | 0 | 0 | 11 | 0 | 0 | 0 | 0 |
| **OTU0937** | 57 | 57 | 114 | 0 | 19 | 27 | 0 | 0 | 0 |
| **OTU0938** | 1 | 1 | 0 | 0 | 1 | 8 | 0 | 0 | 0 |
| **OTU0939** | 65 | 81 | 112 | 1 | 33 | 36 | 0 | 0 | 0 |
| **OTU0940** | 1 | 1 | 1 | 0 | 0 | 0 | 0 | 0 | 0 |
| **OTU0941** | 0 | 0 | 0 | 3 | 3 | 4 | 1 | 0 | 1 |
| **OTU0942** | 0 | 0 | 0 | 0 | 0 | 0 | 0 | 11 | 0 |
| **OTU0943** | 1 | 0 | 1 | 2 | 8 | 3 | 5 | 4 | 7 |
| **OTU0944** | 6 | 15 | 27 | 1 | 8 | 4 | 0 | 0 | 0 |
| **OTU0945** | 1 | 0 | 4 | 59 | 19 | 18 | 21 | 15 | 12 |
| **OTU0946** | 0 | 0 | 4 | 28 | 14 | 15 | 20 | 7 | 7 |
| **OTU0947** | 5 | 1 | 9 | 0 | 3 | 3 | 0 | 0 | 0 |
| **OTU0948** | 173 | 164 | 380 | 9933 | 4495 | 4585 | 6839 | 3959 | 4480 |
| **OTU0949** | 1 | 0 | 2 | 0 | 0 | 7 | 0 | 0 | 0 |
| **OTU0950** | 0 | 0 | 0 | 0 | 8 | 0 | 1 | 0 | 0 |
| **OTU0951** | 4 | 6 | 9 | 0 | 0 | 0 | 0 | 0 | 0 |
| **OTU0952** | 0 | 0 | 0 | 9 | 0 | 0 | 0 | 0 | 0 |
| **OTU0953** | 0 | 0 | 0 | 0 | 0 | 8 | 1 | 0 | 0 |
| **OTU0954** | 0 | 0 | 0 | 0 | 0 | 0 | 0 | 10 | 0 |
| **OTU0955** | 3 | 4 | 7 | 8 | 0 | 1 | 0 | 0 | 0 |
| **OTU0956** | 10 | 2 | 1 | 1 | 0 | 0 | 0 | 0 | 0 |
| **OTU0957** | 0 | 0 | 0 | 0 | 11 | 0 | 0 | 0 | 0 |
| **OTU0958** | 1 | 0 | 0 | 4 | 2 | 1 | 4 | 4 | 0 |
| **OTU0959** | 1120 | 950 | 1936 | 4 | 568 | 517 | 3 | 5 | 7 |
| **OTU0960** | 14 | 4 | 21 | 388 | 262 | 173 | 216 | 126 | 123 |
| **OTU0961** | 59 | 54 | 99 | 0 | 19 | 14 | 0 | 0 | 1 |
| **OTU0962** | 2 | 4 | 15 | 0 | 1 | 0 | 0 | 0 | 0 |
| **OTU0963** | 2 | 8 | 5 | 0 | 0 | 0 | 1 | 0 | 0 |
| **OTU0964** | 11 | 10 | 21 | 1 | 0 | 1 | 2 | 0 | 0 |
| **OTU0965** | 8 | 5 | 4 | 1 | 0 | 0 | 0 | 0 | 0 |
| **OTU0966** | 0 | 0 | 0 | 0 | 0 | 0 | 0 | 15 | 0 |
| **OTU0967** | 0 | 0 | 0 | 0 | 0 | 11 | 0 | 0 | 0 |
| **OTU0968** | 23 | 18 | 25 | 3 | 18 | 6 | 0 | 1 | 1 |
| **OTU0969** | 7 | 13 | 10 | 1 | 1 | 0 | 0 | 0 | 0 |
| **OTU0970** | 0 | 0 | 1 | 8 | 6 | 2 | 7 | 9 | 4 |
| **OTU0971** | 0 | 0 | 1 | 20 | 5 | 6 | 13 | 13 | 6 |
| **OTU0972** | 0 | 0 | 0 | 0 | 0 | 0 | 0 | 0 | 9 |
| **OTU0973** | 0 | 1 | 10 | 0 | 0 | 0 | 0 | 0 | 0 |
| **OTU0974** | 4 | 6 | 1 | 0 | 0 | 0 | 0 | 0 | 0 |
| **OTU0975** | 12 | 17 | 28 | 0 | 3 | 2 | 0 | 1 | 1 |
| **OTU0976** | 1 | 1 | 1 | 13 | 4 | 5 | 6 | 5 | 10 |
| **OTU0977** | 3 | 0 | 1 | 0 | 0 | 0 | 34 | 0 | 0 |
| **OTU0978** | 0 | 6 | 5 | 0 | 0 | 0 | 0 | 0 | 0 |
| **OTU0979** | 0 | 1 | 2 | 5 | 3 | 11 | 7 | 9 | 7 |
| **OTU0980** | 8 | 4 | 11 | 0 | 0 | 0 | 0 | 0 | 0 |
| **OTU0981** | 10 | 4 | 0 | 0 | 0 | 0 | 0 | 0 | 0 |
| **OTU0982** | 0 | 1 | 2 | 8 | 0 | 0 | 0 | 0 | 0 |
| **OTU0983** | 2 | 1 | 5 | 43 | 11 | 13 | 20 | 17 | 8 |
| **OTU0984** | 2 | 1 | 5 | 108 | 61 | 68 | 73 | 46 | 60 |
| **OTU0985** | 60 | 86 | 117 | 0 | 14 | 19 | 0 | 0 | 0 |
| **OTU0986** | 22 | 20 | 51 | 1 | 0 | 7 | 2 | 0 | 11 |
| **OTU0987** | 0 | 0 | 1 | 11 | 2 | 4 | 7 | 2 | 2 |
| **OTU0988** | 5 | 7 | 17 | 1 | 1 | 1 | 0 | 0 | 0 |
| **OTU0989** | 6 | 2 | 3 | 10 | 15 | 22 | 7 | 17 | 27 |
| **OTU0990** | 7 | 4 | 7 | 138 | 51 | 113 | 169 | 40 | 34 |
| **OTU0991** | 17 | 18 | 26 | 0 | 5 | 4 | 0 | 0 | 0 |
| **OTU0992** | 0 | 0 | 0 | 16 | 1 | 0 | 2 | 0 | 0 |
| **OTU0993** | 0 | 0 | 0 | 10 | 0 | 0 | 0 | 0 | 0 |
| **OTU0994** | 1 | 3 | 6 | 65 | 32 | 32 | 41 | 29 | 30 |
| **OTU0995** | 0 | 0 | 2 | 20 | 6 | 4 | 4 | 2 | 1 |
| **OTU0996** | 4 | 3 | 9 | 329 | 158 | 143 | 167 | 136 | 127 |
| **OTU0997** | 0 | 2 | 11 | 1 | 0 | 0 | 0 | 0 | 1 |
| **OTU0998** | 0 | 0 | 3 | 31 | 8 | 5 | 25 | 7 | 7 |
| **OTU0999** | 0 | 3 | 6 | 1 | 0 | 0 | 0 | 0 | 0 |
| **OTU1000** | 2 | 10 | 3 | 0 | 0 | 0 | 0 | 0 | 0 |
| **OTU1001** | 0 | 0 | 0 | 4 | 6 | 4 | 7 | 6 | 5 |
| **OTU1002** | 22 | 16 | 35 | 3 | 12 | 0 | 0 | 1 | 1 |
| **OTU1003** | 4 | 0 | 5 | 0 | 1 | 1 | 0 | 1 | 0 |
| **OTU1004** | 461 | 335 | 875 | 5 | 323 | 231 | 1 | 1 | 2 |
| **OTU1005** | 1 | 2 | 1 | 0 | 1 | 0 | 0 | 0 | 0 |
| **OTU1006** | 1 | 5 | 8 | 1 | 2 | 0 | 0 | 1 | 0 |
| **OTU1007** | 55 | 42 | 93 | 0 | 21 | 16 | 0 | 1 | 0 |
| **OTU1008** | 58 | 35 | 90 | 2 | 40 | 42 | 2 | 0 | 0 |
| **OTU1009** | 1 | 0 | 0 | 0 | 12 | 0 | 0 | 0 | 0 |
| **OTU1010** | 0 | 0 | 0 | 0 | 0 | 0 | 0 | 0 | 11 |
| **OTU1011** | 62 | 75 | 117 | 0 | 20 | 9 | 1 | 0 | 1 |
| **OTU1012** | 7 | 12 | 16 | 0 | 5 | 2 | 0 | 0 | 0 |
| **OTU1013** | 2 | 4 | 10 | 0 | 1 | 1 | 0 | 0 | 0 |
| **OTU1014** | 0 | 0 | 0 | 3 | 2 | 2 | 1 | 1 | 1 |
| **OTU1015** | 9 | 6 | 11 | 0 | 2 | 1 | 0 | 0 | 0 |
| **OTU1016** | 3 | 4 | 11 | 0 | 5 | 6 | 0 | 0 | 0 |
| **OTU1017** | 0 | 0 | 0 | 4 | 0 | 0 | 1 | 1 | 0 |
| **OTU1018** | 2 | 1 | 6 | 0 | 0 | 0 | 0 | 0 | 0 |
| **OTU1019** | 7 | 9 | 10 | 0 | 1 | 4 | 0 | 0 | 0 |
| **OTU1020** | 5 | 6 | 18 | 0 | 2 | 1 | 0 | 0 | 0 |
| **OTU1021** | 14 | 12 | 24 | 0 | 7 | 5 | 0 | 0 | 0 |
| **OTU1022** | 1 | 0 | 0 | 3 | 0 | 3 | 2 | 1 | 1 |
| **OTU1023** | 1 | 1 | 1 | 0 | 1 | 0 | 0 | 0 | 0 |
| **OTU1024** | 0 | 0 | 6 | 0 | 4 | 0 | 0 | 1 | 0 |
| **OTU1025** | 0 | 1 | 0 | 33 | 12 | 10 | 14 | 12 | 6 |
| **OTU1026** | 11 | 11 | 32 | 0 | 9 | 6 | 0 | 0 | 0 |
| **OTU1027** | 724 | 551 | 1336 | 6 | 464 | 402 | 0 | 1 | 0 |
| **OTU1028** | 13 | 21 | 63 | 2 | 0 | 0 | 1 |  |  |
| **OTU1029** | 0 | 0 | 11 | 0 | 0 | 0 | 0 | 0 | 1 |
| **OTU1030** | 1 | 1 | 2 | 72 | 16 | 16 | 32 | 11 | 12 |
| **OTU1031** | 3 | 1 | 0 | 31 | 11 | 11 | 15 | 9 | 11 |
| **OTU1032** | 0 | 0 | 0 | 1 | 0 | 8 | 0 | 0 | 0 |
| **OTU1033** | 0 | 0 | 0 | 1 | 1 | 1 | 0 | 0 | 0 |
| **OTU1034** | 6 | 1 | 2 | 0 | 0 | 1 | 0 | 0 | 0 |
| **OTU1035** | 13 | 25 | 18 | 1 | 1 | 3 | 0 | 0 | 0 |
| **OTU1036** | 3 | 1 | 5 | 0 | 0 | 1 | 0 | 0 | 0 |
| **OTU1037** | 2 | 7 | 14 | 0 | 0 | 1 | 0 | 0 | 0 |
| **OTU1038** | 26 | 29 | 41 | 2 | 0 | 1 | 1 | 1 | 1 |
| **OTU1039** | 166 | 146 | 320 | 3 | 227 | 271 | 0 | 1 | 1 |
| **OTU1040** | 0 | 0 | 0 | 1 | 6 | 12 | 2 | 4 | 5 |
| **OTU1041** | 0 | 0 | 0 | 0 | 0 | 19 | 0 | 0 | 0 |
| **OTU1042** | 18 | 26 | 29 | 0 | 8 | 12 | 0 | 0 | 0 |
| **OTU1043** | 1 | 1 | 2 | 6 | 1 | 1 | 1 | 2 | 0 |
| **OTU1044** | 0 | 0 | 0 | 32 | 8 | 5 | 7 | 3 | 6 |
| **OTU1045** | 47 | 68 | 90 | 0 | 21 | 21 | 0 | 0 | 0 |
| **OTU1046** | 2 | 2 | 1 | 34 | 27 | 20 | 21 | 27 | 18 |
| **OTU1047** | 36 | 50 | 106 | 0 | 11 | 19 | 0 | 0 | 0 |
| **OTU1048** | 216 | 206 | 343 | 2 | 2 | 10 | 1 | 1 | 2 |
| **OTU1049** | 0 | 1 | 0 | 2 | 8 | 2 | 0 | 0 | 1 |
| **OTU1050** | 1 | 1 | 3 | 3 | 4 | 0 | 4 | 0 | 1 |
| **OTU1051** | 2 | 3 | 5 | 152 | 65 | 36 | 84 | 64 | 34 |
| **OTU1052** | 0 | 1 | 1 | 0 | 0 | 7 | 0 | 0 | 0 |
| **OTU1053** | 4 | 6 | 7 | 0 | 0 | 0 | 0 | 0 | 0 |
| **OTU1054** | 9 | 10 | 5 | 0 | 5 | 4 | 0 | 0 | 0 |
| **OTU1055** | 1 | 4 | 8 | 26 | 45 | 65 | 46 | 71 | 65 |
| **OTU1056** | 0 | 0 | 0 | 0 | 11 | 0 | 0 | 0 | 0 |
| **OTU1057** | 0 | 1 | 0 | 16 | 8 | 14 | 16 | 4 | 6 |
| **OTU1058** | 222 | 271 | 657 | 4 | 58 | 34 | 2 | 1 | 0 |
| **OTU1059** | 4 | 0 | 6 | 0 | 0 | 0 | 0 | 0 | 0 |
| **OTU1060** | 1 | 0 | 0 | 0 | 0 | 1 | 0 | 0 | 0 |
| **OTU1061** | 3 | 3 | 7 | 0 | 0 | 0 | 0 | 0 | 0 |
| **OTU1062** | 1 | 0 | 0 | 1 | 1 | 7 | 0 | 0 | 0 |
| **OTU1063** | 0 | 4 | 0 | 14 | 2 | 4 | 16 | 6 | 10 |
| **OTU1064** | 401 | 291 | 684 | 4 | 187 | 162 | 1 | 2 | 2 |
| **OTU1065** | 24 | 19 | 29 | 0 | 8 | 10 | 0 | 0 | 0 |
| **OTU1066** | 0 | 0 | 0 | 0 | 1 | 14 | 0 | 0 | 0 |
| **OTU1067** | 0 | 0 | 10 | 0 | 1 | 0 | 0 | 13 | 0 |
| **OTU1068** | 0 | 3 | 4 | 0 | 1 | 0 | 0 | 0 | 1 |
| **OTU1069** | 2 | 4 | 6 | 0 | 0 | 0 | 0 | 0 | 0 |
| **OTU1070** | 1 | 3 | 7 | 0 | 0 | 0 | 0 | 0 | 0 |
| **OTU1071** | 1 | 1 | 0 | 9 | 0 | 0 | 0 | 0 | 0 |
| **OTU1072** | 0 | 0 | 0 | 1 | 0 | 1 | 1 | 0 | 0 |
| **OTU1073** | 13 | 12 | 28 | 1 | 23 | 10 | 0 | 0 | 1 |
| **OTU1074** | 0 | 0 | 0 | 22 | 3 | 2 | 3 | 3 | 5 |
| **OTU1075** | 0 | 2 | 0 | 0 | 10 | 0 | 1 | 1 | 1 |
| **OTU1076** | 5 | 5 | 4 | 4 | 0 | 0 | 5 | 0 | 0 |
| **OTU1077** | 6 | 1 | 6 | 0 | 14 | 0 | 0 | 0 | 1 |
| **OTU1078** | 8 | 4 | 4 | 0 | 2 | 1 | 0 | 0 | 0 |
| **OTU1079** | 4 | 0 | 0 | 0 | 0 | 0 | 0 | 0 | 6 |
| **OTU1080** | 2 | 2 | 4 | 0 | 0 | 0 | 0 | 0 | 0 |
| **OTU1081** | 258 | 171 | 453 | 1 | 142 | 88 | 0 | 1 | 0 |
| **OTU1082** | 7 | 0 | 2 | 0 | 0 | 0 | 0 | 0 | 0 |
| **OTU1083** | 62 | 68 | 100 | 0 | 12 | 13 | 0 | 0 | 0 |
| **OTU1084** | 0 | 0 | 0 | 10 | 0 | 1 | 0 | 0 | 0 |
| **OTU1085** | 5 | 3 | 3 | 0 | 2 | 5 | 0 | 0 | 0 |
| **OTU1086** | 19 | 20 | 38 | 0 | 10 | 9 | 0 | 0 | 0 |
| **OTU1087** | 0 | 1 | 0 | 7 | 5 | 9 | 7 | 4 | 4 |
| **OTU1088** | 0 | 0 | 1 | 0 | 0 | 1 | 0 | 0 | 0 |
| **OTU1089** | 2 | 9 | 12 | 0 | 0 | 0 | 0 | 0 | 0 |
| **OTU1090** | 0 | 0 | 0 | 0 | 0 | 0 | 1 | 22 | 0 |
| **OTU1091** | 0 | 5 | 4 | 0 | 0 | 0 | 0 | 0 | 0 |
| **OTU1092** | 237 | 322 | 402 | 12 | 16 | 10 | 3 | 3 | 3 |
| **OTU1093** | 2 | 0 | 1 | 26 | 21 | 12 | 20 | 15 | 9 |
| **OTU1094** | 1 | 0 | 0 | 0 | 7 | 0 | 1 | 0 | 0 |
| **OTU1095** | 309 | 363 | 494 | 0 | 62 | 65 | 0 | 1 | 0 |
| **OTU1096** | 0 | 0 | 0 | 1 | 0 | 8 | 1 | 0 | 0 |
| **OTU1097** | 67 | 88 | 278 | 1 | 3 | 24 | 2 | 1 | 2 |
| **OTU1098** | 3 | 2 | 4 | 2 | 0 | 1 | 1 | 0 | 0 |
| **OTU1099** | 2 | 1 | 0 | 1 | 2 | 1 | 1 | 5 | 0 |
| **OTU1100** | 2 | 4 | 3 | 101 | 38 | 35 | 65 | 27 | 22 |
| **OTU1101** | 0 | 0 | 0 | 0 | 0 | 12 | 0 | 0 | 0 |
| **OTU1102** | 0 | 0 | 0 | 0 | 0 | 2 | 1 | 1 | 1 |
| **OTU1103** | 1 | 2 | 2 | 0 | 0 | 1 | 0 | 1 | 0 |
| **OTU1104** | 9 | 20 | 23 | 1 | 2 | 2 | 0 | 0 | 1 |
| **OTU1105** | 0 | 1 | 10 | 0 | 11 | 7 | 1 | 4 | 2 |
| **OTU1106** | 0 | 1 | 1 | 10 | 1 | 0 | 0 | 29 | 18 |
| **OTU1107** | 8 | 12 | 15 | 1 | 0 | 0 | 0 | 0 | 2 |
| **OTU1108** | 0 | 0 | 0 | 0 | 0 | 10 | 0 | 0 | 0 |
| **OTU1109** | 1 | 0 | 0 | 0 | 0 | 1 | 1 | 1 | 0 |
| **OTU1110** | 4 | 2 | 9 | 0 | 0 | 0 | 0 | 1 | 0 |
| **OTU1111** | 1 | 1 | 1 | 0 | 0 | 0 | 0 | 0 | 0 |
| **OTU1112** | 13 | 10 | 28 | 1 | 0 | 1 | 0 | 0 | 0 |
| **OTU1113** | 0 | 0 | 0 | 13 | 0 | 0 | 0 | 0 | 0 |
| **OTU1114** | 5 | 5 | 16 | 0 | 4 | 2 | 0 | 0 | 0 |
| **OTU1115** | 11 | 9 | 15 | 0 | 6 | 7 | 0 | 0 | 0 |
| **OTU1116** | 2 | 0 | 0 | 6 | 1 | 1 | 5 | 3 | 0 |
| **OTU1117** | 23 | 8 | 17 | 112 | 25 | 140 | 278 | 129 | 75 |
| **OTU1118** | 15 | 9 | 20 | 111 | 55 | 58 | 52 | 56 | 41 |
| **OTU1119** | 378 | 459 | 641 | 0 | 73 | 74 | 2 | 1 | 2 |
| **OTU1120** | 2 | 1 | 1 | 17 | 6 | 8 | 3 | 5 | 4 |
| **OTU1121** | 3 | 3 | 4 | 0 | 3 | 1 | 0 | 0 | 0 |
| **OTU1122** | 0 | 0 | 0 | 0 | 1 | 0 | 1 | 0 | 0 |
| **OTU1123** | 5 | 9 | 6 | 6 | 4 | 3 | 7 | 1 | 0 |
| **OTU1124** | 1 | 0 | 9 | 0 | 1 | 1 | 0 | 0 | 0 |
| **OTU1125** | 578 | 431 | 713 | 9 | 99 | 127 | 5 | 3 | 3 |
| **OTU1126** | 3 | 1 | 4 | 240 | 70 | 60 | 108 | 69 | 79 |
| **OTU1127** | 74 | 63 | 99 | 0 | 22 | 11 | 0 | 0 | 0 |
| **OTU1128** | 404 | 488 | 650 | 0 | 83 | 86 | 0 | 2 | 0 |
| **OTU1129** | 0 | 0 | 2 | 27 | 8 | 15 | 13 | 12 | 10 |
| **OTU1130** | 106 | 59 | 115 | 0 | 9 | 4 | 1 | 1 | 0 |
| **OTU1131** | 0 | 0 | 3 | 5 | 13 | 11 | 12 | 11 | 28 |
| **OTU1132** | 6 | 6 | 10 | 193 | 122 | 97 | 99 | 103 | 129 |
| **OTU1133** | 3 | 2 | 3 | 0 | 0 | 0 | 0 | 0 | 0 |
| **OTU1134** | 3 | 4 | 3 | 0 | 0 | 0 | 0 | 0 | 0 |
| **OTU1135** | 0 | 0 | 0 | 0 | 0 | 0 | 0 | 8 | 0 |
| **OTU1136** | 0 | 0 | 0 | 7 | 0 | 0 | 0 | 0 | 0 |
| **OTU1137** | 3 | 1 | 4 | 0 | 0 | 0 | 0 | 0 | 0 |
| **OTU1138** | 428 | 433 | 680 | 3 | 93 | 86 | 4 | 0 | 1 |
| **OTU1139** | 38 | 31 | 29 | 6 | 2 | 2 | 1 | 0 | 0 |
| **OTU1140** | 6 | 1 | 0 | 0 | 0 | 0 | 0 | 0 | 0 |
| **OTU1141** | 13 | 20 | 24 | 0 | 0 | 0 | 1 | 0 | 0 |
| **OTU1142** | 0 | 0 | 0 | 16 | 3 | 5 | 14 | 6 | 5 |
| **OTU1143** | 114 | 105 | 223 | 3 | 6 | 3 | 1 | 0 | 0 |
| **OTU1144** | 27 | 39 | 54 | 0 | 4 | 5 | 0 | 0 | 0 |
| **OTU1145** | 0 | 0 | 0 | 33 | 16 | 11 | 11 | 10 | 5 |
| **OTU1146** | 15 | 24 | 26 | 0 | 4 | 7 | 0 | 0 | 0 |
| **OTU1147** | 14 | 21 | 26 | 0 | 1 | 2 | 0 | 0 | 0 |
| **OTU1148** | 0 | 0 | 0 | 0 | 0 | 2 | 1 | 0 | 1 |
| **OTU1149** | 1 | 0 | 1 | 5 | 3 | 0 | 0 | 0 | 5 |
| **OTU1150** | 7 | 0 | 0 | 0 | 0 | 0 | 0 | 0 | 0 |
| **OTU1151** | 0 | 0 | 0 | 4 | 0 | 0 | 0 | 0 | 8 |
| **OTU1152** | 0 | 0 | 0 | 0 | 7 | 0 | 1 | 0 | 0 |
| **OTU1153** | 20 | 9 | 34 | 0 | 17 | 15 | 0 | 0 | 0 |
| **OTU1154** | 0 | 2 | 11 | 0 | 0 | 0 | 0 | 0 | 0 |
| **OTU1155** | 8 | 3 | 17 | 121 | 123 | 125 | 116 | 144 | 134 |
| **OTU1156** | 0 | 5 | 4 | 0 | 0 | 0 | 0 | 0 | 0 |
| **OTU1157** | 0 | 0 | 0 | 0 | 0 | 1 | 0 | 1 | 1 |
| **OTU1158** | 0 | 0 | 8 | 0 | 0 | 0 | 0 | 0 | 0 |
| **OTU1159** | 27 | 23 | 47 | 1 | 1 | 4 | 9 | 0 | 2 |
| **OTU1160** | 18 | 23 | 24 | 0 | 2 | 1 | 0 | 0 | 0 |
| **OTU1161** | 35 | 18 | 51 | 2 | 10 | 9 | 0 | 0 | 0 |
| **OTU1162** | 10 | 7 | 27 | 382 | 246 | 202 | 412 | 201 | 193 |
| **OTU1163** | 44 | 20 | 30 | 1 | 1 | 0 | 1 | 0 | 0 |
| **OTU1164** | 0 | 0 | 9 | 0 | 0 | 0 | 0 | 0 | 0 |
| **OTU1165** | 1 | 1 | 2 | 1 | 11 | 13 | 3 | 10 | 13 |
| **OTU1166** | 9 | 8 | 10 | 0 | 5 | 5 | 0 | 0 | 0 |
| **OTU1167** | 14 | 10 | 17 | 1 | 7 | 11 | 0 | 0 | 0 |
| **OTU1168** | 4 | 1 | 2 | 95 | 44 | 35 | 75 | 21 | 37 |
| **OTU1169** | 7 | 5 | 12 | 0 | 7 | 5 | 0 | 0 | 0 |
| **OTU1170** | 118 | 125 | 185 | 0 | 19 | 36 | 0 | 1 | 1 |
| **OTU1171** | 18 | 22 | 43 | 0 | 26 | 25 | 0 | 0 | 1 |
| **OTU1172** | 10 | 4 | 9 | 0 | 0 | 2 | 0 | 0 | 0 |
| **OTU1173** | 292 | 214 | 507 | 0 | 163 | 170 | 3 | 0 | 1 |
| **OTU1174** | 696 | 528 | 1144 | 4 | 273 | 293 | 1 | 5 | 4 |
| **OTU1175** | 104 | 51 | 169 | 4 | 6 | 10 | 2 | 0 | 1 |
| **OTU1176** | 1 | 0 | 1 | 10 | 10 | 7 | 11 | 4 | 12 |
| **OTU1177** | 0 | 0 | 1 | 31 | 15 | 24 | 30 | 32 | 15 |
| **OTU1178** | 309 | 233 | 541 | 0 | 372 | 302 | 1 | 0 | 0 |
| **OTU1179** | 2 | 1 | 2 | 10 | 0 | 1 | 0 | 8 | 14 |
| **OTU1180** | 8 | 2 | 10 | 321 | 107 | 138 | 205 | 134 | 123 |
| **OTU1181** | 0 | 1 | 1 | 5 | 0 | 1 | 0 | 0 | 0 |
| **OTU1182** | 0 | 0 | 0 | 0 | 6 | 0 | 0 | 1 | 0 |
| **OTU1183** | 5 | 2 | 5 | 42 | 26 | 18 | 26 | 21 | 14 |
| **OTU1184** | 0 | 0 | 3 | 25 | 13 | 6 | 14 | 13 | 5 |
| **OTU1185** | 143 | 210 | 222 | 4 | 20 | 17 | 3 | 1 | 1 |
| **OTU1186** | 0 | 0 | 0 | 4 | 4 | 5 | 0 | 0 | 0 |
| **OTU1187** | 0 | 0 | 0 | 2 | 0 | 1 | 0 | 7 | 2 |
| **OTU1188** | 1 | 1 | 6 | 96 | 55 | 49 | 1 | 30 | 38 |
| **OTU1189** | 27 | 23 | 72 | 0 | 8 | 19 | 0 | 0 | 0 |
| **OTU1190** | 1 | 2 | 6 | 0 | 0 | 0 | 0 | 0 | 0 |
| **OTU1191** | 0 | 3 | 6 | 0 | 0 | 0 | 0 | 0 | 0 |
| **OTU1192** | 2 | 10 | 8 | 0 | 3 | 0 | 0 | 0 | 1 |
| **OTU1193** | 0 | 1 | 0 | 0 | 0 | 0 | 0 | 10 | 0 |
| **OTU1194** | 0 | 0 | 1 | 0 | 5 | 0 | 0 | 1 | 0 |
| **OTU1195** | 81 | 63 | 106 | 2461 | 983 | 1031 | 1889 | 984 | 1229 |
| **OTU1196** | 0 | 0 | 2 | 19 | 6 | 2 | 9 | 2 | 1 |
| **OTU1197** | 7 | 15 | 24 | 0 | 0 | 0 | 0 | 0 | 0 |
| **OTU1198** | 13 | 17 | 29 | 0 | 7 | 2 | 0 | 0 | 0 |
| **OTU1199** | 0 | 2 | 5 | 0 | 0 | 0 | 0 | 0 | 0 |
| **OTU1200** | 0 | 2 | 2 | 0 | 0 | 1 | 0 | 0 | 0 |
| **OTU1201** | 0 | 0 | 0 | 0 | 0 | 19 | 0 | 0 | 0 |
| **OTU1202** | 17 | 21 | 35 | 0 | 3 | 2 | 0 | 0 | 0 |
| **OTU1203** | 5 | 1 | 1 | 0 | 0 | 0 | 0 | 0 | 0 |
| **OTU1204** | 0 | 8 | 7 | 0 | 0 | 1 | 0 | 0 | 0 |
| **OTU1205** | 9 | 11 | 11 | 129 | 38 | 44 | 39 | 33 | 24 |
| **OTU1206** | 7 | 4 | 15 | 0 | 4 | 6 | 0 | 0 | 0 |
| **OTU1207** | 3 | 0 | 7 | 0 | 2 | 0 | 0 | 0 | 0 |
| **OTU1208** | 1 | 0 | 2 | 13 | 1 | 2 | 9 | 4 | 1 |
| **OTU1209** | 0 | 0 | 0 | 5 | 1 | 2 | 3 | 3 | 1 |
| **OTU1210** | 519 | 628 | 822 | 3 | 99 | 122 | 1 | 2 | 0 |
| **OTU1211** | 0 | 0 | 9 | 0 | 0 | 0 | 0 | 0 | 0 |
| **OTU1212** | 0 | 0 | 3 | 92 | 22 | 20 | 33 | 29 | 30 |
| **OTU1213** | 7 | 0 | 0 | 0 | 0 | 0 | 0 | 0 | 0 |
| **OTU1214** | 1 | 1 | 0 | 5 | 7 | 9 | 1 | 0 | 0 |
| **OTU1215** | 3 | 1 | 2 | 48 | 18 | 22 | 29 | 22 | 25 |
| **OTU1216** | 61 | 54 | 139 | 4202 | 2469 | 2182 | 2775 | 1707 | 1441 |
| **OTU1217** | 295 | 212 | 475 | 1 | 344 | 221 | 0 | 0 | 1 |
| **OTU1218** | 6 | 5 | 8 | 10 | 6 | 4 | 10 | 2 | 4 |
| **OTU1219** | 26 | 8 | 3 | 2 | 11 | 32 | 1 | 8 | 7 |
| **OTU1220** | 373 | 396 | 610 | 1 | 70 | 48 | 1 | 0 | 1 |
| **OTU1221** | 25 | 31 | 51 | 0 | 2 | 5 | 0 | 0 | 0 |
| **OTU1222** | 15 | 13 | 33 | 0 | 3 | 1 | 0 | 0 | 0 |
| **OTU1223** | 11 | 9 | 47 | 1 | 3 | 0 | 0 | 0 | 0 |
| **OTU1224** | 0 | 0 | 0 | 13 | 12 | 6 | 6 | 2 | 3 |
| **OTU1225** | 175 | 114 | 208 | 0 | 64 | 57 | 0 | 0 | 1 |
| **OTU1226** | 26 | 46 | 51 | 0 | 11 | 7 | 0 | 0 | 0 |
| **OTU1227** | 15 | 21 | 21 | 0 | 2 | 5 | 0 | 0 | 0 |
| **OTU1228** | 0 | 1 | 9 | 0 | 0 | 0 | 0 | 0 | 0 |
| **OTU1229** | 9 | 6 | 13 | 0 | 2 | 0 | 0 | 0 | 0 |
| **OTU1230** | 7 | 0 | 0 | 0 | 1 | 0 | 0 | 0 | 0 |
| **OTU1231** | 7 | 1 | 0 | 0 | 0 | 0 | 0 | 0 | 0 |
| **OTU1232** | 0 | 0 | 0 | 7 | 4 | 5 | 7 | 3 | 2 |
| **OTU1233** | 0 | 2 | 0 | 0 | 4 | 0 | 0 | 0 | 0 |
| **OTU1234** | 1 | 1 | 0 | 6 | 1 | 2 | 3 | 1 | 0 |
| **OTU1235** | 692 | 555 | 1031 | 6 | 143 | 273 | 7 | 10 | 5 |
| **OTU1236** | 0 | 0 | 0 | 2 | 2 | 3 | 5 | 1 | 0 |
| **OTU1237** | 33 | 27 | 48 | 0 | 0 | 2 | 0 | 0 | 1 |
| **OTU1238** | 1 | 0 | 0 | 1 | 0 | 1 | 2 | 0 | 1 |
| **OTU1239** | 2 | 0 | 1 | 26 | 12 | 13 | 27 | 11 | 12 |
| **OTU1240** | 28 | 25 | 61 | 0 | 13 | 6 | 0 | 0 | 0 |
| **OTU1241** | 38 | 21 | 68 | 653 | 346 | 378 | 671 | 374 | 356 |
| **OTU1242** | 6 | 0 | 5 | 247 | 58 | 62 | 112 | 72 | 66 |
| **OTU1243** | 0 | 1 | 0 | 0 | 0 | 12 | 0 | 0 | 0 |
| **OTU1244** | 3 | 2 | 13 | 0 | 1 | 0 | 0 | 0 | 0 |
| **OTU1245** | 0 | 0 | 7 | 0 | 0 | 0 | 0 | 0 | 0 |
| **OTU1246** | 1 | 1 | 1 | 17 | 6 | 5 | 8 | 6 | 2 |
| **OTU1247** | 7 | 1 | 1 | 23 | 8 | 0 | 0 | 0 | 17 |
| **OTU1248** | 133 | 81 | 153 | 1053 | 463 | 347 | 821 | 291 | 297 |
| **OTU1249** | 2 | 1 | 3 | 33 | 53 | 47 | 35 | 43 | 47 |
| **OTU1250** | 9 | 11 | 14 | 68 | 22 | 13 | 46 | 22 | 26 |
| **OTU1251** | 9 | 6 | 11 | 506 | 144 | 136 | 276 | 151 | 135 |
| **OTU1252** | 3 | 2 | 8 | 110 | 62 | 48 | 81 | 42 | 53 |
| **OTU1253** | 83 | 67 | 178 | 3664 | 2068 | 1625 | 2232 | 1392 | 1185 |
| **OTU1254** | 433 | 309 | 583 | 6 | 40 | 70 | 1 | 2 | 1 |
| **OTU1255** | 17 | 26 | 37 | 0 | 6 | 6 | 1 | 0 | 0 |
| **OTU1256** | 2 | 3 | 7 | 0 | 2 | 3 | 0 | 1 | 0 |
| **OTU1257** | 275 | 201 | 394 | 2 | 136 | 159 | 1 | 0 | 1 |
| **OTU1258** | 3 | 3 | 6 | 89 | 41 | 55 | 117 | 42 | 26 |
| **OTU1259** | 0 | 0 | 0 | 12 | 4 | 7 | 5 | 4 | 4 |
| **OTU1260** | 143 | 159 | 274 | 0 | 195 | 161 | 1 | 1 | 0 |
| **OTU1261** | 2 | 4 | 7 | 0 | 0 | 0 | 0 | 0 | 0 |
| **OTU1262** | 15 | 35 | 47 | 0 | 3 | 8 | 1 | 0 | 1 |
| **OTU1263** | 0 | 1 | 0 | 3 | 0 | 0 | 2 | 2 | 0 |
| **OTU1264** | 5 | 7 | 9 | 0 | 0 | 1 | 0 | 0 | 0 |
| **OTU1265** | 25 | 32 | 58 | 8 | 47 | 21 | 6 | 3 | 9 |
| **OTU1266** | 26 | 26 | 61 | 0 | 7 | 8 | 0 | 0 | 0 |
| **OTU1267** | 29 | 42 | 79 | 0 | 10 | 6 | 0 | 1 | 0 |
| **OTU1268** | 5 | 12 | 9 | 0 | 0 | 1 | 0 | 0 | 0 |
| **OTU1269** | 14 | 10 | 20 | 0 | 2 | 0 | 0 | 0 | 0 |
| **OTU1270** | 2 | 4 | 1 | 0 | 0 | 0 | 0 | 0 | 1 |
| **OTU1271** | 2 | 1 | 7 | 0 | 0 | 0 | 0 | 0 | 0 |
| **OTU1272** | 20 | 28 | 20 | 0 | 1 | 0 | 0 | 1 | 1 |
| **OTU1273** | 13 | 12 | 18 | 0 | 4 | 3 | 0 | 0 | 0 |
| **OTU1274** | 5 | 14 | 7 | 0 | 2 | 1 | 0 | 0 | 0 |
| **OTU1275** | 8 | 6 | 20 | 0 | 0 | 1 | 0 | 0 | 0 |
| **OTU1276** | 7 | 0 | 0 | 0 | 0 | 0 | 0 | 0 | 0 |
| **OTU1277** | 4 | 7 | 11 | 0 | 4 | 4 | 0 | 0 | 0 |
| **OTU1278** | 6 | 6 | 9 | 0 | 9 | 13 | 0 | 0 | 0 |
| **OTU1279** | 2 | 2 | 2 | 0 | 0 | 0 | 0 | 0 | 0 |
| **OTU1280** | 13 | 21 | 21 | 0 | 2 | 3 | 0 | 0 | 0 |
| **OTU1281** | 0 | 0 | 0 | 0 | 0 | 6 | 0 | 0 | 0 |
| **OTU1282** | 1 | 3 | 2 | 18 | 3 | 16 | 23 | 15 | 12 |
| **OTU1283** | 0 | 0 | 0 | 6 | 0 | 2 | 3 | 4 | 3 |
| **OTU1284** | 2 | 4 | 12 | 37 | 19 | 9 | 17 | 12 | 12 |
| **OTU1285** | 0 | 0 | 0 | 46 | 0 | 1 | 0 | 0 | 1 |
| **OTU1286** | 2 | 8 | 11 | 0 | 1 | 0 | 0 | 0 | 0 |
| **OTU1287** | 5 | 5 | 10 | 0 | 7 | 7 | 0 | 0 | 0 |
| **OTU1288** | 38 | 47 | 111 | 1 | 34 | 19 | 0 | 0 | 1 |
| **OTU1289** | 3 | 2 | 4 | 70 | 31 | 36 | 76 | 39 | 30 |
| **OTU1290** | 0 | 1 | 0 | 0 | 0 | 1 | 10 | 0 | 1 |
| **OTU1291** | 1 | 0 | 1 | 7 | 4 | 4 | 4 | 2 | 2 |
| **OTU1292** | 2 | 4 | 6 | 0 | 2 | 0 | 0 | 0 | 0 |
| **OTU1293** | 0 | 0 | 1 | 0 | 0 | 1 | 0 | 0 | 1 |
| **OTU1294** | 2 | 16 | 1 | 0 | 0 | 0 | 1 | 0 | 0 |
| **OTU1295** | 5 | 4 | 4 | 0 | 0 | 0 | 0 | 0 | 0 |
| **OTU1296** | 1 | 4 | 2 | 11 | 13 | 31 | 8 | 18 | 23 |
| **OTU1297** | 22 | 11 | 22 | 175 | 61 | 423 | 376 | 154 | 153 |
| **OTU1298** | 11 | 12 | 22 | 0 | 11 | 4 | 0 | 0 | 0 |
| **OTU1299** | 0 | 0 | 0 | 2 | 1 | 1 | 0 | 1 | 2 |
| **OTU1300** | 0 | 0 | 0 | 2 | 0 | 0 | 0 | 0 | 0 |
| **OTU1301** | 32 | 20 | 54 | 181 | 69 | 70 | 83 | 62 | 47 |
| **OTU1302** | 22 | 16 | 22 | 0 | 11 | 2 | 0 | 0 | 0 |
| **OTU1303** | 39 | 58 | 86 | 0 | 3 | 9 | 0 | 0 | 0 |
| **OTU1304** | 13 | 12 | 17 | 1 | 1 | 1 | 3 | 0 | 0 |
| **OTU1305** | 170 | 101 | 188 | 3 | 22 | 24 | 2 | 0 | 0 |
| **OTU1306** | 3 | 2 | 7 | 0 | 0 | 1 | 0 | 0 | 0 |
| **OTU1307** | 1 | 0 | 2 | 42 | 26 | 17 | 29 | 24 | 19 |
| **OTU1308** | 0 | 1 | 0 | 12 | 15 | 14 | 10 | 26 | 12 |
| **OTU1309** | 8 | 8 | 9 | 0 | 9 | 4 | 0 | 0 | 0 |
| **OTU1310** | 1 | 0 | 1 | 0 | 0 | 2 | 0 | 0 | 0 |
| **OTU1311** | 85 | 118 | 145 | 7 | 51 | 21 | 3 | 2 | 1 |
| **OTU1312** | 0 | 0 | 0 | 3 | 0 | 0 | 6 | 0 | 0 |
| **OTU1313** | 2 | 8 | 13 | 142 | 69 | 50 | 75 | 67 | 41 |
| **OTU1314** | 438 | 436 | 671 | 16 | 73 | 49 | 7 | 6 | 6 |
| **OTU1315** | 5 | 8 | 10 | 0 | 1 | 0 | 0 | 0 | 0 |
| **OTU1316** | 0 | 0 | 0 | 0 | 0 | 11 | 0 | 0 | 0 |
| **OTU1317** | 91 | 57 | 98 | 0 | 23 | 25 | 1 | 0 | 0 |
| **OTU1318** | 590 | 532 | 836 | 3 | 243 | 279 | 1 | 1 | 2 |
| **OTU1319** | 11 | 10 | 34 | 34 | 358 | 320 | 133 | 299 | 335 |
| **OTU1320** | 0 | 0 | 0 | 0 | 8 | 0 | 0 | 0 | 0 |
| **OTU1321** | 23 | 24 | 64 | 0 | 12 | 2 | 0 | 1 | 1 |
| **OTU1322** | 3 | 11 | 3 | 12 | 8 | 3 | 9 | 2 | 1 |
| **OTU1323** | 0 | 0 | 0 | 1 | 6 | 0 | 0 | 0 | 0 |
| **OTU1324** | 0 | 0 | 0 | 0 | 0 | 8 | 0 | 0 | 0 |
| **OTU1325** | 18 | 22 | 50 | 1 | 2 | 5 | 0 | 0 | 3 |
| **OTU1326** | 1 | 1 | 5 | 0 | 0 | 2 | 0 | 0 | 0 |
| **OTU1327** | 3 | 3 | 1 | 4 | 7 | 7 | 8 | 1 | 0 |
| **OTU1328** | 2 | 2 | 1 | 72 | 39 | 39 | 47 | 37 | 41 |
| **OTU1329** | 1 | 6 | 2 | 1 | 0 | 0 | 0 | 0 | 0 |
| **OTU1330** | 14 | 16 | 29 | 0 | 2 | 3 | 0 | 1 | 0 |
| **OTU1331** | 9 | 6 | 20 | 0 | 2 | 0 | 0 | 0 | 0 |
| **OTU1332** | 4202 | 4442 | 6544 | 79 | 540 | 307 | 41 | 61 | 27 |
| **OTU1333** | 1 | 0 | 1 | 4 | 1 | 2 | 5 | 2 | 1 |
| **OTU1334** | 56 | 40 | 103 | 0 | 99 | 53 | 0 | 0 | 0 |
| **OTU1335** | 0 | 0 | 0 | 0 | 8 | 0 | 0 | 0 | 0 |
| **OTU1336** | 6 | 7 | 2 | 0 | 0 | 0 | 0 | 0 | 0 |
| **OTU1337** | 1 | 3 | 2 | 1 | 0 | 0 | 0 | 0 | 0 |
| **OTU1338** | 149 | 124 | 256 | 1 | 8 | 8 | 0 | 1 | 2 |
| **OTU1339** | 2 | 1 | 0 | 28 | 8 | 15 | 10 | 10 | 7 |
| **OTU1340** | 151 | 186 | 311 | 2 | 23 | 23 | 4 | 2 | 1 |
| **OTU1341** | 5 | 4 | 2 | 0 | 0 | 2 | 0 | 0 | 0 |
| **OTU1342** | 0 | 0 | 1 | 15 | 13 | 11 | 13 | 8 | 9 |
| **OTU1343** | 1 | 1 | 5 | 0 | 0 | 0 | 0 | 0 | 0 |
| **OTU1344** | 1 | 2 | 0 | 21 | 9 | 2 | 7 | 3 | 12 |
| **OTU1345** | 361 | 366 | 487 | 9 | 34 | 20 | 10 | 3 | 1 |
| **OTU1346** | 13 | 8 | 18 | 0 | 4 | 3 | 0 | 0 | 0 |
| **OTU1347** | 0 | 0 | 0 | 0 | 0 | 2 | 15 | 0 | 0 |
| **OTU1348** | 23 | 48 | 77 | 0 | 8 | 7 | 0 | 0 | 0 |
| **OTU1349** | 2 | 1 | 8 | 0 | 0 | 0 | 0 | 0 | 0 |
| **OTU1350** | 0 | 0 | 0 | 2 | 3 | 2 | 1 | 2 | 0 |
| **OTU1351** | 21 | 23 | 61 | 0 | 12 | 8 | 0 | 0 | 0 |
| **OTU1352** | 7 | 0 | 0 | 0 | 0 | 0 | 0 | 0 | 0 |
| **OTU1353** | 0 | 0 | 2 | 19 | 14 | 4 | 14 | 20 | 9 |
| **OTU1354** | 15 | 2 | 10 | 0 | 0 | 0 | 0 | 0 | 0 |
| **OTU1355** | 0 | 1 | 0 | 30 | 15 | 18 | 31 | 19 | 19 |
| **OTU1356** | 2 | 2 | 3 | 77 | 23 | 21 | 34 | 29 | 28 |
| **OTU1357** | 0 | 2 | 1 | 23 | 14 | 25 | 10 | 22 | 16 |
| **OTU1358** | 0 | 2 | 4 | 0 | 0 | 0 | 0 | 0 | 0 |
| **OTU1359** | 20 | 9 | 26 | 2 | 1 | 0 | 3 | 2 | 2 |
| **OTU1360** | 3 | 1 | 5 | 36 | 21 | 21 | 25 | 24 | 21 |
| **OTU1361** | 12 | 9 | 7 | 1 | 15 | 18 | 0 | 0 | 0 |
| **OTU1362** | 7 | 3 | 8 | 3 | 0 | 0 | 0 | 0 | 0 |
| **OTU1363** | 0 | 0 | 0 | 0 | 0 | 0 | 0 | 0 | 13 |
| **OTU1364** | 303 | 244 | 535 | 1 | 187 | 110 | 1 | 1 | 2 |
| **OTU1365** | 15 | 12 | 20 | 2 | 15 | 13 | 0 | 0 | 0 |
| **OTU1366** | 0 | 0 | 0 | 5 | 0 | 1 | 2 | 6 | 5 |
| **OTU1367** | 0 | 0 | 1 | 1 | 7 | 0 | 0 | 0 | 0 |
| **OTU1368** | 0 | 0 | 0 | 0 | 0 | 10 | 0 | 0 | 0 |
| **OTU1369** | 7 | 1 | 2 | 0 | 0 | 0 | 0 | 0 | 0 |
| **OTU1370** | 2 | 0 | 7 | 14 | 6 | 11 | 18 | 5 | 2 |
| **OTU1371** | 0 | 5 | 3 | 0 | 0 | 0 | 0 | 0 | 0 |
| **OTU1372** | 6 | 2 | 9 | 32 | 16 | 12 | 25 | 17 | 21 |
| **OTU1373** | 14 | 14 | 19 | 0 | 6 | 3 | 0 | 0 | 0 |
| **OTU1374** | 1 | 4 | 4 | 66 | 14 | 0 | 62 | 76 | 19 |
| **OTU1375** | 30 | 23 | 41 | 0 | 6 | 10 | 0 | 0 | 0 |
| **OTU1376** | 0 | 0 | 0 | 0 | 6 | 0 | 0 | 0 | 0 |
| **OTU1377** | 2 | 2 | 2 | 0 | 0 | 0 | 0 | 0 | 0 |
| **OTU1378** | 0 | 0 | 0 | 0 | 6 | 0 | 0 | 0 | 0 |
| **OTU1379** | 14 | 27 | 37 | 0 | 8 | 7 | 0 | 0 | 0 |
| **OTU1380** | 6 | 5 | 10 | 0 | 0 | 0 | 0 | 0 | 0 |
| **OTU1381** | 0 | 0 | 0 | 3 | 1 | 0 | 0 | 0 | 3 |
| **OTU1382** | 0 | 1 | 0 | 0 | 0 | 7 | 0 | 0 | 0 |
| **OTU1383** | 0 | 0 | 0 | 13 | 0 | 0 | 1 | 0 | 0 |
| **OTU1384** | 96 | 77 | 127 | 1 | 49 | 41 | 0 | 0 | 0 |
| **OTU1385** | 0 | 0 | 0 | 10 | 4 | 4 | 1 | 2 | 1 |
| **OTU1386** | 39 | 38 | 62 | 0 | 11 | 11 | 0 | 0 | 0 |
| **OTU1387** | 4 | 1 | 5 | 0 | 1 | 0 | 0 | 0 | 0 |
| **OTU1388** | 57 | 37 | 56 | 1 | 3 | 6 | 0 | 0 | 0 |
| **OTU1389** | 0 | 0 | 0 | 1 | 4 | 0 | 2 | 0 | 1 |
| **OTU1390** | 0 | 0 | 0 | 9 | 5 | 2 | 3 | 1 | 2 |
| **OTU1391** | 13 | 15 | 40 | 0 | 1 | 0 | 1 | 1 | 0 |
| **OTU1392** | 0 | 1 | 6 | 96 | 44 | 29 | 46 | 42 | 38 |
| **OTU1393** | 1 | 0 | 0 | 9 | 13 | 12 | 14 | 11 | 44 |
| **OTU1394** | 3 | 1 | 1 | 0 | 0 | 0 | 0 | 0 | 0 |
| **OTU1395** | 27 | 26 | 44 | 229 | 108 | 116 | 122 | 100 | 62 |
| **OTU1396** | 48 | 60 | 240 | 2 | 2 | 3 | 0 | 5 | 1 |
| **OTU1397** | 35 | 49 | 62 | 0 | 10 | 8 | 0 | 1 | 2 |
| **OTU1398** | 1 | 1 | 5 | 0 | 1 | 0 | 0 | 0 | 0 |
| **OTU1399** | 2 | 0 | 2 | 35 | 23 | 22 | 16 | 12 | 17 |
| **OTU1400** | 0 | 0 | 0 | 3 | 1 | 0 | 3 | 2 | 2 |
| **OTU1401** | 0 | 0 | 5 | 0 | 0 | 0 | 0 | 0 | 0 |
| **OTU1402** | 1 | 0 | 1 | 56 | 16 | 15 | 31 | 8 | 13 |
| **OTU1403** | 2 | 0 | 4 | 0 | 0 | 0 | 0 | 0 | 0 |
| **OTU1404** | 0 | 1 | 0 | 29 | 8 | 11 | 14 | 8 | 5 |
| **OTU1405** | 0 | 1 | 3 | 0 | 0 | 0 | 0 | 1 | 0 |
| **OTU1406** | 4607 | 3547 | 6820 | 18 | 2102 | 2143 | 11 | 7 | 8 |
| **OTU1407** | 17 | 31 | 44 | 47 | 60 | 66 | 36 | 52 | 34 |
| **OTU1408** | 2 | 1 | 8 | 0 | 0 | 0 | 0 | 0 | 0 |
| **OTU1409** | 144 | 174 | 225 | 3 | 28 | 34 | 3 | 3 | 0 |
| **OTU1410** | 1 | 1 | 2 | 41 | 13 | 35 | 24 | 19 | 26 |
| **OTU1411** | 0 | 0 | 2 | 25 | 1 | 0 | 0 | 16 | 2 |
| **OTU1412** | 10 | 9 | 22 | 0 | 7 | 3 | 0 | 0 | 0 |
| **OTU1413** | 2 | 0 | 2 | 60 | 23 | 37 | 45 | 42 | 42 |
| **OTU1414** | 26 | 17 | 57 | 0 | 37 | 22 | 0 | 0 | 0 |
| **OTU1415** | 0 | 0 | 0 | 5 | 0 | 0 | 1 | 0 | 0 |
| **OTU1416** | 2 | 0 | 1 | 7 | 14 | 14 | 9 | 14 | 17 |
| **OTU1417** | 292 | 211 | 532 | 1 | 149 | 163 | 1 | 1 | 0 |
| **OTU1418** | 18 | 24 | 39 | 972 | 522 | 597 | 811 | 513 | 534 |
| **OTU1419** | 1 | 1 | 1 | 25 | 16 | 17 | 26 | 13 | 11 |
| **OTU1420** | 9 | 2 | 17 | 147 | 121 | 36 | 110 | 49 | 40 |
| **OTU1421** | 0 | 0 | 0 | 6 | 4 | 1 | 3 | 2 | 0 |
| **OTU1422** | 0 | 0 | 0 | 0 | 7 | 0 | 0 | 0 | 0 |
| **OTU1423** | 17 | 6 | 16 | 0 | 1 | 4 | 0 | 0 | 0 |
| **OTU1424** | 44 | 59 | 130 | 0 | 5 | 13 | 2 | 2 | 0 |
| **OTU1425** | 5 | 0 | 0 | 0 | 0 | 0 | 0 | 0 | 0 |
| **OTU1426** | 2 | 3 | 5 | 105 | 35 | 44 | 57 | 31 | 71 |
| **OTU1427** | 4 | 7 | 14 | 0 | 1 | 0 | 0 | 0 | 0 |
| **OTU1428** | 0 | 0 | 1 | 2 | 4 | 2 | 23 | 7 | 2 |
| **OTU1429** | 6 | 2 | 3 | 73 | 33 | 99 | 61 | 52 | 19 |
| **OTU1430** | 1 | 1 | 2 | 15 | 12 | 11 | 11 | 8 | 6 |
| **OTU1431** | 0 | 0 | 0 | 0 | 1 | 1 | 0 | 1 | 1 |
| **OTU1432** | 2 | 7 | 15 | 13 | 167 | 208 | 79 | 223 | 228 |
| **OTU1433** | 2 | 5 | 5 | 143 | 104 | 102 | 78 | 79 | 80 |
| **OTU1434** | 6 | 20 | 32 | 0 | 2 | 1 | 0 | 0 | 0 |
| **OTU1435** | 29 | 57 | 60 | 0 | 3 | 3 | 0 | 0 | 0 |
| **OTU1436** | 19 | 29 | 27 | 0 | 4 | 9 | 0 | 0 | 0 |
| **OTU1437** | 23 | 25 | 48 | 0 | 5 | 3 | 0 | 0 | 0 |
| **OTU1438** | 1 | 4 | 5 | 0 | 0 | 0 | 0 | 0 | 0 |
| **OTU1439** | 0 | 0 | 3 | 74 | 21 | 15 | 49 | 20 | 24 |
| **OTU1440** | 2 | 0 | 2 | 92 | 18 | 37 | 74 | 21 | 21 |
| **OTU1441** | 1 | 0 | 0 | 4 | 1 | 0 | 3 | 2 | 3 |
| **OTU1442** | 0 | 1 | 1 | 31 | 10 | 14 | 16 | 11 | 5 |
| **OTU1443** | 0 | 0 | 0 | 6 | 3 | 2 | 1 | 4 | 2 |
| **OTU1444** | 3 | 4 | 2 | 0 | 2 | 0 | 0 | 0 | 0 |
| **OTU1445** | 1 | 0 | 1 | 0 | 1 | 2 | 0 | 0 | 0 |
| **OTU1446** | 4 | 3 | 1 | 0 | 0 | 0 | 0 | 0 | 0 |
| **OTU1447** | 3 | 11 | 20 | 0 | 3 | 4 | 0 | 0 | 0 |
| **OTU1448** | 15 | 17 | 15 | 1 | 0 | 3 | 0 | 1 | 0 |
| **OTU1449** | 31 | 40 | 70 | 0 | 19 | 16 | 1 | 0 | 0 |
| **OTU1450** | 34 | 36 | 51 | 0 | 19 | 13 | 1 | 2 | 0 |
| **OTU1451** | 1 | 0 | 0 | 106 | 3 | 2 | 2 | 0 | 0 |
| **OTU1452** | 0 | 0 | 7 | 0 | 0 | 0 | 0 | 0 | 0 |
| **OTU1453** | 55 | 37 | 39 | 0 | 7 | 9 | 0 | 1 | 0 |
| **OTU1454** | 2 | 12 | 7 | 1 | 0 | 0 | 0 | 0 | 0 |
| **OTU1455** | 4 | 7 | 19 | 0 | 1 | 0 | 0 | 0 | 0 |
| **OTU1456** | 0 | 1 | 5 | 0 | 0 | 0 | 0 | 0 | 0 |
| **OTU1457** | 29 | 40 | 65 | 0 | 5 | 7 | 0 | 1 | 0 |
| **OTU1458** | 6 | 0 | 0 | 0 | 0 | 0 | 0 | 0 | 0 |
| **OTU1459** | 8 | 3 | 1 | 0 | 1 | 0 | 0 | 0 | 0 |
| **OTU1460** | 6 | 0 | 0 | 0 | 0 | 0 | 0 | 0 | 0 |
| **OTU1461** | 7 | 8 | 10 | 0 | 0 | 0 | 0 | 0 | 0 |
| **OTU1462** | 7 | 10 | 18 | 0 | 0 | 3 | 0 | 0 | 0 |
| **OTU1463** | 4 | 6 | 3 | 0 | 1 | 1 | 0 | 0 | 0 |
| **OTU1464** | 6 | 1 | 1 | 0 | 0 | 0 | 0 | 0 | 0 |
| **OTU1465** | 15 | 13 | 27 | 0 | 3 | 5 | 2 | 0 | 0 |
| **OTU1466** | 6 | 6 | 6 | 0 | 0 | 3 | 0 | 0 | 1 |
| **OTU1467** | 11 | 10 | 17 | 106 | 85 | 38 | 118 | 25 | 32 |
| **OTU1468** | 12 | 8 | 11 | 1 | 0 | 1 | 0 | 0 | 0 |
| **OTU1469** | 1 | 0 | 6 | 0 | 0 | 0 | 0 | 0 | 0 |
| **OTU1470** | 30 | 35 | 44 | 1 | 4 | 10 | 0 | 0 | 0 |
| **OTU1471** | 4 | 5 | 13 | 0 | 0 | 1 | 0 | 0 | 0 |
| **OTU1472** | 11 | 14 | 27 | 679 | 233 | 285 | 459 | 357 | 311 |
| **OTU1473** | 8 | 3 | 17 | 87 | 27 | 28 | 37 | 24 | 15 |
| **OTU1474** | 0 | 1 | 1 | 1 | 10 | 3 | 107 | 1 | 18 |
| **OTU1475** | 0 | 0 | 1 | 32 | 1 | 2 | 1 | 3 | 1 |
| **OTU1476** | 16 | 24 | 55 | 0 | 8 | 4 | 0 | 0 | 0 |
| **OTU1477** | 0 | 0 | 0 | 1 | 1 | 0 | 2 | 0 | 1 |
| **OTU1478** | 170 | 154 | 335 | 2 | 62 | 49 | 0 | 0 | 1 |
| **OTU1479** | 0 | 1 | 1 | 19 | 16 | 10 | 21 | 14 | 13 |
| **OTU1480** | 6 | 3 | 1 | 24 | 6 | 8 | 13 | 8 | 5 |
| **OTU1481** | 46 | 35 | 67 | 0 | 4 | 3 | 0 | 0 | 0 |
| **OTU1482** | 156 | 110 | 210 | 0 | 54 | 66 | 0 | 0 | 0 |
| **OTU1483** | 0 | 0 | 0 | 0 | 5 | 2 | 0 | 2 | 3 |
| **OTU1484** | 24 | 16 | 32 | 282 | 176 | 108 | 219 | 101 | 81 |
| **OTU1485** | 1 | 0 | 0 | 16 | 1 | 32 | 0 | 33 | 6 |
| **OTU1486** | 21 | 14 | 25 | 0 | 1 | 5 | 1 | 1 | 0 |
| **OTU1487** | 0 | 0 | 0 | 0 | 5 | 0 | 0 | 0 | 0 |
| **OTU1488** | 0 | 0 | 4 | 0 | 0 | 0 | 0 | 0 | 0 |
| **OTU1489** | 0 | 0 | 6 | 0 | 0 | 0 | 0 | 0 | 0 |
| **OTU1490** | 46 | 57 | 74 | 0 | 3 | 15 | 0 | 0 | 0 |
| **OTU1491** | 1 | 0 | 4 | 0 | 7 | 0 | 0 | 0 | 0 |
| **OTU1492** | 7 | 3 | 0 | 0 | 0 | 0 | 0 | 0 | 0 |
| **OTU1493** | 0 | 0 | 4 | 90 | 33 | 40 | 52 | 24 | 42 |
| **OTU1494** | 14 | 11 | 17 | 175 | 101 | 50 | 195 | 53 | 65 |
| **OTU1495** | 11 | 25 | 26 | 0 | 11 | 6 | 0 | 0 | 0 |
| **OTU1496** | 7 | 14 | 17 | 0 | 0 | 0 | 0 | 0 | 0 |
| **OTU1497** | 3 | 3 | 0 | 18 | 11 | 10 | 25 | 18 | 10 |
| **OTU1498** | 0 | 0 | 0 | 0 | 0 | 6 | 0 | 0 | 0 |
| **OTU1499** | 6 | 0 | 0 | 0 | 0 | 0 | 0 | 0 | 0 |
| **OTU1500** | 103 | 87 | 141 | 917 | 434 | 259 | 704 | 289 | 270 |
| **OTU1501** | 11 | 8 | 15 | 0 | 4 | 1 | 0 | 0 | 0 |
| **OTU1502** | 1 | 2 | 0 | 16 | 3 | 5 | 4 | 3 | 3 |
| **OTU1503** | 6 | 2 | 3 | 0 | 0 | 0 | 0 | 0 | 0 |
| **OTU1504** | 177 | 112 | 186 | 3 | 34 | 18 | 2 | 1 | 1 |
| **OTU1505** | 1 | 0 | 0 | 0 | 10 | 0 | 0 | 0 | 13 |
| **OTU1506** | 0 | 0 | 6 | 0 | 0 | 0 | 0 | 0 | 0 |
| **OTU1507** | 0 | 0 | 5 | 0 | 0 | 0 | 0 | 0 | 0 |
| **OTU1508** | 3 | 2 | 6 | 4 | 10 | 4 | 2 | 3 | 0 |
| **OTU1509** | 23 | 21 | 39 | 45 | 746 | 644 | 201 | 420 | 420 |
| **OTU1510** | 0 | 1 | 0 | 0 | 0 | 8 | 0 | 0 | 0 |
| **OTU1511** | 75 | 73 | 135 | 0 | 19 | 21 | 0 | 0 | 0 |
| **OTU1512** | 32 | 36 | 56 | 1 | 4 | 1 | 2 | 0 | 2 |
| **OTU1513** | 149 | 101 | 245 | 0 | 17 | 2 | 0 | 0 | 0 |
| **OTU1514** | 2 | 4 | 9 | 0 | 0 | 0 | 0 | 0 | 0 |
| **OTU1515** | 34 | 35 | 79 | 0 | 6 | 6 | 0 | 1 | 0 |
| **OTU1516** | 6 | 3 | 7 | 4 | 0 | 0 | 23 | 1 | 0 |
| **OTU1517** | 5 | 8 | 18 | 0 | 1 | 0 | 0 | 1 | 0 |
| **OTU1518** | 0 | 0 | 0 | 23 | 14 | 8 | 11 | 14 | 9 |
| **OTU1519** | 22 | 5 | 19 | 113 | 42 | 244 | 244 | 115 | 93 |
| **OTU1520** | 1 | 0 | 0 | 7 | 6 | 0 | 0 | 2 | 0 |
| **OTU1521** | 1 | 1 | 3 | 89 | 31 | 31 | 57 | 19 | 24 |
| **OTU1522** | 2 | 2 | 4 | 0 | 0 | 2 | 0 | 0 | 0 |
| **OTU1523** | 30 | 25 | 87 | 0 | 13 | 10 | 0 | 0 | 0 |
| **OTU1524** | 8 | 10 | 15 | 0 | 2 | 0 | 0 | 0 | 2 |
| **OTU1525** | 35 | 20 | 61 | 1426 | 585 | 577 | 928 | 489 | 747 |
| **OTU1526** | 0 | 0 | 6 | 0 | 0 | 0 | 0 | 0 | 0 |
| **OTU1527** | 1517 | 1045 | 2064 | 24 | 200 | 247 | 15 | 9 | 11 |
| **OTU1528** | 1 | 5 | 7 | 46 | 25 | 24 | 95 | 42 | 26 |
| **OTU1529** | 75 | 60 | 112 | 3 | 18 | 20 | 2 | 1 | 4 |
| **OTU1530** | 6 | 9 | 12 | 394 | 262 | 229 | 234 | 198 | 138 |
| **OTU1531** | 3 | 4 | 3 | 1 | 0 | 0 | 0 | 0 | 0 |
| **OTU1532** | 0 | 0 | 0 | 0 | 5 | 0 | 0 | 0 | 0 |
| **OTU1533** | 393 | 363 | 542 | 2 | 157 | 142 | 0 | 0 | 1 |
| **OTU1534** | 0 | 1 | 5 | 42 | 14 | 9 | 18 | 14 | 15 |
| **OTU1535** | 37 | 32 | 72 | 0 | 16 | 11 | 0 | 0 | 0 |
| **OTU1536** | 1 | 2 | 4 | 95 | 48 | 41 | 46 | 39 | 47 |
| **OTU1537** | 3 | 5 | 1 | 17 | 9 | 41 | 33 | 21 | 19 |
| **OTU1538** | 3 | 1 | 4 | 0 | 0 | 0 | 0 | 0 | 0 |
| **OTU1539** | 5 | 4 | 13 | 1 | 0 | 0 | 1 | 0 | 0 |
| **OTU1540** | 72 | 89 | 104 | 0 | 23 | 26 | 0 | 0 | 0 |
| **OTU1541** | 0 | 0 | 1 | 20 | 8 | 20 | 11 | 15 | 10 |
| **OTU1542** | 11 | 5 | 10 | 36 | 18 | 22 | 24 | 20 | 18 |
| **OTU1543** | 12 | 11 | 9 | 0 | 26 | 22 | 0 | 0 | 0 |
| **OTU1544** | 0 | 0 | 0 | 1 | 1 | 8 | 25 | 0 | 10 |
| **OTU1545** | 13 | 3 | 12 | 496 | 113 | 115 | 212 | 139 | 124 |
| **OTU1546** | 1 | 1 | 11 | 223 | 133 | 129 | 123 | 115 | 109 |
| **OTU1547** | 1 | 0 | 0 | 0 | 0 | 6 | 0 | 0 | 0 |
| **OTU1548** | 84 | 60 | 104 | 0 | 13 | 16 | 2 | 0 | 0 |
| **OTU1549** | 0 | 0 | 0 | 0 | 6 | 0 | 0 | 0 | 1 |
| **OTU1550** | 164 | 98 | 215 | 5 | 26 | 39 | 1 | 1 | 0 |
| **OTU1551** | 33 | 27 | 44 | 0 | 0 | 1 | 0 | 0 | 0 |
| **OTU1552** | 114 | 137 | 170 | 0 | 19 | 26 | 0 | 0 | 0 |
| **OTU1553** | 0 | 0 | 0 | 0 | 0 | 0 | 0 | 5 | 0 |
| **OTU1554** | 0 | 1 | 1 | 33 | 20 | 13 | 28 | 14 | 18 |
| **OTU1555** | 103 | 116 | 198 | 3 | 1 | 1 | 2 | 0 | 0 |
| **OTU1556** | 0 | 0 | 1 | 2 | 2 | 7 | 11 | 6 | 4 |
| **OTU1557** | 5 | 4 | 5 | 0 | 4 | 5 | 0 | 0 | 0 |
| **OTU1558** | 0 | 1 | 3 | 2 | 27 | 34 | 6 | 20 | 17 |
| **OTU1559** | 5 | 5 | 14 | 0 | 0 | 0 | 0 | 0 | 0 |
| **OTU1560** | 14 | 9 | 10 | 0 | 1 | 1 | 0 | 0 | 0 |
| **OTU1561** | 26 | 21 | 31 | 0 | 2 | 7 | 0 | 0 | 0 |
| **OTU1562** | 0 | 0 | 1 | 3 | 5 | 3 | 1 | 4 | 1 |
| **OTU1563** | 5 | 0 | 1 | 1 | 11 | 22 | 11 | 21 | 12 |
| **OTU1564** | 86 | 69 | 129 | 0 | 45 | 43 | 1 | 1 | 0 |
| **OTU1565** | 25 | 18 | 62 | 14 | 2 | 5 | 5 | 13 | 0 |
| **OTU1566** | 9 | 4 | 17 | 0 | 1 | 2 | 2 | 0 | 0 |
| **OTU1567** | 42 | 20 | 52 | 699 | 468 | 508 | 845 | 334 | 220 |
| **OTU1568** | 16 | 13 | 22 | 24 | 6 | 6 | 73 | 34 | 2 |
| **OTU1569** | 10 | 3 | 20 | 67 | 12 | 0 | 6 | 2 | 0 |
| **OTU1570** | 0 | 1 | 0 | 28 | 12 | 7 | 13 | 15 | 10 |
| **OTU1571** | 468 | 388 | 636 | 3 | 191 | 160 | 1 | 0 | 1 |
| **OTU1572** | 6 | 8 | 11 | 300 | 142 | 141 | 186 | 153 | 137 |
| **OTU1573** | 47 | 43 | 93 | 0 | 20 | 34 | 0 | 0 | 1 |
| **OTU1574** | 12 | 28 | 27 | 0 | 2 | 2 | 0 | 0 | 0 |
| **OTU1575** | 4 | 0 | 4 | 96 | 75 | 60 | 93 | 47 | 41 |
| **OTU1576** | 26 | 40 | 56 | 0 | 6 | 7 | 0 | 0 | 0 |
| **OTU1577** | 0 | 0 | 0 | 0 | 6 | 0 | 1 | 0 | 0 |
| **OTU1578** | 77 | 94 | 174 | 0 | 21 | 17 | 0 | 0 | 0 |
| **OTU1579** | 57 | 41 | 69 | 0 | 50 | 57 | 0 | 0 | 0 |
| **OTU1580** | 8 | 2 | 5 | 3 | 0 | 17 | 0 | 0 | 3 |
| **OTU1581** | 0 | 0 | 0 | 1 | 5 | 0 | 0 | 0 | 0 |
| **OTU1582** | 0 | 1 | 2 | 34 | 0 | 19 | 0 | 0 | 0 |
| **OTU1583** | 35 | 17 | 49 | 0 | 11 | 15 | 0 | 0 | 0 |
| **OTU1584** | 5 | 0 | 1 | 0 | 0 | 0 | 0 | 0 | 0 |
| **OTU1585** | 0 | 3 | 4 | 0 | 2 | 0 | 0 | 0 | 0 |
| **OTU1586** | 0 | 0 | 0 | 0 | 0 | 0 | 0 | 1 | 7 |
| **OTU1587** | 269 | 189 | 368 | 43 | 142 | 146 | 40 | 30 | 18 |
| **OTU1588** | 30 | 30 | 42 | 0 | 1 | 0 | 1 | 0 | 0 |
| **OTU1589** | 2 | 0 | 0 | 21 | 9 | 14 | 20 | 17 | 9 |
| **OTU1590** | 69 | 50 | 89 | 5 | 4 | 3 | 7 | 0 | 4 |
| **OTU1591** | 13 | 9 | 22 | 0 | 4 | 2 | 0 | 0 | 0 |
| **OTU1592** | 0 | 1 | 10 | 0 | 0 | 1 | 0 | 0 | 0 |
| **OTU1593** | 0 | 0 | 6 | 0 | 0 | 0 | 0 | 0 | 0 |
| **OTU1594** | 1 | 6 | 9 | 0 | 1 | 0 | 0 | 0 | 0 |
| **OTU1595** | 9 | 11 | 16 | 371 | 220 | 190 | 169 | 224 | 210 |
| **OTU1596** | 12 | 15 | 40 | 1 | 26 | 0 | 1 | 1 | 1 |
| **OTU1597** | 0 | 0 | 0 | 3 | 1 | 2 | 2 | 1 | 0 |
| **OTU1598** | 38 | 26 | 39 | 0 | 0 | 0 | 2 | 0 | 0 |
| **OTU1599** | 5 | 0 | 2 | 66 | 17 | 15 | 49 | 30 | 2 |
| **OTU1600** | 0 | 0 | 4 | 0 | 0 | 0 | 0 | 0 | 0 |
| **OTU1601** | 157 | 75 | 224 | 1 | 18 | 7 | 0 | 2 | 1 |
| **OTU1602** | 0 | 0 | 0 | 5 | 1 | 0 | 1 | 1 | 4 |
| **OTU1603** | 2 | 2 | 0 | 30 | 21 | 21 | 15 | 16 | 11 |
| **OTU1604** | 1 | 1 | 5 | 0 | 0 | 0 | 0 | 0 | 0 |
| **OTU1605** | 27 | 20 | 36 | 0 | 4 | 1 | 0 | 1 | 0 |
| **OTU1606** | 1 | 0 | 2 | 0 | 3 | 0 | 0 | 0 | 0 |
| **OTU1607** | 0 | 0 | 6 | 0 | 0 | 0 | 0 | 0 | 0 |
| **OTU1608** | 3 | 2 | 0 | 0 | 0 | 0 | 0 | 0 | 0 |
| **OTU1609** | 0 | 2 | 2 | 6 | 2 | 19 | 27 | 14 | 5 |
| **OTU1610** | 61 | 40 | 134 | 3 | 14 | 2 | 0 | 1 | 2 |
| **OTU1611** | 5 | 4 | 16 | 0 | 1 | 0 | 0 | 0 | 0 |
| **OTU1612** | 40 | 51 | 64 | 0 | 14 | 13 | 0 | 0 | 0 |
| **OTU1613** | 2 | 1 | 4 | 3 | 3 | 3 | 5 | 2 | 1 |
| **OTU1614** | 13 | 6 | 7 | 0 | 0 | 4 | 0 | 0 | 0 |
| **OTU1615** | 14 | 7 | 27 | 0 | 8 | 3 | 0 | 0 | 0 |
| **OTU1616** | 1 | 0 | 0 | 0 | 3 | 14 | 0 | 0 | 0 |
| **OTU1617** | 27 | 19 | 76 | 4 | 0 | 29 | 1 | 11 | 3 |
| **OTU1618** | 33 | 33 | 67 | 1392 | 856 | 688 | 789 | 805 | 667 |
| **OTU1619** | 74 | 53 | 128 | 1 | 44 | 43 | 1 | 0 | 0 |
| **OTU1620** | 34 | 31 | 50 | 0 | 4 | 7 | 0 | 0 | 0 |
| **OTU1621** | 12 | 14 | 22 | 0 | 6 | 5 | 0 | 0 | 0 |
| **OTU1622** | 3 | 7 | 12 | 0 | 4 | 0 | 0 | 0 | 0 |
| **OTU1623** | 0 | 0 | 4 | 0 | 0 | 0 | 0 | 0 | 0 |
| **OTU1624** | 0 | 0 | 1 | 10 | 4 | 1 | 5 | 5 | 1 |
| **OTU1625** | 0 | 0 | 0 | 1 | 0 | 3 | 0 | 0 | 0 |
| **OTU1626** | 6 | 14 | 23 | 0 | 1 | 0 | 0 | 0 | 0 |
| **OTU1627** | 0 | 0 | 0 | 0 | 2 | 0 | 43 | 1 | 0 |
| **OTU1628** | 12 | 9 | 20 | 59 | 248 | 271 | 134 | 311 | 344 |
| **OTU1629** | 108 | 103 | 229 | 5119 | 2312 | 2534 | 3097 | 1799 | 1573 |
| **OTU1630** | 1 | 4 | 2 | 41 | 20 | 12 | 19 | 17 | 15 |
| **OTU1631** | 0 | 0 | 0 | 2 | 4 | 0 | 0 | 0 | 0 |
| **OTU1632** | 82 | 51 | 129 | 7 | 1 | 3 | 3 | 1 | 1 |
| **OTU1633** | 280 | 183 | 443 | 2 | 90 | 67 | 0 | 1 | 2 |
| **OTU1634** | 0 | 0 | 0 | 0 | 1 | 0 | 0 | 3 | 0 |
| **OTU1635** | 0 | 0 | 1 | 12 | 7 | 3 | 13 | 4 | 9 |
| **OTU1636** | 87 | 68 | 209 | 653 | 2169 | 2543 | 1332 | 2289 | 2456 |
| **OTU1637** | 0 | 0 | 0 | 0 | 4 | 0 | 0 | 0 | 0 |
| **OTU1638** | 6 | 3 | 4 | 0 | 0 | 0 | 0 | 0 | 0 |
| **OTU1639** | 0 | 0 | 0 | 0 | 0 | 4 | 0 | 0 | 0 |
| **OTU1640** | 4 | 3 | 2 | 0 | 0 | 0 | 0 | 0 | 0 |
| **OTU1641** | 317 | 307 | 423 | 8 | 33 | 19 | 6 | 4 | 3 |
| **OTU1642** | 3 | 0 | 3 | 0 | 0 | 0 | 0 | 0 | 0 |
| **OTU1643** | 0 | 2 | 1 | 128 | 52 | 24 | 1 | 4 | 33 |
| **OTU1644** | 19 | 12 | 30 | 20 | 25 | 25 | 12 | 19 | 12 |
| **OTU1645** | 0 | 1 | 2 | 30 | 6 | 14 | 22 | 17 | 14 |
| **OTU1646** | 0 | 0 | 0 | 19 | 1 | 0 | 0 | 0 | 10 |
| **OTU1647** | 1116 | 718 | 1386 | 36 | 35 | 27 | 12 | 8 | 12 |
| **OTU1648** | 0 | 1 | 2 | 19 | 12 | 18 | 6 | 16 | 8 |
| **OTU1649** | 0 | 0 | 0 | 0 | 3 | 0 | 1 | 0 | 0 |
| **OTU1650** | 7 | 4 | 17 | 8 | 48 | 94 | 19 | 79 | 88 |
| **OTU1651** | 4 | 2 | 4 | 116 | 63 | 50 | 111 | 37 | 15 |
| **OTU1652** | 1 | 3 | 7 | 0 | 0 | 0 | 0 | 0 | 0 |
| **OTU1653** | 6 | 2 | 6 | 16 | 8 | 5 | 6 | 5 | 7 |
| **OTU1654** | 0 | 0 | 2 | 4 | 15 | 19 | 6 | 20 | 14 |
| **OTU1655** | 0 | 2 | 1 | 0 | 6 | 8 | 0 | 17 | 3 |
| **OTU1656** | 356 | 476 | 662 | 3 | 58 | 83 | 1 | 2 | 0 |
| **OTU1657** | 492 | 538 | 871 | 1 | 126 | 123 | 0 | 2 | 3 |
| **OTU1658** | 95 | 121 | 184 | 1 | 16 | 17 | 0 | 0 | 0 |
| **OTU1659** | 3483 | 2782 | 5161 | 8 | 1647 | 1349 | 5 | 8 | 2 |
| **OTU1660** | 66 | 43 | 113 | 0 | 27 | 17 | 0 | 0 | 0 |
| **OTU1661** | 3 | 2 | 1 | 102 | 41 | 37 | 47 | 30 | 35 |
| **OTU1662** | 9 | 7 | 12 | 173 | 51 | 49 | 59 | 42 | 36 |
| **OTU1663** | 19 | 11 | 13 | 0 | 4 | 1 | 0 | 0 | 0 |
| **OTU1664** | 2 | 5 | 19 | 0 | 0 | 0 | 0 | 0 | 0 |
| **OTU1665** | 5 | 6 | 6 | 85 | 20 | 9 | 81 | 69 | 79 |
| **OTU1666** | 92 | 107 | 179 | 0 | 20 | 25 | 0 | 0 | 0 |
| **OTU1667** | 1 | 0 | 1 | 1 | 46 | 34 | 11 | 33 | 24 |
| **OTU1668** | 143 | 110 | 240 | 6763 | 3578 | 3102 | 4729 | 3161 | 3049 |
| **OTU1669** | 1 | 2 | 3 | 49 | 26 | 33 | 31 | 21 | 18 |
| **OTU1670** | 21 | 26 | 43 | 5 | 3 | 0 | 9 | 4 | 3 |
| **OTU1671** | 0 | 0 | 0 | 8 | 0 | 0 | 0 | 0 | 0 |
| **OTU1672** | 1 | 1 | 2 | 81 | 37 | 40 | 66 | 21 | 47 |
| **OTU1673** | 4 | 1 | 0 | 0 | 0 | 0 | 0 | 0 | 0 |
| **OTU1674** | 77 | 70 | 120 | 0 | 34 | 35 | 0 | 0 | 0 |
| **OTU1675** | 7 | 0 | 0 | 0 | 0 | 0 | 0 | 0 | 0 |
| **OTU1676** | 1 | 4 | 1 | 1 | 5 | 20 | 1 | 1 | 0 |
| **OTU1677** | 65 | 62 | 104 | 0 | 19 | 14 | 0 | 0 | 0 |
| **OTU1678** | 1 | 6 | 4 | 0 | 0 | 0 | 0 | 0 | 0 |
| **OTU1679** | 0 | 0 | 0 | 0 | 3 | 0 | 0 | 0 | 0 |
| **OTU1680** | 129 | 95 | 180 | 1 | 68 | 69 | 1 | 1 | 1 |
| **OTU1681** | 0 | 3 | 0 | 0 | 1 | 2 | 0 | 2 | 1 |
| **OTU1682** | 0 | 0 | 0 | 3 | 1 | 1 | 4 | 3 | 0 |
| **OTU1683** | 4 | 0 | 2 | 0 | 1 | 0 | 0 | 0 | 0 |
| **OTU1684** | 22 | 18 | 30 | 1 | 7 | 6 | 0 | 1 | 0 |
| **OTU1685** | 18 | 31 | 43 | 0 | 5 | 1 | 0 | 0 | 0 |
| **OTU1686** | 654 | 438 | 868 | 9 | 89 | 109 | 6 | 5 | 3 |
| **OTU1687** | 2 | 6 | 5 | 0 | 1 | 3 | 0 | 0 | 0 |
| **OTU1688** | 29 | 17 | 17 | 631 | 309 | 317 | 406 | 329 | 301 |
| **OTU1689** | 42 | 29 | 80 | 1 | 1 | 2 | 0 | 0 | 0 |
| **OTU1690** | 0 | 0 | 1 | 19 | 6 | 3 | 12 | 2 | 4 |
| **OTU1691** | 4 | 5 | 8 | 0 | 0 | 1 | 0 | 0 | 0 |
| **OTU1692** | 4 | 5 | 11 | 1 | 0 | 1 | 0 | 0 | 0 |
| **OTU1693** | 205 | 202 | 536 | 4352 | 6608 | 6623 | 4890 | 6281 | 7131 |
| **OTU1694** | 0 | 0 | 0 | 0 | 6 | 0 | 0 | 0 | 0 |
| **OTU1695** | 0 | 0 | 5 | 0 | 0 | 0 | 0 | 0 | 0 |
| **OTU1696** | 3 | 0 | 4 | 1 | 1 | 1 | 0 | 0 | 0 |
| **OTU1697** | 26 | 27 | 57 | 0 | 12 | 25 | 0 | 0 | 0 |
| **OTU1698** | 16 | 24 | 32 | 946 | 508 | 322 | 564 | 353 | 484 |
| **OTU1699** | 22 | 17 | 30 | 0 | 2 | 3 | 1 | 0 | 0 |
| **OTU1700** | 4 | 2 | 0 | 0 | 4 | 0 | 0 | 0 | 0 |
| **OTU1701** | 5 | 4 | 0 | 0 | 0 | 0 | 0 | 0 | 0 |
| **OTU1702** | 3 | 4 | 7 | 10 | 2 | 4 | 2 | 6 | 1 |
| **OTU1703** | 565 | 303 | 765 | 8 | 26 | 34 | 4 | 3 | 2 |
| **OTU1704** | 0 | 0 | 0 | 2 | 0 | 1 | 3 | 4 | 4 |
| **OTU1705** | 14 | 25 | 37 | 0 | 7 | 8 | 0 | 0 | 0 |
| **OTU1706** | 4 | 0 | 0 | 1 | 0 | 11 | 1 | 0 | 1 |
| **OTU1707** | 1 | 0 | 0 | 17 | 13 | 8 | 13 | 4 | 1 |
| **OTU1708** | 9 | 3 | 11 | 0 | 2 | 9 | 0 | 0 | 0 |
| **OTU1709** | 0 | 0 | 1 | 3 | 20 | 13 | 2 | 9 | 4 |
| **OTU1710** | 2 | 5 | 3 | 146 | 36 | 45 | 45 | 43 | 32 |
| **OTU1711** | 0 | 0 | 1 | 0 | 6 | 6 | 0 | 0 | 0 |
| **OTU1712** | 0 | 0 | 0 | 41 | 0 | 0 | 0 | 0 | 0 |
| **OTU1713** | 1 | 0 | 0 | 2 | 1 | 7 | 1 | 3 | 1 |
| **OTU1714** | 0 | 2 | 1 | 10 | 6 | 11 | 9 | 6 | 2 |
| **OTU1715** | 2 | 6 | 4 | 0 | 1 | 1 | 0 | 0 | 0 |
| **OTU1716** | 12 | 8 | 33 | 0 | 4 | 4 | 0 | 0 | 0 |
| **OTU1717** | 250 | 260 | 414 | 1 | 61 | 45 | 0 | 1 | 1 |
| **OTU1718** | 16 | 9 | 35 | 0 | 5 | 6 | 0 | 0 | 0 |
| **OTU1719** | 16 | 20 | 40 | 0 | 6 | 8 | 0 | 0 | 0 |
| **OTU1720** | 14 | 13 | 19 | 543 | 296 | 253 | 417 | 378 | 356 |
| **OTU1721** | 22 | 14 | 43 | 0 | 3 | 4 | 0 | 0 | 0 |
| **OTU1722** | 2 | 1 | 0 | 6 | 1 | 0 | 2 | 4 | 0 |
| **OTU1723** | 1255 | 979 | 2018 | 8 | 529 | 452 | 1 | 1 | 2 |
| **OTU1724** | 1 | 4 | 1 | 0 | 0 | 0 | 0 | 0 | 0 |
| **OTU1725** | 61 | 46 | 78 | 0 | 52 | 50 | 0 | 0 | 0 |
| **OTU1726** | 4 | 9 | 20 | 0 | 10 | 8 | 0 | 0 | 0 |
| **OTU1727** | 2 | 2 | 8 | 0 | 1 | 0 | 0 | 0 | 0 |
| **OTU1728** | 9 | 18 | 45 | 1 | 1 | 4 | 0 | 0 | 1 |
| **OTU1729** | 5 | 1 | 3 | 14 | 6 | 51 | 45 | 20 | 17 |
| **OTU1730** | 88 | 116 | 163 | 1 | 0 | 4 | 0 | 0 | 1 |
| **OTU1731** | 55 | 52 | 105 | 0 | 1 | 3 | 0 | 0 | 0 |
| **OTU1732** | 0 | 0 | 0 | 7 | 3 | 3 | 7 | 0 | 3 |
| **OTU1733** | 2 | 0 | 2 | 29 | 11 | 17 | 20 | 15 | 4 |
| **OTU1734** | 21 | 19 | 36 | 0 | 2 | 1 | 0 | 1 | 0 |
| **OTU1735** | 5 | 1 | 2 | 0 | 0 | 0 | 0 | 0 | 0 |
| **OTU1736** | 0 | 0 | 0 | 4 | 2 | 5 | 0 | 4 | 0 |
| **OTU1737** | 26 | 35 | 58 | 50 | 39 | 60 | 27 | 43 | 26 |
| **OTU1738** | 78 | 58 | 111 | 3304 | 1818 | 1411 | 1760 | 1568 | 1576 |
| **OTU1739** | 80 | 54 | 112 | 1 | 16 | 21 | 0 | 2 | 0 |
| **OTU1740** | 165 | 113 | 236 | 0 | 56 | 54 | 0 | 0 | 0 |
| **OTU1741** | 35 | 6 | 31 | 6 | 5 | 107 | 33 | 0 | 1 |
| **OTU1742** | 0 | 0 | 0 | 6 | 1 | 1 | 3 | 4 | 1 |
| **OTU1743** | 1 | 1 | 0 | 44 | 21 | 11 | 22 | 13 | 25 |
| **OTU1744** | 558 | 428 | 872 | 8 | 101 | 128 | 6 | 7 | 4 |
| **OTU1745** | 159 | 180 | 289 | 0 | 48 | 47 | 0 | 2 | 1 |
| **OTU1746** | 80 | 57 | 117 | 2281 | 1048 | 1114 | 2063 | 1094 | 1121 |
| **OTU1747** | 5 | 13 | 7 | 3 | 0 | 1 | 0 | 0 | 0 |
| **OTU1748** | 1 | 4 | 4 | 3 | 33 | 13 | 12 | 29 | 17 |
| **OTU1749** | 6860 | 5322 | 9762 | 21 | 3208 | 2650 | 18 | 22 | 23 |
| **OTU1750** | 0 | 0 | 0 | 0 | 5 | 0 | 0 | 0 | 0 |
| **OTU1751** | 24 | 5 | 30 | 0 | 0 | 0 | 0 | 1 | 1 |
| **OTU1752** | 1 | 2 | 0 | 8 | 2 | 2 | 4 | 1 | 3 |
| **OTU1753** | 3 | 4 | 13 | 0 | 8 | 1 | 0 | 0 | 0 |
| **OTU1754** | 0 | 0 | 0 | 2 | 0 | 4 | 3 | 0 | 4 |
| **OTU1755** | 17 | 30 | 45 | 0 | 1 | 10 | 1 | 0 | 0 |
| **OTU1756** | 13 | 9 | 22 | 0 | 4 | 2 | 1 | 1 | 0 |
| **OTU1757** | 8112 | 5994 | 11368 | 28 | 3589 | 4057 | 21 | 24 | 15 |
| **OTU1758** | 5 | 0 | 8 | 0 | 1 | 0 | 0 | 0 | 0 |
| **OTU1759** | 0 | 0 | 0 | 16 | 19 | 26 | 0 | 1 | 0 |
| **OTU1760** | 0 | 0 | 0 | 2 | 0 | 2 | 0 | 0 | 1 |
| **OTU1761** | 6 | 5 | 12 | 0 | 0 | 2 | 0 | 0 | 0 |
| **OTU1762** | 0 | 0 | 0 | 41 | 8 | 12 | 13 | 7 | 8 |
| **OTU1763** | 18 | 11 | 28 | 878 | 330 | 431 | 485 | 405 | 415 |
| **OTU1764** | 4 | 1 | 6 | 0 | 1 | 0 | 1 | 0 | 0 |
| **OTU1765** | 1 | 0 | 0 | 21 | 0 | 0 | 0 | 26 | 8 |
| **OTU1766** | 0 | 0 | 1 | 20 | 2 | 4 | 6 | 4 | 2 |
| **OTU1767** | 20 | 8 | 14 | 0 | 0 | 1 | 0 | 0 | 0 |
| **OTU1768** | 85 | 61 | 101 | 0 | 32 | 30 | 0 | 0 | 0 |
| **OTU1769** | 0 | 0 | 0 | 0 | 7 | 0 | 1 | 0 | 0 |
| **OTU1770** | 3 | 5 | 9 | 0 | 0 | 2 | 0 | 0 | 0 |
| **OTU1771** | 19 | 30 | 42 | 0 | 0 | 1 | 0 | 0 | 2 |
| **OTU1772** | 3 | 3 | 0 | 0 | 3 | 4 | 0 | 0 | 0 |
| **OTU1773** | 0 | 2 | 4 | 0 | 0 | 0 | 0 | 0 | 0 |
| **OTU1774** | 1 | 1 | 0 | 13 | 7 | 13 | 15 | 7 | 9 |
| **OTU1775** | 70 | 41 | 69 | 29 | 0 | 0 | 0 | 2 | 1 |
| **OTU1776** | 0 | 0 | 1 | 10 | 0 | 0 | 1 | 2 | 0 |
| **OTU1777** | 1 | 0 | 3 | 19 | 39 | 43 | 25 | 47 | 33 |
| **OTU1778** | 44 | 37 | 47 | 0 | 3 | 9 | 0 | 0 | 0 |
| **OTU1779** | 0 | 0 | 0 | 0 | 0 | 4 | 0 | 0 | 0 |
| **OTU1780** | 5 | 4 | 7 | 104 | 49 | 53 | 46 | 46 | 30 |
| **OTU1781** | 101 | 93 | 399 | 6 | 15 | 2 | 1 | 8 | 1 |
| **OTU1782** | 7 | 9 | 14 | 233 | 117 | 134 | 224 | 121 | 135 |
| **OTU1783** | 487 | 279 | 629 | 6 | 14 | 29 | 6 | 3 | 1 |
| **OTU1784** | 6 | 0 | 1 | 8 | 1 | 16 | 0 | 13 | 1 |
| **OTU1785** | 7 | 5 | 9 | 0 | 1 | 1 | 0 | 0 | 0 |
| **OTU1786** | 0 | 0 | 0 | 15 | 4 | 11 | 10 | 5 | 3 |
| **OTU1787** | 1 | 2 | 1 | 15 | 11 | 5 | 5 | 10 | 8 |
| **OTU1788** | 3 | 0 | 0 | 8 | 23 | 13 | 10 | 9 | 30 |
| **OTU1789** | 0 | 0 | 0 | 9 | 0 | 0 | 0 | 23 | 9 |
| **OTU1790** | 0 | 0 | 4 | 0 | 0 | 0 | 0 | 0 | 0 |
| **OTU1791** | 11 | 7 | 13 | 375 | 252 | 177 | 315 | 287 | 235 |
| **OTU1792** | 14 | 16 | 31 | 1019 | 290 | 322 | 322 | 421 | 340 |
| **OTU1793** | 32 | 58 | 88 | 0 | 12 | 13 | 0 | 0 | 0 |
| **OTU1794** | 0 | 0 | 1 | 6 | 1 | 3 | 2 | 3 | 1 |
| **OTU1795** | 4 | 1 | 0 | 0 | 0 | 0 | 0 | 0 | 0 |
| **OTU1796** | 0 | 0 | 4 | 0 | 0 | 0 | 0 | 0 | 0 |
| **OTU1797** | 56 | 26 | 93 | 0 | 54 | 26 | 0 | 0 | 0 |
| **OTU1798** | 2 | 0 | 5 | 0 | 0 | 0 | 0 | 0 | 0 |
| **OTU1799** | 10 | 22 | 31 | 0 | 2 | 3 | 0 | 0 | 0 |
| **OTU1800** | 0 | 1 | 7 | 0 | 0 | 0 | 0 | 0 | 0 |
| **OTU1801** | 0 | 0 | 1 | 21 | 11 | 16 | 18 | 12 | 11 |
| **OTU1802** | 0 | 0 | 0 | 0 | 5 | 0 | 0 | 0 | 0 |
| **OTU1803** | 5 | 1 | 1 | 0 | 0 | 0 | 0 | 0 | 0 |
| **OTU1804** | 91 | 92 | 149 | 0 | 23 | 23 | 1 | 0 | 0 |
| **OTU1805** | 52 | 70 | 126 | 0 | 6 | 7 | 0 | 2 | 1 |
| **OTU1806** | 1556 | 1042 | 1970 | 65 | 43 | 30 | 21 | 17 | 14 |
| **OTU1807** | 7 | 0 | 0 | 12 | 3 | 4 | 9 | 6 | 5 |
| **OTU1808** | 0 | 0 | 0 | 4 | 1 | 2 | 2 | 2 | 1 |
| **OTU1809** | 1 | 0 | 0 | 0 | 11 | 0 | 1 | 0 | 0 |
| **OTU1810** | 0 | 0 | 0 | 0 | 7 | 0 | 0 | 0 | 0 |
| **OTU1811** | 0 | 1 | 0 | 0 | 0 | 5 | 0 | 0 | 0 |
| **OTU1812** | 2265 | 2134 | 3165 | 40 | 293 | 172 | 24 | 24 | 15 |
| **OTU1813** | 0 | 1 | 0 | 0 | 4 | 0 | 0 | 0 | 0 |
| **OTU1814** | 4 | 8 | 14 | 0 | 0 | 2 | 0 | 0 | 0 |
| **OTU1815** | 11 | 11 | 19 | 416 | 304 | 154 | 396 | 143 | 186 |
| **OTU1816** | 0 | 3 | 2 | 0 | 0 | 0 | 0 | 0 | 0 |
| **OTU1817** | 155 | 217 | 263 | 3 | 8 | 5 | 0 | 5 | 1 |
| **OTU1818** | 3 | 3 | 7 | 0 | 0 | 2 | 0 | 0 | 1 |
| **OTU1819** | 0 | 0 | 0 | 4 | 0 | 0 | 0 | 0 | 0 |
| **OTU1820** | 34 | 67 | 100 | 0 | 8 | 11 | 0 | 0 | 0 |
| **OTU1821** | 4 | 0 | 1 | 2 | 4 | 3 | 1 | 3 | 1 |
| **OTU1822** | 51 | 63 | 58 | 0 | 12 | 7 | 0 | 0 | 0 |
| **OTU1823** | 3456 | 2515 | 4338 | 31 | 348 | 424 | 25 | 24 | 13 |
| **OTU1824** | 125 | 98 | 180 | 1030 | 412 | 478 | 570 | 413 | 297 |
| **OTU1825** | 0 | 0 | 0 | 6 | 3 | 3 | 3 | 3 | 0 |
| **OTU1826** | 0 | 0 | 0 | 62 | 1 | 0 | 0 | 0 | 2 |
| **OTU1827** | 21 | 12 | 27 | 594 | 413 | 307 | 430 | 361 | 255 |
| **OTU1828** | 24 | 15 | 51 | 1335 | 781 | 545 | 1035 | 460 | 634 |
| **OTU1829** | 3 | 7 | 11 | 0 | 0 | 1 | 0 | 0 | 1 |
| **OTU1830** | 70 | 42 | 87 | 1408 | 727 | 678 | 1306 | 741 | 668 |
| **OTU1831** | 1 | 0 | 1 | 8 | 6 | 7 | 12 | 7 | 6 |
| **OTU1832** | 0 | 1 | 7 | 0 | 0 | 0 | 0 | 0 | 0 |
| **OTU1833** | 9 | 5 | 19 | 69 | 35 | 34 | 83 | 21 | 7 |
| **OTU1834** | 1 | 0 | 0 | 9 | 5 | 7 | 14 | 8 | 7 |
| **OTU1835** | 134 | 158 | 242 | 0 | 44 | 45 | 1 | 0 | 0 |
| **OTU1836** | 48 | 60 | 78 | 0 | 12 | 7 | 0 | 0 | 0 |
| **OTU1837** | 2 | 1 | 1 | 181 | 24 | 5 | 8 | 3 | 14 |
| **OTU1838** | 12 | 9 | 25 | 0 | 2 | 1 | 0 | 1 | 0 |
| **OTU1839** | 225 | 177 | 279 | 0 | 133 | 114 | 0 | 0 | 0 |
| **OTU1840** | 76 | 100 | 110 | 1 | 31 | 26 | 0 | 1 | 0 |
| **OTU1841** | 1409 | 1064 | 2015 | 2 | 589 | 668 | 2 | 3 | 5 |
| **OTU1842** | 9 | 7 | 21 | 74 | 25 | 26 | 28 | 16 | 11 |
| **OTU1843** | 1 | 1 | 0 | 21 | 0 | 0 | 0 | 1 | 0 |
| **OTU1844** | 349 | 449 | 632 | 0 | 77 | 74 | 1 | 0 | 0 |
| **OTU1845** | 1 | 0 | 0 | 56 | 24 | 12 | 17 | 18 | 6 |
| **OTU1846** | 3 | 3 | 11 | 54 | 37 | 19 | 63 | 16 | 19 |
| **OTU1847** | 20 | 16 | 26 | 672 | 395 | 374 | 510 | 393 | 390 |
| **OTU1848** | 137 | 89 | 195 | 0 | 50 | 21 | 0 | 0 | 0 |
| **OTU1849** | 0 | 1 | 3 | 26 | 10 | 10 | 17 | 21 | 7 |
| **OTU1850** | 3 | 4 | 3 | 6 | 2 | 3 | 6 | 3 | 0 |
| **OTU1851** | 199 | 174 | 347 | 0 | 152 | 91 | 0 | 0 | 2 |
| **OTU1852** | 11 | 0 | 2 | 7 | 26 | 23 | 15 | 18 | 22 |
| **OTU1853** | 0 | 3 | 3 | 0 | 0 | 0 | 0 | 0 | 0 |
| **OTU1854** | 113 | 82 | 240 | 0 | 69 | 72 | 2 | 0 | 2 |
| **OTU1855** | 0 | 0 | 0 | 6 | 0 | 0 | 0 | 0 | 0 |
| **OTU1856** | 3 | 0 | 0 | 18 | 7 | 6 | 27 | 13 | 10 |
| **OTU1857** | 0 | 0 | 0 | 0 | 0 | 4 | 0 | 0 | 0 |
| **OTU1858** | 0 | 1 | 0 | 0 | 0 | 4 | 0 | 0 | 0 |
| **OTU1859** | 1 | 3 | 0 | 14 | 2 | 17 | 5 | 2 | 1 |
| **OTU1860** | 7 | 16 | 10 | 1 | 22 | 16 | 1 | 2 | 4 |
| **OTU1861** | 2 | 2 | 4 | 0 | 0 | 0 | 0 | 0 | 0 |
| **OTU1862** | 4 | 0 | 5 | 42 | 24 | 41 | 53 | 14 | 19 |
| **OTU1863** | 3 | 3 | 2 | 7 | 5 | 4 | 1 | 2 | 4 |
| **OTU1864** | 1 | 3 | 5 | 0 | 0 | 0 | 0 | 0 | 0 |
| **OTU1865** | 0 | 0 | 6 | 0 | 0 | 0 | 0 | 0 | 0 |
| **OTU1866** | 22 | 20 | 30 | 180 | 91 | 77 | 106 | 82 | 51 |
| **OTU1867** | 5 | 5 | 11 | 0 | 1 | 0 | 1 | 0 | 0 |
| **OTU1868** | 44 | 52 | 80 | 2883 | 1374 | 1164 | 2397 | 872 | 1252 |
| **OTU1869** | 0 | 0 | 0 | 0 | 6 | 0 | 0 | 0 | 0 |
| **OTU1870** | 29 | 29 | 77 | 35 | 63 | 76 | 28 | 43 | 25 |
| **OTU1871** | 250 | 282 | 375 | 22 | 74 | 47 | 14 | 9 | 12 |
| **OTU1872** | 5 | 1 | 8 | 0 | 0 | 0 | 0 | 0 | 0 |
| **OTU1873** | 274 | 199 | 356 | 5 | 35 | 55 | 4 | 2 | 3 |
| **OTU1874** | 2 | 4 | 0 | 0 | 0 | 0 | 0 | 0 | 0 |
| **OTU1875** | 3 | 4 | 7 | 141 | 65 | 58 | 82 | 71 | 54 |
| **OTU1876** | 2 | 3 | 9 | 0 | 0 | 2 | 0 | 0 | 0 |
| **OTU1877** | 111 | 89 | 155 | 0 | 42 | 25 | 1 | 0 | 0 |
| **OTU1878** | 0 | 0 | 0 | 6 | 3 | 0 | 4 | 1 | 2 |
| **OTU1879** | 1 | 0 | 0 | 0 | 4 | 0 | 0 | 0 | 0 |
| **OTU1880** | 337 | 291 | 674 | 2 | 411 | 316 | 0 | 0 | 0 |
| **OTU1881** | 5 | 6 | 5 | 68 | 51 | 47 | 58 | 57 | 62 |
| **OTU1882** | 87 | 58 | 199 | 0 | 39 | 38 | 0 | 0 | 0 |
| **OTU1883** | 193 | 167 | 403 | 2 | 108 | 107 | 0 | 0 | 0 |
| **OTU1884** | 0 | 0 | 2 | 38 | 14 | 10 | 21 | 10 | 11 |
| **OTU1885** | 92 | 44 | 146 | 0 | 31 | 28 | 0 | 0 | 0 |
| **OTU1886** | 0 | 3 | 4 | 0 | 0 | 5 | 0 | 0 | 0 |
| **OTU1887** | 4 | 4 | 6 | 102 | 42 | 42 | 48 | 25 | 32 |
| **OTU1888** | 92 | 117 | 218 | 0 | 41 | 49 | 1 | 0 | 0 |
| **OTU1889** | 77 | 75 | 84 | 0 | 12 | 13 | 1 | 0 | 0 |
| **OTU1890** | 0 | 0 | 0 | 3 | 4 | 0 | 0 | 2 | 1 |
| **OTU1891** | 10 | 10 | 15 | 0 | 11 | 1 | 0 | 0 | 0 |
| **OTU1892** | 0 | 1 | 0 | 63 | 25 | 19 | 25 | 17 | 13 |
| **OTU1893** | 12 | 5 | 37 | 190 | 92 | 84 | 107 | 62 | 52 |
| **OTU1894** | 29 | 20 | 37 | 3 | 2 | 1 | 2 | 0 | 2 |
| **OTU1895** | 142 | 106 | 189 | 0 | 56 | 41 | 0 | 0 | 1 |
| **OTU1896** | 112 | 97 | 161 | 0 | 61 | 36 | 1 | 0 | 2 |
| **OTU1897** | 7 | 13 | 25 | 0 | 2 | 5 | 0 | 0 | 0 |
| **OTU1898** | 4 | 2 | 7 | 183 | 111 | 111 | 74 | 89 | 69 |
| **OTU1899** | 0 | 0 | 1 | 83 | 25 | 28 | 29 | 24 | 14 |
| **OTU1900** | 1 | 3 | 7 | 1 | 0 | 0 | 0 | 0 | 0 |
| **OTU1901** | 0 | 1 | 1 | 5 | 1 | 0 | 4 | 3 | 4 |
| **OTU1902** | 8 | 1 | 6 | 0 | 4 | 2 | 0 | 0 | 0 |
| **OTU1903** | 175 | 122 | 235 | 4 | 37 | 26 | 6 | 2 | 5 |
| **OTU1904** | 187 | 238 | 241 | 2 | 10 | 2 | 0 | 0 | 3 |
| **OTU1905** | 13 | 13 | 17 | 65 | 24 | 20 | 37 | 18 | 23 |
| **OTU1906** | 1 | 0 | 5 | 46 | 14 | 16 | 25 | 15 | 15 |
| **OTU1907** | 5 | 1 | 0 | 0 | 0 | 0 | 0 | 0 | 0 |
| **OTU1908** | 0 | 1 | 0 | 0 | 0 | 0 | 18 | 0 | 0 |
| **OTU1909** | 27 | 22 | 30 | 0 | 3 | 6 | 0 | 0 | 0 |
| **OTU1910** | 30 | 31 | 59 | 1 | 10 | 2 | 0 | 0 | 0 |
| **OTU1911** | 6 | 2 | 1 | 18 | 10 | 16 | 28 | 20 | 16 |
| **OTU1912** | 12 | 9 | 24 | 2 | 1 | 1 | 0 | 0 | 1 |
| **OTU1913** | 98 | 45 | 107 | 1 | 13 | 19 | 0 | 1 | 1 |
| **OTU1914** | 18 | 11 | 18 | 28 | 42 | 49 | 8 | 7 | 7 |
| **OTU1915** | 2 | 4 | 5 | 51 | 13 | 18 | 27 | 24 | 13 |
| **OTU1916** | 22 | 15 | 24 | 422 | 367 | 302 | 464 | 210 | 135 |
| **OTU1917** | 7764 | 5700 | 10663 | 16 | 3712 | 3331 | 16 | 15 | 9 |
| **OTU1918** | 8 | 2 | 7 | 36 | 16 | 11 | 13 | 15 | 3 |
| **OTU1919** | 0 | 0 | 0 | 5 | 5 | 5 | 11 | 3 | 1 |
| **OTU1920** | 6 | 2 | 6 | 29 | 12 | 12 | 20 | 6 | 9 |
| **OTU1921** | 0 | 1 | 3 | 0 | 0 | 0 | 0 | 0 | 0 |
| **OTU1922** | 10 | 12 | 30 | 572 | 251 | 299 | 322 | 209 | 189 |
| **OTU1923** | 47 | 47 | 81 | 0 | 32 | 22 | 0 | 0 | 0 |
| **OTU1924** | 2 | 0 | 7 | 0 | 0 | 0 | 0 | 0 | 0 |
| **OTU1925** | 1 | 1 | 2 | 23 | 2 | 5 | 62 | 0 | 1 |
| **OTU1926** | 36 | 10 | 48 | 5 | 7 | 199 | 67 | 0 | 1 |
| **OTU1927** | 2 | 2 | 1 | 0 | 1 | 0 | 0 | 0 | 0 |
| **OTU1928** | 29301 | 19570 | 36088 | 289 | 3789 | 3385 | 186 | 194 | 144 |
| **OTU1929** | 5 | 4 | 6 | 202 | 81 | 90 | 57 | 90 | 43 |
| **OTU1930** | 116 | 129 | 260 | 0 | 50 | 18 | 0 | 0 | 0 |
| **OTU1931** | 15 | 31 | 55 | 121 | 278 | 242 | 145 | 226 | 255 |
| **OTU1932** | 138 | 78 | 152 | 1 | 81 | 68 | 0 | 1 | 2 |
| **OTU1933** | 16 | 22 | 40 | 0 | 23 | 8 | 0 | 0 | 0 |
| **OTU1934** | 9 | 0 | 0 | 0 | 0 | 0 | 0 | 0 | 0 |
| **OTU1935** | 415 | 472 | 635 | 1 | 95 | 118 | 0 | 2 | 2 |
| **OTU1936** | 0 | 0 | 0 | 5 | 0 | 0 | 0 | 0 | 0 |
| **OTU1937** | 431 | 317 | 687 | 0 | 182 | 137 | 3 | 0 | 2 |
| **OTU1938** | 1784 | 1359 | 2829 | 3 | 757 | 746 | 3 | 1 | 6 |
| **OTU1939** | 265 | 203 | 526 | 3512 | 7241 | 6075 | 4400 | 6502 | 6581 |
| **OTU1940** | 1 | 0 | 8 | 0 | 0 | 0 | 0 | 0 | 0 |
| **OTU1941** | 0 | 2 | 4 | 0 | 0 | 0 | 0 | 0 | 1 |
| **OTU1942** | 8 | 5 | 7 | 52 | 43 | 28 | 37 | 23 | 28 |
| **OTU1943** | 63 | 71 | 112 | 0 | 32 | 35 | 3 | 1 | 0 |
| **OTU1944** | 3 | 3 | 5 | 40 | 19 | 24 | 28 | 22 | 12 |
| **OTU1945** | 14 | 23 | 25 | 0 | 4 | 5 | 0 | 0 | 0 |
