# Supplementary material for: The Stain of the Original Salt: Red Heats on Chrome Tanned Leathers and Purple Spots on Ancient Parchments Are Two Sides of the Same Ecological Coin
Source: Front Microbiol. 2019 Oct 29;10:2459. doi: 10.3389/fmicb.2019.02459 (PMC6828845; doi:10.3389/fmicb.2019.02459)
Supplement: Supplementary file 3 [file Table_2.docx]

Supplementary Material

**Supplementary Table 2.** OTUs identification: a bootstrap cut off of 50% has been applied to unequivocally assign a taxonomic hierarchy to sequences; low taxonomic ranks (i.e. family, genus, species) have not yet been reported.

| **#OTU** | **DOMAIN** | **PHYLUM** | **CLASS/SUBCLASS** | **ORDER** |
| --- | --- | --- | --- | --- |
| OTU0001 | Bacteria | Proteobacteria | Gammaproteobacteria | Alteromonadales |
| OTU0002 | Bacteria | Firmicutes | Bacilli | Bacillales |
| OTU0003 | Archaea | Euryarchaeota | Halobacteria | Halobacteriales |
| OTU0004 | Bacteria | Firmicutes | Bacilli | Lactobacillales |
| OTU0005 | Archaea | [Parvarchaeota] | [Parvarchaea] | YLA114 |
| OTU0006 | Bacteria | Proteobacteria | Gammaproteobacteria | Vibrionales |
| OTU0007 | Bacteria | Proteobacteria | Alphaproteobacteria | Rhizobiales |
| OTU0008 | Archaea | [Parvarchaeota] | [Micrarchaea] | pMC2A384 |
| OTU0009 | Bacteria | Proteobacteria | Gammaproteobacteria | Vibrionales |
| OTU0010 | Bacteria | Proteobacteria | Gammaproteobacteria | Aeromonadales |
| OTU0011 | Archaea | Euryarchaeota | Halobacteria | Halobacteriales |
| OTU0012 | Bacteria | Proteobacteria | Gammaproteobacteria | Vibrionales |
| OTU0013 | Archaea | Euryarchaeota | Halobacteria | Halobacteriales |
| OTU0014 | Archaea | Euryarchaeota | Thermoplasmata | E2 |
| OTU0015 | Archaea | Euryarchaeota | Halobacteria | Halobacteriales |
| OTU0016 | Archaea | Euryarchaeota | Halobacteria | Halobacteriales |
| OTU0017 | Bacteria | Firmicutes | Bacilli | Bacillales |
| OTU0018 | Bacteria | Proteobacteria | Gammaproteobacteria | Alteromonadales |
| OTU0019 | Bacteria | Proteobacteria | Gammaproteobacteria | Vibrionales |
| OTU0020 | Bacteria | Proteobacteria | Gammaproteobacteria | Pseudomonadales |
| OTU0021 | Archaea | Euryarchaeota | Halobacteria | Halobacteriales |
| OTU0022 | Archaea | Euryarchaeota | DSEG | DHVE3 |
| OTU0023 | Bacteria | Proteobacteria | Gammaproteobacteria | Pseudomonadales |
| OTU0024 | Bacteria | Proteobacteria | Gammaproteobacteria | Oceanospirillales |
| OTU0025 | Bacteria | Proteobacteria | Gammaproteobacteria | Pseudomonadales |
| OTU0026 | Bacteria | Proteobacteria | Gammaproteobacteria | Pseudomonadales |
| OTU0027 | Bacteria | Proteobacteria | Betaproteobacteria | Burkholderiales |
| OTU0028 | Archaea | Euryarchaeota | Halobacteria | Halobacteriales |
| OTU0029 | Bacteria | Firmicutes | Bacilli | Bacillales |
| OTU0030 | Bacteria | Proteobacteria | Gammaproteobacteria | Vibrionales |
| OTU0031 | Bacteria | Proteobacteria | Gammaproteobacteria | Pseudomonadales |
| OTU0032 | Bacteria | Proteobacteria | Gammaproteobacteria | Enterobacteriales |
| OTU0033 | Bacteria | Bacteroidetes | [Rhodothermi] | [Rhodothermales] |
| OTU0034 | Bacteria | Proteobacteria | Gammaproteobacteria | Alteromonadales |
| OTU0035 | Archaea | Euryarchaeota | Halobacteria | Halobacteriales |
| OTU0036 | Bacteria | Proteobacteria | Gammaproteobacteria | Oceanospirillales |
| OTU0037 | Archaea | Euryarchaeota | Halobacteria | Halobacteriales |
| OTU0038 | Archaea | Euryarchaeota | Halobacteria | Halobacteriales |
| OTU0039 | Bacteria | Proteobacteria | Gammaproteobacteria | Vibrionales |
| OTU0040 | Bacteria | Proteobacteria | Gammaproteobacteria | Aeromonadales |
| OTU0041 | Bacteria | Proteobacteria | Gammaproteobacteria | Vibrionales |
| OTU0042 | Archaea | Euryarchaeota | Halobacteria | Halobacteriales |
| OTU0043 | Bacteria | Proteobacteria | Gammaproteobacteria | Pseudomonadales |
| OTU0044 | Bacteria | Firmicutes | Bacilli | Bacillales |
| OTU0045 | Bacteria | Proteobacteria | Gammaproteobacteria | Vibrionales |
| OTU0046 | Archaea | Euryarchaeota | Halobacteria | Halobacteriales |
| OTU0047 | Archaea | Euryarchaeota | Halobacteria | Halobacteriales |
| OTU0048 | Bacteria | Proteobacteria | Gammaproteobacteria | Pseudomonadales |
| OTU0049 | Bacteria | Proteobacteria | Gammaproteobacteria | Salinisphaerales |
| OTU0050 | Archaea | Euryarchaeota | Methanobacteria | Methanobacteriales |
| OTU0051 | Archaea | Euryarchaeota | Thermoplasmata | E2 |
| OTU0052 | Bacteria | Proteobacteria | Gammaproteobacteria | Pseudomonadales |
| OTU0053 | Archaea |  |  |  |
| OTU0054 | Bacteria | Proteobacteria | Gammaproteobacteria | Pseudomonadales |
| OTU0055 | Archaea | Euryarchaeota | Thermoplasmata | E2 |
| OTU0056 | Bacteria | Actinobacteria | Actinobacteria | Corynebacteriales |
| OTU0057 | Bacteria | Proteobacteria | Gammaproteobacteria | Vibrionales |
| OTU0058 | Bacteria | Proteobacteria | Gammaproteobacteria | Pseudomonadales |
| OTU0059 | Bacteria | Firmicutes | Bacilli | Bacillales |
| OTU0060 | Bacteria | Proteobacteria | Gammaproteobacteria | Vibrionales |
| OTU0061 | Archaea | Euryarchaeota | Halobacteria | Halobacteriales |
| OTU0062 | Bacteria | Proteobacteria | Gammaproteobacteria | Vibrionales |
| OTU0063 | Bacteria | Proteobacteria | Gammaproteobacteria | Pseudomonadales |
| OTU0064 | Bacteria | Proteobacteria | Gammaproteobacteria | Oceanospirillales |
| OTU0065 | Bacteria | Firmicutes | Bacilli |  |
| OTU0066 | Bacteria | Proteobacteria | Gammaproteobacteria | Oceanospirillales |
| OTU0067 | Bacteria | Proteobacteria | Gammaproteobacteria | Enterobacteriales |
| OTU0068 | Bacteria | Firmicutes | Bacilli | Lactobacillales |
| OTU0069 | Bacteria | Proteobacteria | Alphaproteobacteria | Rhizobiales |
| OTU0070 | Bacteria | Firmicutes | Bacilli | Lactobacillales |
| OTU0071 | Archaea | Euryarchaeota | Halobacteria | Halobacteriales |
| OTU0072 | Archaea | Euryarchaeota | Halobacteria | Halobacteriales |
| OTU0073 | Bacteria | Actinobacteria | Actinobacteria | Corynebacteriales |
| OTU0074 | Bacteria | Proteobacteria | Gammaproteobacteria | Pseudomonadales |
| OTU0075 | Archaea | Euryarchaeota | Halobacteria | Halobacteriales |
| OTU0076 | Bacteria | Proteobacteria | Gammaproteobacteria | Pseudomonadales |
| OTU0077 | Archaea | Euryarchaeota | Halobacteria | Halobacteriales |
| OTU0078 | Bacteria | Proteobacteria | Gammaproteobacteria | Pseudomonadales |
| OTU0079 | Bacteria | Firmicutes | Bacilli | Bacillales |
| OTU0080 | Bacteria | Firmicutes | Bacilli | Bacillales |
| OTU0081 | Bacteria | Proteobacteria | Gammaproteobacteria | Vibrionales |
| OTU0082 | Bacteria | Firmicutes | Bacilli | Bacillales |
| OTU0083 | Bacteria | Proteobacteria | Alphaproteobacteria | Kiloniellales |
| OTU0084 | Archaea | Euryarchaeota | Halobacteria | Halobacteriales |
| OTU0085 | Bacteria | Proteobacteria | Gammaproteobacteria | Pseudomonadales |
| OTU0086 | Bacteria | Proteobacteria | Gammaproteobacteria | Salinisphaerales |
| OTU0087 | Archaea | Euryarchaeota | Methanobacteria | Methanobacteriales |
| OTU0088 | Bacteria | Proteobacteria | Gammaproteobacteria | Oceanospirillales |
| OTU0089 | Bacteria | Proteobacteria | Gammaproteobacteria | Enterobacteriales |
| OTU0090 | Bacteria | Proteobacteria | Gammaproteobacteria | Pseudomonadales |
| OTU0091 | Bacteria | Proteobacteria | Gammaproteobacteria | Alteromonadales |
| OTU0092 | Bacteria | Proteobacteria | Alphaproteobacteria | Sphingomonadales |
| OTU0093 | Archaea | Euryarchaeota | Halobacteria | Halobacteriales |
| OTU0094 | Bacteria | Firmicutes | Bacilli | Lactobacillales |
| OTU0095 | Bacteria | Proteobacteria | Gammaproteobacteria | Alteromonadales |
| OTU0096 | Archaea | Euryarchaeota | Halobacteria | Halobacteriales |
| OTU0097 | Archaea | Euryarchaeota | Halobacteria | Halobacteriales |
| OTU0098 | Bacteria | Firmicutes | Bacilli | Lactobacillales |
| OTU0099 | Bacteria | Proteobacteria | Gammaproteobacteria | Pseudomonadales |
| OTU0100 | Bacteria | Actinobacteria | Actinobacteria | Propionibacterales |
| OTU0101 | Bacteria | Firmicutes | Bacilli | Bacillales |
| OTU0102 | Bacteria | Actinobacteria | Actinobacteria | Corynebacteriales |
| OTU0103 | Bacteria | Proteobacteria | Gammaproteobacteria | Enterobacteriales |
| OTU0104 | Archaea | Euryarchaeota | Halobacteria | Halobacteriales |
| OTU0105 | Bacteria | Proteobacteria | Alphaproteobacteria | Sphingomonadales |
| OTU0106 | Bacteria | Firmicutes | Bacilli | Lactobacillales |
| OTU0107 | Bacteria | Proteobacteria | Gammaproteobacteria | Oceanospirillales |
| OTU0108 | Bacteria | Proteobacteria | Gammaproteobacteria | Vibrionales |
| OTU0109 | Bacteria | Proteobacteria | Gammaproteobacteria | Alteromonadales |
| OTU0110 | Bacteria | Proteobacteria | Gammaproteobacteria | Oceanospirillales |
| OTU0111 | Bacteria | Proteobacteria | Gammaproteobacteria | Alteromonadales |
| OTU0112 | Bacteria | Proteobacteria | Betaproteobacteria | Burkholderiales |
| OTU0113 | Bacteria | Proteobacteria | Gammaproteobacteria | Vibrionales |
| OTU0114 | Bacteria | Firmicutes | Bacilli | Lactobacillales |
| OTU0115 | Bacteria | Actinobacteria | Actinobacteria | Micrococcales |
| OTU0116 | Bacteria | Proteobacteria | Gammaproteobacteria | Vibrionales |
| OTU0117 | Bacteria | Proteobacteria | Gammaproteobacteria | Vibrionales |
| OTU0118 | Bacteria | Proteobacteria | Gammaproteobacteria | Vibrionales |
| OTU0119 | Bacteria | Proteobacteria | Gammaproteobacteria | Alteromonadales |
| OTU0120 | Bacteria | Proteobacteria | Gammaproteobacteria | Oceanospirillales |
| OTU0121 | Bacteria | Fusobacteria | Fusobacteriia | Fusobacteriales |
| OTU0122 | Bacteria | Proteobacteria | Betaproteobacteria | Burkholderiales |
| OTU0123 | Bacteria | Firmicutes | Bacilli | Bacillales |
| OTU0124 | Bacteria | Firmicutes | Clostridia | Clostridiales |
| OTU0125 | Bacteria | Firmicutes | Bacilli | Lactobacillales |
| OTU0126 | Bacteria | Proteobacteria | Betaproteobacteria | Neisseriales |
| OTU0127 | Archaea | Euryarchaeota | Methanobacteria | Methanobacteriales |
| OTU0128 | Archaea | Euryarchaeota | Methanobacteria | Methanobacteriales |
| OTU0129 | Archaea | Euryarchaeota | Halobacteria | Halobacteriales |
| OTU0130 | Bacteria | Proteobacteria | Gammaproteobacteria | Aeromonadales |
| OTU0131 | Bacteria | Proteobacteria |  |  |
| OTU0132 | Archaea | Euryarchaeota | Halobacteria | Halobacteriales |
| OTU0133 | Bacteria | Proteobacteria | Gammaproteobacteria | Alteromonadales |
| OTU0134 | Bacteria | Bacteroidetes | Sphingobacteriia | Sphingobacteriales |
| OTU0135 | Bacteria | Firmicutes | Bacilli | Bacillales |
| OTU0136 | Bacteria | Proteobacteria | Alphaproteobacteria | Rhodospirillales |
| OTU0137 | Bacteria | Proteobacteria | Gammaproteobacteria | Alteromonadales |
| OTU0138 | Bacteria | Firmicutes | Bacilli | Bacillales |
| OTU0139 | Archaea | Euryarchaeota | Methanobacteria | Methanobacteriales |
| OTU0140 | Bacteria | Proteobacteria | Gammaproteobacteria | Alteromonadales |
| OTU0141 | Bacteria | Actinobacteria | Actinobacteria | Nocardiales |
| OTU0142 | Bacteria | Firmicutes | Bacilli | Lactobacillales |
| OTU0143 | Bacteria | Proteobacteria | Gammaproteobacteria | Vibrionales |
| OTU0144 | Bacteria | Proteobacteria | Gammaproteobacteria | Pseudomonadales |
| OTU0145 | Bacteria | Firmicutes | Clostridia | Halanaerobiales |
| OTU0146 | Bacteria | Proteobacteria | Gammaproteobacteria | Chromatiales |
| OTU0147 | Bacteria | Firmicutes | Bacilli | Bacillales |
| OTU0148 | Bacteria | Firmicutes | Bacilli | Bacillales |
| OTU0149 | Bacteria | Proteobacteria | Gammaproteobacteria | Pseudomonadales |
| OTU0150 | Bacteria | Firmicutes | Bacilli | Gemellales |
| OTU0151 | Archaea | Euryarchaeota | Methanobacteria | Methanobacteriales |
| OTU0152 | Bacteria | Proteobacteria | Gammaproteobacteria | Vibrionales |
| OTU0153 | Archaea | Euryarchaeota | Halobacteria | Halobacteriales |
| OTU0154 | Bacteria | Firmicutes | Bacilli | Bacillales |
| OTU0155 | Bacteria | Firmicutes | Bacilli | Lactobacillales |
| OTU0156 | Bacteria | Actinobacteria | Actinobacteria | Corynebacteriales |
| OTU0157 | Bacteria | Proteobacteria | Gammaproteobacteria | Aeromonadales |
| OTU0158 | Archaea | Euryarchaeota | Halobacteria | Halobacteriales |
| OTU0159 | Bacteria | Firmicutes | Bacilli | Bacillales |
| OTU0160 | Bacteria | Firmicutes | Bacilli | Bacillales |
| OTU0161 | Bacteria | Firmicutes | Bacilli | Bacillales |
| OTU0162 | Bacteria | Firmicutes | Bacilli | Turicibacterales |
| OTU0163 | Bacteria | Proteobacteria | Gammaproteobacteria | Pseudomonadales |
| OTU0164 | Bacteria | Firmicutes | Bacilli | Bacillales |
| OTU0165 | Bacteria | Proteobacteria | Gammaproteobacteria | Alteromonadales |
| OTU0166 | Bacteria | Firmicutes | Bacilli | Bacillales |
| OTU0167 | Bacteria | Proteobacteria | Gammaproteobacteria | Vibrionales |
| OTU0168 | Bacteria | Actinobacteria | Actinobacteria | Micrococcales |
| OTU0169 | Bacteria | Firmicutes | Bacilli | Bacillales |
| OTU0170 | Archaea | Euryarchaeota | Halobacteria | Halobacteriales |
| OTU0171 | Bacteria | Firmicutes | Bacilli | Bacillales |
| OTU0172 | Archaea | Euryarchaeota | Halobacteria | Halobacteriales |
| OTU0173 | Bacteria | Firmicutes | Bacilli | Bacillales |
| OTU0174 | Bacteria | Firmicutes | Bacilli | Lactobacillales |
| OTU0175 | Bacteria | Firmicutes | Bacilli |  |
| OTU0176 | Archaea | Euryarchaeota | Halobacteria | Halobacteriales |
| OTU0177 | Archaea | Euryarchaeota | Halobacteria | Halobacteriales |
| OTU0178 | Archaea | Euryarchaeota | Methanobacteria | Methanobacteriales |
| OTU0179 | Bacteria | Firmicutes | Bacilli | Bacillales |
| OTU0180 | Archaea | Euryarchaeota | Halobacteria | Halobacteriales |
| OTU0181 | Archaea | Euryarchaeota | Halobacteria | Halobacteriales |
| OTU0182 | Archaea | Euryarchaeota | Halobacteria | Halobacteriales |
| OTU0183 | Bacteria | Proteobacteria | Gammaproteobacteria | Alteromonadales |
| OTU0184 | Archaea | Euryarchaeota | Halobacteria | Halobacteriales |
| OTU0185 | Bacteria | [Thermi] | Deinococci | Thermales |
| OTU0186 | Archaea | Euryarchaeota | Halobacteria | Halobacteriales |
| OTU0187 | Bacteria | Firmicutes | Bacilli | Bacillales |
| OTU0188 | Bacteria | Proteobacteria | Betaproteobacteria | Burkholderiales |
| OTU0189 | Bacteria |  |  |  |
| OTU0190 | Bacteria | Firmicutes | Clostridia | Clostridiales |
| OTU0191 | Archaea | Euryarchaeota | Halobacteria | Halobacteriales |
| OTU0192 | Bacteria | Proteobacteria | Gammaproteobacteria | Vibrionales |
| OTU0193 | Archaea | Euryarchaeota | Halobacteria | Halobacteriales |
| OTU0194 | Bacteria | Proteobacteria | Gammaproteobacteria | Vibrionales |
| OTU0195 | Archaea | Euryarchaeota | Halobacteria | Halobacteriales |
| OTU0196 | Bacteria | Proteobacteria | Gammaproteobacteria | Pseudomonadales |
| OTU0197 | Archaea | Euryarchaeota | Halobacteria | Halobacteriales |
| OTU0198 | Archaea | Euryarchaeota | Halobacteria | Halobacteriales |
| OTU0199 | Bacteria | Proteobacteria | Gammaproteobacteria | Vibrionales |
| OTU0200 | Archaea | Euryarchaeota | Halobacteria | Halobacteriales |
| OTU0201 | Bacteria | Proteobacteria | Gammaproteobacteria | Alteromonadales |
| OTU0202 | Archaea | Euryarchaeota | Halobacteria | Halobacteriales |
| OTU0203 | Bacteria | Firmicutes | Bacilli | Lactobacillales |
| OTU0204 | Archaea | Euryarchaeota | Halobacteria | Halobacteriales |
| OTU0205 | Bacteria | Actinobacteria | Actinobacteria | Micrococcales |
| OTU0206 | Archaea | Euryarchaeota | Halobacteria | Halobacteriales |
| OTU0207 | Bacteria | Actinobacteria | Actinobacteria | Corynebacteriales |
| OTU0208 | Archaea | Euryarchaeota | Halobacteria | Halobacteriales |
| OTU0209 | Bacteria | Firmicutes | Bacilli | Lactobacillales |
| OTU0210 | Archaea | Euryarchaeota | Halobacteria | Halobacteriales |
| OTU0211 | Bacteria | Proteobacteria | Gammaproteobacteria | Chromatiales |
| OTU0212 | Bacteria | Firmicutes | Bacilli | Bacillales |
| OTU0213 | Archaea | Euryarchaeota | Halobacteria | Halobacteriales |
| OTU0214 | Bacteria | Proteobacteria | Gammaproteobacteria | Vibrionales |
| OTU0215 | Bacteria | Proteobacteria | Gammaproteobacteria | Alteromonadales |
| OTU0216 | Bacteria | Proteobacteria | Gammaproteobacteria | Oceanospirillales |
| OTU0217 | Archaea | Euryarchaeota | Halobacteria | Halobacteriales |
| OTU0218 | Archaea | Euryarchaeota | Halobacteria | Halobacteriales |
| OTU0219 | Archaea | Euryarchaeota | Halobacteria | Halobacteriales |
| OTU0220 | Bacteria | Proteobacteria | Betaproteobacteria | Burkholderiales |
| OTU0221 | Archaea | Euryarchaeota | Halobacteria | Halobacteriales |
| OTU0222 | Bacteria | Proteobacteria | Gammaproteobacteria | Oceanospirillales |
| OTU0223 | Archaea | Euryarchaeota | Halobacteria | Halobacteriales |
| OTU0224 | Bacteria | Proteobacteria | Gammaproteobacteria | Pseudomonadales |
| OTU0225 | Bacteria | Proteobacteria | Betaproteobacteria | Burkholderiales |
| OTU0226 | Bacteria | Bacteroidetes | Flavobacteriia | Flavobacteriales |
| OTU0227 | Bacteria | Proteobacteria | Gammaproteobacteria | Oceanospirillales |
| OTU0228 | Archaea | Euryarchaeota | Halobacteria | Halobacteriales |
| OTU0229 | Bacteria | Proteobacteria | Gammaproteobacteria | Alteromonadales |
| OTU0230 | Bacteria | Proteobacteria | Gammaproteobacteria | Vibrionales |
| OTU0231 | Archaea | Euryarchaeota | Halobacteria | Halobacteriales |
| OTU0232 | Bacteria | Proteobacteria | Alphaproteobacteria | Rhodobacterales |
| OTU0233 | Bacteria | Proteobacteria | Alphaproteobacteria | Rhodobacterales |
| OTU0234 | Bacteria | Proteobacteria | Gammaproteobacteria | Pseudomonadales |
| OTU0235 | Bacteria | Proteobacteria | Gammaproteobacteria | Alteromonadales |
| OTU0236 | Archaea | Euryarchaeota | Methanobacteria | Methanobacteriales |
| OTU0237 | Bacteria | Bacteroidetes | Flavobacteriia | Flavobacteriales |
| OTU0238 | Bacteria | Firmicutes | Bacilli | Lactobacillales |
| OTU0239 | Bacteria | Proteobacteria | Alphaproteobacteria | Rhodobacterales |
| OTU0240 | Archaea | Euryarchaeota | Halobacteria | Halobacteriales |
| OTU0241 | Archaea | Euryarchaeota | Halobacteria | Halobacteriales |
| OTU0242 | Bacteria | Firmicutes | Bacilli | Bacillales |
| OTU0243 | Bacteria | Firmicutes | Bacilli | Bacillales |
| OTU0244 | Archaea | Euryarchaeota | Halobacteria | Halobacteriales |
| OTU0245 | Archaea | Euryarchaeota | Methanobacteria | Methanobacteriales |
| OTU0246 | Bacteria | Firmicutes | Bacilli | Lactobacillales |
| OTU0247 | Bacteria | Proteobacteria | Gammaproteobacteria | Enterobacteriales |
| OTU0248 | Archaea | Euryarchaeota | Halobacteria | Halobacteriales |
| OTU0249 | Archaea | Euryarchaeota | Methanobacteria | Methanobacteriales |
| OTU0250 | Bacteria | Firmicutes | Bacilli | Bacillales |
| OTU0251 | Archaea | Euryarchaeota | Halobacteria | Halobacteriales |
| OTU0252 | Archaea | Euryarchaeota | Halobacteria | Halobacteriales |
| OTU0253 | Archaea | Euryarchaeota | Halobacteria | Halobacteriales |
| OTU0254 | Bacteria | Proteobacteria | Gammaproteobacteria | Vibrionales |
| OTU0255 | Bacteria | Firmicutes | Bacilli | Bacillales |
| OTU0256 | Bacteria | Proteobacteria | Gammaproteobacteria |  |
| OTU0257 | Bacteria | Proteobacteria | Gammaproteobacteria |  |
| OTU0258 | Bacteria | Firmicutes | Bacilli | Lactobacillales |
| OTU0259 | Bacteria | Proteobacteria | Gammaproteobacteria | Vibrionales |
| OTU0260 | Bacteria | Actinobacteria | Actinobacteria | Micrococcales |
| OTU0261 | Bacteria | Proteobacteria | Gammaproteobacteria | Vibrionales |
| OTU0262 | Archaea | Euryarchaeota | Halobacteria | Halobacteriales |
| OTU0263 | Archaea | Euryarchaeota | Halobacteria | Halobacteriales |
| OTU0264 | Archaea | Euryarchaeota | Halobacteria | Halobacteriales |
| OTU0265 | Archaea | Euryarchaeota | Halobacteria | Halobacteriales |
| OTU0266 | Bacteria | Proteobacteria | Deltaproteobacteria | Desulfobacterales |
| OTU0267 | Bacteria | Firmicutes | Clostridia | Clostridiales |
| OTU0268 | Bacteria | Proteobacteria | Gammaproteobacteria | Xanthomonadales |
| OTU0269 | Archaea | Euryarchaeota | Halobacteria | Halobacteriales |
| OTU0270 | Archaea | Euryarchaeota | Halobacteria | Halobacteriales |
| OTU0271 | Bacteria | Proteobacteria | Gammaproteobacteria | Alteromonadales |
| OTU0272 | Bacteria | Firmicutes | Bacilli | Lactobacillales |
| OTU0273 | Bacteria | Firmicutes | Bacilli | Bacillales |
| OTU0274 | Bacteria | Proteobacteria | Alphaproteobacteria | Sphingomonadales |
| OTU0275 | Bacteria | Proteobacteria | Gammaproteobacteria | Pasteurellales |
| OTU0276 | Bacteria | Proteobacteria | Gammaproteobacteria | Aeromonadales |
| OTU0277 | Bacteria | Proteobacteria | Alphaproteobacteria | Rhodospirillales |
| OTU0278 | Bacteria | [Thermi] | Deinococci | Deinococcales |
| OTU0279 | Archaea | Euryarchaeota | Halobacteria | Halobacteriales |
| OTU0280 | Bacteria | Firmicutes | Bacilli | Bacillales |
| OTU0281 | Archaea | Euryarchaeota | Halobacteria | Halobacteriales |
| OTU0282 | Bacteria | Actinobacteria | Actinobacteria | Corynebacteriales |
| OTU0283 | Archaea | Euryarchaeota | Halobacteria | Halobacteriales |
| OTU0284 | Bacteria | Proteobacteria | Gammaproteobacteria | Pseudomonadales |
| OTU0285 | Bacteria | Firmicutes | Bacilli | Lactobacillales |
| OTU0286 | Archaea | Euryarchaeota | Halobacteria | Halobacteriales |
| OTU0287 | Bacteria | Proteobacteria | Gammaproteobacteria | Aeromonadales |
| OTU0288 | Bacteria | Proteobacteria | Alphaproteobacteria | Caulobacterales |
| OTU0289 | Archaea | Euryarchaeota | Halobacteria | Halobacteriales |
| OTU0290 | Archaea | Euryarchaeota | Halobacteria | Halobacteriales |
| OTU0291 | Archaea | Euryarchaeota | Halobacteria | Halobacteriales |
| OTU0292 | Bacteria | Proteobacteria | Gammaproteobacteria | Alteromonadales |
| OTU0293 | Archaea | Euryarchaeota | Halobacteria | Halobacteriales |
| OTU0294 | Archaea | Euryarchaeota | Halobacteria | Halobacteriales |
| OTU0295 | Archaea | Euryarchaeota | Methanomicrobia | Methanomicrobiales |
| OTU0296 | Bacteria | Proteobacteria | Gammaproteobacteria | Vibrionales |
| OTU0297 | Bacteria | Proteobacteria | Deltaproteobacteria | Myxococcales |
| OTU0298 | Bacteria | Proteobacteria | Alphaproteobacteria | Rhizobiales |
| OTU0299 | Bacteria | Proteobacteria | Alphaproteobacteria | Rhizobiales |
| OTU0300 | Bacteria | Firmicutes | Bacilli | Lactobacillales |
| OTU0301 | Bacteria | Proteobacteria | Gammaproteobacteria | Alteromonadales |
| OTU0302 | Bacteria | Proteobacteria | Gammaproteobacteria | Pasteurellales |
| OTU0303 | Bacteria | Firmicutes | Clostridia | Clostridiales |
| OTU0304 | Bacteria | Proteobacteria | Gammaproteobacteria | Vibrionales |
| OTU0305 | Bacteria | Proteobacteria | Gammaproteobacteria | Vibrionales |
| OTU0306 | Bacteria | Firmicutes | Bacilli | Bacillales |
| OTU0307 | Bacteria | Bacteroidetes | Sphingobacteriia | Sphingobacteriales |
| OTU0308 | Bacteria | Proteobacteria | Betaproteobacteria | Burkholderiales |
| OTU0309 | Bacteria | Proteobacteria | Gammaproteobacteria | Oceanospirillales |
| OTU0310 | Bacteria | Proteobacteria | Gammaproteobacteria | Vibrionales |
| OTU0311 | Bacteria | Actinobacteria | Actinobacteria | Corynebacteriales |
| OTU0312 | Archaea | Euryarchaeota | Halobacteria | Halobacteriales |
| OTU0313 | Bacteria | Proteobacteria | Gammaproteobacteria | Oceanospirillales |
| OTU0314 | Bacteria | Proteobacteria | Gammaproteobacteria | Alteromonadales |
| OTU0315 | Archaea | Euryarchaeota | Halobacteria | Halobacteriales |
| OTU0316 | Bacteria | Firmicutes | Clostridia | Clostridiales |
| OTU0317 | Archaea | Euryarchaeota | Halobacteria | Halobacteriales |
| OTU0318 | Bacteria | Proteobacteria | Alphaproteobacteria | Rhizobiales |
| OTU0319 | Bacteria | Proteobacteria | Alphaproteobacteria | Rhodobacterales |
| OTU0320 | Archaea | Euryarchaeota | Halobacteria | Halobacteriales |
| OTU0321 | Archaea | Euryarchaeota | Halobacteria | Halobacteriales |
| OTU0322 | Archaea | Euryarchaeota | Halobacteria | Halobacteriales |
| OTU0323 | Bacteria | Proteobacteria | Alphaproteobacteria | Rhizobiales |
| OTU0324 | Bacteria | Proteobacteria | Gammaproteobacteria | Alteromonadales |
| OTU0325 | Bacteria | Firmicutes | Bacilli | Bacillales |
| OTU0326 | Archaea | Euryarchaeota | Methanomicrobia | Methanomicrobiales |
| OTU0327 | Archaea | Euryarchaeota | Halobacteria | Halobacteriales |
| OTU0328 | Bacteria | Firmicutes | Bacilli | Bacillales |
| OTU0329 | Bacteria | Firmicutes | Bacilli |  |
| OTU0330 | Bacteria | Proteobacteria | Alphaproteobacteria | Sphingomonadales |
| OTU0331 | Archaea | Euryarchaeota | Halobacteria | Halobacteriales |
| OTU0332 | Bacteria | Proteobacteria | Gammaproteobacteria | Alteromonadales |
| OTU0333 | Bacteria | Firmicutes | Clostridia | Clostridiales |
| OTU0334 | Bacteria | Proteobacteria | Gammaproteobacteria | Vibrionales |
| OTU0335 | Bacteria | Proteobacteria | Gammaproteobacteria | Pseudomonadales |
| OTU0336 | Archaea | Euryarchaeota | Halobacteria | Halobacteriales |
| OTU0337 | Bacteria | Proteobacteria | Alphaproteobacteria | Rhodobacterales |
| OTU0338 | Bacteria | Proteobacteria | Betaproteobacteria | Burkholderiales |
| OTU0339 | Archaea | Euryarchaeota | Halobacteria | Halobacteriales |
| OTU0340 | Archaea | Euryarchaeota | Methanomicrobia | Methanomicrobiales |
| OTU0341 | Archaea | Euryarchaeota | Halobacteria | Halobacteriales |
| OTU0342 | Bacteria | Proteobacteria | Gammaproteobacteria | Pseudomonadales |
| OTU0343 | Archaea | Euryarchaeota | Halobacteria | Halobacteriales |
| OTU0344 | Archaea | Euryarchaeota | Halobacteria | Halobacteriales |
| OTU0345 | Bacteria | Bacteroidetes | Cytophagia | Cytophagales |
| OTU0346 | Bacteria | Firmicutes | Clostridia | Halanaerobiales |
| OTU0347 | Bacteria | Firmicutes | Bacilli | Lactobacillales |
| OTU0348 | Archaea | Euryarchaeota | Halobacteria | Halobacteriales |
| OTU0349 | Bacteria | Proteobacteria | Gammaproteobacteria | Xanthomonadales |
| OTU0350 | Bacteria | Proteobacteria | Gammaproteobacteria | Alteromonadales |
| OTU0351 | Archaea | Euryarchaeota | Halobacteria | Halobacteriales |
| OTU0352 | Bacteria | Proteobacteria | Gammaproteobacteria | Pseudomonadales |
| OTU0353 | Bacteria | Proteobacteria | Gammaproteobacteria | Vibrionales |
| OTU0354 | Bacteria | Firmicutes | Clostridia | Halanaerobiales |
| OTU0355 | Archaea | Euryarchaeota | Halobacteria | Halobacteriales |
| OTU0356 | Archaea | Euryarchaeota | Halobacteria | Halobacteriales |
| OTU0357 | Bacteria | Proteobacteria | Gammaproteobacteria |  |
| OTU0358 | Bacteria | Proteobacteria | Gammaproteobacteria | Pseudomonadales |
| OTU0359 | Bacteria | Firmicutes | Bacilli | Lactobacillales |
| OTU0360 | Bacteria | Proteobacteria | Gammaproteobacteria | Pseudomonadales |
| OTU0361 | Bacteria | Firmicutes | Bacilli | Bacillales |
| OTU0362 | Bacteria | Proteobacteria | Alphaproteobacteria | Rhizobiales |
| OTU0363 | Archaea |  |  |  |
| OTU0364 | Bacteria | Actinobacteria | Actinobacteria | Pseudonocardiales |
| OTU0365 | Bacteria | Proteobacteria | Gammaproteobacteria | Alteromonadales |
| OTU0366 | Bacteria | Firmicutes | Bacilli | Bacillales |
| OTU0367 | Archaea | Euryarchaeota | Halobacteria | Halobacteriales |
| OTU0368 | Bacteria | Proteobacteria | Gammaproteobacteria |  |
| OTU0369 | Bacteria | Proteobacteria | Alphaproteobacteria | Rhizobiales |
| OTU0370 | Bacteria | Proteobacteria | Gammaproteobacteria | Pseudomonadales |
| OTU0371 | Archaea | Euryarchaeota | Halobacteria | Halobacteriales |
| OTU0372 | Bacteria | Proteobacteria | Gammaproteobacteria | Vibrionales |
| OTU0373 | Archaea | Euryarchaeota | Halobacteria | Halobacteriales |
| OTU0374 | Bacteria | Proteobacteria | Gammaproteobacteria | Thiotrichales |
| OTU0375 | Bacteria | Proteobacteria | Epsilonproteobacteria | Campylobacterales |
| OTU0376 | Bacteria | Proteobacteria | Gammaproteobacteria | Pseudomonadales |
| OTU0377 | Archaea | Euryarchaeota | Halobacteria | Halobacteriales |
| OTU0378 | Bacteria | Firmicutes | Bacilli | Bacillales |
| OTU0379 | Bacteria | Firmicutes | Bacilli | Bacillales |
| OTU0380 | Archaea | Euryarchaeota | Halobacteria | Halobacteriales |
| OTU0381 | Archaea | Euryarchaeota | Halobacteria | Halobacteriales |
| OTU0382 | Bacteria | Proteobacteria | Gammaproteobacteria | Alteromonadales |
| OTU0383 | Bacteria | Proteobacteria | Alphaproteobacteria | Rhodobacterales |
| OTU0384 | Bacteria | Proteobacteria | Gammaproteobacteria | Xanthomonadales |
| OTU0385 | Bacteria | Bacteroidetes | Bacteroidia | Bacteroidales |
| OTU0386 | Bacteria | Actinobacteria | Actinobacteria | Corynebacteriales |
| OTU0387 | Bacteria | Firmicutes | Bacilli | Lactobacillales |
| OTU0388 | Bacteria | Proteobacteria | Gammaproteobacteria | Xanthomonadales |
| OTU0389 | Bacteria | Proteobacteria | Betaproteobacteria | Neisseriales |
| OTU0390 | Bacteria | Proteobacteria | Gammaproteobacteria | Pseudomonadales |
| OTU0391 | Bacteria | Bacteroidetes | Flavobacteriia | Flavobacteriales |
| OTU0392 | Archaea | Euryarchaeota | Halobacteria | Halobacteriales |
| OTU0393 | Bacteria | Actinobacteria | Acidimicrobiia | Acidimicrobiales |
| OTU0394 | Archaea | Euryarchaeota | Halobacteria | Halobacteriales |
| OTU0395 | Bacteria | Proteobacteria | Gammaproteobacteria | Alteromonadales |
| OTU0396 | Archaea | Euryarchaeota | Halobacteria | Halobacteriales |
| OTU0397 | Bacteria | Firmicutes | Bacilli | Bacillales |
| OTU0398 | Archaea | Euryarchaeota | Halobacteria | Halobacteriales |
| OTU0399 | Bacteria | Proteobacteria | Gammaproteobacteria | Xanthomonadales |
| OTU0400 | Bacteria | Proteobacteria | Alphaproteobacteria | Rhodobacterales |
| OTU0401 | Bacteria | Firmicutes | Clostridia | Clostridiales |
| OTU0402 | Unclassified |  |  |  |
| OTU0403 | Bacteria | Actinobacteria | Actinobacteria | Corynebacteriales |
| OTU0404 | Archaea | Euryarchaeota | Halobacteria | Halobacteriales |
| OTU0405 | Bacteria | Proteobacteria | Deltaproteobacteria | Desulfarculales |
| OTU0406 | Bacteria | Firmicutes | Bacilli | Bacillales |
| OTU0407 | Bacteria | Actinobacteria | Actinobacteria | Micrococcales |
| OTU0408 | Bacteria | Proteobacteria | Betaproteobacteria | Burkholderiales |
| OTU0409 | Bacteria | Proteobacteria | Gammaproteobacteria | Alteromonadales |
| OTU0410 | Archaea | Euryarchaeota | Halobacteria | Halobacteriales |
| OTU0411 | Bacteria | Bacteroidetes | Flavobacteriia | Flavobacteriales |
| OTU0412 | Bacteria | Proteobacteria | Betaproteobacteria | Burkholderiales |
| OTU0413 | Bacteria | Proteobacteria | Gammaproteobacteria | Xanthomonadales |
| OTU0414 | Archaea | Euryarchaeota | Halobacteria | Halobacteriales |
| OTU0415 | Bacteria | Proteobacteria | Gammaproteobacteria | Oceanospirillales |
| OTU0416 | Bacteria | Bacteroidetes | Flavobacteriia | Flavobacteriales |
| OTU0417 | Archaea | Euryarchaeota | Halobacteria | Halobacteriales |
| OTU0418 | Bacteria | Proteobacteria | Gammaproteobacteria | Vibrionales |
| OTU0419 | Bacteria | Proteobacteria | Gammaproteobacteria | Enterobacteriales |
| OTU0420 | Bacteria | Firmicutes | Bacilli | Lactobacillales |
| OTU0421 | Bacteria | Actinobacteria | Actinobacteria | Corynebacteriales |
| OTU0422 | Bacteria | Firmicutes | Bacilli | Bacillales |
| OTU0423 | Archaea | Euryarchaeota | Thermoplasmata | E2 |
| OTU0424 | Archaea | Euryarchaeota | Halobacteria | Halobacteriales |
| OTU0425 | Bacteria | Proteobacteria | Gammaproteobacteria | Vibrionales |
| OTU0426 | Bacteria | Proteobacteria | Gammaproteobacteria | Enterobacteriales |
| OTU0427 | Bacteria | Proteobacteria | Gammaproteobacteria | Vibrionales |
| OTU0428 | Archaea | Euryarchaeota | Halobacteria | Halobacteriales |
| OTU0429 | Bacteria | Proteobacteria | Alphaproteobacteria | Rhodobacterales |
| OTU0430 | Bacteria | Proteobacteria | Gammaproteobacteria | Vibrionales |
| OTU0431 | Archaea | Euryarchaeota | Halobacteria | Halobacteriales |
| OTU0432 | Bacteria | Firmicutes | Bacilli | Bacillales |
| OTU0433 | Bacteria | Proteobacteria | Gammaproteobacteria | Alteromonadales |
| OTU0434 | Archaea | Euryarchaeota | Halobacteria | Halobacteriales |
| OTU0435 | Bacteria | Actinobacteria | Actinobacteria | Micrococcales |
| OTU0436 | Bacteria | Proteobacteria | Gammaproteobacteria | Enterobacteriales |
| OTU0437 | Bacteria | Proteobacteria | Betaproteobacteria | Burkholderiales |
| OTU0438 | Bacteria | Cyanobacteria | Chloroplast | Streptophyta |
| OTU0439 | Bacteria | Acidobacteria | OS-K |  |
| OTU0440 | Archaea | Euryarchaeota | Halobacteria | Halobacteriales |
| OTU0441 | Bacteria | Proteobacteria | Gammaproteobacteria | Aeromonadales |
| OTU0442 | Bacteria | Proteobacteria | Gammaproteobacteria | Xanthomonadales |
| OTU0443 | Archaea | Euryarchaeota | Halobacteria | Halobacteriales |
| OTU0444 | Archaea | Euryarchaeota | Halobacteria | Halobacteriales |
| OTU0445 | Bacteria | Proteobacteria | Gammaproteobacteria | Vibrionales |
| OTU0446 | Bacteria | Proteobacteria | Gammaproteobacteria | Oceanospirillales |
| OTU0447 | Archaea | Euryarchaeota | Halobacteria | Halobacteriales |
| OTU0448 | Bacteria | Actinobacteria | Actinobacteria | Micrococcales |
| OTU0449 | Archaea | Euryarchaeota | Halobacteria | Halobacteriales |
| OTU0450 | Bacteria | Firmicutes | Bacilli | Bacillales |
| OTU0451 | Bacteria | Actinobacteria | Actinobacteria | Micrococcales |
| OTU0452 | Bacteria | Firmicutes | Clostridia | Clostridiales |
| OTU0453 | Archaea | Euryarchaeota | Halobacteria | Halobacteriales |
| OTU0454 | Bacteria | Proteobacteria | Alphaproteobacteria | Rickettsiales |
| OTU0455 | Bacteria | Firmicutes | Bacilli | Lactobacillales |
| OTU0456 | Bacteria | Proteobacteria | Alphaproteobacteria | Rhodospirillales |
| OTU0457 | Archaea | Euryarchaeota | Halobacteria | Halobacteriales |
| OTU0458 | Bacteria | Actinobacteria | Actinobacteria | Corynebacteriales |
| OTU0459 | Bacteria | Proteobacteria | Gammaproteobacteria | Vibrionales |
| OTU0460 | Bacteria | Proteobacteria | Gammaproteobacteria | Pseudomonadales |
| OTU0461 | Archaea | Euryarchaeota | Halobacteria | Halobacteriales |
| OTU0462 | Bacteria | Firmicutes | Bacilli | Bacillales |
| OTU0463 | Bacteria | Proteobacteria | Alphaproteobacteria | Rhizobiales |
| OTU0464 | Archaea | Euryarchaeota | Halobacteria | Halobacteriales |
| OTU0465 | Bacteria | Proteobacteria | Alphaproteobacteria | Rhizobiales |
| OTU0466 | Bacteria | Firmicutes | Bacilli | Bacillales |
| OTU0467 | Bacteria | Proteobacteria | Gammaproteobacteria | Pseudomonadales |
| OTU0468 | Bacteria | Actinobacteria | Actinobacteria | Corynebacteriales |
| OTU0469 | Bacteria | Firmicutes | Bacilli |  |
| OTU0470 | Bacteria | Proteobacteria | Gammaproteobacteria | Vibrionales |
| OTU0471 | Archaea | Euryarchaeota | Halobacteria | Halobacteriales |
| OTU0472 | Bacteria | Bacteroidetes | Flavobacteriia | Flavobacteriales |
| OTU0473 | Bacteria | Proteobacteria | Gammaproteobacteria | Enterobacteriales |
| OTU0474 | Bacteria | Proteobacteria | Betaproteobacteria | Burkholderiales |
| OTU0475 | Bacteria | Firmicutes | Bacilli | Lactobacillales |
| OTU0476 | Archaea | Euryarchaeota | Halobacteria | Halobacteriales |
| OTU0477 | Bacteria | Proteobacteria | Alphaproteobacteria | Rhizobiales |
| OTU0478 | Bacteria | Firmicutes | Bacilli | Bacillales |
| OTU0479 | Bacteria | Firmicutes | Bacilli | Bacillales |
| OTU0480 | Bacteria | Proteobacteria | Alphaproteobacteria | Rhizobiales |
| OTU0481 | Unclassified |  |  |  |
| OTU0482 | Bacteria | Bacteroidetes | Flavobacteriia | Flavobacteriales |
| OTU0483 | Bacteria | Firmicutes | Bacilli | Bacillales |
| OTU0484 | Archaea | Euryarchaeota | Halobacteria | Halobacteriales |
| OTU0485 | Archaea | Euryarchaeota | Halobacteria | Halobacteriales |
| OTU0486 | Bacteria | Proteobacteria | Gammaproteobacteria | Pseudomonadales |
| OTU0487 | Archaea | Euryarchaeota | Halobacteria | Halobacteriales |
| OTU0488 | Bacteria | Proteobacteria | Gammaproteobacteria |  |
| OTU0489 | Bacteria | Proteobacteria | Gammaproteobacteria | Pseudomonadales |
| OTU0490 | Bacteria | Proteobacteria | Gammaproteobacteria | Chromatiales |
| OTU0491 | Archaea | Euryarchaeota | Halobacteria | Halobacteriales |
| OTU0492 | Bacteria | Proteobacteria | Gammaproteobacteria | Enterobacteriales |
| OTU0493 | Bacteria | Proteobacteria | Gammaproteobacteria |  |
| OTU0494 | Bacteria | Firmicutes | Bacilli | Lactobacillales |
| OTU0495 | Bacteria | Firmicutes | Erysipelotrichi | Erysipelotrichales |
| OTU0496 | Bacteria | Proteobacteria | Gammaproteobacteria |  |
| OTU0497 | Bacteria | Proteobacteria | Gammaproteobacteria | Aeromonadales |
| OTU0498 | Bacteria | Firmicutes | Bacilli | Bacillales |
| OTU0499 | Archaea | Euryarchaeota | Halobacteria | Halobacteriales |
| OTU0500 | Bacteria | Actinobacteria | Actinobacteria | Corynebacteriales |
| OTU0501 | Bacteria | Bacteroidetes | Flavobacteriia | Flavobacteriales |
| OTU0502 | Bacteria | Proteobacteria | Gammaproteobacteria | Oceanospirillales |
| OTU0503 | Bacteria | Proteobacteria | Gammaproteobacteria | Alteromonadales |
| OTU0504 | Archaea | Euryarchaeota | Halobacteria | Halobacteriales |
| OTU0505 | Bacteria | Firmicutes | Bacilli | Bacillales |
| OTU0506 | Bacteria | Actinobacteria | Actinobacteria | Micrococcales |
| OTU0507 | Bacteria | Proteobacteria | Gammaproteobacteria | Pseudomonadales |
| OTU0508 | Bacteria | Firmicutes | Bacilli | Lactobacillales |
| OTU0509 | Bacteria | Bacteroidetes | Sphingobacteriia | Sphingobacteriales |
| OTU0510 | Bacteria | Actinobacteria | Actinobacteria | Micrococcales |
| OTU0511 | Archaea | Euryarchaeota | Halobacteria | Halobacteriales |
| OTU0512 | Bacteria | Proteobacteria | Gammaproteobacteria | Pseudomonadales |
| OTU0513 | Bacteria | Proteobacteria | Gammaproteobacteria | Pseudomonadales |
| OTU0514 | Archaea | Euryarchaeota | Halobacteria | Halobacteriales |
| OTU0515 | Bacteria | Proteobacteria | Gammaproteobacteria | Oceanospirillales |
| OTU0516 | Bacteria | Proteobacteria | Gammaproteobacteria | Alteromonadales |
| OTU0517 | Bacteria | Firmicutes | Bacilli | Bacillales |
| OTU0518 | Bacteria | Proteobacteria | Gammaproteobacteria | Pseudomonadales |
| OTU0519 | Bacteria | Planctomycetes | Planctomycetia | Pirellulales |
| OTU0520 | Bacteria | Firmicutes | Bacilli | Bacillales |
| OTU0521 | Bacteria | Firmicutes | Erysipelotrichi | Erysipelotrichales |
| OTU0522 | Archaea | Euryarchaeota | Halobacteria | Halobacteriales |
| OTU0523 | Bacteria | Proteobacteria | Alphaproteobacteria | Sphingomonadales |
| OTU0524 | Archaea | Euryarchaeota | Halobacteria | Halobacteriales |
| OTU0525 | Bacteria | Proteobacteria | Gammaproteobacteria | Alteromonadales |
| OTU0526 | Archaea | Euryarchaeota | Methanomicrobia | Methanocellales |
| OTU0527 | Bacteria | Actinobacteria | Actinobacteria | Corynebacteriales |
| OTU0528 | Bacteria | Actinobacteria | Rubrobacteria | Rubrobacterales |
| OTU0529 | Bacteria | Proteobacteria | Gammaproteobacteria | Alteromonadales |
| OTU0530 | Bacteria | Proteobacteria | Gammaproteobacteria | Legionellales |
| OTU0531 | Bacteria | Proteobacteria | Gammaproteobacteria | Vibrionales |
| OTU0532 | Archaea | Euryarchaeota | Halobacteria | Halobacteriales |
| OTU0533 | Bacteria | Firmicutes | Bacilli | Bacillales |
| OTU0534 | Bacteria | Proteobacteria | Gammaproteobacteria | Enterobacteriales |
| OTU0535 | Bacteria | Proteobacteria | Betaproteobacteria | ASSO-13 |
| OTU0536 | Bacteria | Firmicutes | Clostridia | Clostridiales |
| OTU0537 | Bacteria | Proteobacteria | Gammaproteobacteria |  |
| OTU0538 | Bacteria | Bacteroidetes | Bacteroidia | Bacteroidales |
| OTU0539 | Bacteria | Proteobacteria | Gammaproteobacteria | Pseudomonadales |
| OTU0540 | Archaea | Euryarchaeota | Halobacteria | Halobacteriales |
| OTU0541 | Archaea | Euryarchaeota | Halobacteria | Halobacteriales |
| OTU0542 | Bacteria | Firmicutes | Bacilli | Bacillales |
| OTU0543 | Archaea | Euryarchaeota | Halobacteria | Halobacteriales |
| OTU0544 | Bacteria | Proteobacteria | Alphaproteobacteria | Rhizobiales |
| OTU0545 | Bacteria | Proteobacteria | Gammaproteobacteria | Chromatiales |
| OTU0546 | Bacteria | Firmicutes | Bacilli | Bacillales |
| OTU0547 | Archaea | Euryarchaeota | Halobacteria | Halobacteriales |
| OTU0548 | Bacteria | Firmicutes | Clostridia | Clostridiales |
| OTU0549 | Bacteria | Firmicutes | Bacilli | Bacillales |
| OTU0550 | Archaea | Euryarchaeota | Halobacteria | Halobacteriales |
| OTU0551 | Bacteria | Firmicutes | Bacilli | Bacillales |
| OTU0552 | Archaea | Euryarchaeota | Halobacteria | Halobacteriales |
| OTU0553 | Bacteria | Actinobacteria | Actinobacteria | Micrococcales |
| OTU0554 | Archaea | Euryarchaeota | Halobacteria | Halobacteriales |
| OTU0555 | Archaea | Euryarchaeota | Halobacteria | Halobacteriales |
| OTU0556 | Archaea | Euryarchaeota | Halobacteria | Halobacteriales |
| OTU0557 | Archaea | Euryarchaeota | Halobacteria | Halobacteriales |
| OTU0558 | Bacteria | Proteobacteria | Gammaproteobacteria | Enterobacteriales |
| OTU0559 | Bacteria | Bacteroidetes | Flavobacteriia | Flavobacteriales |
| OTU0560 | Bacteria | Proteobacteria | Gammaproteobacteria |  |
| OTU0561 | Archaea | Euryarchaeota | Halobacteria | Halobacteriales |
| OTU0562 | Bacteria | Firmicutes | Bacilli | Bacillales |
| OTU0563 | Bacteria | Firmicutes | Bacilli | Bacillales |
| OTU0564 | Bacteria | Actinobacteria | Actinobacteria | Micrococcales |
| OTU0565 | Bacteria | Proteobacteria | Alphaproteobacteria | Sphingomonadales |
| OTU0566 | Bacteria | Actinobacteria | Actinobacteria | Corynebacteriales |
| OTU0567 | Bacteria | Proteobacteria | Gammaproteobacteria | Pseudomonadales |
| OTU0568 | Bacteria | Bacteroidetes | Flavobacteriia | Flavobacteriales |
| OTU0569 | Archaea | Euryarchaeota | Halobacteria | Halobacteriales |
| OTU0570 | Bacteria | Proteobacteria | Alphaproteobacteria | Rhodobacterales |
| OTU0571 | Bacteria | Proteobacteria | Gammaproteobacteria | Legionellales |
| OTU0572 | Bacteria | Proteobacteria | Gammaproteobacteria | Alteromonadales |
| OTU0573 | Bacteria | Proteobacteria | Gammaproteobacteria |  |
| OTU0574 | Bacteria | Proteobacteria | Betaproteobacteria | Burkholderiales |
| OTU0575 | Archaea | Euryarchaeota | Halobacteria | Halobacteriales |
| OTU0576 | Bacteria | Firmicutes | Bacilli | Bacillales |
| OTU0577 | Bacteria | Proteobacteria | Gammaproteobacteria | Enterobacteriales |
| OTU0578 | Archaea | Euryarchaeota | Halobacteria | Halobacteriales |
| OTU0579 | Bacteria | Firmicutes | Bacilli | Lactobacillales |
| OTU0580 | Bacteria | Proteobacteria | Gammaproteobacteria | Aeromonadales |
| OTU0581 | Bacteria | Bacteroidetes | [Saprospirae] | [Saprospirales] |
| OTU0582 | Archaea | Euryarchaeota | Halobacteria | Halobacteriales |
| OTU0583 | Bacteria | Proteobacteria | Alphaproteobacteria | Rhodobacterales |
| OTU0584 | Archaea | Euryarchaeota | Halobacteria | Halobacteriales |
| OTU0585 | Bacteria | Firmicutes | Bacilli | Bacillales |
| OTU0586 | Bacteria | Firmicutes | Bacilli | Bacillales |
| OTU0587 | Bacteria | Firmicutes | Bacilli | Bacillales |
| OTU0588 | Archaea | Euryarchaeota | Halobacteria | Halobacteriales |
| OTU0589 | Archaea | Euryarchaeota | Halobacteria | Halobacteriales |
| OTU0590 | Bacteria | Proteobacteria | Gammaproteobacteria | Alteromonadales |
| OTU0591 | Bacteria | Proteobacteria | Gammaproteobacteria | Chromatiales |
| OTU0592 | Bacteria | Firmicutes | Bacilli | Turicibacterales |
| OTU0593 | Bacteria | Proteobacteria | Gammaproteobacteria | Vibrionales |
| OTU0594 | Bacteria | Proteobacteria | Gammaproteobacteria | Salinisphaerales |
| OTU0595 | Bacteria | Bacteroidetes | Bacteroidia | Bacteroidales |
| OTU0596 | Bacteria | Firmicutes | Bacilli | Bacillales |
| OTU0597 | Bacteria | Firmicutes | Bacilli | Bacillales |
| OTU0598 | Archaea | Euryarchaeota | Halobacteria | Halobacteriales |
| OTU0599 | Bacteria | Proteobacteria | Gammaproteobacteria | Alteromonadales |
| OTU0600 | Bacteria | Proteobacteria | Gammaproteobacteria |  |
| OTU0601 | Bacteria | Firmicutes | Erysipelotrichi | Erysipelotrichales |
| OTU0602 | Bacteria | Firmicutes | Bacilli | Bacillales |
| OTU0603 | Bacteria | Firmicutes | Bacilli | Bacillales |
| OTU0604 | Archaea | Euryarchaeota | Halobacteria | Halobacteriales |
| OTU0605 | Bacteria | Actinobacteria | Actinobacteria | Corynebacteriales |
| OTU0606 | Bacteria | Firmicutes | Bacilli | Lactobacillales |
| OTU0607 | Bacteria | Proteobacteria | Gammaproteobacteria | Pseudomonadales |
| OTU0608 | Bacteria | Actinobacteria | Actinobacteria | Nocardiales |
| OTU0609 | Bacteria | Firmicutes | Bacilli | Lactobacillales |
| OTU0610 | Archaea | Euryarchaeota | Halobacteria | Halobacteriales |
| OTU0611 | Bacteria | Proteobacteria | Gammaproteobacteria | Alteromonadales |
| OTU0612 | Bacteria | Firmicutes | Bacilli |  |
| OTU0613 | Archaea | Euryarchaeota | Halobacteria | Halobacteriales |
| OTU0614 | Bacteria | Proteobacteria | Gammaproteobacteria | Pseudomonadales |
| OTU0615 | Bacteria | Bacteroidetes | [Rhodothermi] | [Rhodothermales] |
| OTU0616 | Bacteria | Proteobacteria | Gammaproteobacteria | Pseudomonadales |
| OTU0617 | Bacteria | Proteobacteria | Gammaproteobacteria |  |
| OTU0618 | Bacteria | Firmicutes | Bacilli | Bacillales |
| OTU0619 | Bacteria | Proteobacteria | Gammaproteobacteria | Pseudomonadales |
| OTU0620 | Archaea | Euryarchaeota | Halobacteria | Halobacteriales |
| OTU0621 | Bacteria | Proteobacteria | Alphaproteobacteria | Sphingomonadales |
| OTU0622 | Bacteria | Firmicutes | Bacilli | Lactobacillales |
| OTU0623 | Bacteria | Proteobacteria | Alphaproteobacteria | Rhodobacterales |
| OTU0624 | Archaea | Euryarchaeota | Halobacteria | Halobacteriales |
| OTU0625 | Bacteria | Proteobacteria | Gammaproteobacteria | Oceanospirillales |
| OTU0626 | Bacteria | Firmicutes | Bacilli | Bacillales |
| OTU0627 | Bacteria | Proteobacteria | Gammaproteobacteria | Alteromonadales |
| OTU0628 | Bacteria | Firmicutes | Bacilli | Lactobacillales |
| OTU0629 | Bacteria | Proteobacteria | Gammaproteobacteria | Alteromonadales |
| OTU0630 | Bacteria | Actinobacteria | Actinobacteria | Micrococcales |
| OTU0631 | Bacteria | Proteobacteria | Gammaproteobacteria | Pseudomonadales |
| OTU0632 | Bacteria | Proteobacteria | Betaproteobacteria | Burkholderiales |
| OTU0633 | Bacteria | Actinobacteria | Actinobacteria | Corynebacteriales |
| OTU0634 | Archaea | Euryarchaeota | Halobacteria | Halobacteriales |
| OTU0635 | Bacteria | Proteobacteria | Gammaproteobacteria | Pseudomonadales |
| OTU0636 | Bacteria | Actinobacteria | Actinobacteria | Actinomycetales |
| OTU0637 | Archaea | Euryarchaeota | Halobacteria | Halobacteriales |
| OTU0638 | Bacteria | Firmicutes | Bacilli |  |
| OTU0639 | Bacteria | Firmicutes | Bacilli | Bacillales |
| OTU0640 | Bacteria | Proteobacteria | Gammaproteobacteria | Aeromonadales |
| OTU0641 | Bacteria | Proteobacteria | Alphaproteobacteria | Caulobacterales |
| OTU0642 | Archaea | Euryarchaeota | Halobacteria | Halobacteriales |
| OTU0643 | Archaea | Euryarchaeota | Halobacteria | Halobacteriales |
| OTU0644 | Bacteria | Firmicutes | Clostridia | Clostridiales |
| OTU0645 | Archaea | Euryarchaeota | Halobacteria | Halobacteriales |
| OTU0646 | Bacteria | Firmicutes | Bacilli | Bacillales |
| OTU0647 | Archaea | Euryarchaeota | Halobacteria | Halobacteriales |
| OTU0648 | Bacteria | Actinobacteria | Actinobacteria | Micrococcales |
| OTU0649 | Bacteria | Proteobacteria | Deltaproteobacteria | Desulfovibrionales |
| OTU0650 | Bacteria | Proteobacteria | Gammaproteobacteria | Xanthomonadales |
| OTU0651 | Bacteria | Firmicutes | Bacilli | Bacillales |
| OTU0652 | Bacteria | Firmicutes | Bacilli | Bacillales |
| OTU0653 | Bacteria | Firmicutes | Bacilli | Bacillales |
| OTU0654 | Bacteria | Proteobacteria | Alphaproteobacteria | Rhizobiales |
| OTU0655 | Bacteria | Actinobacteria | Actinobacteria | Micrococcales |
| OTU0656 | Bacteria | Proteobacteria | Gammaproteobacteria | Salinisphaerales |
| OTU0657 | Archaea | Euryarchaeota | Halobacteria | Halobacteriales |
| OTU0658 | Bacteria | Firmicutes | Bacilli | Lactobacillales |
| OTU0659 | Archaea | Euryarchaeota | Halobacteria | Halobacteriales |
| OTU0660 | Bacteria | Proteobacteria | Alphaproteobacteria | Rhizobiales |
| OTU0661 | Bacteria | Proteobacteria | Alphaproteobacteria | Rhodospirillales |
| OTU0662 | Bacteria | Proteobacteria | Betaproteobacteria | Burkholderiales |
| OTU0663 | Archaea | Euryarchaeota | Halobacteria | Halobacteriales |
| OTU0664 | Bacteria | Proteobacteria | Gammaproteobacteria | Oceanospirillales |
| OTU0665 | Bacteria | Proteobacteria | Gammaproteobacteria | Oceanospirillales |
| OTU0666 | Bacteria | Firmicutes | Bacilli | Lactobacillales |
| OTU0667 | Bacteria | Bacteroidetes | Sphingobacteriia | Sphingobacteriales |
| OTU0668 | Bacteria | Firmicutes | Bacilli | Bacillales |
| OTU0669 | Bacteria | Firmicutes | Bacilli | Bacillales |
| OTU0670 | Bacteria | Proteobacteria | Gammaproteobacteria | Vibrionales |
| OTU0671 | Bacteria | Proteobacteria | Gammaproteobacteria | Enterobacteriales |
| OTU0672 | Bacteria | Firmicutes | Bacilli | Bacillales |
| OTU0673 | Bacteria | Proteobacteria | Gammaproteobacteria | Oceanospirillales |
| OTU0674 | Bacteria | Firmicutes | Bacilli | Bacillales |
| OTU0675 | Bacteria | Proteobacteria | Gammaproteobacteria | Aeromonadales |
| OTU0676 | Bacteria | Firmicutes | Bacilli | Bacillales |
| OTU0677 | Archaea | Euryarchaeota | Halobacteria | Halobacteriales |
| OTU0678 | Bacteria | Firmicutes | Bacilli | Bacillales |
| OTU0679 | Bacteria | Proteobacteria | Betaproteobacteria | Burkholderiales |
| OTU0680 | Archaea | Euryarchaeota | Halobacteria | Halobacteriales |
| OTU0681 | Archaea | Euryarchaeota | Halobacteria | Halobacteriales |
| OTU0682 | Bacteria | Proteobacteria | Alphaproteobacteria | Rhodobacterales |
| OTU0683 | Bacteria | Firmicutes | Bacilli | Lactobacillales |
| OTU0684 | Archaea | Euryarchaeota | Halobacteria | Halobacteriales |
| OTU0685 | Bacteria | Actinobacteria | Actinobacteria | Corynebacteriales |
| OTU0686 | Bacteria | Proteobacteria | Alphaproteobacteria | Rhodobacterales |
| OTU0687 | Bacteria | Proteobacteria | Gammaproteobacteria | Pseudomonadales |
| OTU0688 | Bacteria | Proteobacteria | Gammaproteobacteria | Vibrionales |
| OTU0689 | Bacteria | Proteobacteria | Gammaproteobacteria | Pseudomonadales |
| OTU0690 | Bacteria | [Thermi] | Deinococci | Deinococcales |
| OTU0691 | Bacteria | Firmicutes | Clostridia | Clostridiales |
| OTU0692 | Bacteria | Proteobacteria | Gammaproteobacteria | Pseudomonadales |
| OTU0693 | Bacteria | Proteobacteria | Gammaproteobacteria |  |
| OTU0694 | Bacteria | Proteobacteria | Gammaproteobacteria | Oceanospirillales |
| OTU0695 | Bacteria | Firmicutes | Bacilli | Lactobacillales |
| OTU0696 | Bacteria | Actinobacteria | Acidimicrobiia | Acidimicrobiales |
| OTU0697 | Archaea | Euryarchaeota | Halobacteria | Halobacteriales |
| OTU0698 | Bacteria | Actinobacteria | Actinobacteria | Micrococcales |
| OTU0699 | Bacteria | Proteobacteria | Gammaproteobacteria | Pseudomonadales |
| OTU0700 | Archaea | Euryarchaeota | Halobacteria | Halobacteriales |
| OTU0701 | Archaea | Euryarchaeota | Thermoplasmata | E2 |
| OTU0702 | Bacteria | Proteobacteria | Gammaproteobacteria |  |
| OTU0703 | Archaea | Euryarchaeota | Thermoplasmata | E2 |
| OTU0704 | Bacteria |  |  |  |
| OTU0705 | Bacteria | Proteobacteria | Deltaproteobacteria | Desulfarculales |
| OTU0706 | Bacteria | Proteobacteria | Gammaproteobacteria | Vibrionales |
| OTU0707 | Bacteria | Proteobacteria | Alphaproteobacteria | Rhizobiales |
| OTU0708 | Bacteria | Proteobacteria | Gammaproteobacteria | Pseudomonadales |
| OTU0709 | Bacteria | Firmicutes | Clostridia | Clostridiales |
| OTU0710 | Bacteria | Firmicutes | Bacilli | Bacillales |
| OTU0711 | Archaea | Euryarchaeota | Halobacteria | Halobacteriales |
| OTU0712 | Bacteria | Firmicutes | Bacilli | Lactobacillales |
| OTU0713 | Archaea | Euryarchaeota | Thermoplasmata | E2 |
| OTU0714 | Bacteria | Firmicutes | Bacilli | Lactobacillales |
| OTU0715 | Bacteria | Firmicutes | Bacilli | Bacillales |
| OTU0716 | Archaea | Euryarchaeota | Halobacteria | Halobacteriales |
| OTU0717 | Bacteria | Proteobacteria | Betaproteobacteria | Burkholderiales |
| OTU0718 | Bacteria | Proteobacteria | Betaproteobacteria | Burkholderiales |
| OTU0719 | Bacteria | Acidobacteria | Sva0725 | Sva0725 |
| OTU0720 | Archaea | Euryarchaeota | Halobacteria | Halobacteriales |
| OTU0721 | Bacteria | Proteobacteria | Gammaproteobacteria | Alteromonadales |
| OTU0722 | Bacteria | Proteobacteria | Gammaproteobacteria | Pseudomonadales |
| OTU0723 | Bacteria | Bacteroidetes | Sphingobacteriia | Sphingobacteriales |
| OTU0724 | Bacteria | Firmicutes | Bacilli | Gemellales |
| OTU0725 | Bacteria | Firmicutes | Bacilli | Bacillales |
| OTU0726 | Archaea | Euryarchaeota | Halobacteria | Halobacteriales |
| OTU0727 | Bacteria | Proteobacteria | Gammaproteobacteria | Alteromonadales |
| OTU0728 | Bacteria | Actinobacteria | Actinobacteria | Actinomycetales |
| OTU0729 | Bacteria | Proteobacteria | Gammaproteobacteria | Pseudomonadales |
| OTU0730 | Bacteria | Proteobacteria | Gammaproteobacteria | Pseudomonadales |
| OTU0731 | Bacteria | Proteobacteria | Deltaproteobacteria | Bdellovibrionales |
| OTU0732 | Bacteria | Proteobacteria | Gammaproteobacteria | Oceanospirillales |
| OTU0733 | Bacteria | Firmicutes | Bacilli | Bacillales |
| OTU0734 | Archaea | Euryarchaeota | DSEG | DHVE3 |
| OTU0735 | Archaea | Euryarchaeota | ANME-1 |  |
| OTU0736 | Bacteria | Proteobacteria | Betaproteobacteria | Neisseriales |
| OTU0737 | Bacteria | Proteobacteria | Alphaproteobacteria | Rhizobiales |
| OTU0738 | Bacteria | Firmicutes | Bacilli | Bacillales |
| OTU0739 | Archaea | Euryarchaeota | Halobacteria | Halobacteriales |
| OTU0740 | Archaea | Euryarchaeota | Archaeoglobi | Archaeoglobales |
| OTU0741 | Bacteria | Bacteroidetes | Flavobacteriia | Flavobacteriales |
| OTU0742 | Archaea | Euryarchaeota | Archaeoglobi | Archaeoglobales |
| OTU0743 | Bacteria | Proteobacteria | Gammaproteobacteria | Pseudomonadales |
| OTU0744 | Bacteria | Proteobacteria | Gammaproteobacteria | Vibrionales |
| OTU0745 | Archaea | Euryarchaeota | Halobacteria | Halobacteriales |
| OTU0746 | Bacteria | Proteobacteria | Gammaproteobacteria | Vibrionales |
| OTU0747 | Bacteria | Proteobacteria | Gammaproteobacteria | Enterobacteriales |
| OTU0748 | Bacteria | Proteobacteria | Gammaproteobacteria | Oceanospirillales |
| OTU0749 | Bacteria | Actinobacteria | Actinobacteria | Corynebacteriales |
| OTU0750 | Bacteria | Proteobacteria | Gammaproteobacteria | Chromatiales |
| OTU0751 | Bacteria | Proteobacteria | Gammaproteobacteria | Alteromonadales |
| OTU0752 | Bacteria | Proteobacteria | Gammaproteobacteria | Alteromonadales |
| OTU0753 | Bacteria | Proteobacteria | Gammaproteobacteria | Aeromonadales |
| OTU0754 | Archaea | Euryarchaeota | Thermococci | Thermococcales |
| OTU0755 | Bacteria | Firmicutes | Bacilli | Bacillales |
| OTU0756 | Bacteria | Proteobacteria | Gammaproteobacteria | Enterobacteriales |
| OTU0757 | Bacteria | Proteobacteria | Alphaproteobacteria | Rhodobacterales |
| OTU0758 | Bacteria | Proteobacteria | Alphaproteobacteria |  |
| OTU0759 | Archaea | Crenarchaeota |  |  |
| OTU0760 | Archaea | Crenarchaeota |  |  |
| OTU0761 | Archaea | Euryarchaeota | DSEG | DHVE3 |
| OTU0762 | Archaea | Euryarchaeota | Halobacteria | Halobacteriales |
| OTU0763 | Archaea | [Parvarchaeota] | [Parvarchaea] | YLA114 |
| OTU0764 | Bacteria | Proteobacteria | Gammaproteobacteria | Salinisphaerales |
| OTU0765 | Bacteria | Actinobacteria | Actinobacteria | Micrococcales |
| OTU0766 | Bacteria | Chloroflexi | Anaerolineae | SBR1031 |
| OTU0767 | Bacteria | Fusobacteria | Fusobacteriia | Fusobacteriales |
| OTU0768 | Bacteria | Firmicutes | Bacilli | Bacillales |
| OTU0769 | Bacteria | Proteobacteria | Alphaproteobacteria | Rhodobacterales |
| OTU0770 | Archaea | Euryarchaeota | Halobacteria | Halobacteriales |
| OTU0771 | Bacteria |  |  |  |
| OTU0772 | Archaea | Euryarchaeota | DSEG | DHVE3 |
| OTU0773 | Bacteria | Actinobacteria | Actinobacteria | Micrococcales |
| OTU0774 | Archaea | Euryarchaeota | Halobacteria | Halobacteriales |
| OTU0775 | Bacteria | Proteobacteria | Gammaproteobacteria | Pseudomonadales |
| OTU0776 | Archaea | Euryarchaeota | Halobacteria | Halobacteriales |
| OTU0777 | Archaea | Euryarchaeota | Thermococci | Thermococcales |
| OTU0778 | Bacteria | Firmicutes | Clostridia | Clostridiales |
| OTU0779 | Bacteria | Proteobacteria | Alphaproteobacteria | Rhodobacterales |
| OTU0780 | Bacteria | Firmicutes | Bacilli | Bacillales |
| OTU0781 | Archaea | Euryarchaeota | Halobacteria | Halobacteriales |
| OTU0782 | Bacteria | Proteobacteria | Gammaproteobacteria | Vibrionales |
| OTU0783 | Bacteria | Firmicutes | Bacilli | Bacillales |
| OTU0784 | Bacteria | Firmicutes | Bacilli | Bacillales |
| OTU0785 | Bacteria | Proteobacteria | Gammaproteobacteria | Vibrionales |
| OTU0786 | Bacteria | Firmicutes | Bacilli | Bacillales |
| OTU0787 | Bacteria | Firmicutes | Bacilli | Bacillales |
| OTU0788 | Bacteria | Proteobacteria | Gammaproteobacteria | Pseudomonadales |
| OTU0789 | Bacteria | OP8 | OP8_2 |  |
| OTU0790 | Bacteria | Proteobacteria | Gammaproteobacteria | Vibrionales |
| OTU0791 | Archaea | Euryarchaeota | Thermococci | Thermococcales |
| OTU0792 | Bacteria | Firmicutes | Bacilli | Lactobacillales |
| OTU0793 | Bacteria | Firmicutes | Bacilli | Lactobacillales |
| OTU0794 | Archaea | Euryarchaeota | Thermococci | Thermococcales |
| OTU0795 | Archaea | Euryarchaeota | Halobacteria | Halobacteriales |
| OTU0796 | Archaea | Euryarchaeota | Halobacteria | Halobacteriales |
| OTU0797 | Archaea | Euryarchaeota | Halobacteria | Halobacteriales |
| OTU0798 | Bacteria | Proteobacteria | Gammaproteobacteria | Pseudomonadales |
| OTU0799 | Bacteria | Firmicutes | Bacilli | Lactobacillales |
| OTU0800 | Archaea | Euryarchaeota | Halobacteria | Halobacteriales |
| OTU0801 | Archaea | Euryarchaeota | Halobacteria | Halobacteriales |
| OTU0802 | Archaea | Euryarchaeota | Halobacteria | Halobacteriales |
| OTU0803 | Bacteria | Proteobacteria | Gammaproteobacteria | Vibrionales |
| OTU0804 | Bacteria | Proteobacteria | Gammaproteobacteria | Pseudomonadales |
| OTU0805 | Bacteria | Firmicutes | Bacilli | Lactobacillales |
| OTU0806 | Bacteria | Proteobacteria | Gammaproteobacteria | Alteromonadales |
| OTU0807 | Archaea | Euryarchaeota | Halobacteria | Halobacteriales |
| OTU0808 | Bacteria | Proteobacteria | Alphaproteobacteria | Rhizobiales |
| OTU0809 | Bacteria | Proteobacteria | Gammaproteobacteria | Enterobacteriales |
| OTU0810 | Archaea | Crenarchaeota | Thermoprotei |  |
| OTU0811 | Bacteria | Firmicutes | Bacilli | Bacillales |
| OTU0812 | Archaea | Crenarchaeota | Thermoprotei | Thermoproteales |
| OTU0813 | Bacteria | Proteobacteria | Gammaproteobacteria | Chromatiales |
| OTU0814 | Bacteria | Proteobacteria | Gammaproteobacteria | Pseudomonadales |
| OTU0815 | Archaea | Crenarchaeota | MCG | pGrfC26 |
| OTU0816 | Archaea | Crenarchaeota | MBGB |  |
| OTU0817 | Bacteria | Proteobacteria | Gammaproteobacteria | Vibrionales |
| OTU0818 | Archaea | Euryarchaeota | Halobacteria | Halobacteriales |
| OTU0819 | Bacteria | Firmicutes | Bacilli | Bacillales |
| OTU0820 | Bacteria | Proteobacteria | Gammaproteobacteria | Aeromonadales |
| OTU0821 | Bacteria | Firmicutes | Bacilli | Lactobacillales |
| OTU0822 | Bacteria | Proteobacteria | Gammaproteobacteria | Pseudomonadales |
| OTU0823 | Archaea | Crenarchaeota | MCG |  |
| OTU0824 | Archaea | Crenarchaeota | Aigarchaeota | [Caldiarchales] |
| OTU0825 | Bacteria | Bacteroidetes | Bacteroidia | Bacteroidales |
| OTU0826 | Bacteria | Bacteroidetes | Bacteroidia | Bacteroidales |
| OTU0827 | Bacteria | Proteobacteria | Gammaproteobacteria | Enterobacteriales |
| OTU0828 | Bacteria | Actinobacteria | Actinobacteria | Micrococcales |
| OTU0829 | Bacteria | Firmicutes | Clostridia | Clostridiales |
| OTU0830 | Archaea | Euryarchaeota | Halobacteria | Halobacteriales |
| OTU0831 | Archaea | Euryarchaeota | Halobacteria | Halobacteriales |
| OTU0832 | Bacteria | Proteobacteria | Gammaproteobacteria | Chromatiales |
| OTU0833 | Bacteria | Proteobacteria | Deltaproteobacteria | Myxococcales |
| OTU0834 | Bacteria | Firmicutes | Bacilli | Bacillales |
| OTU0835 | Bacteria | Proteobacteria | Gammaproteobacteria | Pseudomonadales |
| OTU0836 | Bacteria | Proteobacteria | Gammaproteobacteria | Alteromonadales |
| OTU0837 | Bacteria | Proteobacteria | Gammaproteobacteria | Vibrionales |
| OTU0838 | Archaea | Euryarchaeota | Halobacteria | Halobacteriales |
| OTU0839 | Archaea | Euryarchaeota | Halobacteria | Halobacteriales |
| OTU0840 | Bacteria | Actinobacteria | Acidimicrobiia | Acidimicrobiales |
| OTU0841 | Archaea | Euryarchaeota | Halobacteria | Halobacteriales |
| OTU0842 | Bacteria | Cyanobacteria | Chloroplast |  |
| OTU0843 | Bacteria | Firmicutes | Clostridia | Clostridiales |
| OTU0844 | Bacteria | Bacteroidetes | Sphingobacteriia | Sphingobacteriales |
| OTU0845 | Bacteria | Proteobacteria | Gammaproteobacteria | Salinisphaerales |
| OTU0846 | Bacteria | Firmicutes | Bacilli | Bacillales |
| OTU0847 | Bacteria | Proteobacteria | Gammaproteobacteria | Aeromonadales |
| OTU0848 | Bacteria | Proteobacteria | Gammaproteobacteria | Alteromonadales |
| OTU0849 | Archaea | Euryarchaeota | Halobacteria | Halobacteriales |
| OTU0850 | Bacteria | Proteobacteria | Gammaproteobacteria | Pseudomonadales |
| OTU0851 | Archaea | Euryarchaeota | Halobacteria | Halobacteriales |
| OTU0852 | Bacteria | Proteobacteria | Gammaproteobacteria | Alteromonadales |
| OTU0853 | Bacteria | Actinobacteria | Actinobacteria | Corynebacteriales |
| OTU0854 | Bacteria | Proteobacteria | Gammaproteobacteria | Pseudomonadales |
| OTU0855 | Bacteria | Firmicutes | Bacilli | Bacillales |
| OTU0856 | Archaea | Euryarchaeota | Halobacteria | Halobacteriales |
| OTU0857 | Bacteria | Proteobacteria | Gammaproteobacteria | Vibrionales |
| OTU0858 | Bacteria | Firmicutes | Bacilli | Bacillales |
| OTU0859 | Bacteria | Proteobacteria | Gammaproteobacteria | Oceanospirillales |
| OTU0860 | Bacteria | Bacteroidetes | Sphingobacteriia | Sphingobacteriales |
| OTU0861 | Bacteria | Firmicutes | Bacilli | Bacillales |
| OTU0862 | Archaea | Euryarchaeota | Halobacteria | Halobacteriales |
| OTU0863 | Bacteria | Firmicutes | Bacilli | Lactobacillales |
| OTU0864 | Bacteria | Proteobacteria | Gammaproteobacteria | Chromatiales |
| OTU0865 | Bacteria | Proteobacteria | Betaproteobacteria | Burkholderiales |
| OTU0866 | Archaea | Euryarchaeota | Halobacteria | Halobacteriales |
| OTU0867 | Archaea | Euryarchaeota | Halobacteria | Halobacteriales |
| OTU0868 | Bacteria | Firmicutes | Bacilli | Bacillales |
| OTU0869 | Archaea | Crenarchaeota | pOWA133 |  |
| OTU0870 | Bacteria |  |  |  |
| OTU0871 | Bacteria | Proteobacteria | Gammaproteobacteria | Enterobacteriales |
| OTU0872 | Archaea | Crenarchaeota | Korarchaeota | Korarchaeotales |
| OTU0873 | Bacteria | Proteobacteria | Gammaproteobacteria | Pseudomonadales |
| OTU0874 | Archaea | Crenarchaeota |  |  |
| OTU0875 | Bacteria | Proteobacteria | Gammaproteobacteria | Thiotrichales |
| OTU0876 | Bacteria | Proteobacteria | Gammaproteobacteria | Pseudomonadales |
| OTU0877 | Bacteria | Actinobacteria | Actinobacteria | Actinomycetales |
| OTU0878 | Bacteria | Proteobacteria | Betaproteobacteria | Burkholderiales |
| OTU0879 | Bacteria | Firmicutes | Bacilli | Bacillales |
| OTU0880 | Bacteria | Firmicutes | Bacilli | Lactobacillales |
| OTU0881 | Bacteria | Actinobacteria | Actinobacteria | Corynebacteriales |
| OTU0882 | Bacteria | Fusobacteria | Fusobacteriia | Fusobacteriales |
| OTU0883 | Bacteria | Proteobacteria | Alphaproteobacteria | Rhodospirillales |
| OTU0884 | Archaea | Euryarchaeota | Halobacteria | Halobacteriales |
| OTU0885 | Bacteria | Actinobacteria | Actinobacteria | Actinomycetales |
| OTU0886 | Archaea | Euryarchaeota | Halobacteria | Halobacteriales |
| OTU0887 | Archaea | Euryarchaeota | Halobacteria | Halobacteriales |
| OTU0888 | Bacteria | Actinobacteria | Actinobacteria | Actinomycetales |
| OTU0889 | Bacteria | Proteobacteria | Alphaproteobacteria | Rhizobiales |
| OTU0890 | Bacteria | Firmicutes | Bacilli | Lactobacillales |
| OTU0891 | Bacteria | Proteobacteria | Gammaproteobacteria |  |
| OTU0892 | Bacteria | Verrucomicrobia | [Spartobacteria] | [Chthoniobacterales] |
| OTU0893 | Archaea | Euryarchaeota | Halobacteria | Halobacteriales |
| OTU0894 | Bacteria | Actinobacteria | Coriobacteriia | Coriobacteriales |
| OTU0895 | Archaea | Crenarchaeota | Thermoprotei | Desulfurococcales |
| OTU0896 | Archaea | Crenarchaeota | Thermoprotei | Desulfurococcales |
| OTU0897 | Archaea | Euryarchaeota | Halobacteria | Halobacteriales |
| OTU0898 | Archaea | Crenarchaeota | Thermoprotei | Desulfurococcales |
| OTU0899 | Archaea | Euryarchaeota | Halobacteria | Halobacteriales |
| OTU0900 | Archaea | Euryarchaeota | Halobacteria | Halobacteriales |
| OTU0901 | Bacteria | Firmicutes | Bacilli | Lactobacillales |
| OTU0902 | Bacteria | Proteobacteria | Gammaproteobacteria | Pseudomonadales |
| OTU0903 | Archaea | Crenarchaeota | Aigarchaeota |  |
| OTU0904 | Archaea | Euryarchaeota | Halobacteria | Halobacteriales |
| OTU0905 | Archaea | Crenarchaeota | Thermoprotei | Desulfurococcales |
| OTU0906 | Bacteria | Actinobacteria | Actinobacteria | Bifidobacteriales |
| OTU0907 | Bacteria | Firmicutes | Bacilli | Bacillales |
| OTU0908 | Bacteria | Proteobacteria | Gammaproteobacteria | Pseudomonadales |
| OTU0909 | Bacteria | Proteobacteria | Gammaproteobacteria | Vibrionales |
| OTU0910 | Bacteria | Firmicutes | Bacilli | Bacillales |
| OTU0911 | Bacteria | Firmicutes | Clostridia | Clostridiales |
| OTU0912 | Bacteria | Firmicutes | Bacilli | Bacillales |
| OTU0913 | Bacteria | Proteobacteria | Gammaproteobacteria | Pseudomonadales |
| OTU0914 | Archaea | Euryarchaeota | Halobacteria | Halobacteriales |
| OTU0915 | Bacteria | Proteobacteria | Gammaproteobacteria | Chromatiales |
| OTU0916 | Archaea | Euryarchaeota | Halobacteria | Halobacteriales |
| OTU0917 | Bacteria | Bacteroidetes | Bacteroidia | Bacteroidales |
| OTU0918 | Bacteria | Proteobacteria | Gammaproteobacteria | Chromatiales |
| OTU0919 | Bacteria | Proteobacteria | Gammaproteobacteria | Xanthomonadales |
| OTU0920 | Archaea | Crenarchaeota | Thermoprotei | Desulfurococcales |
| OTU0921 | Archaea | Crenarchaeota | pUWA2 |  |
| OTU0922 | Bacteria | Firmicutes | Clostridia | Clostridiales |
| OTU0923 | Archaea | Euryarchaeota | Halobacteria | Halobacteriales |
| OTU0924 | Bacteria | Proteobacteria | Alphaproteobacteria | Rhodobacterales |
| OTU0925 | Bacteria | Firmicutes | Bacilli |  |
| OTU0926 | Bacteria | Proteobacteria | Gammaproteobacteria | Alteromonadales |
| OTU0927 | Bacteria | Proteobacteria | Alphaproteobacteria | Rhizobiales |
| OTU0928 | Bacteria | Actinobacteria | Actinobacteria | Actinomycetales |
| OTU0929 | Archaea | Crenarchaeota | Thermoprotei | Sulfolobales |
| OTU0930 | Archaea | Euryarchaeota | Halobacteria | Halobacteriales |
| OTU0931 | Bacteria | Bacteroidetes | Flavobacteriia | Flavobacteriales |
| OTU0932 | Bacteria | Proteobacteria | Alphaproteobacteria | Sphingomonadales |
| OTU0933 | Bacteria | Firmicutes | Bacilli | Bacillales |
| OTU0934 | Bacteria | Proteobacteria | Gammaproteobacteria | Pseudomonadales |
| OTU0935 | Bacteria | Proteobacteria | Alphaproteobacteria | Rhizobiales |
| OTU0936 | Bacteria | Acidobacteria | Sva0725 | Sva0725 |
| OTU0937 | Archaea | Crenarchaeota | Thermoprotei | Sulfolobales |
| OTU0938 | Bacteria | Planctomycetes | Planctomycetia | Pirellulales |
| OTU0939 | Archaea | Euryarchaeota | Halobacteria | Halobacteriales |
| OTU0940 | Bacteria | Proteobacteria | Gammaproteobacteria | Pseudomonadales |
| OTU0941 | Bacteria | Proteobacteria | Gammaproteobacteria | Pseudomonadales |
| OTU0942 | Bacteria | Proteobacteria | Alphaproteobacteria | Rhizobiales |
| OTU0943 | Bacteria | Firmicutes | Bacilli | Bacillales |
| OTU0944 | Archaea | Euryarchaeota | Halobacteria | Halobacteriales |
| OTU0945 | Bacteria | Proteobacteria | Gammaproteobacteria | Pseudomonadales |
| OTU0946 | Bacteria | Proteobacteria | Gammaproteobacteria | Pseudomonadales |
| OTU0947 | Archaea | Crenarchaeota | Thermoprotei | Sulfolobales |
| OTU0948 | Bacteria | Proteobacteria | Gammaproteobacteria | Vibrionales |
| OTU0949 | Bacteria | Proteobacteria | Gammaproteobacteria | Xanthomonadales |
| OTU0950 | Bacteria | Planctomycetes | Planctomycetia | Pirellulales |
| OTU0951 | Bacteria | Actinobacteria | Actinobacteria | Corynebacteriales |
| OTU0952 | Bacteria | Actinobacteria | Acidimicrobiia | Acidimicrobiales |
| OTU0953 | Bacteria | GN02 | BD1-5 |  |
| OTU0954 | Bacteria | Planctomycetes | Phycisphaerae | Pla1 |
| OTU0955 | Bacteria | Proteobacteria | Alphaproteobacteria | Caulobacterales |
| OTU0956 | Bacteria | Firmicutes | Bacilli | Lactobacillales |
| OTU0957 | Bacteria | Actinobacteria | Acidimicrobiia | Acidimicrobiales |
| OTU0958 | Bacteria | Firmicutes | Bacilli | Lactobacillales |
| OTU0959 | Archaea | Euryarchaeota | Halobacteria | Halobacteriales |
| OTU0960 | Bacteria | Proteobacteria | Gammaproteobacteria | Vibrionales |
| OTU0961 | Archaea | Euryarchaeota | Halobacteria | Halobacteriales |
| OTU0962 | Archaea | Euryarchaeota | Halobacteria | Halobacteriales |
| OTU0963 | Bacteria | Firmicutes | Bacilli | Lactobacillales |
| OTU0964 | Bacteria | Proteobacteria | Gammaproteobacteria | Pseudomonadales |
| OTU0965 | Archaea | Crenarchaeota | Thermoprotei | Sulfolobales |
| OTU0966 | Bacteria | Proteobacteria | Deltaproteobacteria |  |
| OTU0967 | Bacteria | LD1 |  |  |
| OTU0968 | Bacteria | Firmicutes | Bacilli | Bacillales |
| OTU0969 | Bacteria | Proteobacteria |  |  |
| OTU0970 | Bacteria | Proteobacteria | Gammaproteobacteria | Vibrionales |
| OTU0971 | Bacteria | Proteobacteria | Gammaproteobacteria | Vibrionales |
| OTU0972 | Bacteria | Proteobacteria | Gammaproteobacteria | Pseudomonadales |
| OTU0973 | Bacteria | Proteobacteria | Alphaproteobacteria | Caulobacterales |
| OTU0974 | Bacteria | Bacteroidetes | Sphingobacteriia | Sphingobacteriales |
| OTU0975 | Bacteria | Proteobacteria | Gammaproteobacteria | Oceanospirillales |
| OTU0976 | Bacteria | Proteobacteria | Gammaproteobacteria | Vibrionales |
| OTU0977 | Bacteria | Proteobacteria | Gammaproteobacteria | Chromatiales |
| OTU0978 | Bacteria | Proteobacteria | Alphaproteobacteria | Rhizobiales |
| OTU0979 | Bacteria | Proteobacteria | Gammaproteobacteria | Pseudomonadales |
| OTU0980 | Bacteria | Actinobacteria | Actinobacteria | Micrococcales |
| OTU0981 | Bacteria | Firmicutes | Bacilli | Bacillales |
| OTU0982 | Bacteria | Planctomycetes | Planctomycetia | Planctomycetales |
| OTU0983 | Bacteria | Firmicutes | Bacilli | Lactobacillales |
| OTU0984 | Bacteria | Proteobacteria | Gammaproteobacteria | Pseudomonadales |
| OTU0985 | Archaea | Euryarchaeota | Halobacteria | Halobacteriales |
| OTU0986 | Bacteria | Proteobacteria | Betaproteobacteria | Burkholderiales |
| OTU0987 | Bacteria | Firmicutes | Bacilli | Lactobacillales |
| OTU0988 | Bacteria | Bacteroidetes | [Rhodothermi] | [Rhodothermales] |
| OTU0989 | Archaea | Crenarchaeota | MBGA |  |
| OTU0990 | Bacteria | Proteobacteria | Gammaproteobacteria | Pseudomonadales |
| OTU0991 | Archaea | Euryarchaeota | Halobacteria | Halobacteriales |
| OTU0992 | Bacteria | Actinobacteria | Actinobacteria | Corynebacteriales |
| OTU0993 | Archaea | Crenarchaeota | MBGB |  |
| OTU0994 | Bacteria | Proteobacteria | Gammaproteobacteria | Enterobacteriales |
| OTU0995 | Bacteria | Proteobacteria | Gammaproteobacteria | Pseudomonadales |
| OTU0996 | Bacteria | Firmicutes | Bacilli | Lactobacillales |
| OTU0997 | Bacteria | Proteobacteria | Gammaproteobacteria | Pseudomonadales |
| OTU0998 | Bacteria | Proteobacteria | Gammaproteobacteria | Enterobacteriales |
| OTU0999 | Bacteria | Firmicutes | Bacilli | Lactobacillales |
| OTU1000 | Bacteria | Firmicutes | Erysipelotrichi | Erysipelotrichales |
| OTU1001 | Bacteria | Firmicutes | Bacilli | Bacillales |
| OTU1002 | Archaea | Crenarchaeota | MBGB |  |
| OTU1003 | Bacteria | Proteobacteria | Gammaproteobacteria | Pseudomonadales |
| OTU1004 | Archaea | Euryarchaeota | Halobacteria | Halobacteriales |
| OTU1005 | Bacteria | Firmicutes | Bacilli | Bacillales |
| OTU1006 | Bacteria | Firmicutes | Bacilli | Lactobacillales |
| OTU1007 | Archaea | Euryarchaeota | Halobacteria | Halobacteriales |
| OTU1008 | Archaea | Euryarchaeota | Halobacteria | Halobacteriales |
| OTU1009 | Bacteria | Actinobacteria | Acidimicrobiia | Acidimicrobiales |
| OTU1010 | Bacteria | Chloroflexi | Thermomicrobia | JG30-KF-CM45 |
| OTU1011 | Archaea | Euryarchaeota | Halobacteria | Halobacteriales |
| OTU1012 | Archaea | Crenarchaeota | Thaumarchaeota | Cenarchaeales |
| OTU1013 | Archaea | Euryarchaeota | Halobacteria | Halobacteriales |
| OTU1014 | Bacteria | Proteobacteria | Gammaproteobacteria | Vibrionales |
| OTU1015 | Archaea | Euryarchaeota | Halobacteria | Halobacteriales |
| OTU1016 | Archaea | Euryarchaeota | Halobacteria | Halobacteriales |
| OTU1017 | Bacteria | Firmicutes | Bacilli | Bacillales |
| OTU1018 | Bacteria | Proteobacteria | Gammaproteobacteria | Xanthomonadales |
| OTU1019 | Bacteria | Firmicutes | Bacilli | Bacillales |
| OTU1020 | Archaea | Euryarchaeota | Halobacteria | Halobacteriales |
| OTU1021 | Archaea | Euryarchaeota | Halobacteria | Halobacteriales |
| OTU1022 | Bacteria | Proteobacteria | Gammaproteobacteria | Pseudomonadales |
| OTU1023 | Archaea | Euryarchaeota | Halobacteria | Halobacteriales |
| OTU1024 | Bacteria | Actinobacteria | Actinobacteria | Corynebacteriales |
| OTU1025 | Bacteria | Proteobacteria | Gammaproteobacteria | Vibrionales |
| OTU1026 | Archaea | Euryarchaeota | Halobacteria | Halobacteriales |
| OTU1027 | Archaea | Euryarchaeota | Halobacteria | Halobacteriales |
| OTU1028 | Bacteria | Actinobacteria | Actinobacteria | Corynebacteriales |
| OTU1029 | Bacteria |  |  |  |
| OTU1030 | Bacteria | Firmicutes | Bacilli | Bacillales |
| OTU1031 | Bacteria | Proteobacteria | Gammaproteobacteria | Vibrionales |
| OTU1032 | Bacteria | Proteobacteria | Gammaproteobacteria | Oceanospirillales |
| OTU1033 | Archaea | Crenarchaeota | Thaumarchaeota | Cenarchaeales |
| OTU1034 | Bacteria | Actinobacteria | Actinobacteria | Actinomycetales |
| OTU1035 | Archaea | Euryarchaeota | Halobacteria | Halobacteriales |
| OTU1036 | Bacteria | Proteobacteria | Gammaproteobacteria | Alteromonadales |
| OTU1037 | Archaea | Crenarchaeota | Thaumarchaeota | Cenarchaeales |
| OTU1038 | Bacteria | Proteobacteria | Gammaproteobacteria | Pseudomonadales |
| OTU1039 | Archaea | Euryarchaeota | Halobacteria | Halobacteriales |
| OTU1040 | Bacteria | Firmicutes | Bacilli | Bacillales |
| OTU1041 | Bacteria | Proteobacteria | Deltaproteobacteria | Desulfobacterales |
| OTU1042 | Archaea | Euryarchaeota | Halobacteria | Halobacteriales |
| OTU1043 | Bacteria | Firmicutes | Bacilli | Lactobacillales |
| OTU1044 | Bacteria | Proteobacteria | Gammaproteobacteria | Vibrionales |
| OTU1045 | Archaea | Euryarchaeota | Halobacteria | Halobacteriales |
| OTU1046 | Bacteria | Firmicutes | Bacilli | Bacillales |
| OTU1047 | Archaea | Euryarchaeota | Halobacteria | Halobacteriales |
| OTU1048 | Bacteria | Proteobacteria | Gammaproteobacteria |  |
| OTU1049 | Bacteria | Proteobacteria | Gammaproteobacteria | Alteromonadales |
| OTU1050 | Bacteria | Firmicutes | Bacilli | Bacillales |
| OTU1051 | Bacteria | Proteobacteria | Gammaproteobacteria | Vibrionales |
| OTU1052 | Bacteria | Proteobacteria | Alphaproteobacteria | Rickettsiales |
| OTU1053 | Bacteria | Firmicutes | Bacilli | Bacillales |
| OTU1054 | Archaea | Euryarchaeota | Halobacteria | Halobacteriales |
| OTU1055 | Bacteria | Firmicutes | Bacilli | Bacillales |
| OTU1056 | Bacteria | Proteobacteria | Gammaproteobacteria | Chromatiales |
| OTU1057 | Bacteria | Firmicutes | Bacilli | Bacillales |
| OTU1058 | Bacteria | Bacteroidetes | [Rhodothermi] | [Rhodothermales] |
| OTU1059 | Bacteria | Bacteroidetes | Bacteroidia | Bacteroidales |
| OTU1060 | Archaea | Euryarchaeota | Halobacteria | Halobacteriales |
| OTU1061 | Bacteria | Actinobacteria | Actinobacteria | Actinomycetales |
| OTU1062 | Bacteria | Proteobacteria | Gammaproteobacteria | Methylococcales |
| OTU1063 | Bacteria | Firmicutes | Bacilli | Bacillales |
| OTU1064 | Archaea | Euryarchaeota | Halobacteria | Halobacteriales |
| OTU1065 | Archaea | Euryarchaeota | Halobacteria | Halobacteriales |
| OTU1066 | Bacteria | Proteobacteria | Deltaproteobacteria | NB1-j |
| OTU1067 | Bacteria | Proteobacteria | Betaproteobacteria | Burkholderiales |
| OTU1068 | Bacteria | Actinobacteria | Actinobacteria | Micrococcales |
| OTU1069 | Bacteria | Bacteroidetes | Flavobacteriia | Flavobacteriales |
| OTU1070 | Archaea | Euryarchaeota | Halobacteria | Halobacteriales |
| OTU1071 | Bacteria | Firmicutes | Bacilli | Bacillales |
| OTU1072 | Bacteria | Firmicutes | Bacilli | Bacillales |
| OTU1073 | Bacteria | Firmicutes | Clostridia | Clostridiales |
| OTU1074 | Bacteria | Firmicutes | Bacilli | Bacillales |
| OTU1075 | Bacteria | Firmicutes | Bacilli | Bacillales |
| OTU1076 | Bacteria | Firmicutes | Bacilli | Lactobacillales |
| OTU1077 | Bacteria | Firmicutes | Clostridia | Clostridiales |
| OTU1078 | Archaea | Euryarchaeota | Halobacteria | Halobacteriales |
| OTU1079 | Bacteria | Proteobacteria | Gammaproteobacteria | Enterobacteriales |
| OTU1080 | Bacteria | Proteobacteria | Gammaproteobacteria | Pseudomonadales |
| OTU1081 | Archaea | Euryarchaeota | Halobacteria | Halobacteriales |
| OTU1082 | Bacteria | Firmicutes | Bacilli | Lactobacillales |
| OTU1083 | Archaea | Euryarchaeota | Halobacteria | Halobacteriales |
| OTU1084 | Bacteria | Planctomycetes | Planctomycetia | Pirellulales |
| OTU1085 | Archaea | Euryarchaeota | Halobacteria | Halobacteriales |
| OTU1086 | Archaea | Euryarchaeota | Halobacteria | Halobacteriales |
| OTU1087 | Bacteria | Proteobacteria | Gammaproteobacteria | Vibrionales |
| OTU1088 | Archaea | Crenarchaeota | Thaumarchaeota | Cenarchaeales |
| OTU1089 | Bacteria | Proteobacteria | Epsilonproteobacteria | Campylobacterales |
| OTU1090 | Bacteria | Firmicutes | Bacilli | Bacillales |
| OTU1091 | Bacteria | Firmicutes | Clostridia | Clostridiales |
| OTU1092 | Bacteria | Firmicutes | Bacilli | Bacillales |
| OTU1093 | Bacteria | Firmicutes | Bacilli | Bacillales |
| OTU1094 | Bacteria | Actinobacteria | Thermoleophilia | Solirubrobacterales |
| OTU1095 | Archaea | Euryarchaeota | Halobacteria | Halobacteriales |
| OTU1096 | Bacteria | Proteobacteria | Gammaproteobacteria | Chromatiales |
| OTU1097 | Bacteria | Bacteroidetes | [Rhodothermi] | [Rhodothermales] |
| OTU1098 | Bacteria | Firmicutes | Bacilli | Bacillales |
| OTU1099 | Bacteria | Proteobacteria | Betaproteobacteria | Burkholderiales |
| OTU1100 | Bacteria | Proteobacteria | Gammaproteobacteria | Vibrionales |
| OTU1101 | Bacteria | Spirochaetes | Spirochaetes | Spirochaetales |
| OTU1102 | Bacteria | Firmicutes | Bacilli | Bacillales |
| OTU1103 | Bacteria | Proteobacteria | Gammaproteobacteria | Alteromonadales |
| OTU1104 | Bacteria | Firmicutes | Bacilli | Lactobacillales |
| OTU1105 | Bacteria | Firmicutes | Bacilli | Bacillales |
| OTU1106 | Bacteria | Proteobacteria | Alphaproteobacteria | Rhodobacterales |
| OTU1107 | Bacteria | Proteobacteria | Gammaproteobacteria |  |
| OTU1108 | Bacteria | Chloroflexi | Anaerolineae | SHA-20 |
| OTU1109 | Archaea | Crenarchaeota | Thaumarchaeota | Cenarchaeales |
| OTU1110 | Bacteria | Proteobacteria | Betaproteobacteria |  |
| OTU1111 | Archaea | Euryarchaeota | Halobacteria | Halobacteriales |
| OTU1112 | Bacteria | Proteobacteria | Gammaproteobacteria | Alteromonadales |
| OTU1113 | Bacteria | Proteobacteria | Gammaproteobacteria | Pseudomonadales |
| OTU1114 | Archaea | Euryarchaeota | Halobacteria | Halobacteriales |
| OTU1115 | Archaea | Euryarchaeota | Halobacteria | Halobacteriales |
| OTU1116 | Bacteria | Proteobacteria | Gammaproteobacteria | Pseudomonadales |
| OTU1117 | Bacteria | Proteobacteria | Gammaproteobacteria | Enterobacteriales |
| OTU1118 | Bacteria | Proteobacteria | Gammaproteobacteria | Vibrionales |
| OTU1119 | Archaea | Euryarchaeota | Halobacteria | Halobacteriales |
| OTU1120 | Bacteria | Proteobacteria | Gammaproteobacteria | Aeromonadales |
| OTU1121 | Archaea | Euryarchaeota | Halobacteria | Halobacteriales |
| OTU1122 | Bacteria | Firmicutes | Bacilli |  |
| OTU1123 | Bacteria | Firmicutes | Bacilli | Lactobacillales |
| OTU1124 | Archaea | Crenarchaeota | Thaumarchaeota | Nitrososphaerales |
| OTU1125 | Bacteria | Proteobacteria | Gammaproteobacteria | Alteromonadales |
| OTU1126 | Archaea | Crenarchaeota | Thaumarchaeota | Nitrososphaerales |
| OTU1127 | Archaea | Euryarchaeota | Halobacteria | Halobacteriales |
| OTU1128 | Archaea | Euryarchaeota | Halobacteria | Halobacteriales |
| OTU1129 | Archaea | Crenarchaeota | Thaumarchaeota | Nitrososphaerales |
| OTU1130 | Bacteria | Proteobacteria | Gammaproteobacteria |  |
| OTU1131 | Bacteria | Firmicutes | Bacilli | Bacillales |
| OTU1132 | Bacteria | Proteobacteria | Gammaproteobacteria | Aeromonadales |
| OTU1133 | Bacteria | Firmicutes | Bacilli | Lactobacillales |
| OTU1134 | Bacteria | Firmicutes | Bacilli | Bacillales |
| OTU1135 | Bacteria | Proteobacteria | Gammaproteobacteria | Chromatiales |
| OTU1136 | Bacteria | Actinobacteria | Actinobacteria | Micrococcales |
| OTU1137 | Bacteria | Proteobacteria | Gammaproteobacteria | Pseudomonadales |
| OTU1138 | Archaea | Euryarchaeota | Halobacteria | Halobacteriales |
| OTU1139 | Bacteria | Firmicutes | Bacilli | Lactobacillales |
| OTU1140 | Bacteria | Bacteroidetes | Sphingobacteriia | Sphingobacteriales |
| OTU1141 | Bacteria | Proteobacteria | Gammaproteobacteria |  |
| OTU1142 | Bacteria | Proteobacteria | Gammaproteobacteria | Vibrionales |
| OTU1143 | Bacteria | Proteobacteria | Gammaproteobacteria | Oceanospirillales |
| OTU1144 | Archaea | Euryarchaeota | Halobacteria | Halobacteriales |
| OTU1145 | Bacteria | Proteobacteria | Gammaproteobacteria | Enterobacteriales |
| OTU1146 | Archaea | Euryarchaeota | Halobacteria | Halobacteriales |
| OTU1147 | Archaea | Euryarchaeota | Halobacteria | Halobacteriales |
| OTU1148 | Bacteria | Firmicutes | Bacilli | Bacillales |
| OTU1149 | Bacteria | Bacteroidetes | Bacteroidia | Bacteroidales |
| OTU1150 | Bacteria | Firmicutes | Bacilli | Lactobacillales |
| OTU1151 | Bacteria | Actinobacteria | Actinobacteria | Pseudonocardiales |
| OTU1152 | Bacteria | TM6 | SBRH58 |  |
| OTU1153 | Archaea | Crenarchaeota | MCG | pGrfC26 |
| OTU1154 | Bacteria | Proteobacteria | Gammaproteobacteria |  |
| OTU1155 | Bacteria | Firmicutes | Bacilli | Bacillales |
| OTU1156 | Bacteria | Aquificae | Aquificae | Aquificales |
| OTU1157 | Bacteria | Firmicutes | Bacilli | Bacillales |
| OTU1158 | Bacteria | Firmicutes | Clostridia | Clostridiales |
| OTU1159 | Bacteria | Proteobacteria | Betaproteobacteria | Burkholderiales |
| OTU1160 | Archaea | Euryarchaeota | Halobacteria | Halobacteriales |
| OTU1161 | Archaea | Euryarchaeota | Halobacteria | Halobacteriales |
| OTU1162 | Bacteria | Proteobacteria | Gammaproteobacteria | Enterobacteriales |
| OTU1163 | Bacteria | Firmicutes | Bacilli | Lactobacillales |
| OTU1164 | Bacteria | Proteobacteria | Deltaproteobacteria | GMD14H09 |
| OTU1165 | Bacteria | Firmicutes | Bacilli | Bacillales |
| OTU1166 | Archaea | Euryarchaeota | Halobacteria | Halobacteriales |
| OTU1167 | Archaea | Euryarchaeota | Halobacteria | Halobacteriales |
| OTU1168 | Bacteria | Proteobacteria | Gammaproteobacteria | Vibrionales |
| OTU1169 | Bacteria | Aquificae | Aquificae | Aquificales |
| OTU1170 | Archaea | Euryarchaeota | Halobacteria | Halobacteriales |
| OTU1171 | Archaea | Euryarchaeota | Halobacteria | Halobacteriales |
| OTU1172 | Bacteria | Proteobacteria | Gammaproteobacteria | Pseudomonadales |
| OTU1173 | Archaea | Euryarchaeota | Halobacteria | Halobacteriales |
| OTU1174 | Archaea | Euryarchaeota | Halobacteria | Halobacteriales |
| OTU1175 | Bacteria | Proteobacteria | Gammaproteobacteria | Pseudomonadales |
| OTU1176 | Bacteria | Proteobacteria | Gammaproteobacteria | Aeromonadales |
| OTU1177 | Bacteria | Proteobacteria | Gammaproteobacteria | Pseudomonadales |
| OTU1178 | Bacteria | Aquificae | Aquificae | Aquificales |
| OTU1179 | Bacteria | Proteobacteria | Alphaproteobacteria | Rhodobacterales |
| OTU1180 | Bacteria | Proteobacteria | Gammaproteobacteria | Vibrionales |
| OTU1181 | Bacteria | Aquificae | Aquificae | Aquificales |
| OTU1182 | Bacteria | Actinobacteria | Actinobacteria | Actinomycetales |
| OTU1183 | Bacteria | Firmicutes | Bacilli | Bacillales |
| OTU1184 | Bacteria | Proteobacteria | Gammaproteobacteria | Vibrionales |
| OTU1185 | Bacteria | Proteobacteria | Gammaproteobacteria | Alteromonadales |
| OTU1186 | Bacteria | Bacteroidetes | Flavobacteriia | Flavobacteriales |
| OTU1187 | Bacteria | Proteobacteria | Betaproteobacteria | Burkholderiales |
| OTU1188 | Bacteria | Proteobacteria | Gammaproteobacteria | Pseudomonadales |
| OTU1189 | Archaea | Euryarchaeota | Halobacteria | Halobacteriales |
| OTU1190 | Bacteria | Proteobacteria | Gammaproteobacteria | Oceanospirillales |
| OTU1191 | Bacteria | Firmicutes | Bacilli | Bacillales |
| OTU1192 | Bacteria | Proteobacteria | Gammaproteobacteria |  |
| OTU1193 | Bacteria | Bacteroidetes | Bacteroidia | Bacteroidales |
| OTU1194 | Bacteria | Actinobacteria | Actinobacteria | Actinomycetales |
| OTU1195 | Bacteria | Proteobacteria | Gammaproteobacteria | Aeromonadales |
| OTU1196 | Bacteria | Proteobacteria | Gammaproteobacteria | Pseudomonadales |
| OTU1197 | Bacteria | Aquificae | Aquificae | Aquificales |
| OTU1198 | Archaea | Euryarchaeota | Halobacteria | Halobacteriales |
| OTU1199 | Bacteria | Fusobacteria | Fusobacteriia | Fusobacteriales |
| OTU1200 | Archaea | Euryarchaeota | Halobacteria | Halobacteriales |
| OTU1201 | Bacteria | Proteobacteria | Deltaproteobacteria | Desulfobacterales |
| OTU1202 | Bacteria | Thermotogae | Thermotogae | Thermotogales |
| OTU1203 | Bacteria | Actinobacteria | Actinobacteria | Micrococcales |
| OTU1204 | Bacteria | Thermotogae | Thermotogae | Thermotogales |
| OTU1205 | Bacteria | Proteobacteria | Alphaproteobacteria | Rhizobiales |
| OTU1206 | Archaea | Euryarchaeota | Halobacteria | Halobacteriales |
| OTU1207 | Archaea | Euryarchaeota | Halobacteria | Halobacteriales |
| OTU1208 | Bacteria | Proteobacteria | Gammaproteobacteria | Pseudomonadales |
| OTU1209 | Bacteria | Thermotogae | Thermotogae | Thermotogales |
| OTU1210 | Archaea | Euryarchaeota | Halobacteria | Halobacteriales |
| OTU1211 | Unclassified |  |  |  |
| OTU1212 | Bacteria | Proteobacteria | Gammaproteobacteria | Vibrionales |
| OTU1213 | Bacteria | Firmicutes | Clostridia | Clostridiales |
| OTU1214 | Bacteria | Proteobacteria | Betaproteobacteria | Burkholderiales |
| OTU1215 | Bacteria | Proteobacteria | Gammaproteobacteria | Vibrionales |
| OTU1216 | Bacteria | Proteobacteria | Alphaproteobacteria | Rhizobiales |
| OTU1217 | Archaea | Euryarchaeota | Halobacteria | Halobacteriales |
| OTU1218 | Bacteria | Proteobacteria | Gammaproteobacteria | Pseudomonadales |
| OTU1219 | Bacteria | Actinobacteria | Actinobacteria | Micrococcales |
| OTU1220 | Archaea | Euryarchaeota | Halobacteria | Halobacteriales |
| OTU1221 | Archaea | Euryarchaeota | Halobacteria | Halobacteriales |
| OTU1222 | Archaea | Euryarchaeota | Halobacteria | Halobacteriales |
| OTU1223 | Bacteria | Actinobacteria | Actinobacteria | Corynebacteriales |
| OTU1224 | Bacteria | Proteobacteria | Gammaproteobacteria | Aeromonadales |
| OTU1225 | Archaea | Euryarchaeota | Halobacteria | Halobacteriales |
| OTU1226 | Archaea | Euryarchaeota | Halobacteria | Halobacteriales |
| OTU1227 | Archaea | Euryarchaeota | Halobacteria | Halobacteriales |
| OTU1228 | Bacteria | Bacteroidetes | Sphingobacteriia | Sphingobacteriales |
| OTU1229 | Archaea | Euryarchaeota | Halobacteria | Halobacteriales |
| OTU1230 | Bacteria | Proteobacteria | Alphaproteobacteria | Rhizobiales |
| OTU1231 | Bacteria | Firmicutes | Clostridia | Thermoanaerobacterales |
| OTU1232 | Bacteria | EM19 |  |  |
| OTU1233 | Bacteria | Actinobacteria | Actinobacteria | Micrococcales |
| OTU1234 | Bacteria | Proteobacteria | Gammaproteobacteria | Enterobacteriales |
| OTU1235 | Bacteria | Proteobacteria | Gammaproteobacteria | Oceanospirillales |
| OTU1236 | Bacteria | Firmicutes | Bacilli | Bacillales |
| OTU1237 | Bacteria | Proteobacteria | Gammaproteobacteria | Alteromonadales |
| OTU1238 | Bacteria | Proteobacteria | Gammaproteobacteria | Vibrionales |
| OTU1239 | Bacteria | Proteobacteria | Gammaproteobacteria | Vibrionales |
| OTU1240 | Archaea | Euryarchaeota | Halobacteria | Halobacteriales |
| OTU1241 | Bacteria | Proteobacteria | Gammaproteobacteria | Pseudomonadales |
| OTU1242 | Bacteria | Proteobacteria | Gammaproteobacteria | Enterobacteriales |
| OTU1243 | Bacteria | Firmicutes | Erysipelotrichi | Erysipelotrichales |
| OTU1244 | Archaea | Euryarchaeota | Halobacteria | Halobacteriales |
| OTU1245 | Bacteria | Bacteroidetes | Sphingobacteriia | Sphingobacteriales |
| OTU1246 | Bacteria | Proteobacteria | Gammaproteobacteria | Pseudomonadales |
| OTU1247 | Bacteria | Proteobacteria | Alphaproteobacteria | Rhodobacterales |
| OTU1248 | Bacteria | Proteobacteria | Gammaproteobacteria | Pseudomonadales |
| OTU1249 | Bacteria | Firmicutes | Bacilli | Bacillales |
| OTU1250 | Bacteria | Proteobacteria | Gammaproteobacteria | Pseudomonadales |
| OTU1251 | Bacteria | Proteobacteria | Gammaproteobacteria | Vibrionales |
| OTU1252 | Bacteria | Proteobacteria | Gammaproteobacteria | Vibrionales |
| OTU1253 | Bacteria | Firmicutes | Bacilli | Lactobacillales |
| OTU1254 | Bacteria | Proteobacteria | Gammaproteobacteria | Alteromonadales |
| OTU1255 | Archaea | Euryarchaeota | Halobacteria | Halobacteriales |
| OTU1256 | Bacteria | Chloroflexi | Chloroflexi | Chloroflexales |
| OTU1257 | Archaea | Euryarchaeota | Halobacteria | Halobacteriales |
| OTU1258 | Bacteria | Proteobacteria | Gammaproteobacteria | Pseudomonadales |
| OTU1259 | Bacteria | Proteobacteria | Gammaproteobacteria | Enterobacteriales |
| OTU1260 | Bacteria | Chloroflexi | Chloroflexi | [Roseiflexales] |
| OTU1261 | Bacteria | Actinobacteria | Actinobacteria | Micrococcales |
| OTU1262 | Archaea | Euryarchaeota | Halobacteria | Halobacteriales |
| OTU1263 | Bacteria | Proteobacteria | Gammaproteobacteria | Vibrionales |
| OTU1264 | Bacteria | Firmicutes | Bacilli | Bacillales |
| OTU1265 | Bacteria | Firmicutes | Bacilli | Bacillales |
| OTU1266 | Bacteria | Chloroflexi | Anaerolineae | SJA-15 |
| OTU1267 | Archaea | Euryarchaeota | Halobacteria | Halobacteriales |
| OTU1268 | Archaea | Euryarchaeota | Halobacteria | Halobacteriales |
| OTU1269 | Archaea | Euryarchaeota | Halobacteria | Halobacteriales |
| OTU1270 | Bacteria | Proteobacteria | Gammaproteobacteria |  |
| OTU1271 | Bacteria | Proteobacteria | Gammaproteobacteria | Alteromonadales |
| OTU1272 | Bacteria | Firmicutes | Bacilli | Bacillales |
| OTU1273 | Archaea | Euryarchaeota | Halobacteria | Halobacteriales |
| OTU1274 | Bacteria | Chloroflexi | Anaerolineae | GCA004 |
| OTU1275 | Archaea | Euryarchaeota | Halobacteria | Halobacteriales |
| OTU1276 | Bacteria | Proteobacteria | Alphaproteobacteria | Sphingomonadales |
| OTU1277 | Bacteria | Proteobacteria | Gammaproteobacteria | Oceanospirillales |
| OTU1278 | Bacteria | Proteobacteria | Gammaproteobacteria | Vibrionales |
| OTU1279 | Bacteria | Firmicutes | Bacilli | Lactobacillales |
| OTU1280 | Bacteria | Proteobacteria | Gammaproteobacteria |  |
| OTU1281 | Bacteria | Proteobacteria | Alphaproteobacteria |  |
| OTU1282 | Bacteria | Proteobacteria | Gammaproteobacteria | Enterobacteriales |
| OTU1283 | Bacteria | Proteobacteria | Gammaproteobacteria | Vibrionales |
| OTU1284 | Bacteria | Proteobacteria | Gammaproteobacteria | Vibrionales |
| OTU1285 | Bacteria | Proteobacteria | Alphaproteobacteria | Sphingomonadales |
| OTU1286 | Archaea | Euryarchaeota | Halobacteria | Halobacteriales |
| OTU1287 | Archaea | Euryarchaeota | Halobacteria | Halobacteriales |
| OTU1288 | Archaea | Euryarchaeota | Halobacteria | Halobacteriales |
| OTU1289 | Bacteria | Proteobacteria | Gammaproteobacteria | Pseudomonadales |
| OTU1290 | Bacteria | Proteobacteria | Deltaproteobacteria | Desulfobacterales |
| OTU1291 | Bacteria | Proteobacteria | Gammaproteobacteria | Vibrionales |
| OTU1292 | Archaea | Euryarchaeota | Halobacteria | Halobacteriales |
| OTU1293 | Bacteria | Proteobacteria | Gammaproteobacteria | Pseudomonadales |
| OTU1294 | Bacteria | Bacteroidetes | Flavobacteriia | Flavobacteriales |
| OTU1295 | Bacteria | Firmicutes | Bacilli | Lactobacillales |
| OTU1296 | Bacteria | Firmicutes | Bacilli | Bacillales |
| OTU1297 | Bacteria | Proteobacteria | Gammaproteobacteria | Pseudomonadales |
| OTU1298 | Archaea | Euryarchaeota | Halobacteria | Halobacteriales |
| OTU1299 | Bacteria | Proteobacteria | Gammaproteobacteria | Vibrionales |
| OTU1300 | Bacteria | Proteobacteria | Gammaproteobacteria | Pseudomonadales |
| OTU1301 | Bacteria | Proteobacteria | Gammaproteobacteria | Aeromonadales |
| OTU1302 | Archaea | Euryarchaeota | Halobacteria | Halobacteriales |
| OTU1303 | Archaea | Euryarchaeota | Halobacteria | Halobacteriales |
| OTU1304 | Bacteria | Proteobacteria | Gammaproteobacteria | Alteromonadales |
| OTU1305 | Bacteria | Proteobacteria | Gammaproteobacteria | Alteromonadales |
| OTU1306 | Archaea | Euryarchaeota | Halobacteria | Halobacteriales |
| OTU1307 | Bacteria | Proteobacteria | Gammaproteobacteria | Vibrionales |
| OTU1308 | Bacteria | Firmicutes | Bacilli | Bacillales |
| OTU1309 | Archaea | Euryarchaeota | Halobacteria | Halobacteriales |
| OTU1310 | Bacteria | Actinobacteria | Actinobacteria | Corynebacteriales |
| OTU1311 | Bacteria | Firmicutes | Bacilli | Bacillales |
| OTU1312 | Bacteria | Firmicutes | Bacilli | Bacillales |
| OTU1313 | Bacteria | Proteobacteria | Alphaproteobacteria | Rhizobiales |
| OTU1314 | Bacteria | Firmicutes | Bacilli | Bacillales |
| OTU1315 | Archaea | Euryarchaeota | Halobacteria | Halobacteriales |
| OTU1316 | Bacteria | Firmicutes | Bacilli | Lactobacillales |
| OTU1317 | Bacteria | Proteobacteria | Gammaproteobacteria | Alteromonadales |
| OTU1318 | Archaea | Euryarchaeota | Halobacteria | Halobacteriales |
| OTU1319 | Bacteria | Firmicutes | Bacilli | Lactobacillales |
| OTU1320 | Bacteria | Proteobacteria | Gammaproteobacteria | Chromatiales |
| OTU1321 | Bacteria | Firmicutes | Clostridia | Halanaerobiales |
| OTU1322 | Bacteria | Proteobacteria | Gammaproteobacteria | Vibrionales |
| OTU1323 | Bacteria | Proteobacteria | Betaproteobacteria | Burkholderiales |
| OTU1324 | Bacteria | Proteobacteria | Deltaproteobacteria | NB1-j |
| OTU1325 | Bacteria | Firmicutes | Bacilli | Bacillales |
| OTU1326 | Archaea | Euryarchaeota | Halobacteria | Halobacteriales |
| OTU1327 | Bacteria | Proteobacteria | Gammaproteobacteria | Vibrionales |
| OTU1328 | Bacteria | Proteobacteria | Gammaproteobacteria | Vibrionales |
| OTU1329 | Bacteria | Firmicutes | Clostridia | Clostridiales |
| OTU1330 | Bacteria | Proteobacteria | Gammaproteobacteria |  |
| OTU1331 | Bacteria | Firmicutes | Bacilli | Bacillales |
| OTU1332 | Bacteria | Firmicutes | Bacilli | Bacillales |
| OTU1333 | Bacteria | Proteobacteria | Gammaproteobacteria | Alteromonadales |
| OTU1334 | Archaea | Euryarchaeota | Halobacteria | Halobacteriales |
| OTU1335 | Bacteria | Proteobacteria | Alphaproteobacteria |  |
| OTU1336 | Bacteria | Firmicutes | Bacilli | Lactobacillales |
| OTU1337 | Bacteria | Bacteroidetes | Flavobacteriia | Flavobacteriales |
| OTU1338 | Bacteria | Proteobacteria | Gammaproteobacteria | Pseudomonadales |
| OTU1339 | Bacteria | Proteobacteria | Gammaproteobacteria | Vibrionales |
| OTU1340 | Bacteria | Firmicutes | Bacilli | Bacillales |
| OTU1341 | Bacteria | Proteobacteria | Gammaproteobacteria | Oceanospirillales |
| OTU1342 | Bacteria | Firmicutes | Bacilli | Lactobacillales |
| OTU1343 | Bacteria | Firmicutes | Bacilli | Bacillales |
| OTU1344 | Bacteria | Proteobacteria | Gammaproteobacteria | Vibrionales |
| OTU1345 | Bacteria | Firmicutes | Bacilli | Bacillales |
| OTU1346 | Archaea | Euryarchaeota | Halobacteria | Halobacteriales |
| OTU1347 | Bacteria | Fusobacteria | Fusobacteriia | Fusobacteriales |
| OTU1348 | Archaea | Euryarchaeota | Halobacteria | Halobacteriales |
| OTU1349 | Bacteria | Firmicutes | Bacilli | Bacillales |
| OTU1350 | Bacteria | Proteobacteria | Gammaproteobacteria | Enterobacteriales |
| OTU1351 | Archaea | Euryarchaeota | Halobacteria | Halobacteriales |
| OTU1352 | Bacteria | Proteobacteria | Gammaproteobacteria | Xanthomonadales |
| OTU1353 | Bacteria | Proteobacteria | Gammaproteobacteria | Vibrionales |
| OTU1354 | Bacteria | Bacteroidetes | Flavobacteriia | Flavobacteriales |
| OTU1355 | Bacteria | Proteobacteria | Alphaproteobacteria | Rhizobiales |
| OTU1356 | Bacteria | Proteobacteria | Gammaproteobacteria | Vibrionales |
| OTU1357 | Bacteria | Firmicutes | Bacilli | Lactobacillales |
| OTU1358 | Bacteria | Proteobacteria | Gammaproteobacteria |  |
| OTU1359 | Bacteria | Proteobacteria | Gammaproteobacteria | Pseudomonadales |
| OTU1360 | Bacteria | Proteobacteria | Gammaproteobacteria | Vibrionales |
| OTU1361 | Bacteria | Firmicutes | Clostridia | Clostridiales |
| OTU1362 | Bacteria | Firmicutes | Bacilli | Lactobacillales |
| OTU1363 | Bacteria | Proteobacteria | Alphaproteobacteria | Sphingomonadales |
| OTU1364 | Archaea | Euryarchaeota | Halobacteria | Halobacteriales |
| OTU1365 | Archaea | Euryarchaeota | Halobacteria | Halobacteriales |
| OTU1366 | Bacteria | Proteobacteria | Gammaproteobacteria | Vibrionales |
| OTU1367 | Bacteria | Proteobacteria | Deltaproteobacteria | Desulfarculales |
| OTU1368 | Bacteria | Proteobacteria | Gammaproteobacteria |  |
| OTU1369 | Bacteria | Firmicutes | Clostridia | Clostridiales |
| OTU1370 | Bacteria | Proteobacteria | Gammaproteobacteria | Pseudomonadales |
| OTU1371 | Archaea | Euryarchaeota | Halobacteria | Halobacteriales |
| OTU1372 | Bacteria | Firmicutes | Bacilli | Bacillales |
| OTU1373 | Archaea | Euryarchaeota | Halobacteria | Halobacteriales |
| OTU1374 | Bacteria | [Thermi] | Deinococci | Thermales |
| OTU1375 | Archaea | Euryarchaeota | Halobacteria | Halobacteriales |
| OTU1376 | Bacteria | Proteobacteria | Deltaproteobacteria | Desulfobacterales |
| OTU1377 | Bacteria | Bacteroidetes | Flavobacteriia | Flavobacteriales |
| OTU1378 | Bacteria | Actinobacteria | Acidimicrobiia | Acidimicrobiales |
| OTU1379 | Archaea | Euryarchaeota | Halobacteria | Halobacteriales |
| OTU1380 | Bacteria | Proteobacteria | Alphaproteobacteria | Rhizobiales |
| OTU1381 | Bacteria | Bacteroidetes | Bacteroidia | Bacteroidales |
| OTU1382 | Bacteria | Firmicutes | Clostridia | Clostridiales |
| OTU1383 | Bacteria | [Thermi] | Deinococci | Thermales |
| OTU1384 | Archaea | Euryarchaeota | Halobacteria | Halobacteriales |
| OTU1385 | Bacteria | Proteobacteria | Gammaproteobacteria | Vibrionales |
| OTU1386 | Archaea | Euryarchaeota | Halobacteria | Halobacteriales |
| OTU1387 | Archaea | Euryarchaeota | Halobacteria | Halobacteriales |
| OTU1388 | Bacteria | Proteobacteria | Gammaproteobacteria | Alteromonadales |
| OTU1389 | Bacteria | [Thermi] | Deinococci | Deinococcales |
| OTU1390 | Bacteria | Proteobacteria | Gammaproteobacteria | Vibrionales |
| OTU1391 | Bacteria | Actinobacteria | Actinobacteria | Corynebacteriales |
| OTU1392 | Bacteria | Proteobacteria | Gammaproteobacteria | Vibrionales |
| OTU1393 | Bacteria | Firmicutes | Bacilli | Bacillales |
| OTU1394 | Archaea | Euryarchaeota | Halobacteria | Halobacteriales |
| OTU1395 | Bacteria | Proteobacteria | Gammaproteobacteria | Vibrionales |
| OTU1396 | Bacteria | Actinobacteria | Actinobacteria | Corynebacteriales |
| OTU1397 | Archaea | Euryarchaeota | Halobacteria | Halobacteriales |
| OTU1398 | Bacteria | [Thermi] | Deinococci | Deinococcales |
| OTU1399 | Bacteria | Firmicutes | Bacilli | Bacillales |
| OTU1400 | Bacteria | Proteobacteria | Gammaproteobacteria | Vibrionales |
| OTU1401 | Bacteria | [Thermi] | Deinococci | Deinococcales |
| OTU1402 | Bacteria | Firmicutes | Bacilli | Bacillales |
| OTU1403 | Bacteria | Firmicutes | Bacilli | Bacillales |
| OTU1404 | Bacteria | Proteobacteria | Gammaproteobacteria | Pseudomonadales |
| OTU1405 | Archaea | Euryarchaeota | Methanobacteria | Methanobacteriales |
| OTU1406 | Archaea | Euryarchaeota | Halobacteria | Halobacteriales |
| OTU1407 | Bacteria | Firmicutes | Bacilli | Bacillales |
| OTU1408 | Bacteria | Armatimonadetes | OPB50 |  |
| OTU1409 | Bacteria | Armatimonadetes | SJA-176 | RB046 |
| OTU1410 | Bacteria | Armatimonadetes | SJA-176 | TP122 |
| OTU1411 | Bacteria | Armatimonadetes | SJA-176 | GAB-B06 |
| OTU1412 | Archaea | Euryarchaeota | Halobacteria | Halobacteriales |
| OTU1413 | Bacteria | Proteobacteria | Gammaproteobacteria | Vibrionales |
| OTU1414 | Bacteria | Armatimonadetes | Chthonomonadetes | SJA-22 |
| OTU1415 | Bacteria | Bacteroidetes | Flavobacteriia | Flavobacteriales |
| OTU1416 | Bacteria | Firmicutes | Bacilli | Bacillales |
| OTU1417 | Archaea | Euryarchaeota | Halobacteria | Halobacteriales |
| OTU1418 | Bacteria | Proteobacteria | Gammaproteobacteria | Vibrionales |
| OTU1419 | Bacteria | Proteobacteria | Gammaproteobacteria | Enterobacteriales |
| OTU1420 | Bacteria | Proteobacteria | Gammaproteobacteria | Pseudomonadales |
| OTU1421 | Bacteria | Armatimonadetes | [Fimbriimonadia] | [Fimbriimonadales] |
| OTU1422 | Bacteria | Actinobacteria | Coriobacteriia | Coriobacteriales |
| OTU1423 | Archaea | Euryarchaeota | Halobacteria | Halobacteriales |
| OTU1424 | Bacteria | Bacteroidetes | [Rhodothermi] | [Rhodothermales] |
| OTU1425 | Bacteria | Firmicutes | Clostridia | Clostridiales |
| OTU1426 | Bacteria | Proteobacteria | Gammaproteobacteria | Vibrionales |
| OTU1427 | Bacteria | Proteobacteria | Gammaproteobacteria |  |
| OTU1428 | Bacteria | Nitrospirae | Nitrospira | Nitrospirales |
| OTU1429 | Bacteria | Proteobacteria | Gammaproteobacteria | Oceanospirillales |
| OTU1430 | Bacteria | Firmicutes | Bacilli | Lactobacillales |
| OTU1431 | Bacteria | Proteobacteria | Gammaproteobacteria | Vibrionales |
| OTU1432 | Bacteria | Firmicutes | Bacilli | Bacillales |
| OTU1433 | Bacteria | Firmicutes | Bacilli | Bacillales |
| OTU1434 | Archaea | Euryarchaeota | Halobacteria | Halobacteriales |
| OTU1435 | Archaea | Euryarchaeota | Halobacteria | Halobacteriales |
| OTU1436 | Archaea | Euryarchaeota | Halobacteria | Halobacteriales |
| OTU1437 | Archaea | Euryarchaeota | Halobacteria | Halobacteriales |
| OTU1438 | Bacteria | Bacteroidetes | [Rhodothermi] | [Rhodothermales] |
| OTU1439 | Bacteria | Proteobacteria | Gammaproteobacteria | Pseudomonadales |
| OTU1440 | Bacteria | Proteobacteria | Gammaproteobacteria | Enterobacteriales |
| OTU1441 | Bacteria | Proteobacteria | Gammaproteobacteria | Pseudomonadales |
| OTU1442 | Bacteria | Proteobacteria | Gammaproteobacteria | Pseudomonadales |
| OTU1443 | Bacteria | Proteobacteria | Gammaproteobacteria | Pseudomonadales |
| OTU1444 | Bacteria | Firmicutes | Bacilli | Bacillales |
| OTU1445 | Bacteria | Proteobacteria | Gammaproteobacteria |  |
| OTU1446 | Bacteria | Firmicutes | Bacilli | Lactobacillales |
| OTU1447 | Archaea | Euryarchaeota | Halobacteria | Halobacteriales |
| OTU1448 | Bacteria | Proteobacteria | Gammaproteobacteria | Pseudomonadales |
| OTU1449 | Archaea | Euryarchaeota | Halobacteria | Halobacteriales |
| OTU1450 | Archaea | Euryarchaeota | Halobacteria | Halobacteriales |
| OTU1451 | Bacteria | Firmicutes | Bacilli | Lactobacillales |
| OTU1452 | Bacteria | Nitrospirae | Nitrospira | Nitrospirales |
| OTU1453 | Bacteria | Proteobacteria | Gammaproteobacteria |  |
| OTU1454 | Bacteria | Proteobacteria | Alphaproteobacteria | Caulobacterales |
| OTU1455 | Bacteria | Actinobacteria | Actinobacteria | Corynebacteriales |
| OTU1456 | Bacteria | Proteobacteria | Gammaproteobacteria | Pseudomonadales |
| OTU1457 | Archaea | Euryarchaeota | Halobacteria | Halobacteriales |
| OTU1458 | Bacteria | Actinobacteria | Actinobacteria | Micrococcales |
| OTU1459 | Archaea | Euryarchaeota | Halobacteria | Halobacteriales |
| OTU1460 | Bacteria | Firmicutes | Clostridia | Clostridiales |
| OTU1461 | Bacteria | Proteobacteria | Gammaproteobacteria | Pseudomonadales |
| OTU1462 | Archaea | Euryarchaeota | Halobacteria | Halobacteriales |
| OTU1463 | Archaea | Euryarchaeota | Halobacteria | Halobacteriales |
| OTU1464 | Bacteria | Bacteroidetes | Bacteroidia | Bacteroidales |
| OTU1465 | Bacteria | Proteobacteria | Gammaproteobacteria | Alteromonadales |
| OTU1466 | Bacteria | Proteobacteria | Gammaproteobacteria | Pseudomonadales |
| OTU1467 | Bacteria | Proteobacteria | Gammaproteobacteria | Pseudomonadales |
| OTU1468 | Bacteria | Bacteroidetes | Flavobacteriia | Flavobacteriales |
| OTU1469 | Bacteria | Actinobacteria | Actinobacteria | Micrococcales |
| OTU1470 | Bacteria | Nitrospirae | Nitrospira | Nitrospirales |
| OTU1471 | Bacteria | Bacteroidetes | Bacteroidia | Bacteroidales |
| OTU1472 | Bacteria | Firmicutes | Bacilli | Bacillales |
| OTU1473 | Bacteria | Firmicutes | Bacilli | Lactobacillales |
| OTU1474 | Bacteria | Proteobacteria | Gammaproteobacteria | Vibrionales |
| OTU1475 | Bacteria | Firmicutes | Bacilli | Lactobacillales |
| OTU1476 | Archaea | Euryarchaeota | Halobacteria | Halobacteriales |
| OTU1477 | Bacteria | Firmicutes | Bacilli | Bacillales |
| OTU1478 | Archaea | Euryarchaeota | Halobacteria | Halobacteriales |
| OTU1479 | Bacteria | Proteobacteria | Gammaproteobacteria | Vibrionales |
| OTU1480 | Bacteria | Proteobacteria | Gammaproteobacteria | Pseudomonadales |
| OTU1481 | Archaea | Euryarchaeota | Halobacteria | Halobacteriales |
| OTU1482 | Archaea | Euryarchaeota | Halobacteria | Halobacteriales |
| OTU1483 | Bacteria | Proteobacteria | Betaproteobacteria | Burkholderiales |
| OTU1484 | Bacteria | Proteobacteria | Gammaproteobacteria | Pseudomonadales |
| OTU1485 | Bacteria | Proteobacteria | Gammaproteobacteria | Oceanospirillales |
| OTU1486 | Bacteria |  |  |  |
| OTU1487 | Bacteria | Planctomycetes | Phycisphaerae | WD2101 |
| OTU1488 | Bacteria | Proteobacteria | Gammaproteobacteria | Pseudomonadales |
| OTU1489 | Bacteria | Proteobacteria | Gammaproteobacteria | Xanthomonadales |
| OTU1490 | Archaea | Euryarchaeota | Halobacteria | Halobacteriales |
| OTU1491 | Archaea | Euryarchaeota | Halobacteria | Halobacteriales |
| OTU1492 | Bacteria | Bacteroidetes | Flavobacteriia | Flavobacteriales |
| OTU1493 | Bacteria | Firmicutes | Bacilli | Bacillales |
| OTU1494 | Bacteria | Proteobacteria | Gammaproteobacteria | Pseudomonadales |
| OTU1495 | Archaea | Euryarchaeota | Halobacteria | Halobacteriales |
| OTU1496 | Archaea | Euryarchaeota | Halobacteria | Halobacteriales |
| OTU1497 | Bacteria | Proteobacteria | Gammaproteobacteria | Pseudomonadales |
| OTU1498 | Bacteria | Firmicutes | Clostridia | Clostridiales |
| OTU1499 | Bacteria | Proteobacteria | Alphaproteobacteria | Rhodospirillales |
| OTU1500 | Bacteria | Proteobacteria | Gammaproteobacteria | Pseudomonadales |
| OTU1501 | Archaea | Euryarchaeota | Halobacteria | Halobacteriales |
| OTU1502 | Bacteria | Proteobacteria | Gammaproteobacteria | Pseudomonadales |
| OTU1503 | Bacteria | Firmicutes | Bacilli | Lactobacillales |
| OTU1504 | Bacteria | Proteobacteria | Gammaproteobacteria | Alteromonadales |
| OTU1505 | Bacteria | Actinobacteria | Actinobacteria | Micrococcales |
| OTU1506 | Bacteria | Firmicutes | Clostridia | Clostridiales |
| OTU1507 | Bacteria | Nitrospirae | Nitrospira | Nitrospirales |
| OTU1508 | Bacteria | Nitrospirae | Nitrospira | Nitrospirales |
| OTU1509 | Bacteria | Proteobacteria | Betaproteobacteria | Burkholderiales |
| OTU1510 | Bacteria | OP8 | OP8_2 |  |
| OTU1511 | Archaea | Euryarchaeota | Halobacteria | Halobacteriales |
| OTU1512 | Bacteria | Firmicutes | Bacilli | Bacillales |
| OTU1513 | Bacteria | Proteobacteria | Gammaproteobacteria | Salinisphaerales |
| OTU1514 | Bacteria | Firmicutes | Bacilli | Bacillales |
| OTU1515 | Archaea | Euryarchaeota | Halobacteria | Halobacteriales |
| OTU1516 | Bacteria | Actinobacteria | Actinobacteria | Micrococcales |
| OTU1517 | Bacteria | Verrucomicrobia | Verruco-5 | WCHB1-41 |
| OTU1518 | Bacteria | Firmicutes | Bacilli | Bacillales |
| OTU1519 | Bacteria | Verrucomicrobia | Verruco-5 | WCHB1-41 |
| OTU1520 | Bacteria | Lentisphaerae | [Lentisphaeria] | Victivallales |
| OTU1521 | Bacteria | Firmicutes | Bacilli | Lactobacillales |
| OTU1522 | Bacteria | Proteobacteria | Gammaproteobacteria | Oceanospirillales |
| OTU1523 | Archaea | Euryarchaeota | Halobacteria | Halobacteriales |
| OTU1524 | Bacteria | Proteobacteria | Gammaproteobacteria | Pseudomonadales |
| OTU1525 | Bacteria | Proteobacteria | Gammaproteobacteria | Vibrionales |
| OTU1526 | Bacteria | Verrucomicrobia | Verruco-5 | WCHB1-41 |
| OTU1527 | Bacteria | Proteobacteria | Gammaproteobacteria | Alteromonadales |
| OTU1528 | Bacteria | Proteobacteria | Gammaproteobacteria | Pseudomonadales |
| OTU1529 | Bacteria | Proteobacteria | Gammaproteobacteria | Alteromonadales |
| OTU1530 | Bacteria | Proteobacteria | Alphaproteobacteria | Rhizobiales |
| OTU1531 | Bacteria | Proteobacteria | Betaproteobacteria | Burkholderiales |
| OTU1532 | Bacteria | Proteobacteria | Gammaproteobacteria | Pseudomonadales |
| OTU1533 | Archaea | Euryarchaeota | Halobacteria | Halobacteriales |
| OTU1534 | Bacteria | Proteobacteria | Gammaproteobacteria | Enterobacteriales |
| OTU1535 | Bacteria | Verrucomicrobia | Opitutae | [Cerasicoccales] |
| OTU1536 | Bacteria | Proteobacteria | Gammaproteobacteria | Vibrionales |
| OTU1537 | Bacteria | Proteobacteria | Gammaproteobacteria | Pseudomonadales |
| OTU1538 | Bacteria | Verrucomicrobia | Opitutae | Opitutales |
| OTU1539 | Bacteria | Actinobacteria | Actinobacteria | Corynebacteriales |
| OTU1540 | Archaea | Euryarchaeota | Halobacteria | Halobacteriales |
| OTU1541 | Bacteria | Proteobacteria | Gammaproteobacteria | Vibrionales |
| OTU1542 | Bacteria | Proteobacteria | Gammaproteobacteria | Aeromonadales |
| OTU1543 | Archaea | Euryarchaeota | Halobacteria | Halobacteriales |
| OTU1544 | Bacteria | Proteobacteria | Deltaproteobacteria | Desulfobacterales |
| OTU1545 | Bacteria | Proteobacteria | Gammaproteobacteria | Aeromonadales |
| OTU1546 | Bacteria | Firmicutes | Bacilli | Lactobacillales |
| OTU1547 | Bacteria | Firmicutes | Clostridia | Clostridiales |
| OTU1548 | Bacteria | Proteobacteria | Gammaproteobacteria | Alteromonadales |
| OTU1549 | Bacteria | Proteobacteria | Alphaproteobacteria | Rhizobiales |
| OTU1550 | Bacteria | Proteobacteria | Gammaproteobacteria | Alteromonadales |
| OTU1551 | Bacteria | Verrucomicrobia | [Pedosphaerae] | [Pedosphaerales] |
| OTU1552 | Archaea | Euryarchaeota | Halobacteria | Halobacteriales |
| OTU1553 | Bacteria | Bacteroidetes | Flavobacteriia | Flavobacteriales |
| OTU1554 | Bacteria | Proteobacteria | Gammaproteobacteria | Aeromonadales |
| OTU1555 | Bacteria | Proteobacteria | Gammaproteobacteria | Oceanospirillales |
| OTU1556 | Bacteria | Proteobacteria | Gammaproteobacteria | Pseudomonadales |
| OTU1557 | Archaea | Euryarchaeota | Halobacteria | Halobacteriales |
| OTU1558 | Bacteria | Proteobacteria | Betaproteobacteria | Burkholderiales |
| OTU1559 | Bacteria | Actinobacteria | Actinobacteria | Micrococcales |
| OTU1560 | Bacteria | Proteobacteria | Gammaproteobacteria | Alteromonadales |
| OTU1561 | Archaea | Euryarchaeota | Halobacteria | Halobacteriales |
| OTU1562 | Bacteria | Proteobacteria | Gammaproteobacteria | Pseudomonadales |
| OTU1563 | Bacteria | Firmicutes | Bacilli | Bacillales |
| OTU1564 | Bacteria | Proteobacteria | Gammaproteobacteria |  |
| OTU1565 | Bacteria | Proteobacteria | Gammaproteobacteria | Pseudomonadales |
| OTU1566 | Bacteria | Firmicutes | Bacilli | Bacillales |
| OTU1567 | Bacteria | Proteobacteria | Gammaproteobacteria | Pseudomonadales |
| OTU1568 | Bacteria | Firmicutes | Bacilli | Bacillales |
| OTU1569 | Bacteria | Firmicutes | Bacilli | Bacillales |
| OTU1570 | Bacteria | Verrucomicrobia | Verrucomicrobiae | Verrucomicrobiales |
| OTU1571 | Archaea | Euryarchaeota | Halobacteria | Halobacteriales |
| OTU1572 | Bacteria | Proteobacteria | Gammaproteobacteria | Vibrionales |
| OTU1573 | Archaea | Euryarchaeota | Halobacteria | Halobacteriales |
| OTU1574 | Archaea | Euryarchaeota | Halobacteria | Halobacteriales |
| OTU1575 | Bacteria | Proteobacteria | Alphaproteobacteria | Rhizobiales |
| OTU1576 | Archaea | Euryarchaeota | Halobacteria | Halobacteriales |
| OTU1577 | Bacteria | Proteobacteria | Alphaproteobacteria | Rhodospirillales |
| OTU1578 | Archaea | Euryarchaeota | Halobacteria | Halobacteriales |
| OTU1579 | Archaea | Euryarchaeota | Halobacteria | Halobacteriales |
| OTU1580 | Bacteria | Proteobacteria | Gammaproteobacteria | Oceanospirillales |
| OTU1581 | Bacteria | Proteobacteria | Gammaproteobacteria | Pasteurellales |
| OTU1582 | Bacteria | Fusobacteria | Fusobacteriia | Fusobacteriales |
| OTU1583 | Archaea | Euryarchaeota | Halobacteria | Halobacteriales |
| OTU1584 | Bacteria | Verrucomicrobia | [Spartobacteria] | [Chthoniobacterales] |
| OTU1585 | Archaea | Euryarchaeota | Halobacteria | Halobacteriales |
| OTU1586 | Bacteria | Verrucomicrobia | Verruco-5 | LD1-PB3 |
| OTU1587 | Bacteria | Proteobacteria | Gammaproteobacteria |  |
| OTU1588 | Bacteria | Firmicutes | Bacilli | Bacillales |
| OTU1589 | Bacteria | Proteobacteria | Gammaproteobacteria | Aeromonadales |
| OTU1590 | Bacteria | Proteobacteria | Gammaproteobacteria | Pseudomonadales |
| OTU1591 | Archaea | Euryarchaeota | Halobacteria | Halobacteriales |
| OTU1592 | Bacteria | Firmicutes | Bacilli | Bacillales |
| OTU1593 | Bacteria | Bacteroidetes | Bacteroidia | Bacteroidales |
| OTU1594 | Bacteria | Firmicutes | Bacilli | Bacillales |
| OTU1595 | Bacteria | Firmicutes | Bacilli | Bacillales |
| OTU1596 | Bacteria | Firmicutes | Bacilli | Turicibacterales |
| OTU1597 | Bacteria | Proteobacteria | Gammaproteobacteria | Enterobacteriales |
| OTU1598 | Bacteria | Firmicutes | Bacilli | Bacillales |
| OTU1599 | Bacteria | Proteobacteria | Gammaproteobacteria | Pseudomonadales |
| OTU1600 | Bacteria | Bacteroidetes | Bacteroidia | Bacteroidales |
| OTU1601 | Bacteria | Proteobacteria | Gammaproteobacteria | Pseudomonadales |
| OTU1602 | Bacteria | Proteobacteria | Gammaproteobacteria | Vibrionales |
| OTU1603 | Bacteria | Verrucomicrobia | [Spartobacteria] | [Chthoniobacterales] |
| OTU1604 | Bacteria | Proteobacteria | Gammaproteobacteria |  |
| OTU1605 | Bacteria | Proteobacteria | Gammaproteobacteria | Alteromonadales |
| OTU1606 | Bacteria | Bacteroidetes | Flavobacteriia | Flavobacteriales |
| OTU1607 | Bacteria | Proteobacteria | Alphaproteobacteria | Rhodobacterales |
| OTU1608 | Bacteria | Proteobacteria | Gammaproteobacteria | Alteromonadales |
| OTU1609 | Bacteria | Verrucomicrobia | [Spartobacteria] | [Chthoniobacterales] |
| OTU1610 | Bacteria | Firmicutes | Bacilli | Lactobacillales |
| OTU1611 | Bacteria | Actinobacteria | Actinobacteria | Micrococcales |
| OTU1612 | Archaea | Euryarchaeota | Halobacteria | Halobacteriales |
| OTU1613 | Bacteria | Firmicutes | Bacilli | Bacillales |
| OTU1614 | Archaea | Euryarchaeota | Halobacteria | Halobacteriales |
| OTU1615 | Bacteria | Synergistetes | Synergistia | Synergistales |
| OTU1616 | Bacteria | Firmicutes | Clostridia | Clostridiales |
| OTU1617 | Bacteria | Proteobacteria | Gammaproteobacteria | Oceanospirillales |
| OTU1618 | Bacteria | Proteobacteria | Gammaproteobacteria | Vibrionales |
| OTU1619 | Archaea | Euryarchaeota | Halobacteria | Halobacteriales |
| OTU1620 | Bacteria | Synergistetes | Synergistia | Synergistales |
| OTU1621 | Archaea | Euryarchaeota | Halobacteria | Halobacteriales |
| OTU1622 | Archaea | Euryarchaeota | Halobacteria | Halobacteriales |
| OTU1623 | Bacteria | Proteobacteria | Alphaproteobacteria | Rickettsiales |
| OTU1624 | Bacteria | Firmicutes | Bacilli | Bacillales |
| OTU1625 | Bacteria | Firmicutes | Clostridia | Clostridiales |
| OTU1626 | Archaea | Euryarchaeota | Halobacteria | Halobacteriales |
| OTU1627 | Bacteria | Firmicutes | Bacilli | Bacillales |
| OTU1628 | Bacteria | Firmicutes | Bacilli | Bacillales |
| OTU1629 | Bacteria | Firmicutes | Bacilli | Lactobacillales |
| OTU1630 | Bacteria | Proteobacteria | Alphaproteobacteria | Rhizobiales |
| OTU1631 | Bacteria | Firmicutes | Clostridia | Clostridiales |
| OTU1632 | Bacteria | Proteobacteria | Gammaproteobacteria | Pseudomonadales |
| OTU1633 | Archaea | Euryarchaeota | Halobacteria | Halobacteriales |
| OTU1634 | Bacteria | Actinobacteria | Actinobacteria | Micrococcales |
| OTU1635 | Bacteria | Proteobacteria | Gammaproteobacteria | Aeromonadales |
| OTU1636 | Bacteria | Firmicutes | Bacilli | Bacillales |
| OTU1637 | Bacteria | Proteobacteria | Alphaproteobacteria | BD7-3 |
| OTU1638 | Archaea | Euryarchaeota | Halobacteria | Halobacteriales |
| OTU1639 | Bacteria | Proteobacteria | Deltaproteobacteria | Desulfobacterales |
| OTU1640 | Bacteria | Proteobacteria | Gammaproteobacteria | Pseudomonadales |
| OTU1641 | Bacteria | Firmicutes | Bacilli | Bacillales |
| OTU1642 | Bacteria | OP3 | koll11 |  |
| OTU1643 | Bacteria | OP3 | koll11 | GIF10 |
| OTU1644 | Bacteria | OP3 | BD4-9 |  |
| OTU1645 | Bacteria | Proteobacteria | Gammaproteobacteria | Vibrionales |
| OTU1646 | Bacteria | Actinobacteria | Actinobacteria | Pseudonocardiales |
| OTU1647 | Bacteria | Proteobacteria | Gammaproteobacteria | Pseudomonadales |
| OTU1648 | Bacteria | Proteobacteria | Gammaproteobacteria | Vibrionales |
| OTU1649 | Bacteria | Actinobacteria | Coriobacteriia | Coriobacteriales |
| OTU1650 | Bacteria | Firmicutes | Bacilli | Bacillales |
| OTU1651 | Bacteria | Proteobacteria | Gammaproteobacteria | Alteromonadales |
| OTU1652 | Bacteria | Proteobacteria | Alphaproteobacteria | Rhizobiales |
| OTU1653 | Bacteria | Proteobacteria | Gammaproteobacteria | Vibrionales |
| OTU1654 | Bacteria | Firmicutes | Bacilli | Bacillales |
| OTU1655 | Bacteria | Proteobacteria | Alphaproteobacteria | Sphingomonadales |
| OTU1656 | Archaea | Euryarchaeota | Halobacteria | Halobacteriales |
| OTU1657 | Archaea | Euryarchaeota | Halobacteria | Halobacteriales |
| OTU1658 | Archaea | Euryarchaeota | Halobacteria | Halobacteriales |
| OTU1659 | Archaea | Euryarchaeota | Halobacteria | Halobacteriales |
| OTU1660 | Bacteria | OP3 | koll11 | GIF10 |
| OTU1661 | Bacteria | Proteobacteria | Gammaproteobacteria | Enterobacteriales |
| OTU1662 | Bacteria | Proteobacteria | Alphaproteobacteria | Rhizobiales |
| OTU1663 | Bacteria | Proteobacteria | Gammaproteobacteria |  |
| OTU1664 | Bacteria | Actinobacteria | Actinobacteria | Actinomycetales |
| OTU1665 | Bacteria | Proteobacteria | Gammaproteobacteria | Oceanospirillales |
| OTU1666 | Archaea | Euryarchaeota | Halobacteria | Halobacteriales |
| OTU1667 | Bacteria | Proteobacteria | Betaproteobacteria | Burkholderiales |
| OTU1668 | Bacteria | Proteobacteria | Gammaproteobacteria | Vibrionales |
| OTU1669 | Bacteria | Firmicutes | Bacilli | Bacillales |
| OTU1670 | Bacteria | Proteobacteria | Gammaproteobacteria | Pseudomonadales |
| OTU1671 | Bacteria | Bacteroidetes | [Saprospirae] | [Saprospirales] |
| OTU1672 | Bacteria | Proteobacteria | Gammaproteobacteria | Vibrionales |
| OTU1673 | Bacteria | Actinobacteria | Coriobacteriia | Coriobacteriales |
| OTU1674 | Archaea | Euryarchaeota | Halobacteria | Halobacteriales |
| OTU1675 | Bacteria | Proteobacteria | Gammaproteobacteria | Alteromonadales |
| OTU1676 | Bacteria | Proteobacteria | Gammaproteobacteria | Alteromonadales |
| OTU1677 | Archaea | Euryarchaeota | Halobacteria | Halobacteriales |
| OTU1678 | Bacteria | Firmicutes | Bacilli | Bacillales |
| OTU1679 | Bacteria | Proteobacteria | Gammaproteobacteria |  |
| OTU1680 | Bacteria | OP8 | OP8_1 | HMMVPog-54 |
| OTU1681 | Bacteria | Proteobacteria | Betaproteobacteria | Burkholderiales |
| OTU1682 | Bacteria | Firmicutes | Bacilli | Bacillales |
| OTU1683 | Bacteria | Bacteroidetes | Flavobacteriia | Flavobacteriales |
| OTU1684 | Archaea | Euryarchaeota | Halobacteria | Halobacteriales |
| OTU1685 | Archaea | Euryarchaeota | Halobacteria | Halobacteriales |
| OTU1686 | Bacteria | Proteobacteria | Gammaproteobacteria | Alteromonadales |
| OTU1687 | Archaea | Euryarchaeota | Halobacteria | Halobacteriales |
| OTU1688 | Bacteria | Proteobacteria | Gammaproteobacteria | Vibrionales |
| OTU1689 | Bacteria | Proteobacteria | Gammaproteobacteria | Oceanospirillales |
| OTU1690 | Bacteria | Planctomycetes |  |  |
| OTU1691 | Bacteria | Firmicutes | Bacilli | Bacillales |
| OTU1692 | Bacteria | Proteobacteria | Gammaproteobacteria |  |
| OTU1693 | Bacteria | Firmicutes | Bacilli | Bacillales |
| OTU1694 | Bacteria | Firmicutes | Clostridia | Halanaerobiales |
| OTU1695 | Bacteria | Bacteroidetes | [Rhodothermi] | [Rhodothermales] |
| OTU1696 | Bacteria | Proteobacteria | Alphaproteobacteria | Rickettsiales |
| OTU1697 | Bacteria | Acidobacteria | iii1-8 | DS-18 |
| OTU1698 | Bacteria | Acidobacteria | iii1-8 | SJA-36 |
| OTU1699 | Bacteria | Spirochaetes | [Leptospirae] | [Leptospirales] |
| OTU1700 | Bacteria | Chloroflexi | Thermomicrobia | JG30-KF-CM45 |
| OTU1701 | Bacteria | Firmicutes | Erysipelotrichi | Erysipelotrichales |
| OTU1702 | Bacteria | Firmicutes | Bacilli | Bacillales |
| OTU1703 | Bacteria | Acidobacteria | OS-K |  |
| OTU1704 | Bacteria | Proteobacteria | Gammaproteobacteria | Pseudomonadales |
| OTU1705 | Archaea | Euryarchaeota | Halobacteria | Halobacteriales |
| OTU1706 | Bacteria | Proteobacteria | Alphaproteobacteria | Rhizobiales |
| OTU1707 | Bacteria | Firmicutes | Bacilli | Bacillales |
| OTU1708 | Bacteria | Proteobacteria | Gammaproteobacteria | Enterobacteriales |
| OTU1709 | Bacteria | Proteobacteria | Gammaproteobacteria | Vibrionales |
| OTU1710 | Bacteria | Proteobacteria | Gammaproteobacteria | Vibrionales |
| OTU1711 | Bacteria | Firmicutes | Clostridia | Clostridiales |
| OTU1712 | Bacteria | Acidobacteria | Holophagae | Holophagales |
| OTU1713 | Bacteria | Proteobacteria | Gammaproteobacteria | Pseudomonadales |
| OTU1714 | Bacteria | Proteobacteria | Gammaproteobacteria | Pseudomonadales |
| OTU1715 | Archaea | Euryarchaeota | Halobacteria | Halobacteriales |
| OTU1716 | Archaea | Euryarchaeota | Halobacteria | Halobacteriales |
| OTU1717 | Archaea | Euryarchaeota | Halobacteria | Halobacteriales |
| OTU1718 | Archaea | Euryarchaeota | Halobacteria | Halobacteriales |
| OTU1719 | Archaea | Euryarchaeota | Halobacteria | Halobacteriales |
| OTU1720 | Bacteria | Proteobacteria | Gammaproteobacteria | Vibrionales |
| OTU1721 | Archaea | Euryarchaeota | Halobacteria | Halobacteriales |
| OTU1722 | Bacteria | Proteobacteria | Gammaproteobacteria | Vibrionales |
| OTU1723 | Archaea | Euryarchaeota | Halobacteria | Halobacteriales |
| OTU1724 | Bacteria | Proteobacteria | Gammaproteobacteria | Salinisphaerales |
| OTU1725 | Archaea | Euryarchaeota | Halobacteria | Halobacteriales |
| OTU1726 | Archaea | Euryarchaeota | Halobacteria | Halobacteriales |
| OTU1727 | Archaea | Euryarchaeota | Halobacteria | Halobacteriales |
| OTU1728 | Bacteria | Bacteroidetes | [Rhodothermi] | [Rhodothermales] |
| OTU1729 | Bacteria | Proteobacteria | Gammaproteobacteria | Pseudomonadales |
| OTU1730 | Bacteria | Proteobacteria | Gammaproteobacteria |  |
| OTU1731 | Bacteria | Proteobacteria | Gammaproteobacteria | Alteromonadales |
| OTU1732 | Bacteria | Proteobacteria | Gammaproteobacteria | Vibrionales |
| OTU1733 | Bacteria | Proteobacteria | Gammaproteobacteria | Pseudomonadales |
| OTU1734 | Bacteria | Proteobacteria | Gammaproteobacteria | Alteromonadales |
| OTU1735 | Archaea | Euryarchaeota | Halobacteria | Halobacteriales |
| OTU1736 | Bacteria | Firmicutes | Bacilli | Bacillales |
| OTU1737 | Bacteria | Firmicutes | Bacilli | Bacillales |
| OTU1738 | Bacteria | Proteobacteria | Gammaproteobacteria | Aeromonadales |
| OTU1739 | Bacteria | Proteobacteria | Gammaproteobacteria |  |
| OTU1740 | Archaea | Euryarchaeota | Halobacteria | Halobacteriales |
| OTU1741 | Bacteria | Proteobacteria | Alphaproteobacteria | Kiloniellales |
| OTU1742 | Bacteria | Firmicutes | Bacilli | Bacillales |
| OTU1743 | Bacteria | Proteobacteria | Gammaproteobacteria | Vibrionales |
| OTU1744 | Bacteria | Proteobacteria | Gammaproteobacteria | Oceanospirillales |
| OTU1745 | Archaea | Euryarchaeota | Halobacteria | Halobacteriales |
| OTU1746 | Bacteria | Proteobacteria | Gammaproteobacteria | Enterobacteriales |
| OTU1747 | Bacteria | Proteobacteria | Gammaproteobacteria |  |
| OTU1748 | Bacteria | Firmicutes | Bacilli | Bacillales |
| OTU1749 | Archaea | Euryarchaeota | Halobacteria | Halobacteriales |
| OTU1750 | Bacteria | Acidobacteria | [Chloracidobacteria] | RB41 |
| OTU1751 | Bacteria | Firmicutes | Bacilli | Bacillales |
| OTU1752 | Bacteria | Firmicutes | Bacilli | Bacillales |
| OTU1753 | Archaea | Euryarchaeota | Halobacteria | Halobacteriales |
| OTU1754 | Bacteria | Proteobacteria | Gammaproteobacteria | Pseudomonadales |
| OTU1755 | Archaea | Euryarchaeota | Halobacteria | Halobacteriales |
| OTU1756 | Bacteria | Proteobacteria | Gammaproteobacteria | Alteromonadales |
| OTU1757 | Archaea | Euryarchaeota | Halobacteria | Halobacteriales |
| OTU1758 | Bacteria | Proteobacteria | Gammaproteobacteria | Oceanospirillales |
| OTU1759 | Bacteria | Firmicutes | Bacilli | Lactobacillales |
| OTU1760 | Bacteria | Proteobacteria | Gammaproteobacteria | Vibrionales |
| OTU1761 | Archaea | Euryarchaeota | Halobacteria | Halobacteriales |
| OTU1762 | Bacteria | Proteobacteria | Gammaproteobacteria | Vibrionales |
| OTU1763 | Bacteria | Proteobacteria | Gammaproteobacteria | Vibrionales |
| OTU1764 | Bacteria | Bacteroidetes | Sphingobacteriia | Sphingobacteriales |
| OTU1765 | Bacteria | Proteobacteria | Gammaproteobacteria | Vibrionales |
| OTU1766 | Bacteria | Firmicutes | Bacilli | Bacillales |
| OTU1767 | Bacteria | Proteobacteria |  |  |
| OTU1768 | Archaea | Euryarchaeota | Halobacteria | Halobacteriales |
| OTU1769 | Bacteria | Proteobacteria | Gammaproteobacteria |  |
| OTU1770 | Archaea | Euryarchaeota | Halobacteria | Halobacteriales |
| OTU1771 | Bacteria | Firmicutes | Bacilli | Bacillales |
| OTU1772 | Bacteria | Proteobacteria | Gammaproteobacteria | Oceanospirillales |
| OTU1773 | Bacteria | Firmicutes | Clostridia | Clostridiales |
| OTU1774 | Bacteria | Proteobacteria | Gammaproteobacteria | Pseudomonadales |
| OTU1775 | Bacteria | Proteobacteria | Betaproteobacteria | Burkholderiales |
| OTU1776 | Bacteria | Actinobacteria | Actinobacteria | Micrococcales |
| OTU1777 | Bacteria | Firmicutes | Bacilli | Bacillales |
| OTU1778 | Bacteria | Proteobacteria | Gammaproteobacteria |  |
| OTU1779 | Bacteria | Bacteroidetes | Bacteroidia | Bacteroidales |
| OTU1780 | Bacteria | Firmicutes | Bacilli | Lactobacillales |
| OTU1781 | Bacteria | Actinobacteria | Actinobacteria | Corynebacteriales |
| OTU1782 | Bacteria | Proteobacteria | Gammaproteobacteria | Aeromonadales |
| OTU1783 | Bacteria | Proteobacteria | Gammaproteobacteria | Pseudomonadales |
| OTU1784 | Bacteria | Proteobacteria | Alphaproteobacteria | Sphingomonadales |
| OTU1785 | Bacteria | Firmicutes | Bacilli | Bacillales |
| OTU1786 | Bacteria | Proteobacteria | Gammaproteobacteria | Enterobacteriales |
| OTU1787 | Bacteria | Acidobacteria | Acidobacteria-6 | iii1-15 |
| OTU1788 | Bacteria | Firmicutes | Bacilli | Bacillales |
| OTU1789 | Bacteria | Proteobacteria | Betaproteobacteria | Burkholderiales |
| OTU1790 | Bacteria | Firmicutes | Clostridia | Clostridiales |
| OTU1791 | Bacteria | Proteobacteria | Gammaproteobacteria | Vibrionales |
| OTU1792 | Bacteria | Firmicutes | Bacilli | Bacillales |
| OTU1793 | Archaea | Euryarchaeota | Halobacteria | Halobacteriales |
| OTU1794 | Bacteria | Proteobacteria | Gammaproteobacteria | Pseudomonadales |
| OTU1795 | Bacteria | Firmicutes | Bacilli | Bacillales |
| OTU1796 | Bacteria | Bacteroidetes | Bacteroidia | Bacteroidales |
| OTU1797 | Bacteria | Acidobacteria | Acidobacteria-6 | iii1-15 |
| OTU1798 | Bacteria | Acidobacteria | BPC102 | B110 |
| OTU1799 | Bacteria | Acidobacteria | Acidobacteria-6 | iii1-15 |
| OTU1800 | Archaea | Euryarchaeota | Halobacteria | Halobacteriales |
| OTU1801 | Bacteria | Firmicutes | Bacilli | Bacillales |
| OTU1802 | Bacteria | Proteobacteria | Gammaproteobacteria | Pseudomonadales |
| OTU1803 | Bacteria | Firmicutes | Bacilli | Bacillales |
| OTU1804 | Archaea | Euryarchaeota | Halobacteria | Halobacteriales |
| OTU1805 | Bacteria | Firmicutes | Bacilli | Bacillales |
| OTU1806 | Bacteria | Proteobacteria | Gammaproteobacteria | Pseudomonadales |
| OTU1807 | Bacteria | Firmicutes | Bacilli | Lactobacillales |
| OTU1808 | Bacteria | Proteobacteria | Gammaproteobacteria | Pseudomonadales |
| OTU1809 | Bacteria | Proteobacteria | Alphaproteobacteria | Sphingomonadales |
| OTU1810 | Bacteria | Actinobacteria | Actinobacteria | Corynebacteriales |
| OTU1811 | Bacteria | Actinobacteria | Actinobacteria | Actinomycetales |
| OTU1812 | Bacteria | Firmicutes | Bacilli | Bacillales |
| OTU1813 | Bacteria | Proteobacteria | Gammaproteobacteria | Cardiobacteriales |
| OTU1814 | Archaea | Euryarchaeota | Halobacteria | Halobacteriales |
| OTU1815 | Bacteria | Firmicutes | Bacilli | Bacillales |
| OTU1816 | Bacteria | Actinobacteria | Actinobacteria | Micrococcales |
| OTU1817 | Bacteria | Firmicutes | Bacilli | Bacillales |
| OTU1818 | Archaea | Euryarchaeota | Halobacteria | Halobacteriales |
| OTU1819 | Bacteria | TM7 |  |  |
| OTU1820 | Archaea | Euryarchaeota | Halobacteria | Halobacteriales |
| OTU1821 | Bacteria | Firmicutes | Bacilli | Bacillales |
| OTU1822 | Archaea | Euryarchaeota | Halobacteria | Halobacteriales |
| OTU1823 | Bacteria | Proteobacteria | Gammaproteobacteria | Alteromonadales |
| OTU1824 | Bacteria | Proteobacteria | Gammaproteobacteria | Vibrionales |
| OTU1825 | Bacteria | Proteobacteria | Gammaproteobacteria | Pseudomonadales |
| OTU1826 | Bacteria | Acidobacteria | Acidobacteria-6 | iii1-15 |
| OTU1827 | Bacteria | Firmicutes | Bacilli | Bacillales |
| OTU1828 | Bacteria | Firmicutes | Bacilli | Bacillales |
| OTU1829 | Bacteria | Proteobacteria | Gammaproteobacteria | Oceanospirillales |
| OTU1830 | Bacteria | Proteobacteria | Gammaproteobacteria | Enterobacteriales |
| OTU1831 | Bacteria | Acidobacteria | Acidobacteria-6 | iii1-15 |
| OTU1832 | Bacteria | Bacteroidetes | Bacteroidia | Bacteroidales |
| OTU1833 | Bacteria | Acidobacteria | Acidobacteria-6 | iii1-15 |
| OTU1834 | Bacteria | Proteobacteria | Gammaproteobacteria | Enterobacteriales |
| OTU1835 | Archaea | Euryarchaeota | Halobacteria | Halobacteriales |
| OTU1836 | Archaea | Euryarchaeota | Halobacteria | Halobacteriales |
| OTU1837 | Bacteria | Firmicutes | Bacilli | Lactobacillales |
| OTU1838 | Archaea | Euryarchaeota | Halobacteria | Halobacteriales |
| OTU1839 | Archaea | Euryarchaeota | Halobacteria | Halobacteriales |
| OTU1840 | Archaea | Euryarchaeota | Halobacteria | Halobacteriales |
| OTU1841 | Archaea | Euryarchaeota | Halobacteria | Halobacteriales |
| OTU1842 | Bacteria | Firmicutes | Bacilli | Lactobacillales |
| OTU1843 | Bacteria | Proteobacteria | Betaproteobacteria | Burkholderiales |
| OTU1844 | Archaea | Euryarchaeota | Halobacteria | Halobacteriales |
| OTU1845 | Bacteria | Deferribacteres | Deferribacteres | Deferribacterales |
| OTU1846 | Bacteria | Firmicutes | Bacilli | Bacillales |
| OTU1847 | Bacteria | Proteobacteria | Gammaproteobacteria | Vibrionales |
| OTU1848 | Archaea | Euryarchaeota | Halobacteria | Halobacteriales |
| OTU1849 | Bacteria | Proteobacteria | Gammaproteobacteria | Vibrionales |
| OTU1850 | Bacteria | Deferribacteres | Deferribacteres | Deferribacterales |
| OTU1851 | Archaea | Euryarchaeota | Halobacteria | Halobacteriales |
| OTU1852 | Bacteria | Deferribacteres | Deferribacteres | Deferribacterales |
| OTU1853 | Bacteria | Proteobacteria | Gammaproteobacteria | Xanthomonadales |
| OTU1854 | Bacteria | Chrysiogenetes | Chrysiogenetes | Chrysiogenales |
| OTU1855 | Bacteria | Actinobacteria | Actinobacteria | Pseudonocardiales |
| OTU1856 | Bacteria | Proteobacteria | Gammaproteobacteria | Pseudomonadales |
| OTU1857 | Bacteria | Bacteroidetes | Bacteroidia | Bacteroidales |
| OTU1858 | Bacteria | Cyanobacteria | Oscillatoriophycideae | Chroococcales |
| OTU1859 | Bacteria | Proteobacteria | Gammaproteobacteria | Alteromonadales |
| OTU1860 | Bacteria | Proteobacteria | Gammaproteobacteria |  |
| OTU1861 | Archaea | Euryarchaeota | Halobacteria | Halobacteriales |
| OTU1862 | Bacteria | Bacteroidetes | Bacteroidia | Bacteroidales |
| OTU1863 | Bacteria | Proteobacteria | Gammaproteobacteria | Alteromonadales |
| OTU1864 | Bacteria | Firmicutes | Bacilli | Bacillales |
| OTU1865 | Bacteria | Proteobacteria |  |  |
| OTU1866 | Bacteria | Proteobacteria | Gammaproteobacteria | Vibrionales |
| OTU1867 | Bacteria | Proteobacteria | Gammaproteobacteria |  |
| OTU1868 | Bacteria | Firmicutes | Bacilli | Bacillales |
| OTU1869 | Bacteria | Proteobacteria | Deltaproteobacteria | Myxococcales |
| OTU1870 | Bacteria | Firmicutes | Bacilli | Bacillales |
| OTU1871 | Bacteria | Firmicutes | Bacilli | Bacillales |
| OTU1872 | Bacteria | Actinobacteria | Actinobacteria | Micrococcales |
| OTU1873 | Bacteria | Proteobacteria | Gammaproteobacteria |  |
| OTU1874 | Bacteria | Actinobacteria | Actinobacteria | Actinomycetales |
| OTU1875 | Bacteria | Proteobacteria | Gammaproteobacteria | Vibrionales |
| OTU1876 | Bacteria | Bacteroidetes | [Rhodothermi] | [Rhodothermales] |
| OTU1877 | Archaea | Euryarchaeota | Halobacteria | Halobacteriales |
| OTU1878 | Bacteria | Firmicutes | Bacilli | Bacillales |
| OTU1879 | Bacteria | Bacteroidetes | Bacteroidia | Bacteroidales |
| OTU1880 | Archaea | Euryarchaeota | Halobacteria | Halobacteriales |
| OTU1881 | Bacteria | Firmicutes | Bacilli | Bacillales |
| OTU1882 | Archaea | Euryarchaeota | Halobacteria | Halobacteriales |
| OTU1883 | Archaea | Euryarchaeota | Halobacteria | Halobacteriales |
| OTU1884 | Bacteria | Proteobacteria | Gammaproteobacteria | Aeromonadales |
| OTU1885 | Bacteria | Bacteroidetes | Bacteroidia | Bacteroidales |
| OTU1886 | Bacteria | Actinobacteria | Actinobacteria | Bifidobacteriales |
| OTU1887 | Bacteria | Proteobacteria | Alphaproteobacteria | Rhizobiales |
| OTU1888 | Bacteria | Bacteroidetes | Bacteroidia | Bacteroidales |
| OTU1889 | Bacteria | Bacteroidetes | Bacteroidia | Bacteroidales |
| OTU1890 | Bacteria | Bacteroidetes | Bacteroidia | Bacteroidales |
| OTU1891 | Bacteria | Bacteroidetes | Bacteroidia | Bacteroidales |
| OTU1892 | Bacteria | Proteobacteria | Gammaproteobacteria | Enterobacteriales |
| OTU1893 | Bacteria | Bacteroidetes | Bacteroidia | Bacteroidales |
| OTU1894 | Bacteria | Proteobacteria | Gammaproteobacteria | Pseudomonadales |
| OTU1895 | Archaea | Euryarchaeota | Halobacteria | Halobacteriales |
| OTU1896 | Archaea | Euryarchaeota | Halobacteria | Halobacteriales |
| OTU1897 | Archaea | Euryarchaeota | Halobacteria | Halobacteriales |
| OTU1898 | Bacteria | Firmicutes | Bacilli | Lactobacillales |
| OTU1899 | Bacteria | Proteobacteria | Gammaproteobacteria | Vibrionales |
| OTU1900 | Bacteria | Actinobacteria | Actinobacteria | Micrococcales |
| OTU1901 | Bacteria | Proteobacteria | Gammaproteobacteria | Pseudomonadales |
| OTU1902 | Bacteria | Bacteroidetes | Bacteroidia | Bacteroidales |
| OTU1903 | Bacteria | Proteobacteria | Gammaproteobacteria | Alteromonadales |
| OTU1904 | Bacteria | Proteobacteria | Gammaproteobacteria |  |
| OTU1905 | Bacteria | Firmicutes | Bacilli | Bacillales |
| OTU1906 | Bacteria | Proteobacteria | Gammaproteobacteria | Enterobacteriales |
| OTU1907 | Bacteria | Bacteroidetes | Flavobacteriia | Flavobacteriales |
| OTU1908 | Bacteria | Firmicutes | Bacilli | Bacillales |
| OTU1909 | Archaea | Euryarchaeota | Halobacteria | Halobacteriales |
| OTU1910 | Bacteria | Bacteroidetes | Bacteroidia | Bacteroidales |
| OTU1911 | Bacteria | Proteobacteria | Gammaproteobacteria | Pseudomonadales |
| OTU1912 | Bacteria | Firmicutes | Bacilli | Lactobacillales |
| OTU1913 | Bacteria | Proteobacteria | Gammaproteobacteria | Alteromonadales |
| OTU1914 | Bacteria | Proteobacteria | Gammaproteobacteria |  |
| OTU1915 | Bacteria | Proteobacteria | Gammaproteobacteria | Vibrionales |
| OTU1916 | Bacteria | Proteobacteria | Gammaproteobacteria | Pseudomonadales |
| OTU1917 | Bacteria | Bacteroidetes | Bacteroidia | Bacteroidales |
| OTU1918 | Bacteria | Firmicutes | Bacilli | Bacillales |
| OTU1919 | Bacteria | Bacteroidetes | Bacteroidia | Bacteroidales |
| OTU1920 | Bacteria | Proteobacteria | Gammaproteobacteria | Pseudomonadales |
| OTU1921 | Archaea | Euryarchaeota | Methanobacteria | Methanobacteriales |
| OTU1922 | Bacteria | Firmicutes | Bacilli | Lactobacillales |
| OTU1923 | Archaea | Euryarchaeota | Halobacteria | Halobacteriales |
| OTU1924 | Bacteria | Bacteroidetes | Bacteroidia | Bacteroidales |
| OTU1925 | Bacteria | Proteobacteria | Alphaproteobacteria | Rhodospirillales |
| OTU1926 | Bacteria | Proteobacteria | Alphaproteobacteria | Kiloniellales |
| OTU1927 | Bacteria | Bacteroidetes | Bacteroidia | Bacteroidales |
| OTU1928 | Bacteria | Proteobacteria | Gammaproteobacteria | Alteromonadales |
| OTU1929 | Bacteria | Proteobacteria | Gammaproteobacteria | Vibrionales |
| OTU1930 | Bacteria | Bacteroidetes | Bacteroidia | Bacteroidales |
| OTU1931 | Bacteria | Bacteroidetes | Bacteroidia | Bacteroidales |
| OTU1932 | Archaea | Euryarchaeota | Halobacteria | Halobacteriales |
| OTU1933 | Archaea | Euryarchaeota | Halobacteria | Halobacteriales |
| OTU1934 | Bacteria | Firmicutes | Clostridia | Clostridiales |
| OTU1935 | Bacteria | Bacteroidetes | Bacteroidia | Bacteroidales |
| OTU1936 | Bacteria | Bacteroidetes | Bacteroidia | Bacteroidales |
| OTU1937 | Archaea | Euryarchaeota | Halobacteria | Halobacteriales |
| OTU1938 | Archaea | Euryarchaeota | Halobacteria | Halobacteriales |
| OTU1939 | Bacteria | Firmicutes | Bacilli | Bacillales |
| OTU1940 | Bacteria | Actinobacteria | Actinobacteria | Micrococcales |
| OTU1941 | Bacteria | Firmicutes | Clostridia | Clostridiales |
| OTU1942 | Bacteria | Proteobacteria | Gammaproteobacteria | Pseudomonadales |
| OTU1943 | Bacteria | Proteobacteria | Gammaproteobacteria |  |
| OTU1944 | Bacteria | Proteobacteria | Gammaproteobacteria | Vibrionales |
| OTU1945 | Archaea | Euryarchaeota | Halobacteria | Halobacteriales |
